# Poster Presentations - Abstracts of the 27^th^ Annual Congress of the SBRA. Aracaju/SE - Brazil, 2023

**DOI:** 10.5935/1518-0557.20230071

**Published:** 2024

**P-01. Shared egg donation: comparison of pregnancy rates in fresh or thawn embryo transfers between donors and recipients**

**Vinicius Alves de Oliveira^1^, Rodopiano de Souza Florencio^1^, Waldermar Naves do Amaral^2^**

*^1^ Humana Medicina Reprodutiva - Goiânia - GO - Brasil.*

*^2^ Grupo Fértile - Goiânia - GO - Brasil.*

**Objective:** Determine the chances of clinical pregnancy and live births in donors and recipients in the first fresh or thawed embryo transfer.

**Methods:** Retrospective, cross-sectional study where 211 egg donation cycles and 211 egg reception cycles were studied, from January 2016 to December 2020, at the Humana Medicina Reprodutiva Clinic, in Goiânia - GO. The study investigated pregnancy-related rates in donors and recipients, as well as evaluating pregnancy rates in fresh and thawed embryo transfers. Variables that could interfere with the chance of pregnancy in both donors and recipients were also evaluated.

**Results:** Between donors and recipients there was no statistical difference for clinical pregnancy (*p=*0.7585) and live births (*p*=0.5786). The index of clinical pregnancy and live births in donors showed no statistical difference (*p*=0.6701 and *p=*0.2056) for fresh and thawed transfers. There was also no statistical difference for recipient patients. Among the variables studied that could interfere with the results, only the blastulation index showed a statistical difference in the chance of clinical pregnancy in donors and recipients (tale 1).

**Conclusion:** The studied variables that could interfere in the results showed statistical difference only for the blastulation index in both donors and recipients. The clinical pregnancy rate in the donors was 37.70% and 36.10% in the recipients. There was no difference in the chances of pregnancy between donors and recipients, and there was also no difference in pregnancy rates between fresh and thawed transfers. The results showed no statistical difference in clinical pregnancy and live births when comparing D4 versus D5-D6 transfers.

**Table 1:** Pregnancy results in donors: 1^st^ transfer, fresh or thawed.

	Fresh (113)	Thawed (86)	P
Biochemical pregnancy	4/113 (3.53%)	5/86 (5.81%)	0.5041
Clinical pregnancy	41/113 (39.82%)	34/86 (39.53%)	0.6601
Implantation	51/267 (19.10%)	40/182 (21.97%)	0.4747
Clinical abortion	13/41 (31.70%)	5/34 (14.70%)	0.1078
Live births	28/113 (24.77%)	29/86 (33.72%)	0.2056
Multiple pregnancy	5/28 (17.85%)	6/29 (20.68%)	1.0000
Ectopic pregnancy	1/113 (0.88%)	0	1.0000

**P-02. Alloimmune abortion and the immunopathological mechanisms through a literature review**

**Manuella Amlid Pimenta de Castro Cavalcanti Silva^1^, Alexandre Antônio de Lima Júnior^1^, Fálba Bernadete Ramos dos Anjos^1^, Adriana Fracasso^2^, Karollyne Skarlet Gomes da Silva^1^, Evandro Valentim da Silva^1^**

*^1^Universidade Federal de Pernambuco - Recife - PE - Brasil.*

*^2^Faculdade Integrada de Pernambuco - Recife - PE - Brasil.*

**Objective:** Perform a literature review on what alloimmune abortion is and what specific characteristics can cause alloimmune abortion.

**Methods:** For this review, searches were conducted using Pubmed, Google Scholar, and Scielo, using the keywords: 'alloimmune abortion,' 'in vitro fertilization,' and 'assisted reproduction.' The research criteria included selecting articles in both English and Portuguese, published between the years 2004 and 2022.

**Results:** Abortion is a public health problem and one of the leading causes of maternal morbidity and mortality (MARIUTTI, 2009). Abortions are considered cases occurring before 20 weeks of gestation, with the fetus measuring less than 16 centimeters and weighing less than 500 grams (MINISTRY OF HEALTH, 2005). There are different types of abortions, such as chemical abortions resulting from abortive medications, and spontaneous abortions resulting from organic disorders that occur in the second or third month of pregnancy (MARIUTTI, 2009). Alloimmune abortion falls under spontaneous abortions, characterized by recurrent cases, involving three or more consecutive spontaneous losses and representing approximately 40% to 60% of spontaneous abortion cases (CAETANO, 2006).

There is a difference between autoimmune factors and alloimmune factors. Autoimmune factors are correlated with the action of autoantibodies in the presence of the embryo, influenced by autoimmune factors such as anti-thyroglobulin (TGO) and anti-nuclear factor (FAN). On the other hand, alloimmune factors are correlated with maternal rejection due to the presence of paternal genetic material from the embryo, with Natural Killer cells (NK) being involved as alloimmune factors. Alloimmune abortion is a result of blastocyst implantation, leading to a highly complex and specialized immune response involving both the embryo and the endometrium (THIENGO). The blastocyst is covered by the trophoblast, a cellular layer responsible for the formation of the embryonic placenta (MOORE and PERSAUD, 2008). The trophoblast acts as a semi-allograft, as a part of the genetic material comes from the father, which is unknown to the maternal immune system, increasing the chances of rejection. Some of the immune mediators involved are cytokines, and the maternal endometrium expresses cytokines essential for blastocyst implantation. Trophoblastic cells express human leukocyte antigens (HLA), ensuring maternal-fetal immunotolerance by interacting with NK cells. HLA-G plays a vital role in preventing cell lysis induced by NK cells by interacting with inhibitory death receptors (KIRs), preventing NK cell activation (THIENGO). Recent studies have indicated a correlation between Natural Killer cells (NK) and alloimmune abortion (NIEKERK, 2013). Some studies associate a high concentration of NK cells in the endometrium and peripheral blood with a higher risk of recurrent abortion, while other results suggest that patients undergoing in vitro fertilization (IVF) with a history of recurrent abortion should be investigated for the presence of factors associated with loss. IVF could reduce recurrent abortion cases by up to 29% (BILIBIO, 2020).

**Conclusion:** Alloimmune abortion is one of the main causes of spontaneous abortion, but it still requires further study as its causes are not yet fully understood. The most probable cause is the presence of NK cells that cause cell lysis, contributing to the failure of blastocyst implantation. IVF is a method that needs further improvement to ensure better results in blastocyst implantation, as one of the reasons for IVF failure is the maternal immune response's deficiency, affecting processes from blastocyst nidation to placental formation, thus preventing the progression of pregnancy. This indicates that it is essential to study the mechanisms of immune-modulatory tolerance that occur in natural gestation to replicate them in assisted reproduction.

**P-03. Influence of maternal age on the blastocysts rate and implantation associated with the number of oocytes**

**Karen Melissa Goncalves Oliveira^1^, Aline de Cássia Azevedo^1^, Manuela Baldave Carli Dias^1^, Lilian Pagano Mori^1^, Vitor Armenio Scontre^1^, Luis Roberto Louzada Balducci^1^, Edson Guimarães Lo Turco^2^, Bernardo Rodrigues Lamounier de Moura^2^**

*^1^InVentre Centro Avançado de Medicina Reprodutiva - Santo André - SP - Brasil.*

*^2^Embriológica - São Paulo - SP - Brasil.*

**Objective:** Analyze maternal age related to the number of recovered oocytes as a prognosis factor directly associated with the results of in vitro fertilization treatments.

**Methods:** Was performed observational, retrospective and descriptive study was carried out, in which the medical records of patients who underwent In Vitro Fertilization (IVF) treatment during the period from January 2018 to September 2022 were evaluated, carried out at the InVentre Assisted Reproduction Clinic, located in Santo André in the state of São Paulo. The variables and proportions analyzed were: number of oocytes collected, mature oocytes, fertilization rate, number of blastocysts, number of transfers and implantation rate.

**Results:** 150 autologous IVF cycles were evaluated. Three groups of different maternal ages were compared: up to 35 years old (67 cycles), 36-39 years old (50 cycles) and 40 years old or more (33 cycles).

No significant differences were found in the rates of mature oocytes and fertilization between the different groups.

The average number of oocytes recovered and of embryos that reached the blastocyst stage was significantly lower (*p*<0.05) in the group of patients aged 40 years or older, when compared to the other two younger groups.

The implantation rate after embryo transfer (fresh or frozen-thawed cycle) was significantly higher (*p*<0.05) in the groups of women who underwent treatment before 40 years of age. In the groups of patients up to 35 years old and 36-39 years old, the implantation rate was 68.5% and 52.4%, respectively, while in patients aged 40 years or more, this rate was 36%.

In addition, the existence of a high relationship between the number of oocytes and blastocysts was observed, so that for each 1 more oocyte produced by each patient, regardless of the group, there was a mean increase of 0.304 blastocysts (95% confidence interval).

Finally, it was observed that for every 1 more blastocyst available for transfer, the probability of implantation increased 1.198 times (95% confidence interval), while for every 1 year of age, this probability decreased 0.909 times (interval of 95% confidence).

**Conclusion:** In conclusion, the number of recovered oocytes, degree of maturation and fertilization rate did not show significant differences between the groups, however, based on the analyzed samples, the rate of embryos that reached the blastocyst stage and resulted in an implantation after embryo transfer was significantly lower in patients aged over 40 years. This idea reinforces that a woman's age is an important indicator of the success rate of assisted reproductive technology.

**P-04. Chemically defined 3D matrix for in vitro maturation (IVM) of human oocytes**

**Adriana Bos-Mikich^1^, Gabriella Mamede Andrade^2^, Luis Alberto Loureiro dos Santos^1^, Nilo Frantz^2^**

*^1^ Universidade Federal do Rio Grande do Sul - Porto Alegre - RS - Brasil.*

*^2^ Nilo Frantz Medicina Reprodutiva - Porto Alegre - RS - Brasil.*

**Objective:** We reported earlier the use of a nontoxic PVA-based hydrogel modified with BTCA, for fibroblasts and stem cells culture. The objective of the present study is to test the use of a PVA-based/BTCA gel for the culture of human immature oocytes to improve in vitro conditions that may enhance present oocyte maturation rates. Also, there is the need to find easily manufactured, safe, and economical materials for human IVM, particularly for fertility preservation in cancer patients.

**Methods:** Immature human oocytes and cumulus cells were obtained with informed consent from patients undergoing conventional IVF cycles after superovulation and hCG priming. Cumulus cells and oocytes were cultured in CSC medium supplemented with 10% SSS in a 10% PVA (Mw 130,000, 99% degree of hydrolysis, Sigma Chemical Co., St. Louis, MO) and BTCA (1,2,3,4-butanetetracarboxylic acid, Sigma Chemical Co., St. Louis, MO, 99%) matrix for 24hrs. PVA was added to 30 mL deionized water at 100ºC. After dissolution, 0.3g of BTCA was added and stirred for 20 minutes at 60ºC.The solution was placed in polystyrene Petri dishes. Films were obtained by solvent evaporation. Cumulus cells obtained before hyaluronidase exposure were placed in culture onto PVA-BTCA hydrogel for cytotoxicity and cell attachment test. Eleven GV and 4 denuded oocytes and cumulus cells were added to PVA-BTCA culture system for IVM, at 38ºC and 5%CO2

**Results:** Two replicates of cumulus cells exposure to PVA-BTCA culture system showed that the hydrogel allows for cell survival and adhesion to the substrate, after gentle swirling of the culture dish and change of the medium with the unattached cells, as observed under contrast phase microscopy. Having confirmed the hydrogels supported cell survival, 11 GV and 4 MI oocytes obtained from five different patients were added to the culture system together with cumulus cells isolated from oocytes of the same women. Eight GV and 2 MI oocytes reached MII (73% and 50%, respectively), after 24h culture in the PVA-BTCA /cumulus cells culture system.

**Conclusion:** The PVA/BTCA gel represents a promising new approach to improve IVM culture conditions, particularly for Fertility Preservation. The system may enhance the number of mature oocytes collected after superovulation or during ovarian tissue fragmentation for cryopreservation, when small follicles and immature oocytes are released. PVA-BTCA culture system may also be used for individual follicle growth.

**P-05. Autologous platelet-rich plasma for thin endometrium treatment: a case report**

**Ana Carolina de Souza Mangrich^1^, Thomas Gabriel Miklos^1^**

*^1^ Santa Casa de São Paulo - São Paulo - SP - Brasil.*

**Objective:** The aim of this case report is to share a satisfactory outcome on the use of intrauterine infusion of PRP (platelet-rich plasma) in a patient with thin endometrium and refractoriness to other therapies.

**Methods:** Data from medical records were used to base this case report, as well as scientific studies and other case reports published worldwide. PubMed and Embase were the data base used for the literature review.

**Results:** Patient J.O.R.C, 41 years-old, female, diagnosed with premature ovarian failure, married to E.A.C., male, 40 years-old, healthy and with an unchanged spermogram. The couple had a frozen blastocyst stored, product of an intracytoplasmic sperm injection (ICSI) carried out with egg donation and E.A.C.’s sperm. During the cycle, endometrium was permanently thin and some attempts to thicken were made with both estrogen gel and low-dose aspirin, achieving the maximum thickness of 5mm. Administration of intrauterine PRP was attempted twice in the same cycle, with the gap of three days, in order to magnify the chances of a satisfactory outcome. Endometrial thickness was 7mm by the time of the transfer, after two intrauterine infusions of PRP. The stored frozen blastocyst was transferred, with the outcome of a healthy pregnancy and a child born and alive.

**Conclusion:** Assisted reproduction treatments (ART) may sometimes be challenging. Unsatisfactory endometrial growth (less than 7mm) is considered a bad prognostic factor, with lower chances of a successful implantation and pregnancy. Some embryo transfer cycles are cancelled due to a thin endometrium, leading to a extended treatment and increasing the anxiety which is inherent in the process. Numerous treatment modalities have been proposed, such as estrogen gel, low-dose aspirin, vitamin E, vaginal sildenafil and G-CSF (granulocyte-colony stimulating factor) intrauterine infusion. Nevertheless, in addition to the weak evidence level of those therapies, some patients remain non-responsive. PRP made with autologous blood plasma was tested in some pilot studies with a satisfactory response. It is already used for nerve repair and for multiple regeneration therapies in the Orthopaedic field, but in Gynaecology, studies with a higher number of patients are still needed in order to enrich evidence. It seems to work on proliferation and migration of endometrial cells, also enhancing the expression of regeneration and repairing factors and increasing local blood supply. Studies suggest a higher success rate in IVF (in-vitro fertilization) with the use of PRP, which could be a suitable alternative in cases of low-response to other methods. In addition, there is no risk of contracting infectious diseases or activating the immune system - the blood plasma enriched with platelets is autologous.

**P-06. Treatment of cervical ectopic pregnancy with methotrexate in a patient with recurrent ectopic pregnancy: a case report**

**Gaia Costa Pou^1^, Juan Carlos Pou Florentino^1^**

*^1^ Gaia Centro de Reprodução Assistida - Criciúma - SC - Brasil.*

**Objective:** To evaluate the efficacy and safety of the treatment of cervical ectopic pregnancy with the use of methotrexate (MTX) in a patient with recurrent ectopic pregnancy.

**Methods:** Report of a patient who had three ectopic pregnancies, the last being a cervical ectopic pregnancy. The diagnosis was made based on clinical examination and confirmed by ultrasonography, which identified the presence of a gestational sac with an embryo in the cervix. The treatment adopted for this patient was the use of MTX, a drug that interferes with the growth and development of embryonic cells, inducing the termination of ectopic pregnancy. In this case, the administration of MTX was chosen as a non-surgical alternative, aiming to avoid invasive procedures and preserve the patient's fertility. Regular follow-ups of beta-hCG levels to prepregnancy values were performed.

**Results:** Cervical ectopic pregnancy is regarded as an exceptional location, accounting for less than 1% of ectopic pregnancies. Although rare, this is the most serious and dramatic obstetric pathology. E.V.M., a 28-year-old female patient with no previous history of pathology sought gynecological care due to complaints of infertility after six years of unsuccessful attempts. The patient reports having had two miscarriages in the year 2020, the first being an ectopic pregnancy in May 2019, which required a right salpingectomy, and the second an abortion with curettage performed in August 2019 at 5 weeks gestation. Despite the previous abortions, the patient had no changes on physical and gynecological examination. However, laboratory tests revealed altered anti-cardiolipin antibody and the presence of positive antinuclear factor, indicating possible immunological or autoimmune disorders. In June 2022, the patient returned to the clinic to undergo an in vitro fertilization cycle. After the transfer of two devitrified embryos, the patient presented with a twin pregnancy, with one intrauterine gestational sac and the second right ectopic gestational sac with a positive fetal heartbeat. As a result, the patient needed to undergo a right total salpingectomy to remove the ectopic gestational sac. The intrauterine pregnancy proceeded without complications, resulting in a full-term delivery. In March 2022, the patient returned to the service with the desire to perform a second embryo transfer. However, this pregnancy resulted in an abortion. In April 2022, the patient returned again for her last embryo transfer of her last embryo, resulting in a single pregnancy with cervical implantation. The treatment performed to induce the abortion was with three cycles of MTX. After the use of the medication, there was a regression of beta-hCG to pre-pregnancy values, indicating the resolution of the cervical ectopic pregnancy.

**Conclusion:** The use of MTX is an effective alternative treatment for cervical ectopic pregnancy. This non-surgical approach has the advantage of preserving the patient's fertility while avoiding the need for invasive procedures.

**P-07. Implantation potential when transferring morula stage embryos**

**Andrea Mesquita Lima^1^, Eduardo Gomes Sá^1^, Fábio Eugenio Magalhaes Rodrigues^1^, Marcus Aurelio Bessa Paiva^1^, Sebastiao Evangelista Torquato^1^, Tulius Augustus Ferreira Freitas^1^, Ellayne Cavalcanti Queiroz^1^, Renata Reis Pimentel Castro^1^, Gleicyane Sousa Santos Alam^1^**

*^1^BIOS - Fortaleza - CE - Brasil.*

**Objective:** To evaluate the implantation rate when embryo transfer was performed at the morula stage.

Methods: 30 cases were analyzed, from February 2022 to May 2023, in which 30 transfers were performed, with an average of 1.7 embryos per transfer. The mean age of the patients was 37.7 years. In the study, only transfers with embryos in the morula stage were considered.

**Results:** Among the analyzed cases, there was a 48% implantation rate for transfers performed on D4. Patients younger than 36 years had a higher implantation rate (66.7%) when compared to the group of patients older than 36 years (35.2%), who also transferred morulas, regardless of the number of embryos transferred. The embryos had been previously vitrified on the third day of culture after ICSI (INTRA CYTOPLASMIC SPERM INJECTION), and the transfer was performed 24 hours after devitrification.

**Conclusion:** The transfer of embryos in the morula stage, that is, in the stage that precedes the blastocyst stage, can be considered a viable option, as it demonstrates good results. It is noteworthy that the conduct regarding the stage of the embryo for transfer is a clinical decision, which should take into account the couple's history, based on individual circumstances.

**P-08. The importance of the Quality Management program in an Assisted Reproduction clinic**

**Andrea Mesquita Lima^1^, Sebastião Evangelista Torquato^1^, Fabio Eugenio Magalhães Rodrigues^1^, Marcus Aurelio Bessa Paiva^1^, Tulius Augustus Ferreira Freitas^1^, Socorro Maria Pontes Parente Torquato^1^, Louise Studart Figueiredo^1^**

*^1^BIOS - Fortaleza - CE - Brasil.*

**Objective:** On December 26, 2022, the new Collegiate Board Resolution (RDC) No. 771 was published, which provides for Good Practices in Germ Cells, Germ Tissues and Human Embryos for therapeutic use. This new Resolution replaces RDC No. 23, of May 27, 2011. One of the main changes in this new RDC was the need to implement a QMS (quality management system) in Assisted Human Reproduction (AHR) clinics. The objective of this work was to evaluate the points that showed improvements after the implementation of a QMS in the clinic.

**Methods:** After the publication of the new RDC 771, in December 2022, the implementation of the QMS at the BIOS Clinic began. The clinic's quality management system is still being implemented, and in the present study some points initially implemented were evaluated, and in parallel, the improvements that the changes brought about were analyzed.

**Results:** A thorough validation of the equipment, following a specific standard operating protocol (SOP), demonstrated that their useful life improved considerably, a fact that minimized costs and avoided fluctuations in working conditions, providing a better environment for patient samples. A quality control inspection of the materials and means used, with the implementation of standardized protocols before releasing them for use, increased the safety and reliability of the technical team in using a given lot. The control of non-conformities was a differentiating issue, because when management encountered any non-compliance, this was duly documented, and in the event of an imminent recurrence, a solution was promptly found, so that non-compliance could be avoided, carrying out preventive measures in a timely manner, when necessary. The QMS began to play a key role in ensuring patient safety, which involved establishing an adequate sample tracking system, maintaining ideal laboratory conditions for handling gametes and embryos, with due prevention of cross-contamination and due compliance with biosafety practices, with strict serological screening, meeting all the requirements of the new RDC. The implementation of the QMS solidified its quest for continuous improvement, which involved reviewing and analyzing performance indicators, identifying areas for improvement, and monitoring results.

**Conclusion:** The quality management system plays an extremely important role within an assisted human reproduction clinic. The new Resolution of the collegiate board RDC 771 came to emphasize the complexity of the processes involved in an in vitro fertilization treatment. A quality management system is essential to ensure process quality assurance, and in addition to reducing costs, it minimizes failures and maximizes process efficiency.

**P-09. The Correlation between KIDScore™ generated for each embryo grown in the Embryoscope Plus incubator and body mass index of patients undergoing assisted human recovery treatments**

**Andrea Mesquita Lima^1^, Sebastião Evangelista Torquato Filho^2^, Tulius Augustus Ferreira Freitas^1^, Gleicyane Sousa Santos Alam^1^, Eduardo Gomes Sá^1^, Renata Reis Pimentel Castro^1^, Ellayne Cavalcanti Queiroz^1^**

*^1^BIOS - Fortaleza - CE - Brasil.*

*^2^Sollirium - Fortaleza - CE - Brasil.*

**Objective:** The aim of this study was to correlate the score of KIDScore (artificial intelligence software, which provides a score for embryos grown in the Embryoscope Plus incubator) with the BMI of patients undergoing in vitro fertilization treatment.

**Methods:** From August 2022 to May 2023, 91 patients were analyzed, a total of 277 embryos. Patients were divided into 4 groups: Group 1 (low weight); Group 2 (normal weight); Group 3 (overweight) and Group 4 (obesity). All analyzed embryos were cultured in the Embryoscope incubator, so that they could be evaluated by KIDScore, an artificial intelligence software, which provides a score for the analyzed embryos.

**Results:** Patients in groups 1 and 4 received the lowest scores according to the KIDScore analysis. Patients in Group 3 received intermediate scores, and patients in Group 2 received the highest score.

**Conclusion:** Thus, there is an influence of BMI on embryonic quality, with a significant difference in the values of the scores received by the embryos by the artificial intelligence program of Embryocope Plus, KIDScore.

**P-10. Adenomyosis - the Radiologist's Perspective**

**Francisco Sardinha^1^, Catarina Janicas^2^, Teresa Margarida Cunha^3^**

*^1^ Hospital Garcia de Orta - Portugal.*

*^2^ Centro Hospitalar de Lisboa Ocidental - Portugal.*

*^3^ Instituto Português de Oncologia de Lisboa Francisco Gentil - Portugal.*

**Objective:** This poster presents an overview of adenomyosis from the radiologist's perspective, emphasizing the significance of non-invasive diagnostic techniques. It offers an in-depth analysis of transvaginal ultrasound (TVUS) and magnetic resonance imaging (MRI) findings, reviews their respective attributes, and discusses potentially associated conditions.

**Methods:** Adenomyosis is a benign condition characterized by the presence of heterotopic endometrial tissue within the myometrium, most probably due to invagination of the basal endometrial layer. These heterotopic endometrial cells continuously express high levels of estrogen receptors and apoptosis-suppressing mediators, leading to smooth muscle cell hypertrophy and hyperplasia. The prevalence of adenomyosis is highly variable due to demographic factors and non-uniform diagnostic criteria. Nevertheless, several risk factors, such as estrogen exposure, parity, and prior uterine surgery, have been identified. Patients most frequently present with menorrhagia, dysmenorrhea, and chronic pelvic pain, as well as increased risk of infertility and adverse pregnancy outcomes, including miscarriage. Given its significant prevalence and associated complications, determining an accurate non-invasive diagnostic approach is paramount. The accuracy of TVUS and MRI in diagnosing adenomyosis has been demonstrated, exhibiting specificities of 78% and 88%, respectively, and an equal sensitivity of 78%. However, since TVUS is highly operator-dependant and these results are primarily derived from expert-led studies, its performance is possible overestimated. Notwithstanding, TVUS widespread accessibility, lower cost, and shorter acquisition times make it the preferred initial imaging method. MRI, on the other hand, is mainly used as a secondary diagnostic technique due to its higher cost, longer acquisition times, and potential discomfort for certain patients, namely with claustrophobia. Yet, it remains relevant, as its superior soft tissue contrast resolution and multiplanar reconstructions allow for a better definition of the junctional zone and the diagnosis of potential estrogen-dependent conditions, such as fibroids and endometriosis. Using illustrative images from our institution's TVUS and MRI scans, we showcase a variety of adenomyosis manifestations discernible through both imaging methods.

**Results:** Multiple authors have proposed classification systems for reporting adenomyosis on TVUS and MRI. However, they have yet to achieve validation and universal acceptance. Our approach emphasizes a classification from a histopathological perspective, distinguishing signs as either direct, correlating to ectopic endometrial foci, or indirect, corresponding to secondary myometrial hypertrophy. Direct signs tend to increase specificity, while indirect signs enhance diagnostic sensitivity. We detail and depict the distinct imaging manifestations and signs, establishing a correlation between TVUS and MRI.

**Conclusion:** Over the last few decades, advancements in non-invasive imaging techniques have facilitated the diagnosis of adenomyosis without requiring a surgical-histopathological procedure. This progress has enhanced physicians' understanding of the true incidence of adenomyosis within the general population and its clinical manifestations. Concurrently, comprehending the key imaging findings is vital for performing a non-invasive adenomyosis diagnosis. Understanding these findings' histopathological background and diagnostic value allows for better interpretation, identifying potential pitfalls, and detecting frequently associated conditions. The resulting comprehensive imaging assessment not only detects adenomyosis, but also guides the therapeutic strategy.

**P-11. Does elevated progesterone on day of trigger associate with blastocyst ploidy in egg donor cycles?**

**Priscilla Lopes Caldeira^1^, Aline Rodrigues Lorenzon^2^, Ana Paula Aquino^2^, Bruna Barros^2^, Eduardo Leme Motta^2^, Thais Sanches Domingues Curry^2^, Pedro Augusto Araujo Monteleone^1^, Edmund Chada Baracat^1^, Jose Maria Soares Junior^1^**

*^1^ Faculdade de Medicina da USP - São Paulo - SP - Brasil.*

*^2^ Huntington - São Paulo - SP - Brasil.*

**Objective:** Evaluate if high progesterone levels on day of trigger influences blastocyst ploidy and laboratory outcomes in egg donors cycles.

**Methods:** This retrospective cohort study, performed between April/2013 and February/2019 at Huntington Medicina Reprodutiva Clinic, analyzed laboratory data from ICSI cycles using frozen donated oocytes that underwent embryo biopsy (PGT-A) at blastocyst stage. Patients were divided into two groups according to serum progesterone (P4) on trigger day: group A if P4 <1.5 ng/mL and group B if P4 ≥1.5 ng/mL. Only euploid embryos were transfer on FET cycles. Primary outcome was embryo euploidy and aneuploidy rate. Secondary outcomes were number of blastocysts, number of top quality embryos, number of euploid/aneuploid embryos and clinical pregnancy rate. These parameters were compared between groups A and B. T and Fisher tests were used for statistical analysis.

**Results:** 259 egg donors cycles with PGT-A were analyzed. Group A included 75 cycles (57 donors; 69 recipients) and group B 184 cycles (115 donors; 163 recipients). Group A donors`mean age was 25.15±3.59 and group B 24.46±3.73 years old (*p*=0.19). Total gonadotropins used and basal FSH were not different between the two groups (2777.83±526.02 vs 2814.54±538.78, *p*=0.7363; 5.19±1.51 vs 5.22±1.47, *p*=0.3035). Group B had a significantly higher number of antral follicular count (22.95±10.65 vs 19.60±7.08, *p*= 0.0301) and estradiol at trigger (5255.00±6405.77 vs 5252.83±4346.33 vs, *p*=0.0340). Number of eggs retrieved and number of MII were higher in group B than A (33.96±1.76 vs 28.01±11.78, *p*=0.0014; 24.84±11.74 vs 21.12±10.56, *p*=0.0082). There were no difference between groups in oocytes post-thaw survival rate and fertilization rate (0.95±0.10 vs 0.98±0.17, *p*=0.2626; 0.83±0.14 vs 0.82±0.13, *p*=0.854). The number of blastocysts (3.60±1.52 vs 3.68±1.52, *p*=0.667), number of top-quality embryos (2.27±1.59 vs 2.28±1.43, *p*=0.802), number of euploid embryos (1.92±1.25 vs 1.92±1.13, *p*=0.954) and number of aneuploid embryos (1.23±1.01 vs 1.14±0.94, *p*=0.593) were not significantly different between the two groups. Euploid embryo rate (0.31±0.20 vs 0.30±0.18, *p*=0.626) and aneuploidy embryo rate (0.21±0.19 vs 0.18±0.15, *p*=0.436) did not significantly differ. There was also no difference in clinical pregnancy rate (0.73 vs 0.82, *p*=0.476). Seminal parameters were similar between groups.

**Conclusion:** Our data showed that elevated progesterone levels on trigger’s day is not associated with blastocyst aneuploidy rates or worst laboratory outcomes in egg donor’s cycles, in which the cofounder of maternal age is excluded from the analysis.

**P-12. Impact of the use of the Zymot plate on the outcomes of Assisted Reproduction procedures at an in vitro fertilization clinic in Northeastern Brazil**

**Renata Reis Pimentel Castro^1^, Andrea Mesquita Lima^1^, Sebastião Evangelista Torquato^1^, Tulius Augustus Ferreira Freitas^1^, Gleicyane Sousa Santos Alam^1^, Fábio Eugênio Magalhães Rodrigues^1^, Marcus Aurélio Bessa Paiva^1^**

*^1^BIOS - Centro de Medicina Reprodutiva do Ceará - Fortaleza - CE - Brasil.*

**Objective:** To analyze and relate the use of the Zymot technique in assisted reproduction procedures, as well as its influence on parameters such as the rate of fertilized oocytes and quality of embryonic development, associating these data with the performance indicators of the Vienna Consensus.

**Methods:** This is a retrospective study that used a pre-formed data sheet from a private Assisted Reproduction clinic in northeastern Brazil, using data from October 2020 to June 2023, totaling 69 cases, 1 of which was excluded from the research because calcium was used in the eggs. The mean age of women was between 38 years and normal weight, and the mean age of men was between 46 years and overweight. The seminal processing technique used in all cases was the Zymot.

**Results:** After analyzing the data, it was possible to observe that the results of the parameters of the rates of the clinical indicators using the Zymot technique to process the semen, in order to obtain spermatozoa with better motility and with low levels of sperm DNA fragmentation, presented a good correlation with the quality indicators of the Vienna Consensus. The fertilization rate was 74.8%, the cleavage rate was 100%, and blastulation was 57.9%.

**Conclusion:** Female age may have an influence on the maturity rate parameter, while our findings suggest that male age has a greater impact on sperm DNA fragmentation and due to this, the Zymot technique was used to minimize fragmentation and this way to improve the quality of fertilization and embryonic development. Thus, we can state that the rates of the indicators were close with the use of the Zymot technique when compared with the rates of the Vienna Consensus.

**P-13. The relationship between KIDScore™ score generated for each embryo and implantation rates**

**Gleicyane Sousa dos Santos Alam^1^, Andrea Mesquita Lima^1^, Renata Reis Pimentel de Castro^1^, Eduardo Gomes Sá^1^, Ellayne Cavalcanti^1^, Sebastião Evangelista Torquato^1^, Tulius Augusto de Freitas^1^**

*^1^Sollirium Health Group - Fortaleza - CE - Brasil*

**Objective:** The advancement of artificial intelligence within Assisted Human Reproduction is quite remarkable, and the implementation of Time-lapse technology added more values on the development and evaluation of the quality of embryos, without compromising the culture conditions. Due to the high amount of data generated for each embryo, several predictive implantation models based on morphokinetic parameters were developed. Among these models, KIDScore™ Day 5 stands out, considered a predictive model of implantation after embryonic transfer on the fifth day, developed for Embryoscope™ devices. The KIDScore™ system assigns a score that increases with the embryo's implantation potential, and aims to classify embryos from the same cohort to select the one with the greatest implantation potential for transfer. This study aims to present the relationship between the scores generated by KIDScore™ and the implantation rates for embryo transfer cases.

**Methods:** 180 patients who transferred 1 or 2 embryos from February 2022 to November 2022 were analyzed. The KIDScore™ score of each embryo was recorded and, based on these scores, comparisons were made with the implantation rates. Inclusion criteria to determine implantation rates were: positive Beta HCG result, presence or absence of a gestational sac, and fetal heartbeat. The results of genetic analysis of transferred embryos were considered exclusion criteria for this study.

**Results:** The grades presented on KIDScore day 5 range from 0 to 9.9. Of the embryos analyzed in the study, there was no pattern of notes, as a variation between the Scores was observed, but without significantly affecting the result of the implantation rates. Embryos with high scores, such as 9.5, 8.7, 7.7, showed high implantation rates, but also embryos with low scores, 6.4, 5.8, 4.2, obtained satisfactory results in relation to the implantation rate. However, some parameters were observed, such as: the transfer of 2 embryos, when an embryo has a Score much lower than the first, the result of positive Beta HCG was reported, and the presence of a gestational sac, with a single embryo and with heartbeats heart defects, which may predict that the embryo with the lowest score did not have implantation potential compared to the one with the highest score. About 5% of the analyzed cases that transferred two embryos, the twin pregnancy was confirmed, with the observation that the scores of the embryos had notes with very close values. It was also described in 10% of cases confirmation of positive Beta HCG and presence of gestational sac, but absence of heartbeats, being observed that there is no relationship with a KIDScore™ grade pattern, as the grades of these specific cases vary between 4.5 and 9.1.

**Conclusion:** After analyzing the KIDScore™ scores for the transferred embryos compared with the implantation rates, it is possible to understand that the score that the predictive method determines for each embryo may not interfere with the implantation, since in cases of low scores there were satisfactory implantation results, although it was also observed that the higher the grade, the greater the chance of implantation. A highlight would be the fact of transferring two embryos, which, one has a lower score than the other, the chance of only the one with the highest score implanting is greater than the two embryos implanting.

**P-14. Correlation between the number of pregnancies and abortions and the cause of infertility in patients at a public center for Assisted Human Reproduction (RHA) in Brazil**

**Uaska Bezerra e Silva^1^, Lívia Christina Alves da Silva^1^, José Roberto Izídio Lopes^1^, Pryscila Cynara Soares Vieira^1^, Hosana Fausto de Sousa^1^, Mychelle de Medeiros Garcia Torres^2^, Kelley Rossana do Nascimento Barbosa^1^, Carla Simone Medeiros Silva^1^, Marcela Queiroz Lopes de Melo Martins^1^, Beatriz de Araújo Cruz^2^, Edualeide Jeane Pereira Bulhoes da Nóbrega^2^**

*^1^ EBSERH - Natal - RN - Brasil.*

*^2^ UFRN-Universidade Federal do Rio Grande do Norte - Natal - RN - Brasil.*

**Objective:** To analyze the correlation between the number of pregnancies and abortions and the infertility factor of couples who underwent assisted human reproduction (AHR) procedures in a public center in Brazil.

**Methods:** The cause of infertility and the female age group of 155 couples who had pregnancy by Human Artificial Insemination (HAI) and Intracytoplasmic Sperm Injection (ICSI) between December 2013 and March 2023 and the incidence of missbirth in 123 couples were analyzed. who obtained pregnancy between the years 2013 to 2018 by RHA procedures, according to the cause of infertility and female age group.

**Results:** The highest number of ICSI pregnancies occurred among couples whose cause of infertility was male factor (75), followed by tubal factor (53), ovarian factor (22), endometriosis (14), smaller numbers were found for infertility without apparent cause (ISCA) (5), female age factor (3) and female hypogonadotropic hypogonadism (1). Greater numbers of pregnancies by HAI occurred in couples with ISCA (14), followed by ovarian factor (4), male factor (3) and endometriosis (1). There was no occurrence of abortions in patients who underwent HAI. The number of abortions in couples who underwent ICSI was higher for those with infertility factor male (11) and tubal (11), followed by ovarian factor (4) and female age factor. The female age group with the highest incidence of pregnancy was 31 to 37 years (52%), followed by ≥ 38 years (30%) and ≤ 30 years (18%). And the incidence of abortion accompanied the number of pregnancies, being higher in the female age group from 31 to 37 years (55%), followed by the group ≥ 38 years (32%) and ≤ 30 years (14%).

**Conclusion:** A greater number of pregnancies by ICSI were identified in couples whose cause of infertility was a male factor. Thus, the analysis proves what is already evidenced in the literature, that ICSI has allowed couples in which the partner has some seminal alteration to develop a pregnancy that would not be possible naturally. The second highest number of pregnancies was identified in couples with tubal factor as the cause of infertility, this result was expected because, in vitro fertilization allows external fertilization, this overcomes the limitations of tubal alterations. Pregnancies by HAI occurred in greater numbers in couples with an undetermined cause of infertility. An important result to be emphasize is absent of abortion among couples who underwent HAI between the years 2013 to 2018.In the same period, the highest abortion rate was correlated with the male infertility factor, once to the low genetic quality of spermatozoa can induce miscarriage. The female age group with the highest incidence of pregnancies and also abortions was between 31 and 37 years, significantly higher proportion than for the group of 38 years or older. For patients aged 30 years or less, the number of pregnancies was lower, mainly due to the low demand for AHR treatments in this age group. This work presented an overview of the number of pregnancies and abortions related to the infertility factor and female age group within a group of patients who underwent AHR treatment at a Center of the public helth system.

**Table 1:** Pregnancy outcomes.

	< 35 years			≥ 35 years		
	**Biopsy** **(n=40)**	**Control** **(n=161)**	***p* value**	**Biopsy** **(n=91)**	**Control** **(n=91)**	***p* value**
Implantation rate	53%	53%	0.8679	56%	38%^**^	**0.0064**
β-hCG positive	23(58%)	124(77%)^*^	**0.0168**	54(59%)	53(58%)	1.0000
Chemical miscarriage	2(9%)	12(10%)	1.0000	3(6%)	1(2%)	0.6179
Clinical pregnancy	21(53%)	112(70%)	0.0607	51(56%)	52(57%)	1.0000
Clinical miscarriage	2(9%)	24(19%)	0.3706	15(28%)	5(9%)^*^	**0.0241**
Live birth rate	19(48%)	88(55%)	0.4802	36(40%)	47(52%)	0.1365

^*^P<0.05 *was considered statistically significant*.

**P-15. Non-conformances management in IVF laboratories**

**Patricia Giffron Rodrigues^1^, Rodopiano Souza Florêncio^1^**

*^1^ Humana Medicina Reprodutiva - Goiânia - GO - Brasil.*

**Objective:** The purpose of this study is to guide laboratory teams to structure effective approaches to managing non-conformances.

**Methods:** Analyzes were performed from the NCBI and Pubmed database. The search keywords were “quality management in IVF laboratory”, “IVF errors”, “non-conformance in IVF laboratory”, “cost of non-conformance”, “risk assessment in laboratory” and “corrective action”.

**Results:** The scarcity of published data regarding non-conformances shows that the phrase “to err is human” may not be applicable in the IVF (in vitro fertilization) context. This lack of information is directly associated with the scenario of the infertility treatment journey itself, which tends to be challenging, stressful and decisive for families. In this circumstance, the activities in IVF laboratories must be performed in a standard way, with monitoring and control.

The literature portrays that the standardization of procedures drastically reduces the possibilities of error, in this sense, the concept of non-conformance was established as the non-fulfillment of a previously established requirement. In general, two types of non-conformances can occur in the IVF laboratory: active errors and latent errors. Active errors are generated by insecurity that can cause harm to patients or the system, such as human errors and communication errors. Latent errors arise due to inefficiencies in the system, such as understaffing, ineffective management, poorly maintained equipment, and inaccurate protocols. The non-conformances management requires the implementation of several actions with the objective of solving occurrences, avoiding rework, waste and loss. Structured dynamics must be able to identify and address the root cause and propose actions to minimize the risk of recurrence. The culture of identifying the root cause, implemented by the institutions, has proven to be an effective strategy for managing the quality of laboratories. Studies highlight the importance of recording non-conformances, both to show, in audits, the actions that were carried out and to track opportunities for improvement. After registration, the team will be able to use tools capable of identifying the causes of problems, such as: Ishikawa diagram, 5 whys and Pareto chart. From the identification of the root cause, it is necessary to structure an action plan to deal with occurrences with corrective and preventive actions. As evidenced in the Deming cycle (PDCA), the steps that promote continuous process improvement are based on “plan’, “do”, “check” and “act”. This tool is also widely used in the treatment of non-conformances. In this way, the search for the root cause and the elaboration of action plans are inserted in the planning phase (P) according to the PDCA. Once the root cause analysis has been well structured it will be possible to plan assertive actions so that recurrences do not occur. According to the literature, one of the most used strategies for the elaboration of action plans is the 5W2H. This tool is capable of structuring all the necessary actions to deal with a non-conformance through the following criteria: what to do, where to do it, why, when, who, how, how much. After preparing the action plan, it is necessary to implement it in the institution, this phase corresponds to “doing” (D), later, it will be essential to check (C) whether the processes are working as planned so that actions can be taken (A) adapting to routine.

**Conclusion:** Despite the diversity of non-conformities faced by institutions, it is possible to define a general basis of approaches that prove to be effective when using specific management tools. Therefore, the management of non-conformances is based on the identification, registration, classification, analysis, implementation of corrective actions, monitoring and continuous improvement.

**P-16. The pregnancy outcomes of a good morphology embryos transfer: a comparison between transfer of one euploid embryo versus two morphologically selected embryos**

**Amanda Amorim Araújo^1^, Patrícia Giffron Rodrigues^1^, Gustavo Cardoso Borges^1^, Rodopiano Souza Florêncio^1^**

*^1^ Humana Medicina Reprodutiva - Goiânia - GO - Brasil.*

**Objective:** To compare the transfer of an euploid embryo in relation to two thawed embryos not genetically tested and to correlate with gestational parameters per transfer.

**Methods:** A retrospective observational study was conducted with 383 patients recruited from a single clinical center. Data were obtained from thawed embryo transfers between January 2020 and December 2021. Two groups of thawed embryo transfers were analyzed: Biopsy Group - patients who underwent transfer of 1 euploid blastocyst of good morphological quality [≥3BB, Gardner e Schoolcraft (1999)]; Control Group - patients who underwent transfer of 2 blastocysts of good morphological quality without genetic study (≥3BB). Exclusion criteria were: oocyte recipient patients, change in the patient's or partner's karyotype previously detected that predisposes the couple to aneuploidy. The clinical data of the patients were collected, as well as the follow-up of those who had a confirmed pregnancy and we evaluated gestacional parameters such as: Implantation rate, chemical pregnancy rate (β-hCG > 40mIU/ml); Clinical pregnancy rate (confirmation of gestational sac on ultrasound); live birth rate (≥ 22weeks); chemical miscarriage rate (β-hCG >40mIU/ml without visualization of gestational sac); Clinical miscarriage rate (pregnancy loss after visualization of gestational sac); and miscarriage rate per gestational sac.

**Results:** We identified that the Biopsy group has significantly lower values than the Control Group when comparing the chemical pregnancy rate (59% vs. 70%, *p*=0.0302) and live birth rate (42% vs. 54%, *p*=0.0405). Implantation rate, clinical pregnancy, chemical and clinical miscarriage did not show statistical difference (not shown). Gestational parameters distinguished by patient age are shown in table 1. When comparing abortions due to gestational sacs in the Biopsy group and the Control group with multiple pregnancies, no differences were found when evaluating all patients. Comparing miscarriages per gestational sacs in the Biopsy group versus the Control group with multiple pregnancies, no differences were found when evaluating all patients (22% vs. 22%, *p*=1.000), in patients <35 years (9% vs. 21%, *p*=0.3683) and those aged ≥35years (28% vs. 28%, *p*=1.0000)(not shown).

**Conclusion:** We observed that, when the objective is to have a baby at home, the transfer protocol of two good quality embryos still has an advantage in our clinical center in relation to the transfer of a single euploid embryo, resulting in higher rates of chemical pregnancy and live births. Although the transfer of two embryos provides a lower implantation rate in patients aged ≥35 years, this group has lower rates of clinical abortion, providing values of live births equivalent to the Biopsy group. High rates of multiple pregnancies are highlighted, however, the rates related to abortion do not seem to interfere when we transfer 2 embryos.

**P-17. Evaluation of the use of dydrogesterone in the suppression of luteinizing gonadotropic hormone elevation during ovarian stimulation in in vitro fertilization cycles: a comparative analysis**

**Daniela Fink Hassan^1^, Karina Fattori^1^**

*^1^ Centro de Reprodução Humana Wahib Hassan - Penápolis - SP - Brasil.*

**Objective:** During the in vitro fertilization (IVF) process, controlled ovarian stimulation has been considered one of the most critical steps. Ineffective suppression of the sudden rise in gonadotropic luteinizing hormone (LH) before the trigger moment accounts for 20 to 50% of the reasons for cycle cancellation. Drug protocols for pituitary blockade during ovarian stimulation are scarce, antagonist analogs of gonadotropin-releasing hormone are the most used, reducing the impact of premature luteinization on IVF cancellation rates. The primary objective of this study was to establish the incidence of premature LH surge by determining early ovulation the pituitary blockade, with one group using oral dydrogesterone and the other using a gonadotropin-releasing hormone antagonist, resulting in cancellation of the IVF cycle. The secondary objective was to analyze the time from ovarian stimulation to the moment of triggering of oocyte maturation, correlating with age group and with ultrasonographic evaluation and counting of ovarian antral follicles.

**Methods:** This is a retrospective study carried out in a cohort of patients at the Human Reproduction Center between January 2021 and July 2023. Oocytes from 182 women underwent intracytoplasmic sperm injection and were divided into two groups. We established a descriptive comparative analysis between the group of ovarian stimulation with recombinant gonadotropin (GonalF/Pergoveris^®^) and flexible suppression with the injectable antagonist (Cetrotide 0.25 mg^®^) starting when one of the follicles reached 14 mm in average diameter, remaining until the day of onset and the other group using corifolitropin alfa (Elonva^®^)/recombinant gonadotropin (Pergoveris^®^) associated with an oral dose of 30 mg of dydrogesterone (Duphaston^®^), from the second day of the menstrual cycle until triggering.

**Results:** A comparative analysis of the results between the two protocols was determined. A total of 182 patients aged between 26 and 49 years were allocated, 89 in the antagonist group and 93 in the dydrogesterone group. In the antagonist group, the mean age was 36.9 years, with a previous antral follicle count of 5.8 follicles and a duration of ovarian stimulation of 11.31 days. In the dydrogesterone group, the mean age was 34.3 years, with 9.9 antral follicles evaluated and an ovarian stimulation period of 11.31 and 13.96 days. Premature luteinization with early ovulation was observed in 5.37% of women in the antagonist group and none of the women in the dydrogesterone group (0%). However, we observed that women who ovulated early were of advanced maternal age, with a low ovarian reserve and a low response to ovarian stimulation (Poseidon 4). We observed an extension of time in days from ovarian stimulation to the trigger moment, adding another 3.3 days in the dydrogesterone group, making a total of 12 to 14 days of ovarian stimulation

**Conclusion:** The use of dydrogesterone becomes a possible and effective option to prevent premature LH surges during the pituitary block in ovarian stimulation cycles for IVF. Associated with the benefits of reducing financial costs, ease of oral administration, and especially when the transfer of fresh embryos is not intended.

**P-18. Male infertility and the relationship of seminal viscosity and sperm DNA fragmentation**

**Darlete Lima Matos^1^, Karla Rejane Oliveira Cavalcanti^1^, Fabricio Sousa Martins^1^, Daniel Paes Diogenes de Paula^1^, Lilian Maria da Cunha Serio^1^, Ana Normelia Pereira Morais^1^**

*^1^ Nucleo de Medicina Reprodutiva do Ceara - Fortaleza - CE - Brasil.*

**Objective:** Our objective was to evaluate the existence of a relationship between sperm DNA fragmentation and seminal hyperviscosity.

**Methods:** In our study population, all patients were instructed to maintain a period of abstinence between 48 and 72 hours and perform semen collection using the masturbation method. Patients were divided into groups of samples with normal and altered viscosity parameters, correlated with the sperm DNA fragmentation index test. The samples were conditioned at 37°C for 30 minutes to determine the state of liquefaction. The semen parameters analyzed followed the guidelines protocol of the World Health Organization (WHO). Thus, the parameters of seminal volume, percentage of sperm motility, and total concentration of motile spermatozoa were evaluated. For the evaluation of sperm fragmentation, the chromatin dispersion test was used, where the spermatozoa were fixed in agarose gel and washed in lysis solutions for DNA exposure. Spermatozoa with intact DNA had an expressive halo formed around the head, while spermatozoa with fragmented DNA had no or reduced halo. For statistical analysis, results were expressed as mean ± standard error of the mean (SEM), and the two groups were compared using Student's t-test for independent samples. A significance level of 0.05 (a = 5%) was adopted.

**Results:** The results showed among patients a sperm concentration and progressive motility greater than 16x10^6^ spermatozoa/mL and 30%, respectively. Furthermore, strict Kruger morphology was greater than 4%, with spermatozoa being considered normal according to the analysis reference standard. Samples with increased viscosity (60%) did not show sperm fragmentation above normal. When analyzing the relationship between seminal viscosity and the fragmentation index, it was observed that sperm DNA fragmentation was higher in normal samples without alteration in viscosity (57%) when compared to patients with semen with increased viscosity (above 40%). According to literature data, DNA damage would be associated with low natural pregnancy rates, intrauterine inseminations (IUI), and results obtained from in vitro fertilization (IVF), in addition to the association with increased risk of miscarriages in couples who have undergone assisted reproduction techniques (for example, IVF and Intracytoplasmic Sperm Injection - ICSI). On the other hand, methods for evaluating the status of cellular DNA do not exclude the presence of a subpopulation without damage to the sperm's genetic material. Thus, in order not to justify the incorporation of fragmentation tests into the seminal analysis routine by reaffirming the low performance due to the test sensitivity.

**Conclusion:** In conclusion, this study showed the relationship between sperm viscosity and the fragmentation rate. In our results, no association was found when analyzing these parameters in isolation, which reinforces the hypothesis that this is a transient condition for the patient. In addition, seminal parameters may enable better results over time (collection of new samples), not necessarily associated with the evaluation of hyperviscosity. It is also important to correlate all standard results and additional seminal evaluation tests in an attempt to minimize the direct risks of low pregnancy rates and inappropriate medical management.

**P-19. Ex vivo mature oocyte retrieval resulting in healthy singleton pregnancy and live birth**

**Bruno Ramalho Carvalho^1^, Iris Oliveira Cabral^2^, Taise Moura Franceschi^1^, Georgia Fontes Cintra^3^, Janina Ferreira Loureiro Huguenin^3^, Leandro Santos Araújo Resende^4^, Andrea Tatiane Oliveira Silva Barros^5^**

*^1^ Bruno Ramalho Reprodução Humana - Brasília - DF - Brasil.*

*^2^ Genesis Centro de Assistência em Reprodução Humana - Brasília - DF - Brasil.*

*^3^ Instituto Brasileiro de Controle de Câncer - São Paulo - SP - Brasil.*

*^4^ Oncoclínicas Aliança Asa Sul - Brasília - DF - Brasil.*

*^5^Centro de Tratamento do Câncer Medradius - Maceió - AL - Brasil.*

**Objective:** To report the first pregnancy (surrogate) and live birth from the intracytoplasmic sperm injection (ICSI) of ex vivo retrieved mature oocytes, in a woman with bilateral ovarian carcinoma.

**Methods:** Case report

**Results:** A 34-year-old nulliparous woman with bilateral ovarian tumor, with a risk of malignancy at 96.1%, according to IOTA-ADNEX. Controlled ovarian stimulation was performed with corifolitropin alfa 150mcg, complemented by follitropin beta 100IU/day from day 5 to day 7, and followed by follitropin beta 200IU/day, the latter from the day 8 onwards, in a GnRH antagonist cycle. A single dose of choriogonadotropin alfa 250 mcg was used as trigger on stimulation day 12. Surgery was programmed to occur ~36 hours after the trigger. Follicular aspiration was proceeded in the operating room, guided by ultrasound applied directly to oophorectomized specimens, yielding 12 metaphase II oocytes, which were vitrified. After remission of the disease, the patient returned, counting on her 33-year-old sister for surrogacy. ICSI with donor sperm was performed in the 12 vitrified-thawed oocytes, resulting in the normal development of 9 cleavage-stage embryos, two of which were chosen for transfer to the surrogate mother, according to KIDScore D3. Surplus embryos were cryopreserved. A positive pregnancy test was achieved 12 days after embryo transfer, evolving to a normal healthy singleton pregnancy, an uneventful prenatal follow-up, and the live birth of a healthy 46,XX baby.

**Conclusion:** Ex vivo retrieval and cryopreservation of mature oocytes is a viable and efficacious approach for fertility preservation in women with ovarian cancer, mitigating the risk of tumor cells spillage into the pelvis. This is the first report of pregnancy and live birth resulting from ICSI of ex vivo retrieved mature oocytes.

**P-20. Overlapping doses of corifollitropin alfa in a progestin-primed regimen may be an interesting advance towards a patient-extremely friendly controlled ovarian stimulation: a case report**

**Juliana Késia Araújo Fonseca^1^, Bárbara Veloso Avila Chaves^1^, Maria Júlia Ferreira de Carvalho Mariano Rodrigues Cunha^1^, Nayara Santos Soares^2^, Estella Thaisa Sontag Reis^2^, Bruno Ramalho Carvalho^2^**

*^1^ Centro Universitário de Brasília - Brasília - DF - Brasil.*

*^2^ Bruno Ramalho Reprodução Humana - Brasília - DF - Brasil.*

**Objective:** To report the results of controlled ovarian stimulation using ovelapped doses of corifollitropin alfa, in a progestin-primed cycle, for social egg freezing.

**Methods:** Case report

**Results:** Nuligesta, 37 years old, with regular menstrual cycles, 57kg, body mass index 20.19 k/m2, sought the private clinic for social egg freezing. No alterations were identified in pre-treatment complementary exams and the ultrasonographic antral follicle count was 17. The patient was stimulated following a progestin-primed ovarian stimulation protocol, using corifolitropin alfa (CFA), 100 mcg, on the third day (D3) of the menstrual cycle. A new 100 mcg dose of CFA was administered on treatment day 5 (D5), according to the observation of pharmacokinetic curves described in CFA validation studies. Pituitary supression was also initiated on D5, using desogestrel (DSG), 75 mcg/day. On D11, follicular maturation was triggered with triptorelin acetate, 0.2 mg (two injections of 0.1 mg).Abdominal distention and edema of the lower extremities were the only adverse effects reported, but they were well tolerated without specific medication. Follicular aspiration was performed 36 hours after trigger, yielding 12 oocytes, of which 10 in metaphase II (vitrified), 1 in metaphase 1 and 1 in prophase I (discarded).

**Conclusion:** Overlapped doses of CFA in a DSG-primed ovarian stimulation protocol demonstrated to be a safe, efficacious, well-tolerated, and cost-effective, being an extremely friendly treatment option for social egg freezing. Further well-designed studies are necessary for validation.

**P-21. Assisted Reproduction Techniques for same affective couples: A literature review**

**Fernanda Kunrath Robin^1^, Bruna Andrade da Silva^2^, Gissele Nardini Artigas de Oliveira^2^, Nilo Frantz^1^**

*^1^ Nilo Frantz Medicina Reprodutiva - Porto Alegre - RS - Brasil.*

*^2^ Centro Universitário Ritter dos Reis - Porto Alegre - RS - Brasil.*

**Objective:** The main objective of this research was to identify the main assisted reproduction techniques that can be used by same-sex couples.

**Methods:** This is a narrative review, based on scientific productions published between 2015 and 2021. The search was carried out in the Virtual Health Library (VHL) database. The descriptors “reproduction techniques” and “homosexuality” were consulted on the Health Sciences Descriptors website (DECS) and applied in the VHL, generating the respective search code: reproduction techniques AND homosexuality AND (year_cluster:[2015 TO 2021] As inclusion criteria for the research, full and original texts with publications in the years 2015 to 2021, in the languages: English, Portuguese, French, Spanish and German that fit the subject studied were chosen. publications that did not answer the question to be discussed in this study were defined.

**Results:** The search resulted in 40 publications that were carefully organized and read, remaining in the study 08 articles that answered the guiding question of the research. The studies presented demonstrated that there are several treatment possibilities for same-sex couples to fulfill their desire to build their families through the use of their own gametes and a solidary uterus. Among the possibilities are intrauterine insemination and in vitro fertilization treatments. In Brazil, it is possible to use heterologous gametes, and we have some national and international semen banks, where it is possible to choose the most appropriate characteristics and purchase the material. In the case of eggs, we rely on international banks or on the RHA clinics' own egg banks. Furthermore, there is the possibility for homosexual couples to carry out in vitro fertilization treatments, donating part of their gametes and receiving discounts on their treatments. In addition to the possibility of using a replacement uterus, mainly necessary for male homoaffective couples.

**Conclusion:** This study showed that in Brazil there are several possibilities for same-sex couples to build their families through their own gametes, however, the complexity of treatments combined with high costs are still factors that make access difficult for the population. In addition, even though same-sex unions are recognized in Brazil, there are still many taboos on this subject and many have the vision and defend the concept of traditional family, that is, the one composed of father and mother, of different genders. opposites.

**P-22. The Power of Interdisciplinary Collaboration: Integrating Mental Health Professionals in Multidisciplinary Care of Transgender Individuals**

**Helena Prado Lopes^1^, Flávia Assis Silva Giacon^2^**

*^1^ Pró-Fértil Medicina Reprodutiva - Rio de Janeiro - RJ - Brasil.*

*^2^ Mater Prime Clínica de Reprodução Humana - São Paulo - SP - Brasil.*

**Objective:** This study aimed to analyze narratives of transgender patients received at the primary care clinic for transgender people by the multidisciplinary team of the State Institute of Diabetes and Endocrinology (IEDE) in Rio de Janeiro. Our interest in undertaking this study stemmed from observing narratives of transgender patients, which provided us with a broader understanding of the intricate issues they face, particularly pertaining to the reconfiguration of their roles within society, as well as the profound consequences encountered within familial and social contexts. As researchers, we participated as listeners in team meetings comprising a psychologist, endocrinologist, nurse, social worker, and psychiatrist, engaged in patient screenings at the clinic, and, based on that, noticed that most patients shared past experiences being subjected to discriminatory behavior by healthcare teams in other care centers. In this context, based on the analysis of the psychological interviews conducted during patient screening, we observed that the vast majority of individuals reported experiencing anxiety, insecurity, feelings of worthlessness, family/social isolation, and distress arising from the fear of not being able to access medical treatment for gender affirmation. It is important to highlight that patients’ negative perceptions towards medical care, regardless of the intentions of the healthcare professionals, may lead to feelings of discomfort and mistrust, ultimately discouraging transgender individuals from seeking specialized medical assistance, for example. Numerous patients reported perceiving insensitivity towards their gender identity from the medical staff at care centers, including the use of incorrect pronouns (for example, referring to a transgender woman as “he”). Nevertheless, our experience as listeners during patient screenings and team meetings highlighted the dedicated and respectful approach towards the patients demonstrated by the IEDE team. Our findings suggest that the thoughtful and considerate approach adopted by the multidisciplinary team at IEDE, which incorporates mental health professionals, empowers patients to shape their coping strategies and pursue more adaptive means to mitigate both potential challenges and anxiety experienced during treatment. Furthermore, we highlight the importance of fostering collaborative efforts between healthcare professionals and mental health practitioners within assisted reproduction centers. Finally, it is imperative to recognize and address inherent anxieties associated with medical care while also ensuring the availability of collaborative psychosocial support networks. Such initiatives are vital in enhancing the overall quality of life for transgender individuals.

**Methods:** The methodology used in this study encompassed literature review and data collection from research articles pertaining to the topic. A systematic review of national and international literature was carried out between March and December, 2022 on databases such as the Scientific Electronic Library Online (SciELO), Google Scholar, and PubMed. After the application of inclusion and exclusion criteria, 12 articles were identified. Most studies were international, qualitative in nature, with a specific emphasis on evaluating the quality of care delivered to transgender individuals by multidisciplinary teams.

**Results:** The literature review consistently revealed that transgender individuals often perceive inadequate support from healthcare professionals, which has also been identified based on the patient reports from the IEDE healthcare clinic.

**Conclusion:** We emphasize the importance of establishing collaborative efforts between healthcare professionals and mental health practitioners within assisted reproduction centers, as done at the IEDE. This integration is crucial for effectively addressing the unique needs of the transgender population. The extensive healthcare experience provided to transgender individuals at the aforementioned clinic highlights the vital importance and need for effective interdisciplinary teamwork in facilitating and delivering comprehensive care for these patients.

**P-23. Correlation between the time of disappearance of pronuclei and blastulation rate**

**Andrea Mesquita Lima^1^, Sebastião Evangelista Torquato Filho^2^, Tulius Augustus Ferreira Freitas^1^, Gleicyane Sousa Santos Alam^1^, Eduardo Gomes Sá^1^, Ellayne Cavalcanti Queiroz^1^**

*^1^BIOS - Fortaleza - CE - Brasil.*

*^2^Sollirium - Fortaleza - CE - Brasil.*

**Objective:** The objective of this study was to correlate the blastulation rate of embryos cultured in a Timelapse incubator, with the time in which the pronuclei disappeared.

**Methods:** After verification of fertilization, patients whose zygotes had two pronuclei were grouped according to the time of disappearance of the pronuclei. 303 zygotes whose pronuclei disappeared before 22 hours after intracytoplasmic sperm injection (ICSI) and 170 zygotes whose pronuclei disappeared after 22 hours of ICSI.

**Results:** It was observed that the embryos with the best morphokinetic Evolution and higher rate of blatulation were those in which the pronuclei disappeared less than 22 hours after ICSI (78,2%) when compared to zygotes whose pronuclei disappeared after 22 hours after ICSI (48%).

**Conclusion:** The time-lapse technology is very useful, as it allows the monitoring of events that would not be possible to be evaluated in conventional cultivation. In the present study, it was evidenced that the time of disappearance of the pronuclei seems to be a predictive value for blastulation rate.

**P-24. Definition of a protocol of seminal cryopreservation for Rio de Janeiros’s Sperm Bank**

**Lincoln Bastos Farias Junior^1^, Mariana Duque Mello^1^, Marcel Frajblat^2^, Paula Fontoura^1^**

*^1^ Banco de Sêmen do Rio de Janeiro (BSRJ) - Rio de Janeiro - RJ - Brasil.*

*^2^ Universidade Federal do Rio de Janeiro (UFRJ) - Rio de Janeiro - RJ - Brasil.*

**Objective:** The aim of this study is to compare the crioprotetors Spermfreeze™, Ingá Spermfreezing™ and Spermfreeze SSP™ and define a standard operating procedure (SOP) for sperm freezing at Rio de Janeiro’s Sperm Bank

**Methods:** Samples of patients of Rio de Janeiro Sperm Bank were used. The patients signed a Free and Informed Consent Form, which informs the possibility of using their data in scientific studies. Patients with azoospermia and severe oligospermia were excluded from the study. Before the freezing, the parameters motility, morphology and vitality of the samples were analyzed. The freezing was carried out according to the protocol of each kit and the samples were frozen for a period of one month. After one month, they went through the thawing process to analyze the rates of motility, vitality and sperm morphology.

**Results:** After the analysis before and after freezing the sample of 13 volunteers, the mean of recovery of sperm motility was 59.211±5.027 using Spermfreeze™, 61.15±4.562 using Ingá Spermfreezing and 61.86±6.134 using Spermfreeze SSP™; the mean of recovery of sperm morphology was 91.59±3.299 using Spermfreeze, 86.01±5.536 using Ingá Spermfreezing and 91.20±4.452 using Spermfreeze SSP™, the mean of recovery of sperm vitality was 60.06 ± 4.940 using Spermfreeze, 58.88±6.512 using Ingá Spermfreezing and 56.16±7.275 using Spermfreeze SSP™. No significant difference was found between cryoprotectants in the parameters of survival, motility and sperm morphology.

**Conclusion:** Aiming the reduction of the use of components of animal origin in the laboratory at Rio de Janeiro’s Sperm Bank and considering the cost-effectiveness of each cryopreservation medium, Fertipro^®^ Spermfreeze™ medium was chosen as the SOP of choice for seminal cryopreservation at Rio de Janeiro’s Sperm Bank.

**P-25. TikToking infertility in Brazil**

**Renata Lima Bossi^1^, Raquel Lanna Cerqueira^1^, Debora Alvarenga^1^, Ana Carolina Xavier^1^, Marcos Sampaio^1^, Marcia Mendonça Carneiro^1^**

*^1^ Origen Medicina Reprodutiva - Belo Horizonte - MG - Brasil.*

**Objective:** To determine the prevalence, authorship, and types of fertility-related information shared on TikTok (TT) in Brazil using hashtag and content analysis. Analysis of post content accordingto author type (healthcare professional versus lay people) and the identification of the topics that prevailed in those interactions were secondary outcomes.

**Methods:** We hypothesized that there is a high volume of fertility/infertility health related

content on TikTok (TT) in Brazil and that the accuracy of this content is generally poor. As such, we sought to characterize the presence of fertility/infertility -related information on TT in two ways.

1.quantify the volume of fertility/infertility related content on TT using the following hashtags: maternity, TTC (trying to conceive), IVF, endometrisis and infertility.

2.Classify the health-related content contained on these posts. Post content classification was determined by a single author . In instances where post characterization or accuracy were equivocal, a third author reviewed the post for adjudication.

Each post was characterized by type into:

1.educational/ scientific

2.promotional,

3.testimonial

4.entertainment.

The account from which the post was created was further characterized by account type: (personal, creator, business/clinic/industry, healthcare professional (HCP) and, physician. We also determined the number of followers, likes, comments, and shares for each post on TT.

**Results:** Overall we found 46 accounts that published on the topics searched: #maternity (n=10), #TTC (n=10), #endometriosis (n=10),#IVF (n=10) and infertility (n=10). Content analysis showed that the majority focused on personal testimonials (n=25), educational/scientific (n=20) and entertainment (n=5). As for authroship, most were lay individuals (n=23), followed by those reporting on their personal experience (n=11), medical doctors (n=10), healthcare professionals (n=6) and one clinic. The number of followers ranged from 89 to 4.4 milion with a mean of 3012. The account with most followers (n=4.400.000) belongs to a medical school student talking about infertility , followed by a creator account (n=1.800.000 ) on endometriosis, and third one by another medical school student (n=1.500.000 ) focused on infertility. Engagement measured by the number of likes and comments revealed that the 3 most popular topics were: (1)TTC :entertainment vídeo by a creator (1.7 milion likes, 24.700 comments, 21.900 saved, 14.900 shares; (2)maternity: entertainment vídeo by a creator (1.6 milion likes, 12.700 comments, 63.900 saved, 14.400 shares,; (3)infertility a scientific vídeo by a creator (637.900 likes, 24.300 comments, 16.500 saved, 23.900 shares, type educational.

**Conclusion:** The majority of infertility related TikTok posts are authored by lay people and discuss infertility and endometriosis with millions of followers. Entertainment vídeos on TTC and maternity engaged the most attention followed by a scientifc vídeo. Despite the availability of various scientific vídeos, those with personal content engaged more attention. Official healthcare presence was lacking and healthcare professionals are still a minority. TikToki s an extremely popular social media especially among young people and may represent a great opportunity to educate on relevant themes such as infertility and endometriosis. Increased healthcare professional participation is advisable. More studies examining the volume, reach and accuracy of reproductive health information available on social media such as TikTok are necessary.

**P-26. Surrogacy: a 20-year experience in a Reproductive Medicine clinic in Brazil**

**Cássia Cançado Avelar^1^, Natalia Silva^1^, Leticia Eto^1^, Catharina Cançado Avelar^2^, Ricardo Mello Marinho^1^, Erica Becker Sousa Xavier^1^, Joao Pedro Junqueira Caetano^1^**

*^1^ Clínica Huntington Pró-Criar - Belo Horizonte - MG - Brasil.*

*^2^ Faculdade de Medicina UNIFENAS - Belo Horizonte - MG - Brasil.*

**Objective:** To demonstrate through numbers the main indications of surrogacy, multidisciplinary approach and the outcomes in the treatments of surrogacy.

**Methods:** Retrospective analysis of the psychological follow-up of patients who sought an in vitro fertilization treatment with surrogacy, highlighting aspects related to medical indication, psychological assessment and ethics discussion.

**Results:** A total of 88 patients had the indication of surrogate between April 2003 and April 2023 in a private reproductive medicine clinic in Belo Horizonte/Brazil. The average age of people who underwent treatment was 36 years. The main medical indications for surrogacy were following hysterectomized women for benign diseases in 24 cases (27.3%); serious medical conditions in 19 (21.6%); 16 (18.2%) homosexual patients; 2 (2.3%) single men; congenital absence of uterus in 11 cases (12.5%); cancer surgery in 8 patients (9%); recurrent miscarriage in 6 cases (6.8%) and repeated IVF failures in 2 cases (2.3%). 37 (42%) couples/patients underwent treatment, we had incomplete data from 9 (10,2%) patients, 4 patients (4.5%) underwent oocyte cryopreservation for future treatment and another 38 (43.3%) did not undergo treatments after the indication. Of the 37 (42%) couples/patients underwent treatment, 3 (8.1%) are pregnancy and 14 (37.8%) had a live birth;13 (35.2%) were negative results; 5 (13.5%) progressed to abortion and 2 (5.4%) had a canceled cycle. Of the 38 (43.3%) patients/couple did not undergo treatments after the indication, the main reasons were: 21 (55.3%) had a first medical and psychological evaluation, but chose not to undergo the treatment; 10 (26.3%) was considered unfit by the doctor; 4 (10.6%) were considered psychologically unfit; 1 (2.6%) gave up the treatment, due to the couple broke-up; 1 (2.6%) would have to use donated eggs with surrogacy and this was not authorized in CFM Resolution number 2.013/2013 and 1 (2.6%) was not authorized by the CRM/MG, as the patient was a single man and there was no inclusion of single people in chapter VII of Substitute gestation at CFM Resolution no. 2.121/2015. In these 20 years, we have offered systematic psychological listening - whether individual, couple or in groups - that allows us pay attention to the unique dimension of the experience of people who experience surrogacy treatment. Thus, we can evaluate and follow up the patient/genetic couple, as well as the surrogate and their family, to identify the affective bonds and possible psychological factors that may predispose to treatment, in addition to optimize the conditions of the family environment that will receive the unborn child. In Brazil, the CFM, in its resolution no. 2.320/2022, determines that the patient's medical record must contain a medical report, with a psychological profile, attesting the clinical and emotional adequacy of all those involved, and this has been the practice at our clinic since the first case of surrogacy in 2003.

**Conclusion:** The surrogacy is an alternative treatment that gives patients, unable to conceive, a chance to get a biological child, on the condition that everyone involved in the process must be well evaluated from a medical, psychological and ethical point of view.

**P-27. Female throuple seeking Assisted Reproduction Treatment: Report of desires and reproductive possibilities**

**Cassia Cançado Avelar^1^, Leci Veiga Caetano Amorim^1^, Cristiane Araujo Oliveira^1^, Ana Luisa Mendes Campos^1^, Laura Maria Almeida Maia^1^, Ricardo Mello Marinho^1^, Erica Becker Sousa Xavier^1^, Joao Pedro Junqueira Caetano^1^**

*^1^Clinica Huntington Pró-Criar - Belo Horizonte - MG - Brasil).*

**Objective:** To evaluate desires and possibilities of an assisted reproduction treatment in a female throuple relationship, highlighting aspects related to medical indication, psychological assessment and ethics discussion.

**Methods:** Follow up Female throuple (3 women), who looked for treatment in a private reproductive medicine clinic in Belo Horizonte/Brazil, in January 2023.

**Results:** Case report: Patient 1 (39 years) and patient 2 (38 years) - stable union for 10 years and intended to have children. Patient 3 (34 years old), had heterosexual; transgender and homoaffective relationships. In 2019, patient 3 met patient 1. Later, patient 3 met patient 2 - they had a great identification and began a throuple relationship. They sought the clinic for treatment. After a favorable medical evaluation, the throuple had a psychological evaluation, with individual and group consultation, where they could talk about their desires and treatment possibilities. The case was presented to the Ethics Committee of the clinic (composed by physicians, psychology, nurse and embryologist). Considering the determination of Federal Council of Medicine (CFM) Resolution No. 2.320/2022, that all capable person who have requested the procedure and whose indication does not deviate from the limits of this resolution, may be recipients of assisted reproduction techniques; and in the event of embryos formed from different donors, embryo transfer should be performed with embryos of a single origin for the safety of offspring and traceability, the Ethics Committee determined that there was no contraindication for the treatment involving the throuple. In the psychological sessions, they expressed their desires and expectations regarding the treatment and with the medical advice, they decided by an in vitro fertilization with heterologous semen, using the oocyte of the three, but patient 2 does not want pregnancy.

**Conclusion:** New family arrangements represent a challenge for assisted reproduction treatments. It is necessary a good listening by the multidisciplinary team and adequate guidance on the available treatments, respecting the norms in the Resolution of the Federal Council of Medicine (CFM).

**P-28. Comparative report of psychological care of two cases of transgender men undergoing assisted reproductive techniques**

**Cassia Cançado Avelar^1^, Leci Veiga Caetano Amorim^1^, Laura Maria Almeida Maia^1^, Ricardo Mello Marinho^1^, Erica Becker Sousa Xavier^1^, Joao Pedro Junqueira Caetano^1^**

*^1^ Clínica Huntington Pró-Criar - Belo Horizonte - MG - Brasil.*

**Objective:** To evaluate the history and experiences of two cases of transgender men in an assisted reproduction treatment.

**Methods:** Psychological follow up two couples, with transgender men married with cisgender women, who sought treatment at a private reproductive medicine clinic in Belo Horizonte/Brazil, in January 2021.

**Results: Cases report:First case:** Transgender man (34 years) in a relationship with a cisgender woman (36years). She looked for a treatment in 2020, when single, and she was diagnosed with low ovarian reserve. She reported her anguish at this diagnosis, so she did not do the treatment. After that, she met an old friend (a transgender man) and they had been together ever since. He started the reassignment process seven years ago and he has been using testosterone since then. He underwent a mastectomy and was preparing to have his uterus and ovaries removed, when the Covid pandemic came and he had to postpone the surgical procedure. He spoke about his desire to have children, but that it was not in their plans to carry out the treatment, which changed given the situation of his partner. In 2021, they did a treatment with ovulatory induction on him. He said that the treatment was intense, as he had to stop testosterone and use female hormones, which brought intense symptoms. He was very irritated; in addition, it greatly affected his physical strength, which reflected in his work. He had two blastocysts and they had two transfers into his wife’s uterus, with an anembryonic pregnancy and another a negative result. They decided not to continue the treatment, as he revealed was not willing to undergo hormone treatment again. **Second case:** Transgender man (37 years) married a cisgender woman (43 years) with altered karyotype and low ovarian reserve. She did treatment when single with donor semen with anembryonic pregnancy and negative result. She returned in 2021, in a relationship with a transgender man. He transitioned eight years ago, but ever had a strong desire to be a father. He did intend to remove the uterus, tubes and ovaries, but first he was willing to perform the ovulation induction treatment. He did need double stimulation and said that the use of the hormone was less impactful than he had imagined and his desire to have a child was bigger than that. He had an embryo transferred to his wife with a positive result. The couple's daughter was born in 2022. The question in this case was she has not told anyone that he is a transgender man and they are in therapy.

**Conclusion:** The psychological approach in these cases was before starting treatment, with follow-up during the treatment and all the implications of the technical demand on the transgender patient's body and emotion and follow-up after the result - grieving the negative outcome and decisions about the way forward and the experience of pregnancy and childbirth. This made it possible to provide the necessary support in view of the psycho-emotional demands of each couple.

**P-29. Effect of body mass index on blastocyst morphokinetics in polycystic ovary syndrome (PCOS): a time-lapse analysis of embryos obtained after progestin primed ovarian stimulation cycles**

**Luciana Campomizzi Calazans^1^, Camila Cruz Moraes^1^, Leci Caetano Amorim^1^, Ana Luisa Menezes Campos^1^, Erica Becker Souza Xavier^1^, Ricardo Melo Marinho^1^, João Pedro Junqueira Caetano^1^**

*^1^ Huntington Pró-Criar - Belo Horizonte - MG - Brasil.*

**Objective:** To demonstrate if there are differences in blastocyst morphokinetics between normal weight and overweight/obese women with PCOS after controlled ovary hyperstimulation with progestin primed ovarian stimulation (PPOS) protocol.

**Methods:** PCOS is a common endocrine disorder associated with overweight and obesity in women of reproductive age. An increasing number of PCOS women are seeking for assisted reproduction techniques (ART) and the impact of overweight and obesity on the reproductive outcome of PCOS women who undergo IVF/ICSI have yielded conflicting results. PCOS obese women require higher doses of gonadotropin for a longer duration to achieve ovarian hyperstimulation, and overweight and obesity are associated with higher miscarriage rate in IVF/ICSI patients. However, the effect of overweight and obesity in PCOS on the morphokinetics parameters of blastocysts is yet to be determined. This was a retrospective cohort study on blastocyst morphokinetics from February/2021 and January/2022, in a single ART center. One-hundred twenty-three blastocysts from 24 PCOS patients submitted to PPOS protocol were included and classified into three groups: normal weight (n=86), overweight (n=20) and obese (n=17), according to the World Health Organization (WHO) classification. There were no underweight women in this cohort. Embryos from patients with endometriosis, previous ovarian surgery diabetes, autoimmune diseases and male factor were excluded from this analysis. Embryos were cultured in Embryoscope Plus incubator and classified according to KIDScore (Vitrolife) morphokinetic-based algorithm in good (7.0-9.9), fair (4.0-6.9) and poor quality (1.0-3.9). We performed two-by-two comparisons between the average scores obtained from the quality of the embryos fornormal x overweight; normal x obese; overweight x obese and normal x overweight + obese groups.

**Results:** The normal and obese groups had a mean age statistically higher than the overweight group (36.7±4.0; 38.5±2.7 and 33.2±2.8 respectively, *p*<0.0001). The number of oocytes retrieved was significantly higher in the normal weight and overweight groups (20.0±7.3 and 22.3±8.1), when compared to the obese group (10.6±5.0, *p*<0.0001). As for the blastocyst formation rate, the normal and overweight groups had rates statistically higher than those in the obese group (0.6±0.2; 0.6±0.1 and 0.5±0.2, *p*=0.0114). The embryos generated by patients in the normal BMI group had a mean score of 6.3(±1.8); the overweight group had a mean score of 6.1(±1.9) and the obese group had a mean score of 7.0(±1.71). There was no significant difference in relation to the average quality score obtained between the three groups analyzed (*p*>0.05), according to the Tukey Test for multiple comparisons. In the analyzes two by two (normal x overweight; normal x obese; overweight x obese and normal x overweight + obese groups), there was also no statistical significance (*p*>0.05), according to the unpaired T test. Limitations, reasons for caution:

We found no relation between BMI and embryo morphokinetics scores, despite the obese group having higher mean age.

**Conclusion:** Many studies have shown a detrimental effect of obesity on IVF/ICSI and obstetrics outcomes in PCOS women, but a few have focused on the embryo quality. Our study found no relation between BMI and embryo morphokinetics scores in PCOS women who underwent PPOS IVF/ICSI cycles.

**P-30. Increased Anti-Mullerian Hormone secretion per antral follicle is linked to decreased ovarian response after controlled stimulation for IVF**

**João Sabino Cunha-Filho^1^, Rita Chapon^1^, Vanessa Genro^1^, Rafaela Donato^1^, Camila Bessow^1^, Tatiane Souza^1^**

*^1^ Insemine - POA - RS - Brasil.*

**Objective:** The main objective of this study is to investigate if the anti-Mullerian Hormone secretion per antral follicle is linked to ovarian response after controlled stimulation in infertile patients undergoing IVF.

**Methods:** We did a retrospective study with 483 patients submitted to IVF with 24 - 45 years of age, during 2015-23. All of the studied patients presented both ovaries, with no morphological abnormalities (such as cysts, endometriomas, etc.), no history of past surgery and adequately visualised in transvaginal ultrasound scans; regular menstrual cycle lengths ranging between 25 and 35 days; no current or past diseases affecting ovaries or gonadotrophin or sex steroid secretion, clearance or excretion; no clinical signs of hyperandrogenism and BMI ranging from 16 to 30 kg/m2. The controlled ovarian stimulation protocol was initiated with recombinant FSH therapy, the individual dosage was according to the physician’s option and continued until the day of hCG administration. All included patients received GnRH antagonist (flexible protocol) and daily FSH doses were adjusted according to the number of growing follicles. Patients were divided into two groups according to the AMH/AFC ratio. Group 1 (n=359) 0.39 and group 2 (n=124) when this ratio reaches the 75th percentile (>0.39). The primary outcome was determined by the number of mature (MII) oocytes after pickup. p was considered significant when < 5%.

**Results:** Groups were comparable in age (mean±SD) 35.6 ± 4 for group 1 and 35.1±5 for group 2, *p*=0.293. However, AMH (2.1±1.8 versus 7.6±2.8) and AFC (11±6 versus 9±5) were significantly different between both groups (*p*<0.05). Moreover, the group with a higher AMH secretion per AFC presented a lower number of collected oocytes (5±3 versus 4±2, *p*=0.003) and a decreased number of mature oocytes (4±3 versus 3±2) compared to the group with AMH per AFC less than 0.4, *p*<0.05. Considering the AMH secretion per AFC (higher than 0.39) and the individual chance to present, after controlled ovarian stimulation, less than 5 mature oocytes, the odds were 2.10, 95%CI(1.30-3.45) in this group of patients.

**Conclusion:** Anti-Mullerian Hormone is an essential tool for the ovarian response after gonadotropin administration. This hormone is largely used to predict and decrease the incidence of ovarian hyperstimulation syndrome. However, this anti-gonadotrophic action could be detrimental when this secretion (per AFC) is above the 75th percentile cut-off.

**P-31. Endometriosis does not affect Antral follicle responsiveness to follicle stimulating hormone administration in patients undergoing to IVF**

**João Sabino Cunha Filho^1^, Rita Chapon^1^, Rafaela Donato^1^, Tatiane Souza^1^, Camila Bessow^1^, Vanessa Genro^1^**

*^1^ Insemine - POA - RS - Brasil.*

**Objective:** The main objective of this research is to investigate if the presence of endometriosis in patients undergoing IVF affects the Antral Follicle responsiveness to exogenous gonadotropins for controlled ovarian stimulation.

**Methods:** We retrospectively studied 806 patients submitted to IVF with 24 - 45 years of age, during 2015-22. All of the studied patients met the following inclusion criteria: (i) both ovaries present, with no morphological abnormalities (such as cysts, endometriomas, etc.), no history of past surgery and adequately visualised in transvaginal ultrasound scans; (ii) regular menstrual cycle lengths ranging between 25 and 35 days; (iii) no current or past diseases affecting ovaries or gonadotrophin or sex steroid secretion, clearance or excretion; (iv) no clinical signs of hyperandrogenism; (v) BMI ranging from 16 to 30 kg/m2. Recombinant FSH therapy was then initiated, at an individual dosage according to the physician’s option and continued until the day of hCG administration. From the 6th day of recombinant FSH therapy onwards, daily FSH doses were adjusted according to the number of growing follicles and GnRH antagonist was administered. The main outcome was determined by the Follicular Output RaTe (FORT) that was calculated by the ratio between Peri-ovulatory Follicular Count on day-hCG × 100/Antral Follicular Count at baseline. We compared endometriosis x non-endometriosis patients in terms of baseline and some reproductive outcomes. p was considered significant when < 5%.

**Results:** The mean age of the patients was (mean±SD) 35.1±4 for endometriosis and 35.3±5 for the control group, *p*=0.722. Moreover, AMH (3.88±3.4 versus 3.45±3.3), AFC (10±5 versus 11±6), and the number of mature oocytes (3.6±2 versus 3.9±3) were similar between both groups (*p*>0.05). Furthermore, the responsiveness to gonadotropin measured by FORT was also similar between endometriosis and non-endometriosis: 43% x 42.9%, *p*=0.957.

**Conclusion:** Endometriosis did not affect the follicular responsiveness to follicle-stimulating hormone during controlled ovarian stimulation for IVF. Moreover, some reproductive outcomes after COS were also similar between both groups.

**P-32. Endometriosis is associated to an increased serum progesterone level during late follicular phase in controlled ovarian stimulation for in vitro fertilization**

**Tatiane Oliveira de Souza^1^, Rita Chapon^1^, Rafaela Donatto^1^, Camila Bessow^1^, Vanessa Krebs Genro^1^, João Sabino Cunha Filho^1^**

*^1^ Centro Reprodução Humana Insemine - Porto Alegre - RS - Brasil.*

**Objective:** To investigate the serum progesterone, LH and estradiol levels during late follicular phase in infertile patients with endometriosis submitted to controlled ovarian stimulation for IVF.

**Methods:** We prospectively studied 102 infertile patients undergoing the first IVF cycle using the same controlled ovarian stimulation protocol (hMG + GnRH antagonist), during 2021-22. All of the studied patients met the following inclusion criteria: (i) both ovaries present, with no morphological abnormalities (such as cysts, endometriomas, etc.), no history of past surgery and adequately visualised in transvaginal ultrasound scans; (ii) regular menstrual cycle lengths ranging between 25 and 35 days; (iii) no current or past diseases affecting ovaries or gonadotrophin or sex steroid secretion, clearance or excretion; (iv) no clinical signs of hyperandrogenism; (v) BMI ranging from 16 to 30 kg/m2. Patients were divided into two groups. Study group: peritoneal endometriosis diagnosticated using laparoscopy, n=27 and control group: tubal or masculine infertility etiology, n=72.Progesterone, estradiol and LH were collected on hcg-day and analyzed using chemiluminescence immunoassay. We also compared some important reproductive outcomes. Our data were analyzed using parametric and non-parametric correlation tests and multivariable analysis (Linear regression). P was considered significant when < 5%.

**Results:** Mean age (35.4±4 x 35.7±4), Antral Follicle Count (10±5 x 8.5±5), AMH (2.33±1.9 x 2.33±2.3), number of MII oocytes (6±4 x 6±5) and number of embryos (5±1 x 5±1) were not different between control and endometriosis groups, respectively (*P*>0.05). Furthermore, serum estradiol (2743±222 x 3046±278) and LH (2.54±3 x 2.53±4) levels did not differ also. However, serum progesterone levels on the HCG-day were increased in endometriosis patients compared to controls (1.12±0.6 x 0.87±0.5, *P*=0.026). Using the cutoff of serum progesterone above 1.5 ng/ml to freeze embryos and transfer in another cycle, 50% of endometriosis patients reached that criterion versus 33% of the control group (*P*=0.09).

**Conclusion:** Patients with endometriosis demonstrated a granulosa cell secretion of progesterone after controlled ovarian stimulation for IVF different if compared to tubal or male fector. Those endometriotic patients are at risk of premature progesterone increase during the late follicular phase and need to be closely monitored.

**P-33. Incubation time to ICSI does not affect laboratory results**

**Bruna Campos Galgaro^1^, Luiza da Silva Rodrigues^1^, Lisiane Knob de Souza^1^, João Sabino Lahorgue Cunha-Filho^1^**

*^1^ Insemine - Porto Alegre - RS - Brasil.*

**Objective:** To evaluate whether incubation time from oocyte collection to ICSI fertilization affects laboratory results.

**Methods:** A retrospective study which included all fresh ICSI cycles from January to June 2023. The fertilization procedure was performed between three to six hours after ovarian aspiration (i.e. 39 to 42 hours after hCG trigger). Cycles were divided in two groups according to the incubation period until ICSI: group 1 [≤ 4 hours (or 180-240 minutes)] and group 2 [>4 hours (or 241-360 minutes)]. Age of patients, AMH (ng/mL) and the number of oocytes retrieved were assessed. The primary laboratory outcome compared was good quality blastocyst rate, and the secondary were: fertilization rate, good quality cleavage embryo rate, and blastulation rate. Statistical analysis was conducted by paired test and results, expressed as mean ± standard deviation, were considered significant if *p*<0.05.

**Results:** There were 96 ICSI cycles included, 77 of which were performed up to 4 hours and 19 after 4 hours of oocyte collection. The average time to ICSI was 215 (minimum 180 and maximum 348 minutes). The mean age of patients [36.13 ± 4.48 vs 35.94 ± 4.80 (*p*=0.876)], AMH [2.72 ± 2.71 vs 2.47 ± 1.97 (*p*=0.707)] and number of oocytes retrieved [8.82 ± 6.33 vs 10.58 ± 13.06 (*p*=0.396)] were not significantly different for groups 1 and 2 respectively. Moreover, no statistical difference were observed in relation to all laboratory results evaluated between groups: good quality blastocyst rate [40.0% ± 0.33 vs 31.4% ± 0.36 (*p*=0.439)], fertilization rate [83.9% ± 0.23 vs 86.5% ± 0.16 (*p*=0.639)], good quality cleavage embryo rate [53.3% ± 0.30 vs 52.4% ± 0.28 (*p*=0.912)] and blastulation rate [55.2% ± 0.27 vs 60.9% ± 0.35 (*p*=0.548)].

**Conclusion:** IVF laboratory results are not affected by the incubation time to ICSI. Therefore, embryologists can perform the fertilization safely from three to six hours after ovarian aspiration.

**P-34. Does the air temperature of IVF laboratory affect good quality blastocyst rate?**

**Bruna Campos Galgaro^1^, Luiza da Silva Rodrigues^1^, Lisiane Knob de Souza^1^, João Sabino Lahorgue Cunha-Filho^1^**

*^1^ Insemine - POA - RS - Brasil.*

**Objective:** To evaluate the influence of IVF laboratory air temperature at the time of ICSI on embryo culture.

**Methods:** Retrospective study including all fresh ICSI cycles from January to June 2023. The temperature of the IVF laboratory was measured with a thermometer at the time of ICSI and the cycles were splitted in two temperature range groups: control (22-25.9ºC) and study (<22ºC or ≥26ºC). Mean age of patients, AMH (ng/mL) and the number of oocytes retrieved were assessed. The primary laboratory outcome compared was good quality blastocyst rate, and the secondary were: fertilization rate, good quality cleavage embryo rate, and blastulation rate. Statistical analysis was conducted by paired test with multiple linear regression, and results were considered significant when *p*<0.05.

**Results:** We included 97 ICSI cycles, enrolled into two groups: 66 were included in the control group (air temperature range of 22-25.9ºC) and 31 in the study group (lower than 22ºC [n=10] or higher than 26ºC [n=21]). There was no statistical difference for the mean age of patients [36.2 vs 35.9 (*p*=0.810)], AMH [2.83 vs 2.31 (*p*=0.359)] and number of oocytes retrieved [8.89 vs 9.64 (*p*=0.669)] for control and study groups respectively. Normal fertilization rate (2PN/2PB) was not statistically different [76.6% vs 78.6% (*p*=0.703)] between both groups, as well as D3 good quality embryo [57% vs 45.1% (*p*=0.062)] and blastulation rates [61.7% vs 48.5% (*p*=0.107)]. However, a statistical significant reduction of good quality blastocyst rate was observed when the temperature in the IVF laboratory at the time of ICSI. The study group presented a reduction 16.5% compared to the control group 44.9% [OR: 0.284 (0.108-0.460), *p*=0.002]. This result was confirmed by multiple linear regression, indicating that the only variable associated with good quality blastocyst rate was the IVF lab temperature (*p*=0.014).

**Conclusion:** The good quality blastocyst rate is greatly affected by the air temperature of the IVF laboratory at the time of ICSI, therefore temperatures lower than 22ºC and higher than 26ºC should be avoided.

**P-35. Impact of pre-diabetes on seminal parameters of patients undergoing assisted reproduction techniques: A retrospective analysis of cases from 2017-2018**

**Mayara Lobato Lourenço Guedes^1^, Gabriel Acacio de Moura^2^, Eduardo Gomes Sá^3^, Ellayne Cavalcanti Queiroz^3^, Gleicyane Sousa Santos Alam^3^, Andrea Mesquita Lima^3^, Eduardo de Paula Miranda^1^, Sebastião Evangelista Torquato Filho^1^, Roberto Nicolete^2^**

*^1^ Sollirium Educação e Pesquisa - Fortaleza - CE - Brasil.*

*^2^ Universidade Federal do Ceará/ Fundação Oswaldo Cruz - Fortaleza - CE - Brasil.*

*^3^ BIOS Centro de Reprodução Humana - Fortaleza - CE - Brasil.*

**Objective:** To verify the impact of pre-diabetes on the seminal parameters of patients undergoing assisted reproduction techniques (ART).

**Methods:** The present work is a retrospective cohort study that consists of the analysis of medical records of patients undergoing ART at the human reproduction clinic Evangelista Torquato (Fortaleza-Ce, Brazil). This study was approved by the ethics and research committee involving human beings under protocol number (58462022.8.0000.5054). To carry out the study, clinical records corresponding to the period from January 1, 2017 to December 31, 2018 were verified. diabetes and medical records of apparently fertile couples (control group). As exclusion criteria: Couples in which the partner had a history of adjacent comorbidities that led to infertility and medical records with incomplete information were excluded. The data extracted from the medical records were: patient's age, glycated hemoglobin, sperm concentration in the ejaculate, motility, sperm morphology, and ejaculate volume. Quantitative seminal analysis data were expressed as mean ± standard deviation. Data referring to continuous quantitative variables were calculated using the t-test. In all tests, a significance value of p < 0.05 was adopted. To perform the statistical analysis, the R studio software (version 4.2.2) was used.

**Results:** Overall, data were obtained from 70 patients in the study: 40 pre-diabetics and 30 patients in the control group. The mean age of patients in the pre-diabetic group was 38.66±1.48 years, while in the control group 38.05±1.28 years and there was no significant difference between them (*p*=0.75). The percentage of glycated hemoglobin among pre-diabetic patients was between 5.67±0.04%, while among patients in the control group it was 5.031±0.04%. Our work showed no significant difference between the ejaculate volume parameter of the prediabetic group compared to the control group (2.80±0.27; 3.03±0.20; *p*=0.49). The same was also verified in the parameter of sperm concentration whose pre-diabetic group presented a mean of 39.03±6.07x10^6^, while the control group presented 45.40±8.73x10^6^ (*p*=0.46). Furthermore, no correlation was found between sperm motility in prediabetic patients and the control group (37.43±3.00; 42.61±3.17; *p*=0.24). Kruger sperm morphology also showed no significant difference between prediabetic and control groups respectively (7.68±1.02; 9.71±2.65; *p*=0.24).

**Conclusion:** Given the above, pre-diabetes conditions may not influence the seminal parameters of patients undergoing ART. However, further studies addressing a larger representative population should be performed in order to validate this hypothesis.

**P-36. Evaluation of the positive beta hCG profile in relation to the mean score of the KIDScore and Mitoscore embryonic analysis systems**

**Mayara Lobato Lourenço Guedes^1^, Gabriel Acacio de Moura^2^, Maria Clara Parente Torquato^1^, Roberto Nicolete^2^, Eduardo Gomes Sá^3^, Ellayne Cavalcanti Queiroz^3^, Matheus Pontes Parente Travassos^4^, Eduardo de Paula Miranda^1^, Sebastião Evangelista Torquato Filho^1^**

*^1^ Solliriium Ensino e Pesquisa - Fortaleza - CE - Brasil.*

*^2^ Universidade Federal do Ceará / Fundação Oswaldo Cruz - Fortaleza - CE - Brasil.*

*^3^ BIOS Centro de Reprodução Humana - Fortaleza - CE - Brasil.*

*^4^ Programa de Pós-Graduação dos Hospitais Universitários da Universidade Federal do Ceará - Fortaleza - CE - Brasil.*

**Objective:** To verify the profile of positive beta-hCG in relation to the mean KIDScore and Mitoscore scores in patients with reproductive complications.

**Methods:** This article is a retrospective cohort study that consists of the analysis of medical records of patients undergoing assisted human reproduction procedures at the BIOS human reproduction clinic (Fortaleza-Ce, Brazil). A review of clinical records corresponding to the period from January 1, 2022 to July 1, 2023 was carried out. As main clinical deciders, patients with confirmed diagnoses of endometriosis, tubal factor problems, adenomyosis and male factor problems were selected. Exclusion criteria were: patients with medical records that contained incomplete information and patients with infertility without apparent cause. The entire selection and sorting of medical records was carried out in an automated way from the Gs Doctor system. The data from the present study included the KIDScore and Mitoscore values, in addition to the beta-hCG result and cause of the patient's infertility. To perform the statistical analysis, the statistical package R studio (version 4.2.2) was used. For which, all quantitative data were represented by mean ± standard deviation. Data referring to continuous quantitative variables were calculated using the t test and the significance value adopted was *p*<0.05.

**Results:** In total, 62 patients were selected for analysis according to the inclusion criteria. Of these, 24 were diagnosed with endometriosis, six with problems in the tubal factor, 18 patients with adenomyosis and 14 with complications in the male factor. In patients with endometriosis, the mean between KIDScore values with positive and negative beta was between 6.89 ± 0.43 and 6.92 ± 0.40 respectively, showing no statistical difference (*p* = 0.95). This was also observed for the mitoscore, which exhibited mean values of 13.94 ± 1.42 and 18.03 ± 1.620 for beta positive and negative patients (*p* = 0.09). All patients with tubal factor included in the study did not obtain positive beta hCG. Patients diagnosed with adenomyosis also did not show significant differences between the means in the KIDScore values when compared to the beta hCG result (6.48±0.91; 5.60±0.60; *p*=0.41). Regarding the mitoscore, beta-positive and beta-negative patients with adenomyosis also did not show significant differences regarding the mean value (14.86±1.69; 15.34±1.97; *p*=0.86). Patients with altered male factor did not show a difference between the KIDScore mean and the beta hCG result (6.48±1.18; 6.55±0.99; *p*=0.96). The mitoscore value for patients with altered male factor also showed no difference when compared to beta-positive and beta-negative patients respectively (14.04 ± 1.46; 16.09 ± 1.35; *p* = 0.32)

**Conclusion:** The present result indicates that the mean between KIDScore and mitoscore scores may not be significantly associated with the rate of clinical pregnancy. However, further studies are needed to confirm this result.

**P-37. Can the use of sperm selection techniques increase the euploidy rate?**

**Jose Gonzales^1^, Gilana Jazmin VillaLobos^1^, Moises Sanchez^1^, Flor Carvallo^1^, Alberto Franco^1^, Jose Pimentel^1^, Jonathan Vasquez^1^**

*^1^ Centro de Fertilidad Germinar - Peru.*

**Objective:** To determine if the use of sperm selection techniques, compared to conventional morphological selection before intracytoplasmic sperm microinjection (ICSI) in a group of fertility patients, generates a significant difference in the rate of embryonic euploidy.

**Methods:** The euploidy results of 181 cycles with donated eggs were analyzed. These procedures were performed as part of the egg donation program of the Germinar Fertility Center (Lima, Peru). The study included 3 groups: 69 cycles with standard morphological selection (ICSI), 56 cycles with selection by physiological ICSI (PICSI) and 56 cycles with selection by annexin columns (CA), for which ejaculated semen samples were used where the initial sample count was greater than 1 million motile sperm. The statistical analysis was by means of a Kruskal Wallis test to compare the proportions of the fertilization, blastulation and euploidy variables with respect to each ICSI, PICSI and CA group.

**Results:** When comparing the number of fertilized eggs (n=608, n=480, n=522), blastocysts (n=332, n=269, n=285) and euploid embryos (n=175, n=138, n =152) for each of the groups, no differences were observed in fertilization rates (ICSI, 81.6%; PICSI, 80.3%; CA, 81.8%, *p*=0.54) and blastulation (ICSI, 54.6%; PICSI, 56.0%; CA, 54.6%, *p*=0.38) among the three groups, an increase in the euploidy rate (72.25%) was observed in the PICSI group compared to CA (66.38%) or conventional ICSI (67.05%), however, it was not statistically significant (*p*=0.29).

**Conclusion:** The use of sperm selection techniques such as PICSI or CA on standard morphology selection does not show differences in the fertilization or blastulation rate and does not generate an increase in the embryonic euploidy rate.

**P-38. Egg reception: a multidisciplinar approach**

**Ariana Fortes de Carvalho^1^, Cassia Cançado Avelar^1^, Luciana Campomizzi Calazans^1^, Cristiane Araujo de Oliveira^1^, Ricardo Melo Marinho^1^, Erica Becker Souza Xavier^1^, Joao Pedro Junqueira Caetano^1^**

*^1^ Huntington Pro-Criar - Belo Horizonte - MG - Brasil.*

**Objective:** Demonstrate, through numbers, the experience of egg reception patients, with a multidisciplinary approach, in a particular assisted reproduction clinic.

**Methods:** Retrospective analysis of the follow-up of patients who sought an in vitro fertilization treatment with egg reception, highlighting aspects related to evaluation of psychology and nursing.

Results: A total of 131 patients were indicated and underwent a treatment of egg reception between 2021 -2022 in a private reproductive medicine clinic in Belo Horizonte/Brazil. The average age of people who underwent treatment was 43 years. 127 (96.9%) patients did the treatment with anonymous egg donation; 11 (8.3%) patients did the treatment with egg and semen donated; 04 (3.0%) patients did the treatment with egg donation and surrogacy and 03 (2.,2%) patients did the treatment with family egg donation. All of them had psychological and nursing consultations. Treatment outcome resulted in 64 (48.8%) positive result in the first treatment and 27 (20.6%) in another attempt; 17 (12.9%) gestational loss; 35 (26.7%) negative results. The patients with gestational loss and negative result had a return with doctor, psychology and nurse, 15 (11.4%) patients decided to take a new treatment and 11 (8.3%) decided gave up treatment.

**Conclusion:** Receiving donated eggs raises several feelings and doubts about the treatment and its possibilities. When patients are welcomed by a specialized and trained team a space is opened, so that the questions can be verbalized and validated and the acceptance and outcome of the treatment can be more favorable.

**P-39. How ICSI with PGT-A impact success pregnancy rates across women age groups?**

**Isadora Braga Seganredo^1^, Victor Rega Lazar^2^, Alecsandra Prado Gomes^2^, Hamilton De Martin^2^, Elizabeth Noemia Nanni Martinez^2^, Tatiana Carvalho Souza Bonetti^3^, Pedro Augusto Araújo Monteleone^2^**

*^1^ Disciplina de Ginecologia - Departamento de Obstetrícia e Ginecologia, Faculdade de Medicina da Universidade de São Paulo - São Paulo - SP - Brasil.*

*^2^ Centro de Reprodução Humana Monteleone - São Paulo - SP - Brasil.*

*^3^ Departamento de Ginecologia, Escola Paulista de Medicina da Universidade Federal de São Paulo - São Paulo - SP - Brasil.*

**Objective:** Preimplantation genetic testing for aneuploidy (PGT-A) is a largely used tool in embryo selection on the grounds of euploidy. Formally, PGT-A has only few indications, such as recurrent implantation failure (RIF), recurrent miscarriage, couples with karyotype alterations or personal history of sex related hereditary diseases. However, it’s use has been routinely expanded for a wide group of patients, such as advanced maternal age (AMA), cases of severe male infertility and even for women at all ages with no formal indication, followed by an elective single embryo transfer as an attempt of increasing the pregnancy success rates in a shorter time and reduce the abortion incidence. This study aims to evaluate the ongoing pregnancy success of ICSI cycles with PGT-A and euploid blastocyst transfer, according to maternal age, using real world data.

**Methods:** Retrospective cohort including 226 ICSI cycles with PGT-A conducted at a private fertility center in São Paulo, Brazil, between 2019 and 2022 were included. The inclusion criteria were ICSI cycles using own oocytes, with embryos until blastocyst development followed by blastocyst biopsy for PGT-A on Day 5 of development. The frozen-thawed euploid D5 blastocyst transfers were performed in a subsequent hormone replacement cycle. Women included were 29 to 45 years old (yo) and the clinical outcomes are presented according to women ages ranges: <35 yo (n=44); 35-37 yo (n=61); 38-40 yo (n=78); 41-42 yo (n=33); >42 yo (n=10).

**Results:** The average age for each group were: <35 (32.6±1.8); 35-37 (36.1±0.9); 38-40 (38.9±0.8); 41-42 (41.3±0.5); >42 (43.2±0.6); *p*<0.001. The mean number of cycles per couple (1.3±0.7; 1.5±0.7; 1.9±1.6; 2.0±1.8; 1.7±1.0; *p*=0.298) and the number of blastocysts transferred were similar in all groups (1.2±0.4; 1.1±0.3; 1.1±0.3; 1.0±0.0; 1.2±0.6; *p*=0.085), respectively. Clinical outcomes evaluated included total pregnancy (bhCG+) (<35: 59%; 35-37: 39%; 38-40: 65%; 41-42: 61%; >42: 70%, *p*=0.369), miscarriage (<35: 35%; 35-37: 33%; 38-40: 36%; 41-42: 26%; >42: 17%, *p*=0.854) and ongoing pregnancy rates (<35: 39%; 35-37: 33%; 38-40: 42%; 41-42: 44%; >42: 56%, *p*=0.629). The multivariate logistic regression analysis confirmed that the ongoing pregnancy is not associated to women age: <35 (reference); 35-37 (OR 0.88; *p*=0.767; 38-40 (OR 1.35; p=0.461); 41-42 (OR 1.57; *p*=0.362); >42 (2.07; *p*=0.346), adjusted for confounders (number of cycles and number of embryos transferred).

**Conclusion:** This study based on real-world data demonstrated that the woman age has no impact on clinical pregnancy rates when a D5 euploid blastocyst transfer is placed. Those outcomes are similar to SART National Report Summary, which also shows similar live birth rates per transfer when PGT-A is placed, for all women ages rages. It is important to note the limitation of our study, as we presented clinical outcomes per blastocyst transfer, while cumulative outcomes per started cycle were not available. It is known that the number of oocytes obtained and the rate of euploid blastocysts decrease as the woman's age increases. Thus, the woman age can had been an impact on the availability of euploid embryos for transfer. On the other hand, we have shown here, once a euploid blastocyst is available and transferred, the success chances are similar at all women ages.

**P-40. Additional contribution of the stable environment in time-lapse incubator for women over 38 years of age**

**Mayra Satiko Lemos Nakano^1^, Priscila Melantonio^1^, Alecsandra Prado Gomes^1^, Amanda Turato Barbosa Amaral^1^, Janaina Gisela de Brito Cunha^1^, Tatiana Carvalho Souza BonettI^2^, Pedro Augusto Araújo Monteleone^1^**

*^1^ Centro de Reprodução Humana Monteleone - São Paulo - SP - Brasil.*

*^2^ Departamento de Ginecologia, Escola Paulista de Medicina da Universidade Federal de São Paulo - São Paulo - SP - Brasil.*

**Objective:** Since the advent of the time-lapse incubator, it has been widely used around the world with the aim of selecting the best quality embryo based on morphokinetic characteristics. However, the time-lapse system allows continuous observation of the embryos without removing them from the incubator, which can bring other benefits due to the more stable culture environment than conventional incubators. The aim of this study was to evaluate blastocyst development and ongoing pregnancy rates according to the type of incubator used for embryo culture in women over 38 years old (≥38yo), compared with younger women (<38yo), undergoing ICSI cycles.

**Methods:** This was a retrospective cohort study including 177 ICSI cycles performed between December 2020 and March 2021 in a private IVF clinic in São Paulo. In those period, conventional and time-lapse incubators were being used concomitantly in the embryology laboratory. Embryos were cultured in one of the incubator randomly. The embryos selection for transfer was based on morphological characteristics, independently of incubator. We have excluded fertility preservation cycles, oocyte donation cycles or frozen-thawed oocyte cycles, and 137 ICSI cycles were included in this analysis, as 80 cycles in the time-lapse incubator (TL group) and 57 in the conventional incubator (CONTROL group). Cycles were also split according to women age, as <38yo or ≥38yo.

**Results:** For women ≥38yo, the outcomes of cycles included in the TL (n=37) and CONTROL (n=32) groups were compared. The demographic characteristics were similar (age: 40.5±2.0 and 40.5±1.6; *p*=0.664 / presence of male factor: 8% and 6%; p=0.740 / MII collected: 5.5±4.4 and 5.9±4.4; *p*=0.610, respectively). Despite of cycles had equivalent basement characteristics, the blastocyst development rate was higher in embryos cultured in time-lapse than conventional incubator (53.9% versus 34.1% *p*=0.019). Moreover, cumulative pregnancy rate was expressively higher in TL group compared to conventional (33.0% and 19.0%; *p*=0.214), despite of no statistically significant, in women ≥38yo. For younger women (<38yo), the cycles included in the TL (n=43) and CONTROL (n=25) groups were compared (age: 35.8±4.6 and 36.4±5.1; *p*=0.735 / presence of male factor: 14% and 16%; p=0.818 / MII collected: 13.1±11.7 and 7.1±4.2; *p*=0.014, respectively). The blastocyst development rates were similar (56.7% and 59.3%; *p*=0.673) and cumulative ongoing pregnancy rates did not differ between groups (50.0% and 48.0%; *p*=0.865). The multiple logistic regression model confirmed the higher blastocyst development rate is associated to increased chances of pregnancy (OR 0.03; *p*= 0.038) in women ≥38yo; but it do not in younger women (OR: 0.01; *p*=0.529) adjusted for confounders (MII oocytes collected and PGT-A).

**Conclusion:** When embryo selection is based in the morphological characteristics, the embryo culture in a time-lapse incubator does not seem to affect the laboratorial or clinical outcomes for young women (<38yo). On the other hand, for women ≥38yo, the time-lapse incubator contributes to promoting a higher rate of blastocyst, which in turn is associated with greater chances of pregnancy. Therefore, the more stable environment of time-lapse incubator during culture time seems to support blastocyst formation when the oocytes are impaired by the advanced women’s age.

**P-41. Understanding the implications of follicular output rate (FORT) and follicle to oocyte index (FOI) on human embryo morphokinetics**

**Daniela Paes Almeida Ferreira Braga^1^, Amanda Souza Setti^1^, Mario Firmino^2^, Christina Morishima^2^, Assumpto Iaconelli Jr^1^, Edson Borges Jr^1^**

*^1^ Fertility Medical Group / Instituto Sapientiae - Sao Paulo - SP - Brasil.*

*^2^ Fertility Medical Group - São Paulo - SP - Brasil.*

**Objective:** To evaluate the effects of follicular output rate (FORT) and follicle to oocyte index (FOI) on embryos morphokinetics.

**Methods:** This historical cohort study was performed in a private university-affiliated IVF centre between February 2019 and December 2021. Kinetic data were analysed in 8,376 embryos, individually cultured in a time-lapse imaging (TLI) incubator and derived from 2,470 patients undergoing ICSI cycles. The timing of specific events from the point of insemination was determined using TLI. Recorded kinetic markers were: timing to pronuclei appearance (tPNa) and fading (tPNf), timing to two (t2), three (t3), four (t4), five (t5), six (t6), seven (t7), and eight cells (t8), and timing morulation (tM), timing to start blastulation (tSB) and to blastulation (tB). The durations of the second (cc2, t3-t2) and third cell cycles (cc3, t5-t3) and the timing to complete synchronous divisions t2-tPNf (s1), t4-t3 (s2) and t8-t5 (s3) were also calculated. The effects of FORT and FOI the levels on morphokinetic events and ICSI clinical outcomes were investigated. For that, embryos were split into groups according to FOI value: FOI< 50 (Low-FOI, n=247 cycles and 894 embryos) and FOI≥ 50 (high-FOI, n=2,223 cycles and 7,482 embryos) and according to the FORT value: FORT values below the 33rd percentile, FORT< 27.3 (low-FORT, n= 753 cycle and 2,556 embryos), FORT values between the 33rd and the 67th percentile, FORT: 27.3-47.6 (medium-FORT, n=874 cycles and 2,970 embryos), and FORT values above the 67th percentile, FORT> 47.6 (high-FORT, n=843 cycles and 2,850 embryos). Embryo morphokinetics and ICSI outcomes were compared among the FOI and FORT groups.

**Results:** A significant difference was noted in almost all morphokinetic parameters, where embryos derived from cycles with an FOI <50 presented slower development than embryos derived from cycles with an FOI <50, considering tPNf, t2, t4, t6, t7, t8, tM, tB, s1, s2, s3, and cc2. A significantly higher KID score D5 was observed among embryos derived from cycles with an FOI ≥ 50 when compared with those with an FOI < 50 (5.60 ± 0.03 vs. 5.1 ± 0.09, *p*< 0.001). Additionally, increased blastocyst formation (53.6 ± 0.92 vs. 44.85 ± 1.87, *p*< 0.001) and implantation (24.8 ± 0.32 vs. 26.08 ± 0.53, *p*=0.037) rates were noted among cycles with higher FOIs. An increased time to complete morphokinetic events was observed among embryos derived from cycles with low FORT, followed by those with medium FORT, while embryos derived from cycles with high FORT presented a faster development competence: tPNa, t2, t4, t5, t6, t7, t8, tsB, s2, s3. Embryos derived from cycles with high FORT presented a higher Kid Score D5, followed by those derived from cycles with medium FORT, and embryos from cycles with low FORT presented the lowest KID score (5.4 ± 0.5; 5.5 ± 0.5; and 5.6 ± 0.6, *p*=0.021). Significantly higher rates of blastocyst formation and implantation were observed in embryos derived from cycles with high FORT, followed by those with medium FORT, while embryos from cycles with low FORT presented the lowest blastocyst formation (49.2% ± 1.4; 50.8% ± 1.4; and 55.5% ± 1.4, *p* < 0.001) and implantation rates (23.6% ± 0.4; 24.5% ± 0.4; and 27.1 ± 0.5, *p*< 0.001).

**Conclusion:** In conclusion, this study highlights the association between FORT and FOI and embryo morphokinetic development. Significant positive relationships were observed between embryo development and these parameters, which would not be detected in embryos cultured in conventional incubators. It is possible that the deselection of these embryos may have prevented an effect on clinical pregnancy, but additional research is needed to validate this assumption.

**Table 1:** Comparison of known implantation diagnosis (KID) score D5 and intracytoplasmic sperm injection (ICSI) outcomes between the low and high Follicle-To-Oocyte (FOI) index groups and between the low, medium and high follicular output rate (FORT) groups.

Variable	Low FOI	High FOI	p value	Low FORT	Medium FORT	High FORT	p value
**n**	894	7532		2,556	2,970	2,850	
**Kid score**	5.1 ± 0.09	5.60 ± 0.03	< 0.001	5.4 ± 0.5^a^	5.5 ± 0.5 ^a,b^	5.6 ± 0.6 ^b^	0.021
**Blastocyst rate (%)**	53.6 ± 0.92	44.85 ± 1.87	< 0.001	49.2 ± 1.4^a^	50.8 ± 1.4 ^a^	55.5 ± 1.4 ^b^	< 0.001
**Implantation rate (%)**	24.8 ± 0.32	26.08 ± 0.53	0.037	23.6 ± 0.4^a^	24.5 ± 0.4 ^a^	27.1 ± 0.5 ^b^	< 0.001

**P-42. The predictive value of post warming morphokinetic factors for embryo implantation**

**Melissa Cavagnoli^1^, Daniela Paes Almeida Ferreira Braga^2^, Amanda Souza Setti^2^, Patricia Guilherme^3^, Assumpto Iaconelli Jr^2^, Edson Borges Jr^2^**

*^1^ Clinica Hope - São Paulo - SP - Brasil.*

*^2^ Fertility Medical Group / Instituto Sapientiae - São Paulo - SP - Brasil.*

*^3^ Fertility Medical Group - São Paulo - SP - Brasil.*

**Objective:** Even though the initial blastocyst survival rate after vitrification and warming surpasses 90%, vitrified human blastocysts show varied re-expansion capacity after warming and little is known about the effect of embryo behavior after warming on implantation potential. Therefore, the goal for the present study was to investigate whether frozen-thawed blastocyst morphokinetics, observed using time-lapse imaging (TLI), may be an indicator for implantation in intracytoplasmic sperm injection (ICSI) cycles, with deferred embryo transfers (freeze-all cycles).

**Methods:** From January to October/2022, an observational cohort study was conducted at a private university-affiliated IVF center. This included 411 vitrified/warmed blastocysts with known implantation data (no implantation and full implantation per transfer), which were evaluated by TLI. Blastocysts were incubated in EmbryoScope+ (Vitrolife, Denmark) for up to 4 hours, immediately after warming until transfer. Blastocysts morphological dynamics variables after warming were recorded, and their potential association with clinical pregnancy were investigated. The recorded variables were: (i) the initial blastocyst area (IBA) and zona pellucida thickness (IZP) immediately after warming, (ii) the maximum blastocyst area (MBA) and the minimum ZP thickness (MZP) immediately before transfer, and (iii) expansion (whether the embryo re-expands or not after warming). Embryos were split into percentiles groups according to the values of IBA, MBA, IZP, and MZP. For each parameter, values below the 33rd percentile, between the 33rd and the 67th percentile, and values above the 67th percentile were recorded. Embryos received a final score from 0 to 9, according with the sum of the scores from each parameter, in each percentile, and the influence of the score on embryos implantation was evaluated.

**Results:** The average maternal age in the study population was 37.4 ± 5.0 years old. The after warming factors values divided according with < 33rd percentile, from 33rd to 67th percentile, and > 67th percentile were respectively: IBA: 9611.1 µm2, 11851.4 µm2, and 26845.0 µm2; MBA: 14110.0 µm, 18572.6 µm2, and 93381.0 µm2; IZP: 13.0 µm, 16.0 µm, and 28.0 µm, and MZP: 11.0 µm, 14.0 µm, and 23.0 µm. No significant effect of after warming parameters on implantation results were observed when the parameters were individually evaluated. Clinical outcomes are given in Table 1. A significant increase in the implantation rate was noted among embryos presenting higher scores, followed by those with median scores, while embryos with lower scores presented the lowest implantation rate. Miscarriage rates tended to be higher in the low and medium total score groups compared to high total score group, however statistical significance was not reached. The logistic regression analysis showed that embryos in the low total score group present a 12.3% decreased chance of implantation when compared with embryos in the high total score group (OR: 0.877, CI: 0.843 - 0.912, *p*<0.001), while embryos in the medium total score group presents a 9.6% decreased implantation chance when compared with embryos in the high score group (OR: 0.904, CI: 0.870 - 0.939, *p*<0.001).

**Conclusion:** The blastocyst behavior after warming, by evaluating the blastocyst area, the ZP thickness, and re-expansion, together with the pre-vitrification embryo classification may provide important information for selecting the best embryo for transfer or in managing the patient´s expectations.

Values are percentage ± standard error, unless otherwise noted. a ≠ b ≠ c.

**Table 1:** Comparison of maternal age, implantation, and miscarriage rates between embryos in the low total score, medium total score, and high total score groups.

Score Groups	Low Score	Medium Score	High Score	P value
**Maternal age (years old)**	37.8 ± 5.3	37.2 ± 5.2	37.4 ± 5.7	0.743
**Implantation rate (%)**	34.8 ± 5.3^a^	35.9 ± 5.2^b^	39.7 ± 5.2^c^	<0.001
**Miscarriage rate (%)**	10.0 ± 4.2	13.2 ± 4.1	5.1 ± 2.9	0.239

Values expressed as percentage or average ± standard deviation. a≠b≠c.

**P-43. Association between meteorological season at the time of embryo transfer and pregnancy outcomes in freeze-all cycles: lessons from seasonal reproductive mammals**

**Amanda Souza Setti^1^, Daniela Paes Almeida Ferreira Braga^1^, Edward Carrilho^2^, Patricia Guilherme^2^, Assumpto Iaconelli Jr^1^, Edson Borges Jr^1^**

*^1^ Fertility Medical Group / Instituto Sapientiae - São Paulo - SP - Brasil.*

*^2^ Fertility Medical Group - São Paulo - SP - Brasil.*

**Objective:** Extensive research has consistently shown that most mammals are seasonal breeders, and as day length increases, gonadotropin secretion increases, to anticipate reproduction in the spring and summer months. There is intriguing evidence that there is a seasonal variance in human reproduction, but the data are inconclusive. Therefore, the goal for the present study was to evaluate the effect the meteorological season at the time of embryo transfer on clinical outcomes of freeze-all cycles.

**Methods:** This historical cohort study, performed in a private university-affiliated IVF centre between February 2018 and December 2022, included 4952 frozen-thawed embryos transferred to 3725 cycles in a single clinic. Both vitrification and the warming procedures were performed using the Cryotop method. Embryos survival and clinical outcomes were analyzed by the season, at the time of embryo transfer, using generalized linear models adjusted for potential confounders.

**Results:** The mean maternal age was 37.0 ± 4.7 years. Embryos were cryopreserved for a mean of 8.1 ± 17.3 months. Autumn was designated as the reference season for all analyses. No significant differences were observed in embryo survival rates when the seasons were compared, however, lowest implantation and pregnancy rates were observed when embryo transfers occurred in autumn (44.6%), followed by winter (45.8%, OR: 1.049, CI: 0.876 - 1.257, *p*=0.603) and summer (49.0%, OR: 1.196, CI: 1.010 - 1.415, *p*=0.038), while spring embryo transfers had a 30% increased odds of pregnancy (51.2%, OR: 1.302, CI: 1.100 - 1.541, *p*=0.002). An increased miscarriage rate was observed when embryo transfers occurred in autumn (13.9%), followed by winter (11.4%, OR: 0.510, CI: 0.323 - 0.805, *p*=0.004) and summer (10.4%, OR: 0.461, CI: 0.292 - 0.727, *p*=0.001), while spring embryo transfers had a 63% decreased odds of miscarriage (5.6%, OR: 0.369, CI: 0.230 - 0.592, *p*<0.001).

**Conclusion:** In seasonal breeding mammals, melatonin, synthesized in the pineal gland, leads increased release of gonadotropins during the transition period (between anestrus and estrus onset, usually after winter and before summer). Melatonin rise has been proved to favor embryo implantation and protect offspring by increasing progesterone levels. In the present study, optimal conditions for embryo implantation appeared to be associated with spring and summer. In contrast, frozen embryo transfers performed in autumn and winter are associated with reduced ongoing pregnancy rates. Our evidence raises the question of how human reproductive potential may be influenced by climatic variations and whether this information may be useful in assisted reproduction cycles.

**P-44. Oocyte maturation rate after double trigger**

**Kahisa Natiele Fontana^1^, Thainá Carlesso Setoyama^2^, Rodrigo Santos Andrade^1^, Regis Yukio Cho^1^, Lidio Jair Ribas Centa^1^, Priscila Cristina Almeida^1^, Camila Rambo^2^**

*^1^ Androlab - Clínica da Fertilidade - Curitiba - PR - Brasil.*

*^2^ CHC-UFPR - Curitiba - PR - Brasil.*

**Objective:** Coadministration of GnRH agonist and human chorionic gonadotropin (hCG) in IVF cycles is associated with a higher number of oocytes, mature oocytes and number of blastocysts compared to triggering with hCG alone. hCG is used in order to simulate an LH surge at the end of controlled ovarian hyperstimulation to induce final oocyte maturation. The combination of GnRH agonist and low dose hCG, called "double trigger", has been suggested by previous studies as a method to improve IVF outcome and pregnancy rates (HAAS, J. et al., 2020). The aim of this study is to evaluate oocyte maturity at metaphase II (MII) rate with the use of Double Trigger versus Single Trigger.

**Methods:** The respective study, performed at Androlab, an Assisted Reproduction Technology (ART) Center located in Curitiba, Brazil, between january/2021 and july/2023. All cycles of Intracytoplasmic Sperm Injection (ICSI) performed at this center were included. Patients were separated into five groups, two of which underwent the double trigger procedure, while the other three underwent the single trigger.

**Results:** The investigation evaluated a total of 749 patients, of whom 254 underwent the double trigger procedure with a combination of Gonapeptyl (GnRH agonist) and Choriomon (hCG) or Gonapeptyl and Ovidrel (hCG); and the other 495 underwent the single trigger with administration of Gonapeptyl, Ovidrel or Choriomon alone. 2328 of the 3378 recovered oocytes from patients receiving Choriomon alone were MII oocytes, resulting in a maturity rate of 69%. In patients who received Ovidrel alone, 35 of the 51 recovered oocytes were MII oocytes, also resulting in a maturity rate of 69%. 1341 of the 1851 recovered oocytes from patients receiving Gonapeptyl alone were MII oocytes, amounting to a maturity rate of 72%. Whereas in the case of double triggers, 77 of the 99 recovered oocytes from patients who received the association of Gonapeptyl and Ovidrel reached MII, resulting in a maturity rate of 78%. In patients who received the association of Gonapeptyl and Choriomon, 1892 of the 2528 recovered oocytes were MII, which constitutes a maturity rate of 75%. Without distinguishing between the drugs used, the study found that 3704 of the 5280 recovered oocytes from patients receiving a single trigger were MII, resulting in a 70% maturity rate, while 1969 of the 2627 recovered oocytes from patients receiving a double trigger reached MII, accounting for a 75% maturity rate.

**Conclusion:** The use of the double trigger showed better oocyte maturity rate results compared to the single trigger. Limitations of the present study include the difference in the amount of follicles of the two groups (single trigger and double trigger), data from a single institution and retrospective design.

**P-45. Rate of euploidy and embryonic implantation of blastocysts biopsied on D6 and D7**

**Amanda Cerquearo Rodrigues dos Santos^1^, Luiz Mauro Oliveira Gomes^1^, Letícia Emi Kadomoto Ferreira^1^, José Fernando Macedo^1^**

*^1^ Clínica Reproferty - São José dos Campos - SP - Brasil.*

**Objective:** The objective of this study was to evaluate the rate of euploidy, from the Preimplantation Genetic Test for Aneuploidies (PGT-A), and the embryonic implantation of biopsied blastocysts in 6 days of culture (D6) and 7 days of culture (D7).

**Methods:** Retrospective study was conducted at Reproferty Clinic, evaluating patients who underwent infertility treatment using the intracytoplasmic sperm injection (ICSI) technique, followed by embryonic biopsy on D6 or D7, and Preimplantation Genetic Testing for Aneuploidies (PGT-A) from July/2021 to July/2023. The decision on the day of biopsy was based on the stage of embryonic development, with advanced-stage embryos at the stage of expansion and hatching undergoing biopsy on the 5th day of culture (D5), while those with lesser expansion or that had not yet hatched were kept under observation for biopsy on D6 or D7. In each report, the presence of biopsied embryos on D6 and D7 was verified, and their respective results classified them as “Euploid” and “Aneuploid”. The clinical history was used to determine whether or not embryo transfer was performed, and the transfers were classified as: no transfer, no transfer of D6/D7 embryos, positive Beta HCG, negative Beta HCG, and miscarriage. After data collection, the percentages were calculated and tabulated in spreadsheets.

**Results:** A total of 100 Preimplantation Genetic Testing for Aneuploidies (PGT-A) reports from different patients were examined, encompassing 348 analyzed embryos. Among these, 42 patients had embryos biopsied on Day 6 (D6), resulting in 75 embryos, with 25% being euploid and 75% aneuploid. Three patients had embryos biopsied on Day 7 (D7), resulting in 3 embryos, with 33% being euploid and 67% aneuploid. Regarding the transfer of euploid blastocysts on D6, 37% of the patients have not yet undergone the transfer, 32% did not transfer D6 embryos but transferred D5 embryos, 25% transferred one D6 embryo resulting in a negative Beta HCG test, 0% experienced a miscarriage, and only 5% transferred D6 embryos resulting in a positive Beta HCG test. Only one D7 blastocyst was euploid, and it was transferred, resulting in a positive Beta HCG test.

**Conclusion:** The results suggest a high prevalence of aneuploidy in D6 and D7 blastocysts and a low implantation rate of euploid D6 blastocysts, which is consistent with the literature. However, considering that advanced maternal age is a predisposing factor for delayed blastocyst growth, extending embryo culture to D6 and D7 may be a relevant strategy to increase the success rate of in vitro fertilization, necessitating an individualized assessment of each clinical case. It is important to acknowledge that this is an observational study with a limited sample size of euploid D7 blastocysts and that individual factors of the couple may influence euploidy and implantation outcomes.

**P-46. An observational study involving 440 screened embryos: correlation between euploidy, morphology, maternal age and blastocyst stage (Day 5/Day 6)**

**Samara Artuso Giacomin^1^, Camila Dutra Souza Francisquini^1^, Vinicius Bonato Rosa^2^, Alessandro Schuffner^1^**

*^1^ Conceber Centro de Medicina Reprodutiva - Curitiba - PR - Brasil.*

*^2^ Brown Fertility - Florida Fertility Clinics - Estados Unidos.*

**Objective:** The embryo cryopreservation technique combined with preimplantation genetic screening has been widely used to optimize results in treatments of In Vitro Fertilization (IVF). The objective was to determine the correlation between conventional blastocyst morphological evaluation, blastocyst stage (Day 5/Day 6) and chromosomal composition of these embryos into different age groups.

**Methods:** Retrospective observational study from 2020 to 2023, including 440 embryos biopsied and analyzed by Next Generation Sequence for Preimplantation Genetics Test for aneuploidies. Data were collected from blastocyst morphology, blastocyst stage (Day 5/Day 6), euploidy rate and number of chromosomal alterations, from patients submitted to IVF treatment. Data were categorized in: age (G1: <35 years; G2: 35-37 years; G3: 38-40 years; G4: >41 years), blastocyst morphology (Excellent: AA; Good: AB/BA; Average: BB/AC/CA; Poor: BC/CB/CC) and blastocyst stage (Day 5 - 120 hours post-ICSI; Day 6 - 144 hours post-ICSI). Inclusion criteria: fresh and cryopreserved oocytes, homologous and heterologous; and ejaculated and surgically retrieved, fresh and cryopreserved, homologous and heterologous semen, only embryos who reached the expanded stage on day 5 or day 6. Statistical analysis was performed using a two-porportions Z-test (p<0.05).

**Results:** For general euploidy rate, there was no difference (p>0.05) between Day 5 (48.2%) and Day 6 embryos (38.6%). According to the age, it was observed that in G1, the euploidy rate was similar between Day 5 and Day 6 (57.1% and 42.9% respectively, *p*>0.05), however, in the other groups, this rate was higher (*p*<0.05) on Day 5 (G2: 66.0% vs 34.0%; G3: 77.5% vs 22.5%; and G4: 80.0% vs 20.0%). In groups G1, G2 and G3 there was a higher aneuploidy rate in poor embryos (51.4%; 45.5%, 36.3% respectively, *p*<0.05) compared to excellent embryos (10.8%; 9.1%; 23.5%), good (18.9%; 21.8%; 18.6%) and average (18.9%, 23.6% and 21.6%) for the same age groups, while in the G4 group, there was no difference (*p*>0.05) for the aneuploidy rate between the blastocyst morphologies (Excellent: 14.4%; Good: 24.4%; Average: 30.6% and Poor: 30.6%). In all groups, there was a higher percentage (*p*<0.05) of embryos with only 1 chromosomal alteration (G1: 70.3%; G2: 76.4%; G3: 58.8%; G4: 58.0%) when compared with 2, 3 or multiple alterations. Embryos with multiple abnormalities appear evenly distributed between age groups (G1: 10.8%; G2: 5.5%; G3: 7.9% and G4: 6.0%, *p*>0.05).

**Conclusion:** In patients aged ≥35 years, faster growing blastocysts (Day 5) had higher euploidy rate. The relationship between morphology and euploidy in patients up to 40 years old, indicated that poor embryos showed a higher aneuploidy rate. However, >41 years old, all morphology should be considered for biopsy because in this group the traditional morphology selection cannot be used as euploidy predictive, justifying the strong indication of preimplantation genetic screening. These data can collaborate for the individualization of cases and optimizing the cycle management in the embryology laboratory, helping patients regarding the chances of finding euploid embryos in their age group and blastocyst quality.

**P-47. Analysis of aneuploidies in blastocysts of multinucleated blastomeres using the Preimplantation Genetic Test (PGT-A)**

**Amanda Cerquearo Rodrigues dos Santos^1^, Luiz Mauro Oliveira Gomes^1^, Letícia Emi Kadomoto Ferreira^1^, José Fernando Macedo^1^**

*^1^ Clínica Reproferty - São José dos Campos - SP - Brasil.*

**Objective:** The objective of this study was to analyze numerical chromosomal alterations in blastocysts using Preimplantation Genetic Testing for Aneuploidy (PGT-A), correlating them with the presence of multinucleation, degree of fragmentation, maternal age, and subsequent pregnancy after the transfer of a euploid embryo.

**Methods:** A retrospective study was conducted at Reproferty clinic involving patients who underwent infertility treatment using intracytoplasmic sperm injection (ICSI), followed by embryonic biopsy and Preimplantation Genetic Testing for Aneuploidy (PGT-A), from March 2021 to March 2023. Data were collected regarding patient name, age, biopsy date, identification of analyzed embryos, and PGT-A results. Time-lapse technology was used to assess potential morphological alterations such as multinucleation and degree of fragmentation, based on retrospective images from the EmbryoViewer software, integrated with the EmbryoScope incubator. All embryos identified in the PGT-A reports were checked and classified based on nuclear morphology (One nucleus, Binucleation, Multinucleation) and degree of fragmentation (Grade I: fragmentation < 10%; Grade II: fragmentation 10-25%; Grade III: fragmentation 25-50%; Grade IV: fragmentation > 50%). The clinical history was used to determine whether embryo transfer was performed or not, classifying the outcomes as: no transfer, no euploid embryos, positive Beta hCG, negative Beta hCG, biochemical pregnancy, ectopic pregnancy, and miscarriage. After data collection, percentages were calculated, and statistical analysis was performed. The groups were analyzed using the Chi-square test, considering a significance level of 99% (p<0.01), and correlation analysis was conducted.

**Results:** During the study period, 89 patients with a mean age of 40 years underwent PGT-A. A total of 371 embryos were analyzed from 113 cycles, with 66% (245 embryos) resulting in aneuploidies. Approximately 94% of patients over 40 years old obtained at least one aneuploid embryo. The tables below show the classification of embryos in each study group, along with the percentage of different classifications, indicated within parentheses, in relation to the "Euploid" and "Aneuploid" groups.

**Table:** 

	Grade I	Grade II	Grade III	Grade IV	Total
**Euploid**	**47 (0.37)**	**52 (0.41)**	**22 (0.18)**	**5 (0.04)**	**126 (1.0)**
**Aneuploid**	**4 (0.02)**	**78 (0.32)**	**140 (0.57)**	**23 (0.09)**	**245 (1.0)**

**Table:** 

	1 Nucleus	Binucleation	Multinucleation	Total
**Euploid**	**97 (0.77)**	**11 (0.09)**	**18 (0.14)**	**126 (1.0)**
**Aneuploid**	**110 (0.45)**	**64 (0.26)**	**71 (0.29)**	**245 (1.0)**

**P-48. Impacts of the absence of legislation on assisted reproductive technology in Brazil**

**Thais Meirelles de Sousa Maia R^1^, Luciana Batista Munhoz^1^**

*^1^ Maia & Munhoz Advocacia - Brasília - DF - Brasil.*

**Objective:** Assisted reproductive technology (ART) is a multidisciplinary field of study, which allows people with any reproductive obstacles to be able to generate offspring. ART techniques changed human relationships and presented conflicts and ethical dilemmas. Over the decades ART implemented several changes, however Brazilian legislation has not followed suit, which means up to this day we do not have a Law on the subject. The task of regulating the issue was left to the Brazilian Federal Council of Medicine, Conselho Federal de Medicina (CFM), which has no Legislative Power. All legislative power is vested in the Federal Congress, meaning that it is the only part of the government that can make new laws or change existing laws. Therefore, CFM regulations are only administrative in nature, not enforceable to patients and other professionals involved in ART procedures. Those regulations only have ethical implications for professionals and medical institutions.

**Methods:** For this research we used the qualitative method and a survey of Brazil's legislation on ART.

**Results:** The absence of legislation on ART means that its practitioners suffer a direct impact on the relationships with patients and other health professionals. Some example of these impacts:

- The administrative nature of CFM regulations does not bring any implications to patients since these regulations are directed to medical professionals and medical enterprises. Thus, in many situations that the current resolution (Resolution CFM nº 2320/2022) does not cover, patients may propose treatments considered ethical violations;

- The absence of a specific law on ART causes legal uncertainty both for patients and for clinics, laboratories and professionals, since there are no consolidated rights and duties;

- The documentation, as well as the entire internal flow of clinics and laboratories tends to be flawed and exposes all these actors, that are part of the ART process, to eventual damages or worse to civil litigation;

- Situations not foreseen in the CFM resolutions cause doubts and insecurities on the day-to-day activities of ART professionals;

- CFM resolutions are not usually accompanied by explanatory memoranda, which causes doubts, especially of interpretation, for ART professionals, when substantial changes occur between resolutions.

Despite the examples described, the CFM resolutions are extremely important for ART practice. A very positive aspect of CFM's resolutions is the fact that the process of developing and voting on these resolutions is dynamic, different from the legislative process, which can take years and therefore lag behind scientific and social development.

**Conclusion:** All in all, it would be of great value for ART to rely on the existence of a specific law that addresses general aspects, guarantees rights and defines duties to patients, professionals and institutions, for example the right to discard cryopreserved embryos. CFM resolutions play an important role in ART and should continue to be encouraged, focusing on technological changes and the dynamism that ART represents to society.

The Chi-square test yielded a p-value of <0.001 for both nucleation and degree of fragmentation. These results indicate a significant association between the number of aneuploid embryos and nucleation variables, as well as fragmentation grades II and III. Similarly, a correlation was observed between euploid embryos and nuclear normality and fragmentation grade I. Regarding fragmentation grade, the analysis demonstrated a positive correlation with aneuploid embryos, indicating a direct relationship, while a negative correlation was observed with euploid embryos, indicating that higher fragmentation grades correspond to a lower number of euploid embryos. After the PGT-A results were released, 28% of patients did not undergo embryo transfer due to aneuploidy in all analyzed embryos, 16% had not undergone transfer at the time, 28% underwent transfer resulting in a positive Beta hCG, 21% resulted in a negative Beta hCG, 2% experienced miscarriage, 5% had a biochemical pregnancy, and 0% had an ectopic pregnancy.

**Conclusion:** The results presented provide statistical evidence to reject the hypothesis of independence between nucleation and fragmentation grade variables in relation to PGT-A outcomes. Thus, increased fragmentation grade and nucleation abnormalities (binucleation or multinucleation) are strongly associated with an increased number of aneuploid embryos, corroborating to the literature. In the same way, nuclear normality and fragmentation grade I are strongly associated with an increased number of euploid embryos.

**P-49. The significance of oncofertility in adolescents and young adults of reproductive age**

**Mariane Cristina Carlucci Molina Felix^1^, Nilo Frantz^2^, Luciana Lopes Manfredini^3^**

*^1^ Faculdade Israelita de Ciencias da Saude Albert Einstein, Nilo Frantz Medicina Reprodutiva - São Paulo - SP - Brasil.*

*^2^ Nilo Frantz Medicina Reprodutiva - São Paulo - SP - Brasil.*

*^3^ Faculdade Israelita de Ciencias da Saude Albert Einstein - São Paulo - SP - Brasil.*

**Objective:** The aim of this review is to study about how oncofertility is approached in adolescents and young adults of reproductive age.

**Methods:** A review of the last five years' worth of literature was done. The search was performed in the Virtual Health Library (BVS) and Pubmed database with the descriptors "oncology", “fertility preservation”, “infertility." and “reproductive health”. Portuguese, English, and Spanish-language complete articles were taken into consideration.

**Results:** Fertility preservation is an emerging topic in oncology but is not widely discussed among adolescents and young adult survivors. When we consider that a portion of this patient population will experience future cancer remission and must contemplate having children in the future, it is crucial that they have the right to be informed about potential adverse effects of cancer treatment, such as hypogonadism and infertility; Considering the efficacy of both treatments, we need to refer the patient to a fertility specialist before the cancer treatment begins. There is a discrepancy between the patient's desire to preserve their reproductive capacity and the frequency with which they are provided with this information. This discussion will alleviate their anxiety and enhance their quality of life. It must be acknowledged that cancer and infertility are not explicitly linked, but treatment can have a negative impact in this area. Oncologists have limited experience with fertility preservation and have little time to elucidate the diagnosis and treatment of cancer, as well as their implications. Thus, it is necessary to have a fertility reference for patients who express interest or are unsure of their decision.

**Conclusion:** This literature review would appear to demonstrate that patients need and desire information about the effects of cancer treatment on their fertility. In cancer survivors, we can see the significance of this theme. Some gaps need to be filled so that we can provide the right information at the right moment about all of the available fertility treatment options and so that the quality of life of cancer patients can be assured upon completion of cancer treatment.

**P-50. Outcome of pregnancy rate according to oocyte donor BMI**

**Kahisa Natiele Fontana Dal Toso^1^, Camila Rambo^2,^ Priscila Cristina Almeida^1^, Regis Yukio Cho^1^, Lidio Jair Ribas Centa^1^, Thaina Carlesso Setoyama^2^**

*^1^ Androlab Clinica da Fertilidade - Curitiba - PR - Brasil.*

*^2^ CHC-UFPR - Curitiba - PR - Brasil.*

**Objective:** Suboptimal reproductive outcomes have been associated with increased Body Mass Index (BMI), particularly in assisted reproductive technology (ART). Studies have demonstrated that women who are overweight and obese have lower rates of fertilization, implantation, and pregnancy. The main objective of the present study, therefore, was to assess the outcome of pregnancy rate according to BMI. Knowing that the quality of eggs reduces with advancing age, only oocytes from donor patients were included in this study.

**Methods:** Retrospective study performed at the ART center Androlab, located in Curitiba, Brazil, with oocytes from donors in the period between January/2021 and June/2023. All patients who transferred embryos originated from a donated oocyte were included in the study. Patients were separated into four groups according to the donor's BMI and the outcomes were analyzed considering positive, negative, and biochemistry results.

**Results:** The analyzes included a total of 56 donated oocytes. Being 23 oocytes were from donors classified as eutrophic, with a BMI under 24.9. The recipients of this group had an implantation rate of 61% and a pregnancy rate of 65%. Nineteen oocytes were from overweight donors, with a BMI between 25 and 29.9, and reached an implantation rate of 47% and a pregnancy rate of 63%. Eleven oocytes were from donors classified as class I obese, with a BMI between 30 and 34.9, and the recipients had an implantation rate of 27% and a pregnancy rate of 36%. Finally, three oocytes were from donors with a BMI greater than 35, and the recipient had an implantation and a pregnancy rate of 33%.

**Conclusion:** Despite the difficulty in finding donor candidates, it is interesting to assess the BMI when selecting the patient for donation. Limitations include the retrospective design, sample size, data from a single institution and the combination of the donor oocyte´s BMI and the BMI of the patient who received the embryo. The observations of this study suggest a possible effect of BMI at the oocyte level, before fertilization and implantation, and the need for further investigation. Having a multi-professional team, including a nutritionist, is an important point for the comprehensive treatment of the patient. Considering the results, patients with an increased BMI should be instructed to practice physical activities and regulate their diet before the oocyte donation.

**P-51. Decoding the Microbial Puzzle: The Seminal Microbiome and its Association with Male Infertility**

**Filipe Tenorio Lira Neto^1^, Marina Correia Viana^2^, Sandro Cassiano Esteves^2^**

*^1^ Andros RECIFE - Recife - PE - Brasil.*

*^2^ Androfert - Campinas - SP - Brasil.*

**Objective:** To summarize the current evidence regarding the seminal microbiome and its association with male infertility and suggest topics that should be addressed by future research to fulfill the existing knowledge gaps.

**Methods:** We performed a narrative review including all studies published regarding the male tract microbiome in humans published from January 1990 to January 2023. We reviewed studies applying culture-based, polymerase chain reaction (PCR)-based, and next-generation sequencing (NGS)-based approaches to evaluate the seminal microbiome. We extracted data concerning the type of sample (semen or testicular tissue), study design, participants’ characteristics, techniques used, and main findings.

**Results:** We included 34 studies, comprising 2326 participants. Sixteen studies used culture-based methods, 14 used NGS, and four applied multiple methods to identify microorganisms. No study assessed fungi or viruses. All NGS-based studies identified the presence of bacteria in all semen samples evaluated. Two distinct features of the seminal microbiome were the wide variation of species composition among men and the clustering in microbial communities with a predominant species. The most common genera identified were Lactobacillus, Pseudomonas, Gardnerella, Prevotella, Streptococcus, and Staphylococcus. The genera Lactobacillus was associated with normal semen analysis parameters and asthenozoospermia, whereas the genera Prevotella was consistently linked to poor semen quality. Studies evaluating the testicular microbiome demonstrated that the testicular compartment is not sterile. The phyla Actinobacteria, Bacteroidetes, Firmicutes, and Proteobacteria were the most frequent in testicular samples. In these lines, despite rigorous antiseptic and aseptic precautions, contamination was responsible for 50-70% of all the detected bacterial reads, suggesting that sperm retrieval procedures are not performed under sterile conditions. The relationship between seminal and vaginal microbiomes has also been investigated. Couples shared 56% of predominant genera, and 61% of the couples with positive cultures in both partners shared at least one genital pathogen. Accordingly, in couples with explained infertility, there was an overlap between the bacterial composition of the seminal and vaginal microbiomes, including increased prevalence of Staphylococcus and Streptococcus genera. The seminal microbiome also impacted reproductive outcomes. Higher counts of Alphaproteobacteria and Gammaproteobacteria in washed sperm and Corynebacterium sp. in raw semen samples were associated with lower embryo quality. In contrast, the mean proportion of the Enterobacteriaceae group in raw semen was higher in couples with improved embryo quality. In addition, bacterial reads were found in the IVF media in 8% of the samples by NGS and in more than 70% by real-time PCR method, with Lactobacillus and Phyllocterium being the most frequent genera. However, bacteria in IVF culture media did not influence the pregnancy rate.

**Conclusion:** The current literature supports the notion that several genera of bacteria colonize the entirety of the male reproductive tract. These organisms are not living independently. Indeed, they are essential in regulating functions and marinating the hemostasis. Dysbiosis, alterations in the seminal microbiota such as an imbalance in the composition of the microbial community, which includes loss of symbionts, outgrowth of pathobionts or opportunists, and disturbed inter-microbial competition and microbial diversity, may be implicated in the development or worsening of several male infertility conditions. The future directions in understanding the seminal microbiome and its associations with male infertility and semen alterations encompass several crucial areas of investigation. Standardizing protocols, identifying informative variable regions, applying shotgun metagenomics, and addressing contamination concerns are necessary to ensure research findings' reliability and comparability. Longitudinal and prospective studies and investigations into the impacts of infertility causes and commonly prescribed drugs will further our understanding of the seminal microbiota's role in reproductive health.

**P-52. Prospective performance assessment of an artificial intelligence software for embryo selection and clinical pregnancy prediction**

**Mariana Nicolielo Barreto^1^, Renata Erberelli^1^, Bruna Lourenço^1^, José Roberto Alegretti^1^, Eduardo Leme Alves Motta^1^, Mauricio Barbour Chehin^1^, Dóris Spinosa Chéles^1^, Marcelo Fábio Gouveia Nogueira^2^, José Celso Rocha^2^, Aline Rodrigues Lorenzon^1^**

*^1^ Huntington Medicina Reprodutiva - Eugin Group - São Paulo - SP - Brasil.*

*^2^ UNESP - ASSIS - SP - Brasil.*

**Objective:** New technologies are emerging to assist embryologists on selecting the best embryo for transfer. Shortening time to pregnancy and preventing extra emotional distress on ART patients is a crucial step in any IVF treatment. The development of artificial intelligence (AI) algorithms based on machine learning methods to increase reproductive medicine outcomes is a new frontier worldwide. The use of time-lapse technology incubators provides new data regarding embryo morphokinetics and morphological details that were unnoticed. The aim of this study is report the performance of an AI software developed in our center for embryo selection and clinical pregnancy prediction.

**Methods:** Prospective cohort study including patients undergoing a single embryo transfer (sET, blastocyst stage) after an in vitro fertilization (IVF) treatment between June/2022 and June/2023 (n=230 patients). Embryo selection and clinical pregnancy (presence of gestational sac and heartbeat) prediction software was previously built through artificial neural networks technique and genetic algorithms using the input of morphological data of 1.000 blastocyst with known reproductive outcomes. Blastocyst digital image processing were analyzed considering 33 mathematic variables. All embryos were cultured in a time-lapse incubator (Embryoscope Plus, Vitrolife). Input data were randomized for training, validation and test (70, 15 and 15%, respectively). Software performance in the validation test achieved an area under curve of 0,62 for positive clinical pregnancy (CP) and 0,52 for negative CP. Prediction rates were assessed in elective (2 or more embryos available to transfer, n=148 patients) and non-elective (1 available embryo to transfer, n=82 patients) sET, prospectively analyzed in the software, which classified the embryos accordingly to its chance (%) for clinical pregnancy. Embryos were also analyzed by embryologists according to morphological and morphokinetics features.

**Results:** In 58.1% of the patients from the elective sET group (n=86/148), the top embryo choice for transfer was concordant between software and embryologists. In this group, CP rate was 62.8%, with a software performance for positive CP of 96.3% (the software may predict none of the embryos in a cohort would achieve a CP). Overall, the performance of the software in cohort (positive + negative CP prediction) was 64%. In non-concordant cases for top embryo choice for elective transfers (n=62, 41.9%), the embryo chosen by the embryologist was transferred and CP rate was 64.8%. The software performance for negative CP was 83.9%. In non-elective sET, CP was achieved by 47.6% of the cohort. Software performance for negative CP was 72.1% and overall positive and negative prediction was 62.2%.

**Conclusion:** Our software was able to predict 64% of CP outcomes for concordant elective sET and 62.2% for non-elective sET, similar to the performance of validation test (62%). Proper validation of new AI tools for embryo selection and clinical pregnancy prediction is crucial for potentially use in the IVF laboratory. Currently, the software may be used as a support tool for embryologist’s decision.

**P-53. Association with the use of gonadotropins in the treatment of infertility in men with Kallmann Syndrome: a report of 2 cases**

**Lívia Santos Souza^1^, Danilo Modafaris Araujo^1^, Tássia Souza Leão Silva^1^, Bianca Catapano Aragão^1^, Janaina Jardelha Mendes Maciel^1^, Joaquim Roberto Costa Lopes^1^**

*^1^ Cenafert - Salvador - BA - Brasil.*

**Objective:** To evaluate the sperm production response in 2 (two) azoospermic patients diagnosed with Kalmann Syndrome (KS) and treated at the Human Reproduction Center - Cenafert.

**Methods:** Patient 1 (first), 35 years old, started the protocol with human menopausal gonadotropin (HMG) 75 IU daily and, after 60 days, added human chorionic gonadotropin (HCG) 5,000 IU every 5 days. Then, showed a response after 5 months of using the medications, with rare motile spermatozoa in the sample. However, the patient became azoospermic again, being diagnosed with varicocele, that has done a surgery for correction. There was no interruption of the hormonal protocol and at 6 months sperm production returned. An in vitro fertilization (IVF) cycle was performed using fresh and freeze ejaculated semen from all the embryos: 6 inseminated oocytes, 4 frozen embryos. Two months after the first oocyte collection, a second IVF cycle was performed with fresh ejaculated semen: 6 inseminated oocytes, 4 frozen embryos. Performed a transfer of 2 frozen embryos, twin pregnancy and babies at home.

Patient 2 (second), 34 years old, used HMG 75 IU daily for 60 days, then added HCG 5,000 IU every 5 days. One year after the beginning of the hormonal protocol, a spermogram was performed at the clinic and the presence of rare motile spermatozoa was observed and an IVF cycle was performed: 16 oocytes were inseminated, 7 blastocysts were frozen and medication was discontinued. Three transfers of frozen embryos were performed, resulting in a pregnancy that progressed to abortion. Two years later, the patient restarted the same gonadotropin protocol for 80 days, and rare motile spermatozoa were observed, which were used in the second cycle of IVF: 14 oocytes were inseminated, 5 blastocysts were frozen after trophectoderm biopsy, and cellules were sent for preimplantation genetic testing for aneuploidy (PGT-a), resulting in 3 euploid embryos with a single transfer and ongoing clinical pregnancy.

**Results:** The two patients treated with the hormonal protocol of gonadotropins were successful in the production of spermatozoa; and submitted to IVF techniques they achieved pregnancy.

**Conclusion:** The protocol using gonadotropins HMG and HCG was effective in the treatment of male infertility caused by KS in both patients in this study, but more controlled studies with a representative sample are needed to confirm this protocol and determine the safety of this treatment.

**P-54. Perinatal impacts related to assisted reproduction techniques**

**Antônio Carvalho Azevedo^1^, José Welligton de Oliveira Santos Júnior^1^, Leonardo de Oliveira^1^, Matheus Barbosa Souza^1^, Alexandre Machado de Andrade^1^**

*^1^ Fundação Universidade Federal de Sergipe - São Cristóvão - SE - Brasil.*

**Objective:** This literature review seeks to examine the perinatal ramifications associated with Assisted Reproduction Techniques (ART).

**Methods:** The research utilized the Boolean Operators '(Assisted Reproductive Techniques) AND (Child Development OR Health Risks OR Related Health Problems OR Child Health)' to conduct a comprehensive search. Databases such as U.S. National Library of Medicine (PUBMED), Biblioteca Virtural em Saúde (BVS) and Cochrane were employed. The initial search included filters for free articles and publications between 2018 and 2023. In the PUBMED database, strict selection criteria focused on clinical trials, meta-analyses, randomized clinical studies, and systematic reviews, yielding 203 articles. Conversely, the BVS and Cochrane databases allowed unrestricted study types, generating 191 and 46 articles, respectively. After meticulous review and exclusion of irrelevant and duplicate studies, 49 articles remained. The final selection encompassed 10 articles, chosen based on robust evidence and minimal methodological limitations.

**Results:** The body of research on children conceived through Assisted Reproduction Techniques (ART) suggests a higher susceptibility to unfavorable perinatal outcomes when compared to natural conceptions. These outcomes include twinning, imprinting disorders, gestational complications, and cancer, with the probability of these risks varying based on the specific ART technique utilized. A systematic review revealed a 43% higher risk of congenital defects in children conceived through ART compared to those conceived naturally. Notably, prematurity and very low birth weight emerged as the most significant perinatal risks associated with ART conceptions. Consistent with these findings, a Canadian study also reported substantially higher risks for adverse perinatal outcomes linked to ART, such as twinning, cesarean delivery, and prematurity. Moreover, children conceived through ART displayed a significantly higher association with complex chronic conditions at one year of age compared to those from spontaneous pregnancies. Furthermore, studies analyzing the correlation between congenital defects and the incidence of childhood cancer found that ART conception increased the risks of non-chromosomal congenital defects. Additionally, these defects appeared to heighten the baseline risk of developing cancer in affected individuals. The increase in the rate of monozygotic and monochorionic twinning was also linked to ART in a meta-analysis and systematic review, particularly when embryo transfer occurred at the cleavage stage and in women under 35 years old. When it comes to imprinting disorders, research indicated that the risk was significantly higher in children conceived through ART, with Neonatal Diabetes Mellitus (NDM) being the most associated condition. Nonetheless, the authors of these studies highlighted the potential influence of various factors such as the type of female infertility, mode of conception, obesity, and diabetes, which could have affected the observed results. On the other hand, a Danish study suggested that existing results on the risk of imprinting disorders were ambiguous and that such an association might be limited to specific cases, such as Beckwith-Wiedemann Syndrome. Overall, these findings underscore the need for continued research to better understand the potential risks and implications associated with ART-conceived pregnancies, considering various contributing factors and the specific techniques employed.

**Conclusion:** Children conceived through Assisted Reproduction Techniques (ART) exhibit higher risks of adverse perinatal outcomes and congenital defects compared to spontaneous conceptions. ART methods vary in their specific risks. However, some studies stress the importance of considering confounding factors like twinning, parental age, comorbidities, and subfertility when evaluating these findings. Epigenetic alterations, notably imprinting disorders, may be induced by ART, as suggested by the literature. Fortunately, the risks associated with ART have been progressively declining over time due to advancements and safer technique selection.

**P-55. The use of LH levels on the day of hCG administration as a predictor of pregnancy in Frozen embryo transfer (FET) - a pilot study of quality improvement activity**

**Carlos Alberto Link^1^, Rafaela Amaro Link^1^, Ricardo Francalacci Savaris^2^, Noeli Cecília Sartori^1^, Carolina Giordani Andreoli^1^, Andreia Moro Tore^1^, Mariana SaikoskI Faller^1^, Betina Iser^1^, Renata Pedo^1^**

*^1^ Clínica ProSer - Porto Alegre - RS - Brasil.*

*^2^ UFRGS - Universidade Federal do Rio Grande do Sul - Porto Alegre - RS - Brasil.*

**Objective:** To verify the mean values and standard deviation of serum luteinizing hormone (LH) on the day of hCG administration in FET cycles (natural cycle modified) for sample size calculation.

**Methods:** In this historical cohort, patients who underwent ovulation control for embryo transfer, between February and June 2023 at Clínica Proser - Porto Alegre, RS, Brazil - had their serum LH (U/l) measured on the day of human chorionic gonadotropin hCG injection according to local protocol; 7 weeks after embryo transfer, a transvaginal ultrasound was performed to verify the presence of an intrauterine pregnancy. The main outcome was the presence of an intrauterine gestational sac. Mean levels were compared between groups and the area under the curve was analyzed.

**Results:** The mean(±SD) levels of LH were 19.7±16.7 and 13.68±10.4 in women who got pregnant and not, respectively (*p*=0.2 Student t-test with Welch's correction). The area under the curve was 0.5981 (95%CI=0.398 to 0.797; *p*=0.3). The power of the study was 25%.

**Conclusion:** A minimum of 240 patients are required to have a 90% chance of detecting, as significant at the 5% level, a decrease in serum LH from 20 U/I in the pregnant group to 13 U/I in the non-pregnant group, based on this pilot study. The area under the curve found herein is similar to the one published in the literature.

**P-56. Sperm capacitation by swim-up, are we really selecting the best spermatozoa?**

**João Paolo Bilibio^1^, Lucia Bevilacqua^1^, Arivaldo Meireles^2^, Paulo Marcelo Silveira^2^, Vanessa Cristina Freitas Moreno^1^, Fabio Nascimento^2^**

*^1^ FertiBC- Centro de Reprodução Humana - Balneário Camboriú - SC - Brasil.*

*^2^ Pronatus- Centro de Reprodução Humana - Belém - PA - Brasil.*

**Objective:** In infertile couples, sperm capacitation is used as a complement to the spermogram evaluation and especially in seminal samples that will be submitted to In-Vitro Fertilization (IVF). Despite the effectiveness related to the selection of motile spermatozoa, there are few studies in the literature that demonstrate the impact of swim-up on the improvement of morphology. This fact impairs the evaluation of the real efficiency of this method, which can result in samples with good motility, but of low quality, since, although mobile, these cells can present important morphological alterations, capable of negatively influencing IVF success rates. For this reason, the aim of this study was to evaluate the impact of sperm capacitation (swim-up) on the seminal parameters of samples of infertile couples with teratozoospermia, through pre and post swim-up analysis.

**Methods:** A prospective cohort study was performed that included 71 seminal samples from infertile couples with the following criteria: age > 18 years, presence of teratozoospermia (Kruger <4%), sperm concentration >5x106/ml and vitality >58%. The study was approved by the Research Ethics Committee under CAAE 39759020.1.0000.0018. Patients who met the inclusion criteria underwent seminal collection and evaluation of seminal characteristics shortly after liquefaction. Subsequently, seminal processing (swim-up) and a new reassessment of concentration, motility and morphology were performed. Categorical variables were compared with Chi-square, continuous using t-tests and associations through logistic regression. Differences between groups were considered significant when *p*<0.05.

**Results:** Mean age was 37.4 years (± 6.4) and abstinence time was 5.5 days (± 0.9). The analysis after swim-up showed that despite the reduction in total sperm concentration (74.5 x 106 versus 27.8 x 106 per ml, *p* < 0.001), there was progressive motility increase (46.2% versus 94.8%, *p* < 0.001). There was a small increase in the mean percentage in the rate of normal spermatozoa (1.4% versus 2.2%, *p* < 0.001), despite this, morphology indices still remained abnormal in 86% of patients (below 4%).

**Conclusion:** We observed a slight increase in the rate of morphologically normal spermatozoa after sperm capacitation, however, there was no normalization of the morphology index after the swim-up - which remained below 4% in 86% of individuals suggesting that there is little benefit of the method in morphological selection. Thus, the increase in the percentage of progressive motile spermatozoa does not translate into a significant gain in the quality of the samples, because the morphological abnormality was maintained in most patients and it is known that abnormal morphology is associated with worse embryonic development and worse results in IVF.

**P-57. The relevance of ethnicity in social fertility preservation: should it be included as a variable while counseling our patients?**

**Thais Sanches Domingues^1^, Ana Paula Aquino^1^, Patricia Leme^1^, Renata Fioravanti Schaal^1^, Talita Devecchi^1^, Aline Rodrigues Lorenzon^1^**

*^1^ Huntington Medicina Reprodutiva - Eugin Group - São Paulo - SP - Brasil.*

**Objective:** There is a growing demand on oocyte cryopreservation for non-medical reasons as a tool to mitigate age-related fertility decline. The ideal number of frozen oocytes to reach a good future prognostic is estimated around 10 to 20, depending on women’s age, in infertile patients. Still, there is a lack of knowledge on the ideal number in social preservation and how other variables, besides age, would affect the treatment planning for this population. There are growing evidence that ethnicity may be related to ovarian response in controlled ovarian hyperstimulation. The aim of this study was to assess the number of mature oocytes retrieved from donors considering their ethnicity.

**Methods:** Large observational cohort study from 1757 egg retrievals of 820 oocyte donors, performed between April/2013 and December/2022 in a single ART center. Oocyte donors are young women (between 18 and 35 years old) with regular menstrual cycles, no historic of genetically and sexual transmitted diseases, with antral follicle count (AFC) ≥12. Donors were treated with a conventional starting gonadotrophin dose of 150-225 IU recombinant FSH (rFSH) in a fixed GnRH antagonist protocol. They were categorized in four groups according to their ethnicity: A - Latin american (n = 587), B - Caucasian white (n = 109), C - African American (n = 88) and D - Asian (n = 36). Kruskal-Wallis with Dunn’s multiple comparisons test was applied for statistical analysis. *p*<0,05 was considered significant.

**Results:** Asian donors were slightly older (27.4±3.68 yo) than A (25.1±3.91), B (25.7±4.13) and C (24.2±3.26, *p*<0.001). White Caucasian showed lower body mass index (BMI) in comparison to D (23.37±3.28 vs 24.8±2.43, *p*=0.01). Asians received the highest recombinant FSH dosage (total rFSH) during controlled ovarian hyperstimulation (2917.01±694.03) in comparison to A (2481.08±692.30), B (2487.77±780.38) and C (2537,18±618,55, *p*<0.001). Asians also showed the lowest AFC in comparison to groups A, B and C (16.30±4.49 vs 20.45±9.32, 19.52±7.09, 20.35±6.40, *p*<0.01, respectively) and the lowest number of oocytes retrieved (21.54±7.45 vs 25.91±12.07, 24.00±10.49, 26.12±9.55, p=0.02, respectively). White Caucasians showed less mature oocytes in comparison to African Americans (17.11±8.30 vs 19.11±7.59, *p*=0.01). When analyzing the percentage of mature oocytes per number of oocytes retrieved, Asians showed highest %MII (76%±13.3%) in comparison to A (71.2%±15.2%) and B (68.5%±15.5%, *p*<0.001). White Caucasians (B) also showed lower %MII in comparison to A and C (73.9%±14.3%, *p*<0.001). In despite of showing the highest age, BMI and total rFSH and lowest AFC and number of oocytes retrieved, Asians showed the highest %MII in our cohort while White Caucasians the lower %MII.

**Conclusion:** White Caucasians showed lower %MII in comparison to other ethnicities. This may have an impact on the number of oocytes available for cryopreservation and reinforce the importance to consider other variables in the moment of patient’s counseling and treatment planning.

**P-58. Dydrogesterone versus GnRH Antagonist: Comparative study in normal responders submitted to ovarian stimulation in IVF cycles**

**Fulvia Estefania Padre e Fechine^1^, Mariana Santos Costa^1^, Edlla Mikaine Padre e Fechine^1^, Patricia Tourinho da Silva^1^, Raissa Ramos Coelho^1^, Waleska Sharron Padre e Fechine^1^**

*^1^ Fertvida São Luis - São Luís - MA - Brasil.*

**Objective:** Ovarian stimulation is a fundamental part of assisted human reproduction treatment cause it enables multiple recruitment and follicular maturation to capture oocytes. Currently, assisted reproduction clinics can rely on several protocols to promote the best result for the patient. Drugs that act as GnRH agonists or antagonists are normally used, but another ovarian stimulation protocol is through the use of progesterone. The objective of this work is to analyze the results of ICSI cycles performed in our unit, to compare the results between the use of GnRH antagonists and the use of dydrogesterone to stimulate ovulation in normal responders.

**Methods:** This research is a retrospective study involving 183 ICSI cycles performed at Clinica Fertvida - São Luis, between the years 2016-2021. The study groups were divided into 76 patients treated with GnRH antagonist in a short protocol and the second group has 107 patients treated with Dydrogesterone. All patients aged less than 38 years and had a normal hormonal profile. The patients participating in the study didn’t have endometriosis, endometrioma or polycystic ovaries. Data were analyzed by Software R 4.3.1, using the Kruskal-Wallis test with post-hoc in Duun.

**Results:** No significant difference was observed between the two groups regarding the number of oocytes collected and maturation. A significant difference was seen between the final amount of blastocysts, being greater in the dydrogesterone group

**Conclusion:** It was observed that the use of progesterone was effective for ovarian stimulation, as well as the use of antagonists. This result is important to assist in our clinical practice.

**P-59. Has the COVID-19 pandemic affected mental health in the same way in infertile and fertile patients?**

**João Paolo Bilibio^1^, Pânila Longhi Lorenzzoni^2^, Arivaldo Meireles^3^, Lígia König^4^, Maria Eduarda Branco^4^, Joao Sabino Cunha-Filho^5^**

*^1^ FertiBC - centro de Reprodução Humana - Balneário Camboriú - SC - Brasil.*

*^2^ Programa de Pós-Graduação em Medicina: Ciências Médicas, Universidade Federal do Rio Grande do Sul - Porto Alegre - RS - Brasil.*

*^3^ Pronatus - Centro de Reprodução Humana - Belém - PA - Brasil.*

*^4^ UNIFEBE - Faculdade de Medicina - Brusque - SC - Brasil.*

*^5^ Insemine - Centro de Reprodução Humana - Porto Alegre - RS - Brasil.*

**Objective:** The COVID-19 pandemic has affected the mental health of many people around the world, and for infertile women, the challenges have been even greater. The pandemic has led to a postponement of fertility treatments and this may have exacerbated the levels of stress, anxiety and depression in these women. Therefore, the objective of this study was to analyze the impact of the COVID-19 pandemic on the levels of anxiety, depression, stress of infertile and fertile women.

**Methods:** Prospective cohort study that evaluated the rates of anxiety, depression and stress using the DASS scale in two moments, at the beginning of the COVID19 pandemic (Phase 1), when infertile patients had to suspend their treatments and after 6 months of the pandemic (Phase 2). The study group comprised of infertile women (Infertile group, N=37) and the control group comprised of fertile women (fertile group, N=33).

Inclusion criteria: - Infertile group: women with more than one year of infertility, with indication of In-Vitro fertilization, and who were going to transfer their embryos and had to suspend the transfer due to the pandemic.

- Control group: fertile women who gave birth more than 6 months ago and never had difficulty in becoming pregnant.

The study was approved by the National Research Ethics Committee under CAAE number 32964620.3.1001.5334.

**Results:** Mean age was higher in the infertile group (35.5 vs 37.8, *P*=0.001). In phase 1 of the study, comparing the fertile group vs. the infertile group, we found: higher levels of stress (5.6 vs 7.7, *P*=0.018) and depression (2.3 vs 4.0, *P*=0.002) in the infertile group, and similar rates of anxiety (2.7 vs 3.4, *P*=0.389). In phase 2 of the study, comparing the fertile group vs. the infertile group, we found: levels of stress (3.1 vs 3.7, *P*=0.201), depression (3.5 vs 4.0, *P*=0.446), and anxiety (5.4 vs 5.8, *P*=0.666).

Comparing the two phases, we found a significant decrease in stress (*P*=0.027), similar levels of anxiety (*P*=0.101) and worsening of depression levels (*P*=0.006)

**Conclusion:** At the beginning of the pandemic, when assisted reproduction treatments were suspended, infertile patients were more stressed and depressed than the control group, probably due to the delay in treatment and the difficulty in getting pregnant. After 6 months, there was a decrease in stress, possibly due to the lockdown, with removal from the day-to-day rush. On the other hand, there was an increase in the levels of anxiety and depression, possibly because it is a period associated with the fear of losing a loved one.

**P-60. Two DuoStim, two healthy babies case report of a rare non-classic congenital adrenal hyperplasia patient after preimplantation genetic test during in vitro fertilization**

**Melissa Cavagnoli^1^, Amanda Volpato Alavarez^1^, Maria Augusta Tamm^1^, Vickie White Loureiro Souza^1^, Rafael Favero Ambar^1^, Maite Del Collado^2^**

*^1^ Fertility Medical Group / Instituto Sapientiae - São Paulo - SP - Brasil.*

*^2^ Fertility Medical Group - São Paulo - SP - Brasil.*

**Objective:** Non-classic congenital adrenal hyperplasia (NCAH) due to 21-hydroxilase deficiency is an autosomal recessive disorder. NCAH patients typically have 21-hidroxylase (21-OH) deficiency associated with CYP21A2 mutations. Over 200 CYP21A2 mutations have been reported, and approximately 10 of them account for the majority of affected alleles. Here, we describe a case report of two successful pregnancies of a NCAH patient presenting with one heterozygous deletion of the gene CYP21A2 plus a rare substitution in the promoter region (-102 G>A), and a partner who also has one heterozygote mutation in the same gene (Q318X).

**Methods:** She (31 years old) has NCAH confirmed by genetic testing with the diagnosis of a heterozygous deletion in the gene CYP21A2 plus a rare substitution in the promoter region, -102 G>A. Her husband (32 years old) was also tested and a heterozygous mutation (Q318X) was detected. After a year of infertility, the couple underwent two IVF treatments (DuoStim cycles) and Preimplantation Genetic Testing for Monogenic Diseases (PGT-M) to achieve two pregnancies. A direct diagnosis of Q318X mutation and an indirect diagnosis using informative Small Tandem Repeats (STRs) were optimized for the couple. Two independent diagnoses were applied to each embryo during PTG-M. The causative mutation detection was performed by Polymerase Chain Reaction (PCR), and the mutant site was detected using the MiniSequencing Reaction. A total of six STRs (RING3, TAP1, D6S1666, TNFa, D6S265 and D6S1683) were found surrounding the gene CYP21A2 and used as indirect diagnosis. The STRs were chosen if contained a tetranucletide repetition core, linked to the gene (upstream or downstream) and having the highest heterozygosity value. Genomic DNA was extracted from peripheral blood from the couple and both sets of parents. The STRs were classified as being informative (both couple heterozygous), partially informative (one of the couple homozygous and the other heterozygous) or not informative (both homozygous). After embryo genetic test by PGT-M, artificial endometrial preparation was performed for the transfer of frozen-thawed embryos.

**Results:** From the first DuoStim cycle, six top-quality embryos were biopsied and frozen. Four embryos were identified as carrier of two mutations (maternal and paternal), one embryo was carrier of only one maternal mutation, and one was carrier of both maternal mutations and the wild paternal allele. The combined PGT-A study of the embryos revealed four euploids, being two males and two females, one mosaic aneuploid and one triploid. A pregnancy was achieved after the transfer of two euploid male carrier embryos. For the second pregnancy, the couple was subjected to another DuoStim cycle resulting in eight top-quality embryos, which were genetically analyzed. Three embryos were carriers of a maternal and a paternal mutation, four were identified as carrier of only a maternal mutation and one embryo was non informative. From the four embryos carriers of a maternal mutation, three were euploid. After an implantation failure of a single carrier euploid embryo, the couple got pregnant of a second baby after a transfer of two carrier euploid embryos. Both babies were born healthy after an IVF PGT-M treatment.

**Conclusion:** The association of genetic counseling and assisted reproductive technologies with the use of preimplantation diagnosis can help couples to have healthy babies in a rare NCAH cases.

**P-61. Association of thrombophilic and immunological factors with recurrent pregnancy loss**

**João Paolo Bilibio^1^, Lucia Bevilacqua^1^, Arivaldo Meireles^2^, Gabriel Enrico Andrigheto^3^, Jose Armando Boos Vásquez^3^, Letícia Mocellin^3^**

*^1^ FertiBC - Centro de Reprodução Humana - Balneário Camboriú - SC - Brasil.*

*^2^ Pronatus - Centro de Reprodução Humana - Belem - PA - Brasil.*

*^3^ UNIFEBE- Faculdade de Medicina - Brusque - SC - Brasil.*

**Objective:** Recurrent pregnancy loss (RPL) is defined as the loss of two or more consecutive pregnancies. The possible causes of RPL can be genetic, thrombophilic, immunological, among others, however, in approximately 30% the cause is unknown. Therefore, the objective of this study was to evaluate the association of thrombophilic (acquired and hereditary) and immunological (autoimmune and alloimmune) factors with recurrent pregnancy loss.

**Methods:** Case-control study, paired 1 control to 4 cases. It included 53 women with recurrent pregnancy loss in whom genetic analysis was performed on the abortion material, being the Aneuploid Group (control, n= 10) and the Euploid Group (study, n= 43). Study approved by the Ethics and Research Committee under protocol CAAE 79524317.1.0000.0018. Exclusion criteria: presence of known factors for RPL. A panel of acquired thrombophilia (IgG and IgM anticardiolipin, lupus anticoagulant), hereditary (protein C and S, prothrombin mutation, factor V Leiden, MTHFR 677 and 1298, antithrombin III, homocysteine), autoimmune immunological (ANA, anti DNA, antithyroglobulin, TSH, TRAB) and alloimmune (NK cell activity, CH50 and C3).

Categorical variables compared with Chi-square or Fisher, continuous with t-test and associations with logistic regression, significant differences when *p*<0.05.

**Results:** We found no differences between the Aneuploid Group versus the Euploid Group in age (35.6 vs. 36.0 years, P=0.753), antral follicle count (13.4 vs. 15.3, P=0.646) and FSH on day 3 of the menstrual cycle (7.1 vs. 5.9, P=0.317). Comparing the autoimmune factors (aneuploid versus euploid group), we found an association: ANA (25.0% vs 12.0%), TSH and antithyroglobulin (40.0% vs 25.8%, P=0.603). Likewise in the comparison of alloimmune factors: NK cell activity, CH50 and C3. As for thrombophilic factors, we found no association in any evaluated parameter, whether acquired (IgG and IgM anticardiolipin, lupus anticoagulant), or hereditary (Protein C, Protein S, Prothrombin mutation, Factor V Leiden, MTHFR 677 and 1298, Antithrombin III, Homocysteine).

**Conclusion:** We compared whether recurrent pregnancy loss patients with embryonic euploidy could have present thrombophilic or immunological factors that could be associated with loss when compared with the group with RPL due to aneuploidy. We found no association of any factor, suggesting that patients with RPL even with an euploid embryo must have other causes, not those evaluated in this study.

**P-62. Analysis of embryo transfer catheter: the effect of mucus, blood and retained embryo in pregnancy rates**

**Luiza da Silva Rodrigues^1^, Lisiane Knob de Souza^1^, Bruna Campos Galgaro^1^, João Sabino Lahorgue Cunha Filho^1^**

*^1^ Insemine - Porto Alegre - RS - Brasil.*

**Objective:** To evaluate if embryo retention and the presence of blood or mucus in the embryo transfer catheter affects embryo transfer outcome.

**Methods:** Frozen embryo transfers performed in a single center between January 2022 and May 2023 were analyzed retrospectively. Only patients with good quality embryos were included in the study. At the time of embryo transfer, information about the presence of blood, mucus and embryo retention in the catheter set were recorded. Pregnancy outcome was assessed through a β-human chorionic gonadotropin (β-hcg) blood test 12 days after embryo transfer.

**Results:** One hundred and twelve patients were eligible for the study. In 51 cases (45.5%), the catheter was clean after transfer, whereas blood was detected in 34 (30.3%), mucus in 47 (42%) and embryo retention in 9 (8%). Forty eight patients had a positive β-hcg test, corresponding to 42.8%. Embryo retention did not impact pregnancy outcome [33.3% vs 43.6% (*p*=0.547)], neither the presence of blood [38.2% vs 44.8% (*p*=0.514)] or mucus [34% vs 49.2% (*p*=0.109)] in the catheter (presence and absence groups, respectively). Multivariable analysis also confirmed our results that blood, mucus and retained embryo did not affect pregnancy rates (*p*>0.05).

**Conclusion:** Our data indicates that embryo retention, blood or mucus in the embryo transfer catheter has no impact on pregnancy rates.

**P-63. Successful ultrasound guided extracorporeal mature oocyte cryopreservation after ovarian cancer**

**Maria Eduarda Bonavides Amaral^1^, Erika Vieira Souza Jordao^2^, Iris Cabral^3^, Bruno Ramalho Carvalho^4^, Leonardo Martins Campbell^2^**

*^1^ Genesis Centro de Assistência em Reprodução Humana e Hospital Sírio Libanês Brasília - Brasília - DF - Brasil.*

*^2^ Invideo Instituto de Cirurgia Minimamente Invasiva de Brasília e Hospital Sírio Libanês Brasília - Brasilia - DF - Brasil.*

*^3^ Genesis - Centro de Assistência em Reprodução Humana - Brasília - DF - Brasil.*

*^4^ Bruno Ramalho Reprodução Humana e Hospital Sírio Libanês Brasília - Brasília - DF - Brasil.*

**Objective:** Ovarian cancer is a challenge for fertility preservation because of the risks of tumoral cell spillage in a traditional transvaginal oocyte pick-up. The possibility of aspirating follicles directly from the ovarian specimen already excised is an option in this scenario.

**Methods:** Case report of successful ultrasound guided extracorporeal mature oocyte cryopreservation after ovarian cancer.

**Results:** A 23 year old woman with a past history of ovarian dysgerminoma treated conservatively with unilateral right oophorectomy presented with tumor recurrence in the remaining ovary. The patient underwent ovarian stimulation followed by laparotomy and left oophorectomy, scheduled accordingly to follicular development and predicted egg retrieval. Follicular aspiration was performed under direct ultrasound guidance in the operation room and follicular fluid was transported in a heated device to the IVF lab within a maximum ischemia time of 63 minutes. A total of 11 mature oocytes were vitrified and an additional number of 4 immature oocytes were submitted to in vitro maturation (IVM) and also vitrified.

**Conclusion:** Mature oocytes can be retrieved from an oophorectomy specimen after controlled ovarian hyperstimulation, reducing the risk of cancer cell spillage associated with the standard procedure.

**P-64. Surgery versus expectant management for endometriomas: what is the best before IVF? Systematic review with metanalysis**

**Priscilla Menezes Queiroga Fonseca^1^, Sofia Andrade de Oliveira^2^**

*^1^ UNEB - Salvador - BA - Brasil.*

*^2^ Cenafert - Salvador - BA - Brasil.*

**Objective:** Evaluate if resection of endometriomas by stripping or cystectomy increases pregnancy and live births rates. Secondary outcomes include cancellation rate, fertilization rate and number of oocytes retrieved.

Methods: It is a review systematic with meta-analysis, with the results guided according to the Guideline PRISMA. Data synthesis was tabulated in Microsoft Excel and statistical analysis by R Studio. Two reviewers performed the screening.

**Results:** Among 1518 articles were screened. Seven studies were included - a randomized clinical trial and six observational studies, mostly with moderate risk of bias. The odds ratio of pregnancy rate after surgery (compared with conservative treatment before IVF/ICSI) was 0.82 (95% CI 0.61 - 1.08, seven studies, I2 = 0% Tau 2 = 0, X 2 6 = 2.97), and for the live birth rate was 0.67 (95% CI 0.46 - 0.96, four studies, I 2 = 0, Tau 2 = 0, X 2 3 = 0.41). The average difference in the number of oocytes retrieved between the two subgroups was -1.03 (95% CI -1.27 - -0.78, six studies, I 2 = 50%, CI 0%-80%; Tau 2 < 0.0001, X^2^ 5 = 10.03).

**Conclusion:** The metanalisys showed no increase in the pregnancy rate after surgery and it showed a reduction in ovarian reserve and live birth rate. The number of oocytes retrieved and the live birth rate were higher in the conservative treatment. However, better quality studies are needed.

**P-65. Development of a comprehensive panel including the primary bacteria linked to endometritis**

**Délis Oliveira Ferreira^1^, Martha Giovanna Mâcedo Costa^1^, Raissa Santos Belarmino^1^, Danielle Barbosa Morais^1^, Yago Tomaz Vieira Silva^1^, Daniel Carlos Ferreira Lanza^1^**

*^1^ Federal University of Rio Grande do Norte-UFRN - NataL - RN - Brasil.*

**Objective:** This study aims to present a panel that includes the main genera of bacteria commonly associated with endometritis.

**Methods:** To characterize the microbiota associated with endometritis, we conducted a comprehensive search in two databases: PubMed and Web of Science, without restricting the language and year of publication. The search was performed using the following parameters: “endometritis women” OR “endometritis female” OR "pelvic inflammatory disease" AND bacteria* OR “uterine microbiome”. We excluded reviews, meta-analyses, book chapters, studies conducted in animal models, and publications that did not report original clinical data or did not mention "endometritis" in the title or abstract.

**Results:** The panel is based on an analysis of 39 studies published over the past 38 years. We have identified 31 bacterial genera, with the following five being the most frequently observed: Chlamydia and Ureaplasma with 11.03% each, Streptococcus and Mycoplasma with 9.56% each, and Enterococcus with 8.09%. Additionally, we discuss the etiological aspects of endometritis and the main techniques used for detecting these pathogens. Regarding its etiological aspects, we found that bacterial infection is the most prevalent cause of the disease, occurring as a result of predisposing factors such as invasive procedures like uterine curettage, cesarean section, or insertion of intrauterine devices (IUDs), preterm birth, premature rupture of membranes, postpartum infection, and miscarriage, among others. These events can facilitate the entry of pathogenic microorganisms into the uterus, resulting in an inflammatory response and subsequent development of endometritis. It is important to note that the etiology of endometritis may vary in different population groups and clinical contexts. The main techniques used for detecting these pathogens associated with endometritis were microbial culture, Polymerase Chain Reaction (PCR), and Next-Generation Sequencing, were microbial culture has been the most employed, followed by PCR, or a combination of both techniques. Considering the articles that composed this review, one study employed a sequencing approach, enabling a more comprehensive analysis of microbial diversity. This diversity of techniques has greatly expanded our understanding of the presence and identification of microorganisms associated with the pathophysiology of endometritis.

**Conclusion:** These findings serve as a foundation for further investigations of microorganisms related to endometritis, and such analyses will help to clarify the relationship between endometritis and the bacteria that cause it. This, in turn, will improve our understanding of the epidemiology of the condition and enhance the accuracy of its diagnosis and treatment recommendations.

**P-66. Methods of fertility preservation in female teenagers: A systematic review**

**Giuliana Potthoff Passos^1^, Juliana Fernandes Dutra^1^, Paula Vaccarezza Lopes^1^, Márcia Sacramento Cunha Machado^1^**

*^1^ Escola Bahiana de Medicina e Saúde Pública - Salvador - BA - Brasil.*

**Objective:** Main objective: Define which are the main methods for fertility preservation during the adolescence of young women. Secondary objective: Indicate what are the most frequent motivation for fertility preservation among female teenagers.

**Methods:** This systematic review followed the updated Preferred Reporting Items for Systematic Review (PRISMA) guidelines, with the aim of evaluating and identifying the most appropriate methods for fertility preservation in adolescent girls. A systematic search was conducted in the electronic databases of BVS/Lilacs, PubMed/Medline, SciELO and Cochrane, on June13th, 2023. The final syntax was obtained using the Boolean operators AND and OR for multiple combinations of the search terms: adolescent [MeSH terms], fertility preservation [MeSH terms] and Therapeutic Uses [MeSH Terms]. Articles were included following PICOS strategy, being considered for inclusion clinical trials, systematic reviews, case-control, and cohort studies that addressed the theme in the female population. Editorials, author’s opinions, books, and in vitro rehearsals were excluded. Articles written in English and Portuguese were included.

**Results:** 2918 articles were founded, of which 2904 were excluded. Of the 14 remaining studies, ovarian tissue cryopreservation was the most common recommendation for fertility preservation in this population, being the indicated method for 10 of these studies. This procedure is advantageous because it does not require additional hormonal stimulation and can be performed immediately without interfering with other treatments, such as oncological therapy. However, because the procedure is performed laparoscopically under general anesthesia, there are risks such as bleeding, infection, and reinsertion of malignant cells, that should be considered, especially, for patients with leukemia, lymphoma or neuroblastoma. The procedure is indicated for women who don’t have a sexual partner, don’t want to use a sperm donor, or have religious concerns about freezing embryos. It’s also a dispendious technique that requires long-term maintenance. Similarly, embryo cryopreservation was recommended by the studies, although, in the scenario of such young patients, the existence of a partner to perform in vitro fertilization is unlikely, making ovarian tissue cryopreservation more suitable for this age group. However, 9 of the selected articles discussed alternative methods for secondary options of treatment, such as hormonal suppression with a GnRH agonist, which in the scenario of cancer patients, reduces exposure to chemotherapy at the uterine and ovarian levels, thereby reducing gonadotoxicity. In vitro maturation, oophoropexy and other techniques for ovarian preservation were also mentioned. The most common motivation for adolescent fertility preservation, discussed in 12 articles, was its use in the treatment of oncologic diseases like, for instance, leukemia and ovarian cancer. Additionally, two other articles brought to light other distinct events in which fertility preservation is required. One of them dealt with transgender children and teenagers, while the other discussed primary and secondary hypogonadism.

**Conclusion:** Therefore, most of the articles analyzed point in the direction of ovarian cryopreservation as the best alternative for fertility preservation in adolescent females. However, other techniques were presented as viable alternatives, such as GnRH agonist (GnRH-a), gamete cryopreservation, in vitro maturation, and oophoropexy. Treatment of neoplasms has been the main driver for fertility preservation in adolescent females. However, further clinical trials on this topic are needed, as the available literature lacks significant, well-designed studies on fertility preservation in this age group.

**P-67. In vitro exposure of cannabidiol impairs expansion of cumulus cells during oocyte maturation: outcomes using bovine model**

**Caroline Schiavao Fernandes^1^, Sarah Gomes Nunes^2^, Alan Brunholi Giroto^1^, Bruno Carrino Suave^1^, Ana Caroline Silva Soares^2^, Anthony Cesár Souza Castilho^1^**

*^1^ UNOESTE - Presidente Prudente - SP - Brasil.*

*^2^ UNESP - Botucatu - SP - Brasil.*

**Objective:** Oocyte maturation is a crucial process involving complex molecular signaling pathways that significantly impact successful fertilization, embryonic development, and implantation in both humans and animals. Environmental factors such as lifestyle choices, dietary habits, and the use of legal or illegal drugs can easily influence these processes, leading to reduced oocyte, sperm, and embryo quality. Scientists in reproductive medicine have expressed interest in the potential role of cannabidiol (CBD), a cannabisderived compound known for its ability to modulate hormonal processes and reduce inflammation. Several studies have investigated the effects of CBD on different aspects of the reproductive process, aiming to understand its influence on oocyte quality and overall fertility. Some evidence suggests that CBD may interact with the endocannabinoid system, which plays a crucial role in regulating reproductive function. However, further research is required to fully elucidate the specific mechanisms by which CBD affects oocyte maturation and reproductive outcomes. This study aimed to explore the effects of CBD during the in vitro maturation of oocytes using cattle as a model to assess potential future implications.

**Methods:** Bovine ovaries were collected from the slaughterhouse and transported to the laboratory in a thermal container at 37°C in saline solution (0.9% NaCl). Antral follicles with diameters ranging from 3 to 8 mm were aspirated using a syringe and an 18G needle. Cumulus-oocyte complexes (COCs) were selected, and classified based on quality grade under a stereomicroscope, with only COCs grades I and II used for the study. The COCs were divided into four groups. The base maturation medium was supplemented with CBD, diluted in 0.05% DMSO, at concentrations of 0.1, 1, and 10 µM, depending on the experimental group. The control group was conducted without the addition of CBD but with 0.05% DMSO. The base maturation medium consisted of TCM199 with bicarbonate and Earle's salts, supplemented with recombinant follicle-stimulating hormone (0.1 IU/mL), sodium pyruvate (22 µg/mL), amikacin (75 µg/mL), and bovine serum albumin without fatty acids (BSA; 4 mg/mL). Four replicates were performed, each with 35 COCs. The COCs were matured at 38.5°C in a humid atmosphere for 22-24 hours. Subsequently, matured COCs were photographed to assess the expansion of cumulus cells: poorly expanded (score 0), partially expanded (score 1), and fully expanded (score 2). The meiotic progress was examined under a microscope to determine the meiotic stage of the oocytes (Hoeschst 33342).

**Results:** Overall, CBD did not significantly affect meiosis progression in bovine oocytes (*P* = 0.20). However, CBD did lead to a decrease in the number of oocytes with a score of 1 (*P*= 0.05) and 2 (P= 0.02) and resulted in a significant increase in COCs with poor expansion (score 0, *P*= 0.04).

**Conclusion:** In vitro exposure to CBD resulted in the blockade of cumulus cell expansion in bovine oocytes matured in vitro. While some findings show promise, the use of CBD in fertility treatments must be approached cautiously, as ongoing studies are needed to assess safety and long-term reproductive effects. A deeper understanding of the relationship between CBD and oocyte maturation could provide valuable insights to address fertility challenges as research progresses.This study was financially supported by PIBIC/UNESP, CAPES, and FAPESP.

**P-68. Association between genetic polymorphisms of the FSHR gene and infertility in women undergoing hormonal stimulation: a systematic review**

**Ana Júlia Siqueira Guimarães^1^, João Marlus Costa da Gama Filho^1^, Nathalia Maria Santos Alves^1^, Danilo Menezes de Melo^1^, Alice Caroline Alves da Silva^1^, João Victor Rocha de Almeida^1^, Bruno Lasmar Bueno Valadares^1^**

*^1^ Universidade Federal de Sergipe - Aracaju - SE - Brasil.*

**Objective:** To investigate, based on the existing literature, the genetic polymorphism of the follicle-stimulating hormone receptor (FSHR) gene and its association with infertility in women undergoing hormonal stimulation.

**Methods:** Using the PRISMA guidelines, a search was performed in the PubMed/Medline and BVS databases in June 2023, restricted to articles published between 2013 and 2023, in English. The DeCS/MeSH descriptors “FSH receptor”, “polymorphism” and “female infertility” were used, excluding systematic reviews. As inclusion criteria, this systematic review used all studies that: 1) mapped genetic polymorphisms of the gene for the Follicle-Stimulating Hormone Receptor (FSHR); 2) in infertile women due to any etiologies; 3) evaluated the patients according to their ovarian responses to different methods of hormonal stimulation; and 4) performed statistical correlation analysis between the gene present for FSHR and the ovarian response found for a given hormonal stimulation. Data from the articles were collected independently by the authors and extracted using an extraction table developed in the Google Docs tool.

**Results:** After searching the electronic databases, 95 published full articles were found. Of the total results, after applying the inclusion and exclusion criteria, as well as removing duplicates, 18 publications remained for analysis. The total population (N) found consisted of N = 7032 women evaluated according to the type of genetic polymorphism found for the FSHR gene, being 6111 infertile patients, who integrate the study groups, and 921 fertile women, who form the control groups. The most cited method of assisted reproduction was *in vitro* fertilization with intracytoplasmic sperm injection (IVF/ICSI) present in 8 articles, followed by conventional IVF, present in 5 articles. 6 articles do not specify whether conventional IVF or with ICSI was performed. Furthermore, in relation to ovarian stimulation, 5 articles commented on the adoption of a GnRH agonist protocol, 5 articles used the GnRH antagonist protocol, 10 studies pointed to the use of exogenous FSH, 3 articles cited the use of hMG and 1 article mentions the administration of clomiphene citrate. 6 articles do not cite the methods adopted at this stage of the process.Three polymorphisms for the FSHR gene were established in the total female population: 15 studies addressed the polymorphism at position c.2039G>A (rs6166:C>T, p.Ser680Asn), 9 articles studied the polymorphism at position c.919G>A (rs6165:C>T, p.Ala307Thr) and 5 served the polymorphism at position c. -29G>A (rs1394205). As for the statistical analysis, the allelic variations of the c.2039G>A gene (rs6166:C>T, p.Ser680Asn) were related as a potential infertility factor in 11 of the 15 articles (73.3%) that study it. The alleles of the c.919G>A gene (rs6165:C>T, p.Ala307Thr) were classified as a possible infertility factor in all 9 articles (100%) that analyzed it. The different alleles of the c. -29G>A (rs1394205) exciting statistical link to fertility in only 2 of 5 papers (40%) describing it. Overall, the presence of at least one genetic polymorphism was considered statistically relevant in 15 articles of the 18 studies (83%) that make up the present systematic review, while in 3 publications (17%) it was considered statistically irrelevant.

**Conclusion:** In women undergoing hormonal stimulation, the presence of at least one genetic polymorphism for the FSHR gene seems to be related to infertility.

**P-69. Unveiling the Role of lncRNAs in PCOS among Patients Undergoing Assisted Reproductive Cycles**

**Eloiza Adriane Dal Molin^1^, Fernanda Souza Peruzzato^1^, Virgínia Meneghini Lazzari^1^, Yara Maria Rauh Müller^1^**

*^1^ Federal University of Santa Catarina - Florianópolis - SC - Brasil.*

**Objective:** To systematically review the potential roles of long non-coding RNAs (lncRNAs) in polycystic ovary syndrome (PCOS) among patients undergoing assisted reproductive technologies. In this scope, we aim to highlight the relevance of lncRNAs as promising prognostic biomarkers for assisted reproductive outcomes.

**Methods:** The literature search, study selection, and data extraction process adhered to the recommended review structure described in the 2020 Preferred Reporting Items for Systematic Reviews and Meta-Analyses (PRISMA) guidelines. The search was conducted in the Science Direct, Scopus, and Embase databases to identify articles highlighting the relevance of lncRNAs in PCOS management within assisted reproduction. The inclusion criteria encompassed patients undergoing *in vitro* Fertilization (IVF) or Intracytoplasmic Sperm Injection (ICSI) diagnosed with PCOS based on the Revised Rotterdam Diagnostic Criteria. The search was limited to English articles published within the last 10 years (2013-2023). Studies conducted in species other than humans or outside the scope of the study were excluded. For each study, information regarding study design, publication date, sample size, analyzed lncRNA, and, if available, additional results were collected. Subsequently, categories were created based on the composition of the included articles and their main focus. These categories included parameters such as apoptosis rates, cell proliferation, patient characteristics, and relevant additional predictors.

**Results:** The search yielded 17 prospective articles, encompassing a total of 1,534 analyzed patients, of whom 821 were diagnosed with PCOS. All 17 studies included in this review reported variances in the expression of specific lncRNAs between patients with and without PCOS. In this context, five studies have linked specific lncRNAs to physiological characteristics of patients. AC095350.1 lncRNA was found to correlate with age, LINC-01572:28 expression was linked to basal testosterone levels, HCG26 upregulation was associated with antral follicle count (AFC), and MALAT1 was associated with BMI. Moreover, MALAT1 expression was also correlated with pregnancy outcomes in PCOS. The significantly elevated lncRNA named HUPCOS in GCs was positively correlated with follicular fluid testosterone of PCOS patients. Additionally, seven lncRNAs (NONHSAT101926.2, NONHSAT136825.2, NONHSAT227177.1, NONHSAT010538.2, NONHSAT191377.1, NONHSAT230904.1, ENST00000607307) were related to hormone levels and AFC. Six studies have reported an association between increased rates of apoptosis in granulosa cells (GCs) and the overexpression of LINC00173, HOTAIRM1, lnc-CCNL1-3:1, SRLR, HLA-F-AS1, and PLAC2. Additionally, three other studies have linked the inhibition of GCs proliferation with the expression levels of lncRNAs MAP3K13-7:1, MALAT1, and LINC-01572:28. Conversely, TUG1 expression may contribute to excessive follicular activation and growth, while the lncRNA H19 may play a role in regulating GC growth.

**Conclusion:** In conclusion, our systematic review highlights specific lncRNAs that are associated with physiological variables, such as patients' age, BMI, AFC, and testosterone levels, while also showing correlations with GCs proliferation and apoptosis rates. These findings underscore the emerging significance of lncRNAs as promising therapeutic targets and biomarkers, offering valuable insights into treatment prognosis for effectively managing PCOS within the field of assisted reproduction.

**P-70. Seminal concentration of patients who performed laboratory evaluation pre and post COVID pandemic**

**Andressa Thais Culpi^1^, Kahisa Natiele Fontana^1^, Mayara Fatima Frazao Patussi^1^, Vanessa Anjos Ribeiro^1^, Helena Lourenço Zielonka^1^, Lidio Jair Ribas Centa^1^**

*^1^ Androlab - CURITIBA - PR - Brasil.*

**Objective:** Changes in the lifestyle of the general population during the pandemic, not just the coronavirus infection, may have had an impact on male fertility. The aim of this study was to compare the sperm concentration of patients who underwent sperm analysis before the pandemic and those who underwent it after the beginning of the COVID-19 pandemic in Brazil.

**Methods:** Retrospective study based on the analysis of the results of spermograms and questionnaires with authorization to use patient data from a private fertility clinic located at Curitiba in the period between October/2017 and July/2023. Patients were divided into two groups: spermograms performed pre-Covid pandemic (October/2017 to February/2020) and post-Covid pandemic (March/2020 to July/2023). The seminal parameters were evaluated as recommended by the World Health Organization (WHO) at the WHO laboratory manual for the examination and processing of human semen in 2021, and the concentration of 15 million spermatozoa/mL was considered as the cutoff line.

**Results:** 2127 spermograms were evaluated, 1000 of them pre-Covid pandemic and 1127 after the start of the Covid pandemic. In pre-pandemic patients, 18% of them had concentrations below 15 million/mL, whereas in post-pandemic patients, 28% had concentrations below 15 million/mL. Regarding the average concentration of spermatozoa/mL, in the pre-pandemic period it was 63 million, while in the post-pandemic period the average concentration was 54 million spermatozoa/mL.

**Conclusion:** This study showed that after the coronavirus pandemic there was an increase in the number of patients with low concentration and a drop in average sperm concentration. Considering the size of the sample analyzed in this study, male fertility was affected by the pandemic. More studies are still needed to understand how this occurs.

**P-71. Fertility preservation in patients with endometriosis: which are the treatments that shows the better results**

**Giuliana Potthoff Passos^1^, Ana Clara Monteiro Alves^1^, Jaqueline Correia Ribeiro^1^, Juliana Fernandes Dutra^1^, Paula Vaccarezza Lopes^1^, Márcia Sacramento Cunha Machado^1^**

*^1^ Escola Bahiana de Medicina e Saúde Pública - Salvador - BA - Brasil.*

**Objective:** Identify the more effective treatments in the fertility preservation in patients with endometriosis

**Methods:** This systematic review followed the recommendations of Preferred Reporting Items for Systematic Review (PRISMA), reaching identify the better therapeutic outcomes for fertility preservation in women affected with endometriosis according to scientific evidence. A systematic search was performed in the electronic database of PubMed, Cochrane, Scielo and LILACS, in the last 10 years. It was included randomized clinical trial, meta-analysis, cohort studies, in Portuguese and English languages. It was excluded cases report, systematic reviews, book chapter or studies that don’t directly approach the relation between endometrioses and fertility preservation. The keywords used in articles search were Fertility Preservation; Endometriosis; Treatment Outcome; Pregnancy Rate. The final results were obtained from the use of Boolean operator AND for multiples combinations of the Mesh terms corresponding to the descriptors.

**Results:** It was found 21 articles in search platforms, however 13 of them weren’t used due to the inclusion criteria. Of the 8 revised articles, 4 of them showed that surgical laparoscopy must be considered in women with severe and symptomatic endometriosis, increasing the fertility rate. One of them highlighted the importance of an experienced and qualified multidisciplinary team in the successes of surgical procedure. Only one of them suggests surgery after ovarian stimulation. However, there was a disagreement between theses 2 studies, that contraindicated surgery for reducing lesions of endometriosis before pregnancy attempts, despite the gravity of the disease. Two other studies assessed clinical treatment for fertility preservation in women with endometriosis, and one of them concluded that Dienogest (DNG), which is a fourth-generation progestogen, have the same efficiency of the GnRH agonist (GnRH-a). However, the DNG has lower cost and less side effects such as hit waves, body pain, sleep disorder, vaginal dryness, and depressed humor, making the DNG an adequate substitute. Although the other study obtains the same results, when comparing the Progestin Primed Ovarian Stimulation (PPOS) protocol with the GnRH agonist, it was found that the first one has less side effects and added value.

**Conclusion:** Therefore, the majority of the article analyses points to surgical laparoscopic as the better therapeutic alternative for women with severe endometriosis that presents important symptoms and wish to get pregnant. When analyzing clinical treatments, the drug GnRH agonist (GnRH-a) it is shown as more efficient. Though with more added value and side effects than two other different drugs with the same efficiency clamed in the results of two articles. Yet, it is still necessary other studies on this topic with similar results to contribute with these findings, with more efficient treatments that would improve the patient’s quality of live.

**P-72. Type of SARS-CoV-2 vaccine does not affect embryo development outcomes in Assisted Reproduction**

**Carla Giovana Basso^1^, Mariane Cristina Carlucci Molina^1^, Samuel Fortini^1^, Nilo Frantz^2^**

*^1^ Nilo Frantz Medicina Reprodutiva - São Paulo - SP - Brasil.*

*^2^ Nilo Frantz Medicina Reprodutiva - Porto Alegre - RS - Brasil.*

**Objective:** During the SARS-CoV-2 vaccination campaign in Brazil, 4 main types of vaccines have been administered: Pfizer/BioNTech, Sinovac-CoronaVac, FioCruz/AstraZeneca, and Janssen Pharmaceuticals. Pfizer vaccine is an mRNA-based vaccine that do not contain live viral particles, Fiocruz/AstraZeneca and Janssen vaccines are based on the action of adenoviruses, which do not have replicative activity and can stimulate the immune response without the presence of adjuvants and Sinovac-CoronaVac is an inactivated whole-virion SARS-CoV-2 vaccine. The impact of vaccination and type of vaccine on fertility and health in general is the subject of numerous rumors and much contradictory information. Vaccine hesitancy has been a severe problem in eradicating the disease. Therefore, the objective of our study was to evaluate if there was an influence of the different types of SARS-CoV-2 vaccine on the embryo development outcomes of patients undergoing assisted reproduction treatment.

**Methods:** This was a retrospective and observational study conducted from January 2022 to December 2022 in women vaccinated against SARS-CoV-2 who underwent assisted reproductive treatment. The medical records of the patients who had received covid vaccine were retrospectively reviewed to identify the type of vaccine (mRNA vaccine, adenoviruses vaccine or inactivated SARS-CoV-2 vaccine) and the outcomes of their treatment. All data were obtained from our clinical database. We exclude all patients with severe male factor or those who use donor oocytes/sperm. Patients’ characteristics are presents as mean ± standard deviation or percentage. Data was analyzed through personalized multiple linear regression model to access vaccine type influence in embryo development outcomess. Was considered as laboratory outcomes: number of follicles, number of oocytes retrieved, number of mature oocytes, fertilization rate, blastulation rate and good quality embryos rate. Was considered as confusion variables the covariates of maternal age, BMI and infertility cause. Statistical analysis was performed with SPSS 20 (IBM- Chicago,IL,UDSA)

**Results:** We included 64 patients, distributed as follows: 46,8% received a mRNA vaccine (n=36), 19% (n=15) received a adenoviruses vaccine and 15% (n=12) received an inactivated SARS-CoV-2 vaccine. Also, most of the patients underwent treatment with more than 6 months after the first vaccine, only 3% underwent treatment with less than 6 months. None of the couples had any know underlying diseases. Patients included in the study present medium age of 37.6 ± 3.67 and BMI 23.45 ± 3.30, search for treatment was primarily for female reasons (20% Endometrioses, 17% Anatomic factor, 23% Endocrine factor, 11% ovarian insufficiency, 13% male factor, 3% ESCA and 13% other factors). Laboratory rates follow the recommended by the Vienna Consensus. Our data analyses show no influence of type of SARS-Covid vaccination in any of the laboratory reproductive outcomes of patients undergoing assisted reproduction treatment: number of follicles (*p*=0.14), number of oocytes collected (*p*=0.30), number of mature oocytes (*p*=0.15), fertilization rate (p=0.14), blastulation rate (*p*=0.13), good quality embryos (*p*=0.22).

**Conclusion:** Our results suggest that the type of vaccine administered do not influence laboratory or embryo development outcomes of patients undergoing assisted reproductive treatments. Our data agrees with current evidence in the literature regarding SARS-CoV-2 vaccine effects on human fertility, although data is still limited, the findings show no relevant difference in the outcomes observed. Previous publish studies already unanimously state that vaccination does not lead to any detrimental effect on the female reproductive physiology as ovarian reserve, ovarian stimulation, folliculogenises, implantation and sustained implantation rates. Few studies had evaluated laboratory outcomes of IVF, and although our data had a small sample size and must be evaluated with caution, our study provided encouraging results showing that the type of SARS-Covid vaccine does not influence in IVF laboratory outcomes and that from a reproductive perspective vaccine should continuously be safely recommended.

**P-73. Correlation of female and male factors in the embryonic development arrest**

**Fábio Vieira Vilela^1^, Atila Sena Almeida^1^, Daniele Pinheiro de Freitas^1^, Genevieve Marina Coelho^1^**

*^1^ Instituto Valenciano de Infertilidade (IVI) - Salvador - BA - Brasil.*

**Objective:** To evaluate the relevance of female and male factors in vitro fertilization (IVF) cycles where embryonic development arrest occurred.

**Methods:** Descriptive observational case series study in which IVF cycles were analyzed from January 2020 to December 2022, in which embryos did not develop to the blastocyst stage (vs. embryonic development arrest). Couples undergoing ovarian stimulation for ICSI with their own eggs and semen from spontaneous ejaculation were included in the study. Cycles using frozen eggs, donated eggs, frozen semen, testicular/epididymal semen, and donated semen were excluded from the study. The following variables were examined: couple's age, body mass index (BMI), type of ovarian stimulation protocol, number of mature eggs (MII), fertilization rate, day of embryonic arrest and oocyte quality; which was evaluated based on the number of morphological alterations (normal: up to 2/mild: 2-3/severe: ≥3). Among male factors, in addition to age and BMI, the type of semen preparation and quality were also observed, which was classified according to Kruger's morphology as normal (≥4%), mild alteration (2-4%), and severe alteration (≤1%).

**Results:** During the study period, 297 ICSI cycles with arrested embryonic development were screened, of which 128 were excluded. 169 cycles were included in the study according to predefined inclusion criteria. In the general analysis of the data, it was observed that the maternal average age was 40.4 years (±3.5), being under 38 years (24.2%) and over 38 years (75.7%), among which 9.6% were obese (Average BMI: 24.2 ± 3.9). The most used ovarian stimulation protocols were: 225 HMG (45.6%), 31.4% of the low responders used the minimal ovarian stimulation protocol (clomiphene citrate 50 mg/day associated with 150 recombinant (r) FSH + 75 HMG on alternate days) and 18.3% used 150 FSH (r) + 75 HMG. Pituitary blockade was performed with GnRh Antagonist (68.6%) or Dydrogesterone 10 mg/day (31.4%); and the types of “trigger ovulation”: Dual Trigger (93.5%), GnRh Agonist (4.7%) and HCGr (1.8%). The average number of days of ovarian stimulation was 10. A total of 567 mature eggs were recovered, with an average of 3 MII per case, with a fertilization rate of 78% (± 23%). Most ovules had severe alterations (75.3%) and the average time of embryonic arrest was on the 6th day of development (50.3%). Men average age was 41.9 (± 7.4) years, with a avarage BMI of 27.8 (± 4.5). Regarding seminal quality, severe alterations were found in most cases (82.8%) and the main preparation method used was swim-up (50%), followed by washing (32.9%).

**Conclusion:** In IVF treatments, the blastocyst formation rate is a key determinant for reproductive success. Inefficiency of embryonic development is associated with a variety of female and male factors. However, correlating embryonic arrest with only one factor would not be plausible, as this study showed that the majority of women were above 38 years old, which could have already predicted poor oocyte quality and compromise embryo development, despite a good fertilization rate. Therefore, prospective studies with more homogeneous groups will be necessary to better elucidate the causal factors.

**P-74. Impacts of the diagnosis of endometriosis and the importance of the multidisciplinary team in Fertilization Clinics**

**Laura Aires Cavalcante Leite^1^, Tairane Farias Lima^2^, Thaís Farias Lima^2^**

*^1^ Universidade Estadual da Paraíba - Campina Grande - PB - Brasil.*

*^2^ Unifacisa - Centro Universitário - Campina Grande - PB - Brasil.*

**Objective:** To reflect on the impacts of the diagnosis of endometriosis and the importance of the multidisciplinary team in Fertilization Clinics

**Methods:** A search was carried out on a digital platform (Virtual Health Library - VHL), through Health Descriptors (DECS) entitled Endometriosis; Stress, Psychological; Fertility Clinics. After crossing data, 04 articles of the type observational cross-sectional study, descriptive research, randomized clinical trial and quantitative and qualitative research were obtained.

**Results:** Endometriosis is defined as the development and growth of endometrial tissue outside the uterine cavity. According to Ribeiro et al (2021), the general incidence of endometriosis affects between 6 and 10% of women of reproductive age and was found in pre-menarcheal and post-menopausal women. The average age at diagnosis is approximately 28 years. Deep endometriosis as the only form of disease in the absence of other endometriotic lesions was present in only 6.5% of patients. Intestinal infiltration occurs at an incidence ranging from 5 to 12%. In addition to the physical aspects, ranging from dyspareunia and dysmenorrhea to inability to perform work activities, mental aspects are also present, such as, for example, anxiety and depression. Silva et al. (2021) report that the experience of patients until the diagnosis of the disease goes through a series of issues: the alleviation of symptoms through the bond of friends, the difficulty in carrying out the diagnosis and pilgrimage by several professionals until they receive the proper assistance (in addition to the financial strain of the journey). Another aspect that permeates the life of a woman with endometriosis is infertility. Most of the time, due to the reduction of signs and symptoms, women only discover the diagnosis when they try to get pregnant and fail. Pretzel et al. (2021) brought as a result of their research that, of 6,354 women of eligible age with a diagnosis of endometriosis, 421 had a diagnosis of concomitant infertility. Therefore, Fertilization Clinics are, in most cases, the patient's first contact with the frightening diagnosis of endometriosis and infertility. Associated with this, there is an urgency to act, many times, in relation to the treatments/procedures available for pregnancy or preservation of fertility. Many need IVF (In Vitro Fertilization) or egg freezing for social preservation, and therefore undergo the stages of the process as they “digest” such a diagnosis. Lorençatto et al. (2007) concluded in their study that the multidisciplinary intervention was effective in reducing pain and depression in women with endometriosis, and could be incorporated into the conventional treatment offered to women with this disease. The patient assisted by a multidisciplinary team, especially a specialist physician, nurse, nutritionist and psychologist, enjoys different perceptions and, therefore, specialized assistance throughout the treatment.

**Conclusion:** The impacts of the diagnosis of endometriosis range from physical, to mental and social consequences, directly impacting the patient's quality of life. The multidisciplinary team provides specialized care centered on the needs of patients arriving at the Fertilization Clinics. Thus, becoming a necessary strategy.

**P-75. FGF4 supplementation, in in vitro culture of bovine embryos, does not alter embryo kinetics**

**Isabella Maran Pereira^1^, Mariana Gabriély Cândido Bonfim^1^, Lara Ereno Ferreira^1^, Brisa Bertocco Oliveira^1^, Camila Bortoliero Costa^1^, Ramon Cesar Botigelli^2^, Marcelo Fabio Gouveia Nogueira^1^**

*^1^ UNESP - Assis - SP - Brasil.*

*^2^ UCDAVIS - Estados Unidos.*

**Objective:** The fibroblast growth factor (FGF) family has many roles in initial embryo development and can be highlighted as crucial in cell signaling, survival, and division. The transition between totipotent-pluripotent cells is a crucial event to promote the success of embryo development. Moreover, the second cell differentiation event will promote the inner cell mass (ICM) to differentiate into two new cell lines: epiblast (origin of germ layers) and hypoblast (origin of extraembryonic tissues). In special, FGF4 has been reported at the initial embryo development of many species and its exogenous supplementation is inferred to block the epiblast lineage favoring the hypoblast lineage during the ICM’s differentiation, which may have multiple applications at cattle as an important biotechnology tool at manipulating the embryo’s gene expression and advancing the field of embryo chimerism. Thus, this research aimed to test FGF4’s influence on the number of cells per blastocyst, the diameter of the blastocyst and ICM, to evaluate FGF4 supplementation is detrimental to the embryo at kinetics parameters.

**Methods:** Therefore, bovine embryos were produced in vitro and different FGF4 concentrations (0, 10, 100, and 1,000ng/mL) were added on IVC from 120 to 204 hours post-insemination. At least 40 oocytes were used per experimental group at each one of the 8 replicates. After 7 and 8.5 days, the number of cells, blastocyst’s diameter, and ICM’s diameter were measured with the ImageJ software. The data was analyzed by the ANOVA test, differences with the probability of *p*<0.05 were considered significant.

**Results:** None of the groups showed statistical difference at cell count (*p*=0.74), blastocyst’s diameter (*p*=0.14) and ICM’s diameter (*p*=0.07)

Conclusion: It can be inferred that FGF4 has not prejudiced the bovine embryo’s parameters tested at the supplemented concentrations, opening gates for future research involving this molecule.

**P-76. The effect of cannabis use on female fertility**

**Maria Clara Elise Santana^1^, Luana Nayara Gallego Adami^1^**

*^1^ Embriológica - São Paulo - SP - Brasil.*

**Objective:** To gather and understand the impact of marijuana/Cannabis consumption on female reproductive health and the outcomes of neonatal complications.

**Methods:** From a bibliographic review, data were collected from several articles related to the proposed subject, published between 1979 and 2022. The articles were selected after conducting a search in the literature, through the platforms Google Scholar and PubMed. Studies were included after a thorough reading of the abstracts and after the complete articles, with a English and Portuguese language, including papers with both basic and clinical approach.

**Results:** After the search strategy, 61 articles were selected. Two of those were excluded due to lack of access to the full article. Therefore, related articles were included in the proposed review. Cannabis/marijuana is one of the oldest known psychotropic drugs and a champion of consumption especially among people of reproductive age. The best studied components in cannabis are the cannabinoids named cannabidiol (CBD) and ∆9-tetrahydrocannabinol (THC), which is the main psychoactive ingredient. Cannabinoid-like compounds have been identified in vivo, collectively referred to as endocannabinoids (ECB). The endocannabinoid system (ECS) is a multifunctional homeostatic system involved in diverse physiological and pathological conditions. The biological effects of cannabis are mediated through this system, and studies report the presence of cannabinoid receptors in the female reproductive tract, suggesting that the endocannabinoid system plays a role in regulating reproductive physiology. ECS ligands are ECB, whose actions are mimicked by exogenous cannabinoids such as THC and CBD. Responses to ECS ligands are mediated by numerous receptors such as CB1 (cannabinoid receptor 1) and CB2 (cannabinoid receptor 2), both of which are present in the female reproductive system. Thus, we observed that exposure to cannabinoids can have differential impacts on female reproductive health throughout a woman's life, from preconception to pregnancy, either naturally or through assisted reproduction techniques, and during lactation. Many studies have shown that follicular development is negatively affected as the presence of exogenous cannabinoids reduces the release of hormones from the hypothalamic-pituitary-ovarian axis including sex hormones such as LH (luteinizing hormone) and FSH (follicle stimulating hormone). Furthermore, user’s ovaries were shown to be anovulatory, leading to primary infertility, in addition to the shortening of the luteal phase in women with chronic use of the substance. Pregnant women using either natural or synthetic derivatives were associated with gestational disorders, such as abnormal embryo development, tubal pregnancy, implantation failure, premature delivery, intrauterine growth restriction, low birth weight and increased risk of spontaneous miscarriages. Although little is known about its effects on lactation, it is known that THC easily crosses the placental membrane and, being liposoluble, is transferred through breast milk. Several results indicate that appropriate and regulated endocannabinoid signaling, both in the blastocyst and in the uterus, is necessary for the establishment of uterine receptivity and blastocyst implantation competence, reducing the chance of pregnancy failures. However, it is necessary to better understand the doses and physiological mechanisms that involve the cannabinoid system so that there is an established threshold between benefits and risks.

**Conclusion:** The data indicate a negative effect of cannabis use on female fertility during many stages of female reproductive life, even in hormone and gamete production, or during pregnancy. However, the literature still lacks concrete, controlled and sufficient data to actually ensure that there is damage, since it is still not well understood how the endocannabinoid system acts in reproductive and fertilization processes.

**P-77. Cytogenetic analysis in couple with recurrent pregnancy loss: The state of art**

**Héllen Néo da Rocha^1^, Cley Gabriel Lima Carvalho Dantas^1^, Emerson de Santana Santos^1^, Fabricia Correia de Azevedo^1^, Gabriel Silva Morato^1^, Gilberta Guadalupe de Souza Santos^2^, José Edeilson dias Vitorino^1^, Pollyanna Andreza Ribeiro dos Santos^2^, Yasmin Casado Fortunato^1^, Leonardo de Oliveira^1^, Antônio Carvalho Azevedo^1^, Ritta de Kássia Oliveira de Santana^1^, Hélison de Jesus Oliveira^1^, Danilo Santana Santos^1^, Aline de Jesus Lima^1^**

*^1^ Fundação Universidade Federal de Sergipe - São Cristóvão - SE - Brasil.*

*^2^ Hospital Universitário da Universidade Federal de Sergipe - Aracaju - SE - Brasil.*

**Objective:** To present the prevalence of chromosomal abnormalities detected by conventional karyotyping in couple with recurrent pregnancy loss (RPL) and their products of conception (POC).

**Methods:** Literature search was perfomed using health databases Medline, Lilacs, Scielo, and PubMed, with the keywords "Cytogenetic analysis" AND "Abortion, Habitual" AND "Embryo loss". Articles in Portuguese, Spanish and English available on virtual platforms were included. Only papers published within the last 5 years were selected and the duplicated ones were removed. Cases reports were also excluded. Cross-sectional studies, cohort studies, and literature reviews were considered eligible for inclusion.

**Results:** 87 papers were found. After a thorough reading of the potentially relevant articles, 8 studies met the inclusion criteria and were used to produce this review. Chromosomal anomalies are found in more than half of early pregnancy losses. Cytogenetic analysis holds clinical relevance by allowing couples to make decisions after finding out whether a new conception is viable or not. karyotype has limitations as a method of cytogenetic analysis, especially with regards to analysis of products of conception (POC), with frequent culture failure, contamination of the material by maternal cells, and low resolution that does not allow the detection of subtle chromosomal anomalies. Therefore, chromosomal microarray analysis (CMA) has been considered as the first-tier test for genetic investigation of pregnancy loss but it is still costly and difficult to access. The prevalence of fetal chromosomal aberrations detected in cases of Recurrent Pregnancy Loss (RPL) ranged from 40.4% to 61% in the analyzed papers. The main abnormalities observed were trisomies of chromosomes 13, 16, 18, 21, and monosomy of the X chromosome. Regarding the karyotype investigation of the evaluated couples, a variation of 3.74% to 7.4% of abnormalities was found. The main structural aberrations were balanced translocations, Robertsonian translocations, and inversions, respectively. These abnormalities were more frequent in women than in men, and the chromosomically affected women were on average 3 years younger than the unaffected ones. Another finding was the positive correlation between the number of previous miscarriages and the frequency of chromosomal abnormalities. Furthermore, one of the studies infers that if the fetal karyotype result is normal, the analysis of the genetic material of the parents is unnecessary since it guarantees that the spontaneous abortion is not related to any hereditary chromosomal abnormality, and it is also cost-effective. If the cytogenetic test of the Product of Conception (POC) is not available, a peripheral blood test for the karyotype of both partners should be considered. Even in cases of abortion after assisted conception with pre-implantation genetic diagnosis, the evaluation should include the analysis of the peripheral karyotype of the parents and the POC.

**Conclusion:** Cytogenetic analysis of couples with RPL showed a predominance of structural chromosomal abnormalities in females and the occurrence of these alterations at a younger age compared to women without chromosomal anomalies. Therefore, genetic counseling and cytogenetic analysis should be part of the investigation for any couple, as it allows better support during the grieving process, in addition to being economically viable.

**P-78. Food and optimizing fertility: How nutritional choices can enhance the journey to conception**

**Ligia Previatto^1^, Carolina Colombelli Pacca^1^, Kelly Colussi^1^, Letícia Rolin^1^, Leonardo Previato Araujo^1^, Edilberto Araujo Filho^1^**

*^1^ C.R.H. Centro de Reprodução Humana, São José do Rio Preto/SP - São José do Rio Preto - SP - Brasil.*

**Objective:** This study aims to conduct an integrative literature review investigating the influence of nutrition on fertility and assisted reproduction, emphasizing the significance of nutritional modulation.

Methods: An integrative review was performed using the PubMed and Scielo databases. The selected descriptors were infertility, assisted reproduction, nutrition, and plant-based food. Initially, 234 articles were found, and after applying the eligibility criteria, 47 articles were chosen for analysis.

**Results:** Adequate modulation of nutrition plays a pivotal role in fertility, with a particular focus on body weight adjustment and the reduction of inflammatory processes and their markers. A diet rich in specific nutrients, such as vitamins, minerals, antioxidants, omega-3, and omega-6 fatty acids, can improve sperm and egg quality, thereby increasing the chances of conception. In this study, we observed that 31.8% of the analyzed articles highlighted the fundamental role of vitamin B12 in improving fertilization and fertility, as it is responsible for transporting folic acid (B9) into the cells, requiring joint supplementation of vitamins. This combination becomes essential for successful DNA synthesis, cell replication, and healthy embryonic development. Another relevant aspect is the modulation of homocysteine, observed in 10.6% of the analyzed studies. Elevated levels of homocysteine are associated with damage to the endothelium, affecting microcirculation, and increasing the risk of pre-eclampsia. Studies indicate that supplementing vitamins B12 and B9, along with other nutrients, can lower homocysteine levels, improving vascular health and fertility. The modulation of inflammatory factors through the intestinal microbiota has also been studied. Components such as LPS (lipopolysaccharides), Toll-like receptors, and TNF-alpha can trigger inflammatory and immunological responses, such as a TH1 response, impairing implantation. Promoting a healthy, balanced microbiota through a diet low in saturated fat, a good supply of fiber-rich carbohydrates, and supplementation with probiotics, when necessary, can modulate inflammation, thus improving reproductive health. In this study, we were able to observe the importance of the microbiota in 12.8% of the analyzed articles. Insulin regulation also plays a significant role in fertility. Insulin resistance can interfere with ovulation and egg quality, affecting fertilization, as observed in 12.8% of the reviewed articles. Decreasing the consumption of foods rich in saturated fat, ensuring an adequate intake of macronutrients, and engaging in regular physical exercise can improve insulin sensitivity, promoting optimized reproductive function. Mitochondrial biogenesis, the process of forming new mitochondria, is also associated with fertility. In this sense, it was observed that nutritional modulation, tailored to individualized needs for macronutrients, omega-3 and omega-6 fatty acids (8.5% of articles), antioxidants (19.1% of articles), CoQ10 (4.2% of articles), and B vitamins (19.1% of articles), can improve mitochondrial function, thereby promoting better quality of eggs and embryos.

**Conclusion:** Nutritional modulation plays a crucial role in fertility. Adequate vitamin B12 and B9 supplementation, homocysteine modulation, reduction of inflammation, and improvement of insulin sensitivity are important strategies to enhance both female and male fertility. These nutritional interventions can create a favorable environment for embryonic implantation, improving success rates, and increasing the chances of clinical pregnancy.

**P-79. Platelet-Rich Plasma Infusion in Infertile Women - The Future of Assisted Human Reproduction?**

**Ashlley Souza de Moraes^1^, Hellen Ingrid Santos Cardoso^1^, Igor Ventura Brandão^2^, Jose de Melo Bomfim Neto^1^, Maria Clara Santos Chaves^2^, Sabrina Maria Rodrigues Jacinto Costa^1^, Thamara Matos dos Santos^1^, Emilly Freitas Oliveira^1^**

*^1^ Clínica Integrada de Assistência Médica da Mulher - Fertilitá - Aracaju - SE - Brasil.*

*^2^ Universidade Tiradentes - Aracaju - SE - Brasil.*

**Objective:** To perform a comparative analysis of the variables involved in Platelet-Rich Plasma (PRP) processing and its effects on the outcomes of patients undergoing infusion for the treatment of infertility.

Methods: This is a systematic literature review of a descriptive, exploratory, and cross-sectional nature. The search was conducted on major scientific article platforms such as Scielo, PUBMED, Google Scholar, and Science Direct. The inclusion criteria were publications from 2015 to 2023 with titles directly related to the action of PRP (Platelet-Rich Plasma) in the endometrium, processing methodologies, pathologies, and patient screening for this study. Articles that did not address the topic or were outside the time range of 2015 to 2023 were excluded. As a result, 9 relevant articles were found, comprising 4 randomized studies and 5 case reports.

**Results:** The analysis revealed variability in PRP methods and handling, but some common points regarding the patients' profile drew attention. Pathologies such as repeated implantation failure (RIF), Asherman's syndrome, salpingitis, and polycystic ovary syndrome were more prevalent, along with two or more previous frozen embryo transfer (FET) attempts, ages between 20-45 years, and endometrial thickness <7 mm. Regarding the PRP processing and handling methodology, the collected blood volume varied, but the predominant anticoagulant was acid citrate dextrose (ACD-A). The centrifugation force/velocity was measured in revolutions per minute (RPM) and gravitational force (G-force), with double centrifugation being most common. The final platelet concentrate ranged between 0.5 - 1.0 mL. It is important to note that all studies involved patients under hormonal replacement therapy (HRT) protocols during the FET cycle. Additionally, the predominant PRP infusion site was intrauterine, and the interval between infusion and FET varied from 24 to 48 hours, as reported by the authors who provided this information. To assess the post-infusion effects, endometrial thickness was measured using ultrasonography (USG), and the majority showed thicknesses >7 mm compared to the previous measurement. Out of the 9 studies, 7 obtained pregnancy rates above 40% concerning the sample size of patients.

**Conclusion:** As an experimental technique, there are variables that can be adjusted to optimize PRP processing and achieve more significant results. For instance, the standardization of a base methodology for PRP processing, from collection to infusion, could be beneficial. Moreover, attention is drawn to the lack of an exact interval of hours between infusion and FET, which is an important factor considering the implantation window, favoring embryo implantation and support. Further standardized studies are needed to verify such considerations. Nevertheless, It is evident that PRP infusion treatment in the endometrium has shown increased pregnancy rates compared to previous frozen embryo transfer (FET) attempts and has positive effects on endometrial thickness, making it a promising adjuvant alongside hormonal treatments and in the FET cycle.

**P-80. Case report of assisted hatching (AH) and multiple pregnancy**

**Rodrigo Romano^1^, Graziela Yuri Ninomiya^1^, Carolina Federicci Haddad^2^, Guilherme Loureiro Fernandes^3^, Francoise Elia Mizhari^4^, Jonathas Borges Soares^4^**

*^1^ Projeto Beta Reprodução - São Paulo - SP - Brasil.*

*^2^ Departamento de Obstetrícia e Ginecologia da Santa Casa de Misericórdia de São Paulo - São Paulo - SP - Brasil.*

*^3^ Medicina Fetal da Faculdade de Medicina do ABC - São Paulo - SP - Brasil.*

*^4^ Projeto Alfa Reprodução - São Paulo - SP - Brasil.*

**Objective:** To report a case of in vitro fertilization (IVF), with transfer of 2 embryos resulting in clinical pregnancy with 4 gestational sacs.

**Methods:** Case report description from a fertility clinic in São Paulo, Brazil.

**Results:** Couple with infertility for 12 months with male factor (idiopathic oligoasthenozoospermia). Intracytoplasmic sperm injection (ICSI) was performed and all embryos were frozen, with delayed transfer due to risk of ovarian hyperstimulation syndrome. Due to thickening of zona pellucida and unfavorable laboratory outcome in previous cycle, AH was performed. Two thawed embryos were transferred at the cleavage stage with positive pregnancy test. In the 7th week, four gestational sacs were identified, all with yolk sac and embryonic pole with present cardiac activity. In the 1st trimester ultrasound, a twin pregnancy and missed abortion were observed in two gestational sacs.

**Conclusion:** Although AH is not routinely indicated, some laboratories use it in selected cases due to thickening of zona pellucida or for embryonic biopsy. Patients should be informed about the possibility of embryonic cleavage and development of multiple pregnancies, with their associated risks. The case corroborates the literature as there is evidence of assisted hatching and increased incidence of multiple pregnancies.

**P-81. Evaluation of Ovarian Reserve in Women with Thyroid Autoimmunity**

**Adriana Leal Griz Notaro^1^, Alex Sandro Rolland Souza^1^, Filipe Tenorio Lira Neto^2^, Giuliano Marchetti Bedoschi^3^, Maria Jéssica da Silva^4^, Mariana Corrêa Nunes^4^, Catharina Cavalcanti Pessoa Monteiro Lira^5^, José Natal Figueiroa^6^**

*^1^ IMIP - Recife - PE - Brasil.*

*^2^ ANDROS Recife - Recife - PE - Brasil.*

*^3^ USP Ribeirão Preto - Ribeirão Preto - SP - Brasil.*

*^4^ AMARE - Recife - PE - Brasil.*

*^5^ CM Medicina Reprodutiva - Recife - PE - Brasil.*

*^6^ IMIP - Recife - PE - Brasil.*

**Objective:** To compare the ovarian reserve of women of reproductive age with and without thyroid autoimmunity (TAI)

**Methods:** We performed a retrospective analysis of medical records from an assisted reproduction clinic from February 2017 to December 2021. Women aged between 18 and 49 years with data on antithyroperoxidase and antithyroglobulin (anti-Tg) antibodies and assessment of ovarian reserve by anti-müllerian hormone (AMH) and antral follicle count (AFC) were included. Participants positive for anti-thyroperoxidase (anti-TPO) and/or anti-thyroglobulin (anti-Tg) antibodies, according to the reference range of the laboratory where the tests were performed, were considered to have TAI. Low ovarian reserve was defined based on the criteria adapted from POSEIDON (AMH < 1.2ng/mL and/or AFC < 5 follicles). AMH and AFC were compared between groups. Subanalysis based on age, types of antibodies, and thyroid function markers were performed. In addition, bivariate analysis and regression models were used.

**Results:** Among the 188 participants included, 63 were diagnosed with TAI, and 125 had both antibodies negative. The two groups were similar regarding the baseline characteristics. Overall, there were no differences in the median levels of AMH or AFC between the groups. However, in the subgroup analysis by age, we observed a trend towards lower median levels of AMH in women over 39 years with TAI (0.9 ng/mL vs. 1.5 ng/mL, *p* = 0.08). In a subanalysis according to antibodies, we found a significantly lower median AFC in the group with anti-Tg than in the group without this antibody (8.0 follicles vs. 11.5 follicles, *p* = 0.036). We also found a significantly higher prevalence of anti-Tg in patients with low ovarian reserve compared to those with normal reserve (60.7% vs. 39.3%, *p* = 0.038). A multivariate regression analysis indicated that age (*p* < 0.001) and the presence of other autoimmune diseases (*p* = 0.035) were the only variables independently associated with low ovarian reserve (Table 1)

**Conclusion:** Our study showed a trend towards lower AMH levels in women over 39 years of age with TAI compared to age-matched controls without TAI. In addition, we found lower AFC in women with anti-Tg, and that women with low ovarian reserve had a higher prevalence of this antibody. Thus, the ovarian reserve of women with TAI appears to be insidiously compromised over the years, with a decreased ovarian reserve in women with antithyroglobulin positive. To confirm our findings, longitudinal studies including women screened for the presence of both antibodies and with their ovarian reserve evaluated through more than one marker are needed.

**Table 1:** Results of Poisson multivariable regression models to identify variables associated with ovarian reserve.

Variables	RR (95%CI)	p value
BMI (Kg/m^2^) Obesity No obesity	1.1 (0.71 - 1.73) 1.0	0.645
**Other autoimmune disease** Yes No	1.7 (1.04 - 2.70) 1.0	**0.035**
**Endometriosis** Yes No	1.1 (0.78 - 1.47) 1.0	0.671
**Thyroid autoimmunity** Yes No	1.2 (0.90 - 1.71) 1.0	0.183
**Age (years)** < 35 35 - 39 ≥ 40	1.0 2.07 (1.17 - 3.65) 3.8 (2.23 - 6.53)	**< 0.001**
**TSH**	1.12 (0.98 - 1.28)	0.108

RR = relative risk; CI = confidence interval; BMI = body mass index; TSH = thyroid-stimulating hormone.

**P-82. Advanced Paternal Age and laboratory outcomes in IVF treatment**

**João Roberto Paladino Jr^1^, Carla Giovana Basso^1^, Gabriela Nascimento da Silva^1^, Nilo Frantz^1^**

*^1^ Nilo Frantz - Sao Paulo - SP - Brasil.*

**Objective:** Advanced paternal age (APA) has been recognized as a potential risk factor in In vitro fertilization (IVF) outcomes in the last years with increasing evidence showing that as much as advance maternal age (AMA), APA can also have deleterious effect on gamete genetics, development and conception rates. However, there still no consensus for the definition of APA and few studies had explore a clear effect of paternal age stratification in IVF results, especially embryo development outcomes. Our objective was to access the possible correlations between paternal age stratify and embryo development outcome.

**Methods:** We collect data from January 2020 to December 2022 from all IVF cycles perform in our center, we excluded all procedures with mild/severe male factor or those conducted with donor/frozen samples. The medical records of the patients include in the study were retrospectively reviewed to identify embryo development parameters. Patients’ characteristics are presented as mean ± standard deviation or percentage. Male patients were categorized by age in the time of procedure and stratified in 3 groups as ≤34, 35-39, ≥40 years old. We included as embryo development outcomes: fertilization rate (number of fertilized embryos/number of inject oocytes), blastulation rate ( total number of blastocysts/ number of fertilized embryos) and good quality embryo rate (number of good quality blastocyst/ number of fertilized embryos) - being considered good quality embryos those with morphological classification A or B in inner cell mass and trophectoderm as Garner parameters. Continuous variables were compared between age groups using analysis of variance if normally distributed or Kruskal-Wallis if not normally distributed. Chi-square or Fisher’s exact test were used whenever appropriate for comparing categorical data. Male age group was correlate trough Pearson correlation test with the embryo development outcomes to access possible correlation between male age category and laboratory outcomes in IVF cycles. Statistical analyses was conducted with the software SPSS 20 (IBM- Chicago,IL,UDSA).

**Results:** A total sample of 240 IVF cycles were included in our study. Medium male age of ≤34 group was 34 ±0 years old(N=7), 35-40 group was 37.23 ± 1.82 years old (N=113), ≥40 group 44.58 ± 5.22 years old (N=110). Respectively female age was 35.33 ± 2.58, 35.99 ± 2.99, 38.8 ± 2.65. No significant difference was found between age groups in any of the continuous or categorical variables analyzed. Medium fertilization rate in each group was, respectively, 81,5%, 70,1% and 72,1%, medium blastulation rate was, respectively, 49.2%, 50.7% and 50.7%, and lastly medium good quality embryos rate was, respectively, 23.3%, 36.2% and 31.6%. A negative correlation was observed between male age and good quality embryos (-0,138, *p*= 0.034). However no significant result was observed with male age stratifications.

**Conclusion:** Previous data already extensively assessed the potential influence of paternal age in IVF outcomes, still few studies have found clear correlation between advanced paternal age and rates of fertilization, implantation, pregnancy, miscarriage, and live birth. In our results paternal age was found to correlated with embryo quality at blastocyst stage, what could reflect male genomic activation within the embryo. Our goal to observed if male age stratification could show impact on laboratory results show no significance.

**P-83. Fresh or Vitrified eggs? Evaluation of embryonic development and euploidy in an egg donation program**

**Jose Manuel Gonzales^1^, Gilana Jazmin VillaLobos^1^, Moises Sanchez^1^, James Milthon Mestanza^1^, Flor Carvallo1, Alberto Franco^1^, Jose Pimentel^1^, Jonathan Vasquez^1^**

*^1^ Centro de Fertilidad Germinar - Peru.*

**Objective:** To compare the use of fresh and vitrified eggs regarding the results in rates of fertilization, blastulation, day of blastulation, and embryonic euploidy in an egg donation program.

**Methods:** The embryonic development results of 427 egg donation cycles were analyzed, 147 using fresh donated eggs and 280 using vitrified donated eggs. These procedures were performed as part of the egg donation program of the Germinar Fertility Center (Lima, Peru). The study included only intracytoplasmic spermatozoa microinjection (ICSI) procedures, using ejaculated semen and where the total motile sperm count of the initial sample is greater than 1 million spermatozoa. Comparison of means with the student's test was used to compare the embryonic development of fresh (n=1535) and vitrified (n=2908) eggs, by evaluating the percentage of fertilization and blastulation, and to analyze the proportion of embryos that reached the blastocyst stage on the fifth or sixth day. Also, within our egg donation program, a group of 166 cases had an indication for genetic study, of which 72 cases were performed with fresh egg and 94 with vitrified egg, and by means of the chi-square test, we determined whether the origin of the egg influenced the euploidy result. Finally, applying simple linear regression, the performance of the egg donation program was analyzed depending on the origin of the egg by comparing the number of blastocyst embryos produced between both groups and taking as a starting point the number of donated eggs given to each group (fresh vs. vitrified).

**Results:** A lower fertilization (79.6% vs. 85.2%, *P*<0.05) and blastulation (49.2% vs. 61.5%, *P*<0.05) rate was observed in cases where vitrified eggs were used compared to cases where fresh eggs were used, in addition, a higher percentage of blastocysts were produced in D5 from fresh eggs compared to vitrified eggs (79.6% vs. 74.6%, *P*=0.012). However, when analyzing the rate of embryonic euploidy using fresh egg, 220 euploid embryos were obtained out of 316 analyzed (69.6%) and using vitrified egg, 205 euploid embryos were obtained out of 314 analyzed (65.3%), with no significant differences between both groups (*p*=0.52). Finally, with simple linear regression, we were able to determine that, in egg donation procedures, there is a greater effect (12.5%) using fresh eggs in the generation of blastocysts with respect to procedures with vitrified eggs. This means that to equal the number of blastocysts obtained in a patient using fresh eggs, 12.5% more eggs must be used in cases with vitrified eggs. For example, if a recipient is allocated 8 fresh eggs, it must be considered to give 9 eggs (12.5% more) in case we use vitrified eggs and thus produce a similar number of blastocysts between both types of procedures.

**Conclusion:** Egg donation procedures using vitrified eggs have lower fertilization and blastulation percentages compared to egg donation cycles using fresh eggs, however, the euploidy rate between both groups is similar. To match the number of blastocysts achieved in the fresh egg group, we only need to provide 12.5% more eggs if we use vitrified eggs.

**P-84. Use of high dose modified protocol, with GnRHant and Cabergoline in the treatment of Ovarian Hyperstimulation Syndrome**

**Lister Lima Salgueiro^1^, Suelen Patricia Pinheiro Machado Lima^1^, Bernardo Rodrigues Lamounier Moura^1^, Adrielly Silva Camargo Stefani^1^, Samira Pedro Orlando^1^, Edson Guimarães Turco^1^**

*^1^ Clínica Fértilis - Sorocaba - SP - Brasil.*

**Objective:** Analyze the Ovarian Hyperstimulation Syndrome (OHSS) data, in the clinic cases, when used, High Dose GnRH antagonist, plus Cabergoline and symptomatic (adjuvant) medications.

**Methods:** A retrospective observational study, with the evaluation of, Fértilis Assisted reproduction clinic data. Fifty-five patients with OHSS were treated with Cetrorelix in high dosage, plus Cabergoline and symptomatic medications (Albumin, Metamucil, Kyss Kryss, Tramadol, Aspirin, Simethicone, and Oral Hydration) on the day of oocyte pickup. In patients with more than 15 eggs collected, Estradiol more than 3000 pg/ml and low body composition. Because of the possibility of lethality in the cases, we did not have a control group, and we compared the results with published data.

**Results:** Of 55 patients with OHSS, submitted to the protocol, (78.0%) were classified as mild, (22.0%) as moderate, and we did not have severe cases. Of the mild cases (24.0%) did not have clinical symptoms, (55.0%) had a 4-day period with symptoms, (14.0%) had 7 days with symptoms, and (7.0%) had 10 days with symptoms. Sixteen patients (29.0%) were submitted to a Culdocentesis in the clinic, due to the ascites, and there were no hospitalizations. With respect to age, patients (55.0%) had less than 35 years, (36.0%) had between 35 and 39 years, and (9.0%) had more than 40 years. In (60.0%) of the cases, the patients were diagnosed with PCO, (20.0%) with Endometriosis, and (25.0%) with low testosterone levels. Some cases had more than one pathology.

**Conclusion:** The use of the protocol with Cabergoline, and GnRHant alone, or in conjunction, diminishes but does not avoid the presence of severe OHSS. The modified treatment with high-dose GnRHant, plus Cabergoline and adjuvant medications, after egg collection, showed efficiency in the control of OHSS. The action of the GnRHant diary plus Cabergoline is fast (24h), but the higher dose at once, seems to have a more drastic effect on the hormonal block. The modified treatment with Cetrorelix in high doses plus Cabergoline and adjuvants were made on an outpatient basis, avoiding hospitalizations, shortening the time, diminishing the level of the symptoms, avoiding severe cases and hospitalizations, and could be a safe alternative to conventional treatments.

**P-85. Lose to win? - Psychoeducation, Infertility and bariatric surgery**

**Rose Marie Massaro Melamed^1^, Kátia Maria Straube^2^**

*^1^ Fertility Medical Group - São Paulo - SP - Brasil.*

*^2^ Androlab & Feliccitá - Curitiba - PR - Brasil.*

**Objective:** Infertility is a condition currently recognized as a source of suffering that can be intense and devastating, especially when associated with physical and/or psycho-emotional comorbidities. One of these could be obesity, a situation that has been proven to hamper hormonal functions, which affects daily activities and health, and possibly reproductive health. Having adequate weight is an important factor for success in reproductive treatments. Often, these become unnecessary after treatments for obesity or, at least, are favored with increased possibilities of success. This leads many women and men to undergo bariatric surgery, even on medical advice, with the aim of increasing the chances of pregnancy, when this delay occurs naturally. However, the difficulty to get pregnant is associated with the restrictions resulting from the surgery, the waiting time for the best conditions to receive the embryo and be able to carry it without the risk of low weight or premature birth, as well as the possibilities of indication for the replacement uterus procedure, as an alternative to continue the process, since the expected positive result does not always happen. It is known that the vital crisis that triggers infertility generates significant emotional disorders, emphasizing the need for the multidisciplinary team of Assisted Reproduction to be aligned, including the Endocrinology team. Clinical practice through the observation of these cases, in individual and couple interviews, made us think of a project to welcome patients who underwent bariatric surgery to get pregnant through reproductive treatments and obtained a negative result, with a view to: - offer the necessary contributions to the psycho-emotional development of these patients, who find themselves in the “intergame of losing to win, not winning”; - contribute to more conscious decisions about treatment and its continuity; - provide professional host support, needed at this time.

**Methods:** Psychoeducation, with individual and group sessions, can provide important support from four areas: Endocrinology - metabolic and endocrinological diagnosis; Nutrition - with diet customization, food re-education, habits and needs of each patient; Physical Education - with individualized physical training, physiotherapy, massages, outdoor activities; and Psychology - work on bereavement, psychological motivation for maintaining habits and lifestyle.

**Results:** It is expected that the conduct of the professionals involved can contribute to minimize the self-responsibility of these patients and the consequent characteristic guilt, for not meeting the necessary standard, whether clinical, metabolic, aesthetic or psycho-affective, of not fulfilling the desire to become pregnant.

**Conclusion:** Psychoeducation is a recognized therapeutic intervention capable of providing an important instrument to promote the expansion of knowledge and management about illnesses and their repercussions through an integral approach, which offers space for openness to clear and consistent information, as well as sharing of experiences and increment to self-care.

**P-86. What is the role of psychoeducation when assisted reproduction is a resource for family building?**

**Helena Prado Lopes^1^, Valéria Teixeira Batista^2^**

*^1^ PRO-FERTIL - RIO DE JANEIRO - RJ - Brasil.*

*^2^ Clínica Perfetto Reprodução Humana - Goiânia - GO - Brasil.*

**Objective:** Desiring to have children and dealing with difficulties and/or the inability to conceive stirs up a mix of emotions. This study aims to analyze emotional responses of couples facing infertility while undergoing reproductive treatments, evaluating the impact of their experiences on marital, social, and familial relationships. In the diagnosis of infertility, psychoeducation aims to help patients express their fears, fantasies, and concerns, providing consistent support to both patients and the multidisciplinary team, minimizing the stress arising from conflicts related to choices, doubts, and uncertainties during the process. Psychoeducation plays a crucial role in supporting patients, fostering communication between partners, and offering strategies so they can adapt to the treatment and be prepared for accepting or not the technique used. Infertility and its treatments present opportunities for psychoeducation to be carried out from the initial consultation to the final result, as there are several moments where clarification, support, discussion, and reflection on various issues that arise along the way are necessary. In the context of infertility, the medical team and psychological support should prioritize the individual over the problem, enabling the identification of psychosocial aspects that act as causes and effects of the individual’s health and psychological conditions. Through psychoeducation, both for the professional team and the patients, there is space and time to look, listen, reflect, go beyond the problem, give it another meaning, and become informed about available options.

**Methods:** The reviewed articles were obtained from international databases: PubMed, Scientific Electronic and Library Online. The inclusion criteria were: literature published in national or foreign language, with the selection of 6 eligible bibliographies for the study, which aimed to clarify the importance of psychoeducational work in the complex context of assisted reproduction.

**Results:** The results of this study are organized based on research articles related to the topic, exploring experiences and emotional coping mechanisms related to parenthood, aiming to dissociate the conscious demand to have a child, and the unconscious desire that influences subjective production, manifesting in the desire for parenthood and its implications.

Conclusion: It is evident that the benefits of psychoeducation extend beyond commitment to treatment, also encompassing the reduction of anxiety and a better understanding of the choice to have children through assisted reproduction, which suggests that psychosocial interventions promote reflection, favoring the reinterpretation of certain beliefs and enhancing the coping process amidst the difficulties experienced during treatment.

**P-87. Efficacy and safety of ovarian stem cell transplantation in patients with primary ovarian insufficiency and poor ovarian response: a systematic review**

**Genifer Cristiane Freitas^1^, Hilton J P Cardim1, Janaina Favaro Soares^2^, Jordana Andrade Santos^3^, Benedito R S Neto^3^**

*^1^ Fertclinica - Clínica de Reprodução Humana - Maringá - PR - Brasil.*

*^2^ UEM - Maringá - PR - Brasil.*

*^3^ Universidade Federal de Goiás - Goiania - GO - Brasil.*

**Objective:** The aim of this study was to conduct a systematic review to assess the effectiveness and safety of mesenchymal stem cell transplantation as a therapeutic approach for patients with reduced ovarian function or poor response to infertility treatments.

**Methods:** A systematic review was performed, encompassing studies published within the past decade (from January 2012 to June 2022). PubMed, Cochrane Library, Scopus, and the National Library of Medicine databases were utilized as primary sources. The PRISMA Statement, 2020, was followed for the checklist of materials and methods. The risk of bias in the studies was evaluated using the Cochrane Risk of Bias (RoB) 2.0 tool, with assessments carried out independently by two reviewers. The systematic review was registered on the Prospero platform with the identifier number CRD42022354259.

**Results:** Fifteen studies that met the selection criteria were within the scope of this review. These studies assessed stem cell transplantation in patients with diminished ovarian function or poor ovarian response. Data were summarized and organized in two tables according to study design, degree of ovarian failure, source of stem cells, route of administration and dose applied, follow-up and other criteria.

**Conclusion:** Based on the analysis of studies that fulfilled the inclusion criteria, it can be inferred that there is a notable scarcity of randomized trials with substantial patient cohorts. Additionally, there was an absence of studies evaluating the safety of long-term therapy proposed by ovarian stem cell transplantation. Considering the obtained results, ovarian stem cell transplantation holds promise as a potential therapeutic approach for this group of patients, who currently lack established treatment options.

**P-88. Evaluation of Seminal Parameters According to WHO 2021 Guideline in an Obese Population**

**Camila Antunes Domingues^1^, Nicoly Abido Borilli^1^, Helena Vitoria Fauth^1^, Iáskara Vieira Oliveira^1^**

*^1^ Gerarte - Passo Fundo - RS - Brasil.*

**Objective:** Infertility can occur due to various factors, and currently, obesity has become a serious problem when it comes to fertility. In men, it causes alterations in the levels of crucial sex hormones. It increases estradiol levels and reduces testosterone levels, which may be related to the accumulation of adipose tissue. This can potentially compromise sperm production. The general objective of this study was to determine if semen parameters, according to the WHO 2021 guidelines, are negatively affected by obesity in a subset of individuals

**Methods:** For the collection of semen samples, participants underwent a brief questionnaire, containing information about age, weight, height, and some questions about daily habits, such as drug and anabolic steroid use. The samples were collected through masturbation in a specific and private room using a disposable and sterile plastic container. Patients were sexually abstinent for two to three days before the collection. The analysis was performed through a semen analysis, following the criteria set by the World Health Organization (2021). The analysis included the use of a Micrometric Reticule to evaluate sperm size, macroscopic analysis of the sample (color, volume, liquefaction, viscosity, and pH), and microscopic analysis of the sample (concentration, motility, morphology, and vitality). They were divides into three groups: gruop 1 with normal BMI, group 2 with overweight BMI, and group 3 with obese BMI.

**Results:** The study found a negative relationship in the value of morphology (*p*=0.008) in group 3, which consisted of individuals classified as obese. This suggests a decrease in sperm quality in obese patients. The results did not show a relationship between BMI and the rest of the evaluated semen parameters.

**Conclusion:** In conclusion, the study indicates that BMI is related to a decrease in normal morphology in individuals with a BMI above the ideal range for their height, specifically, obese individuals in the studied population. The other semen parameters did not show significant differences between the groups.

**P-89. The importance of Key Performance Indicators (KPI's) for tracking deviations in results**

**Lucileine Keico Nishikawa^1^, Jhuly Laurentino Nunes^1^, Ianaê Ichikawa Ceschin^1^, Alvaro Pigatto Ceschin^1^, Onel Alejandro Amaya Goitia^1^**

*^1^ Feliccità Instituto de Fertilidade - Curitiba - PR - Brasil.*

**Objective:** To demonstrate the importance of using and analyzing KPI's, in order to identifying possible problems that could affect laboratory rates.

**Methods:** The KPI's used to evaluate the laboratory results were selected based on the Vienna Consensus, namely: fertilization rate >65%, D3 cleavage 45%, blastocyst development >40% and implantation >35%. Based on the laboratory routine, the analysis of the results is carried out monthly. In a retrospective evaluation, a decrease in the rate of evolution to Blastocyst was evidenced, so that, according to the quality parameter of the center, a non-compliance was generated. Some of the following interferences were included: laboratory environment, operator, incubator, disposable materials, mineral oil and culture medium

**Results:** Regarding the analysis of the laboratory environment, the air certification parameters, temperature and humidity were satisfactory. From operator analysis, the rates of fertilization and embryo manipulation did not demonstrated variations in rates between operators. There was no difference between the incubators used for embryos culture, as well there was no exchange of gas cylinders used. Disposable materials and mineral oil did not change. Regarding the traceability of the culture medium, it were evidenced that the type of medium was changed, from sequential to single, both being from the same manufacturer and having a pH value within the recommendation. This was the only modification made in this period. Corrective action of the culture medium was carried out with reestablishment of blastulation results in the following month.

**Conclusion:** Currently, there are several culture medium available on the market, with scientifically satisfactory results, however, it depends on each laboratory to assess which one best supports embryologic development within its reality. Unforeseen circumstances in the laboratory routine occur, so that alternatives need to be taken quickly. Therefore, it is important to maintain strict quality control with properly documented materials and processes. Quality indicators, combined with good quality management, make it possible to quantify, monitor and constantly improve results. These are essential for patient safety and laboratory autonomy, allowing early detection of problems and the adoption of corrective actions. Any variable, no matter how small, will be observed and quickly controlled, avoiding clinical impact.

**P-90. The Clinical Effectiveness of Pre Implantation Genetic Test (PGTA) in Assisted Reproductive Technology**

**Edilberto Araujo Filho^1^, Leonardo Previato Araujo^1^, Bruna Ribeiro Gobi^1^, Kelly Colussi Pinheiro Precipito^1^, Leticia Alfo Rolim^1^, Ligia Fernanda Previato^1^**

*^1^ Centro de Reprodução Humana de S J Rio Preto - São José do Rio Preto - SP - Brasil.*

**Objective:** to evaluate the impact of PGTA in the clinical pregnancy rate, implantation rate and live birth rate on *In Vitro* Fertilization (IVF)

**Methods:** This is an observational, retrospective cohort study. Sixty patients submitted to Control Ovarian Hyperstimulation for IVF and PGTA in the period of 2020 to 2022 in a private clinic were analized. Patients age ranging from 34 to 44 years (media: 39). All patients used antagonist protocol with recombinant FSH and Human Menopausal gonadotrophin for ovulation induction and recombinant HCG for oocyte maturation. Oocyte aspiration was performed 36 hours after HCG guided by endovaginal ultrasound .The embryos were cultured to blastocist stage and biopsy was performed in the 5th or 6 th day of culture an All the biopses were sent to the same Lab (DASA/Chromosome) and the technique used to run the DNA was Next Generation Sequencing (NGS). The indications for PGTA were: Advanced maternal AGE (AMA),Habitual Abortion (HA), Implantation Failure (IF), Chromosome Abnormality (CA), Severe Male Factor or Personal Reasons.

**Results:** Thirty one patients underwent IVF/PGTA due to Advanced Maternal Age (51,7%), 04 due to Habitual Abortion (6,7%), 06 due to IF (10%), 04 due to Chromosome Abnormality (6.7%), 07 for Severe Male Factor (11.7%) and 08 due to personal reasons (13.3%). Thirty out of 60 patients (50%) had euploid embryos to transfer. Twenty four of them transferred their embryos: 23 of them transferred only one euploid embryo and one patient decided to transfer two euploid embryos. Twenty one had a positive BhCG (87.5%), but one was biochemical and 03/21 patients had a clinical abortion (14.3% abortion rate). Our clinical pregnancy rate was: 83% (20/24) and our implantation rate was 88%. Seventeen patients out of 24 transfers delivered 18 healthy babies (71% take home baby rate), one of these patients had twins (multiple gestation rate: 4.3%). Between patients with euploid embryos, 23.3% (7/30) were ≥ 40 years of age and 76.7% had < 40 years of age.

**Conclusion:** Implantation Genetic Test for Aneuploidy improved the pregnancy rate, implantation rate and take home baby per embryo transfer in this particular group of patients.

- The percentage of euploid embryo obtained in patients of ≥ 40 years is very low and this population of patients should be aware of it and think about egg donation

- PGTA decreased multiple gestation rate by selecting the best embryo to transfer without affecting the chance per transfer, what gives more security for patients to decide toward Single Embryo Transfer (SET).

**P-91. Multidisciplinary Care in Sex Reassignment**

**Helena Prado Lopes^1^, Flávia Giacon^2^, Karen de Marca^3^**

*^1^ Pró-Fértil Centro de Medicina Reprodutiva / Instituto de Ginecologia da Universidade Federal do Rio de Janeiro - UFRJ - Rio de Janeiro - RJ - Brasil.*

*^2^ Mater Prime Clínica de Reprodução Humana - São Paulo - SP - Brasil.*

*^3^ IEDE - Instituto Estadual de Diabetes e Endocrinologia - Rio de Janeiro - RJ - Brasil.*

**Objective:** This study aims to highlight the importance of multidisciplinary care for transgender persons, drawing attention to the psychological support throughout the sex reassignment process. During transition stages, individuals may be offered hormone therapy and surgical procedure to induce body changes. Hormone therapy involves the administration of hormones to help develop physical characteristics that align with the person’s gender identity. Testosterone is used to stimulate clitoral growth and body hair growth, suppress menstrual cycles, and deepen the voice in the female-to-male transition. Conversely, estrogen is used to trigger the development of feminine traits, usually combined with an anti-androgen to inhibit hair growth, particularly facial hair, spontaneous erections, and to promote breast development. Testicular atrophy with a reduction in sperm volume is common. The impact of this type of intervention on fertility and its management is a subject of ongoing research.

**Methods:** The methods used to conduct this study were a literature review and data collection from previous research and articles related to the gender transition process. Specially, a bibliographic search was conducted using the keywords “transgender person” and “sex reassignment” in the following international databases: PubMed, Scientific Electronic Library Online. The inclusion criteria for this study consisted of literature published in national or foreign language. Following these criteria, six eligible bibliographies were selected for the research.

**Results:** Currently, the increased inclusion and attention to the healthcare needs of transgender persons have led to a growing desire to form families in a new context. Transgender men and women now have access to reproduction methods, and healthcare professionals must be well-informed about this dynamic, gender transition therapies available, and their impact on fertility in order to provide them with better guidance throughout the decision-making process. Regarding fertility preservation, healthcare professionals should advise patients to cryopreserve eggs, sperm, embryos, ovarian tissue, or testicular tissue before starting hormone therapy or undergoing definitive procedures such as the removal of ovaries or testicles, preserving the possibility of future parenthood. Our findings emphasize interdisciplinarity as a promising approach for all the people involved, including professionals and, most importantly, patients. We highlight the importance of the work of mental health professionals within the team to raise awareness among its members so that access to healthcare for this population is offered and provided meeting their specific demands.

**Conclusion:** The establishment of collaborative efforts among the multidisciplinary team, which should include mental health professionals, is essential, aiming to provide meticulous and supportive care that meets the needs and demands of the transgender population throughout the sex reassignment process. It is important that health care professionals are able to provide general advice on the proper use of hormones and closely monitor the treatment process. By doing so, this team will be able to provide both medical and emotional support, thus leading to a reduction in the occurrence of adverse events. This approach is essential for promoting the general well-being of this population.

**P-92. Embryo secretome is essential for endometrium remodelling during the window of implantation: in vitro analyzes of several cytokines**

**Tatiana Carvalho de Souza Bonetti^1^, Luciana Le Sueur-Maluf^2^, Adriana Almeida Bassani^1^, Giovana Aparecida Gonçalves Vidotti^3^, Ana Paula Girol^3^, Melina Mizusaki Iyomasa Pilon^3^, Alfeu Cornelio Accorsi Neto^3^, Eduardo Leme Alves Motta^1^**

*^1^ Departamento Ginecologia - Escola Paulista de Medicina - Universidade Federal de São Paulo (EPM-UNIFESP) - São Paulo - SP - Brasil.*

*^2^ Departamento de Biociências - Universidade Federal de São Paulo (UNIFESP) - Campus Baixada Santista - Santos - SP - Brasil.*

*^3^ Centro Universitário Padre Albino - UNIFIPA - Catanduva - SP - Brasil.*

**Objective:** Successful embryo implantation is paramount in medically assisted reproduction. Defects in the endometrium have been associated with reproductive disorders, may cause implantation failures and these anomalies have been identified as one of the top research priorities in reproductive field. However, given the numerous endometrial functions that collectively allow the embryo to attach, the short period to implant and ethical considerations, makes difficult the full understanding of the molecular network that safeguards the embryo-endometrium crosstalking. This study aimed to evaluate the influence on the embryo secretome in modelling endometrium in vitro, possibly representing the embryo-endometrium crosstalking during the implantation window.

**Methods:** Endometrium biopsies were collected from fertile women during gynecologic routine procedures after consenting term signature between July-December 2022. Tissue biopsies were digested, and primary endometrial stromal cell cultures (EC) were established as described by D'Amora et al. 2009, with minor modifications. Briefly, cells were seeded onto 12-well plates at a density of 5,000 cells/cm2 and cultured for 48h (70% confluence). EC were hormone primed with estrogen (E2; 100 nmol/L for 24h), progesterone (P4; 100 nmol/L for 24h), mimicking the window of implantation. A pool of conditioned embryo culture medium (CEmbCM) collected from IVF cycles was prepared. E2/P4-primed EC were stimulated with CEmbCM in a volume of 20% of total culture media and then maintained for 24h. As controls, EC were grown in the following conditions: (i) Control: no E2/P4-priming or CEmbCM-stimulation; (ii) Hormone: E2/P4-priming and no CEmbCM-stimulation; (iii) CEmbCM: no E2/P4-priming and with CEmbCM-stimulation. Concentration of molecular factors (EGF, FGF-2, FRACTALKIN, GM-CSF, IL-6, IL-8, IL-15, IL-18, IP-10, TNF-A, VEGF-A, and LIF) were detected in the EC supernatant by a magnetic bead-based assay (MAGPIX^®^ System, Luminex). Experimental procedures used three biological replicates, and experiments were performed in triplicates.

**Results:** A two-way Generalized Linear Model (GzLM) was used to evaluate the effect of the E2/P4-priming, CEmbCM-stimulation, considering each factor independently and the interaction between them (experimental condition), on secretion of the molecular factors by the EC. Statistical interaction is defined as the combined effects of factors on a measure of interest (dependent variable). If an interaction effect is present, it means that the impact of one factor depends on the other factor. An interaction between the variables was detected, showing a synergistic effect of E2/P4-priming and CEmbCM-stimulation on EC, resulting in a significant increase of molecular factors secretion, compared to control (mean±standard error): FGF-2 (2731.6±526.8 vs. 80.1±1.8; *p*<0.001), GMC-SF (54.4±3.6 vs. 33.1±3.8; *p*<0.001), IL-6 (12691.7±5319,4 vs. 13.7±5.3; *p*<0.001), IL-8 (2145.8±255.0 vs. 8.3±3.4; *p*<0.001), IL-18 (24.7±0.9 vs. 15.3±0.5; *p*=0.027), IP10 (60.7±2.6 vs. 51.8±1.9; *p*=0.016), TNF-a (15.8±0.4 vs. 11.6±0.3; *p*=0.019), VEGF-a (164.6±6.3 vs. 39.7±6.7; *p*<0.001) and LIF (87.6±5.2 vs. 28.7±0.2; *p*<0.001).

**Conclusion:** E2/P4-primed EC strongly responded to embryo secretome stimulation producing cytokines and growth factors, clearly demonstrating the crosstalk between embryo-endometrium. The factors secreted by the EC may participate in the endometrium receptivity and/or the subsequent implantation process. Our results reinforce that human embryo implantation mainly requires the interaction between the endometrium and the blastocyst secretome to achieve a successful pregnancy. Also this line of research intends to test other conditions, such as infertile patients presenting implantation failure, to assess how EC could be remodeled upon embryo stimulation.

**P-93. The impact of the nursing team's work on patients’ evaluations undergoing in vitro fertilization treatment**

**Bruna Mercante da Silva^1^, Patricia de Castro Gonçalves^1^, Diana Caroline da Silva Bastos^1^, Eliana Junko Morita Yokota^1^**

*^1^ CITI Hinode - São Paulo - SP - Brasil.*

**Objective:** The objective of the study seeks to comprehend patients perception and satisfaction regarding the treatment received, with a focus on the nursing team’s performance, through the analysis of Google reviews, where specific keywords were investigated.

**Methods:** The study was based on 302 patient reviews on the Google platform about the CITI HINODE clinic - Centro de Investigação e Importação da Infertilidade, São Paulo. The inclusion criteria were the reviews containing comments written text, totaling 238 analyzed comments. The analysis was conducted by identifying patterns and trends related to patient satisfaction, as well as possible areas for improvement in the care and treatment quality. The research focused on identifying the most mentioned words. As well as pre-defined words considered important to assess the nursing team’s performance, such as: team, nursing, care, competence, kindness, professionals, human and trust. All comments made on the platform were transferred to a Word document, which allowed for word identification and counting.

**Results:** The analysis showed that the most mentioned word was “team” with 151 citations, present in 63% of the comments. The second most cited word was “dream” with 125 citations, present in 52% of the comments. We also evaluated the prioritized words during the treatment: "kindness" was mentioned in 34% of the evaluations, "professionals" in 29%, "human" in 15%, "care" in 9%, "competence" in 6% and " trust” by 7%. Regarding the nursing professionals, there were 8.3% of the citations, with 5% found through the Search term “nursing” and 3.3% mentioning the names of the nursing staff members.

**Conclusion:** The study highlights the significant impact that the nursing team’s work has on the patients' journey during in vitro fertilization treatment. The emotional support provided by the nursing team, along with safe and qualified care, helps patients deal with the emotional challenges associated with treatment, strengthening their confidence and security throughout the process. The findings reflect the ongoing importance of investing in a highly qualified nursing team, attentive to the emotional needs of patients, providing exceptional care and support throughout the in vitro fertilization treatment process.

The results demonstrate how the patient's feedback on Google reviews can be studied and utilized to continuously improve services, ensure more personalized care, and enhance the overall patient experience in human reproduction treatment.

**P-94. The plasticity of natural cycle for frozen embryo transfer: more friendly than expected**

**Marcelo Ferreira^1^, Gabriella Mamede Andrade^1^, Nilo Frantz^1^**

*^1^ Nilo Frantz Medicina Reprodutiva - Porto Alegre - RS - Brasil.*

**Objective:** There is a growing interest in frozen embryo transfer using the natural cycle for endometrial preparation. However, there is little information available to support clinician in the daily practice of this protocol. Eventually, a cycle is suspended or leaves the clinician unsure if it differs from “ideal” physiology or had laboratory monitoring and ultrasound scans below the desirable. Herein, the study objective is to describe natural cycles that resulted in deliveries or ongoing pregnancies to assess the main variables with implications in clinical practice.

**Methods:** Natural cycles that resulted in live births or ongoing pregnancy (from 20 weeks) were retrospectively evaluated from January 2018 to December 2022. All transfers were performed with good quality embryos according to Gardner et al. (2000) classification. Variables analyzed on LH peak day were: the cycle day, LH dosage, E2 dosage, follicle size and endometrial thickness. Regarding transfer, the variables evaluated were: number of days after LH peak, cycle day and weekday. The total number of ultrasound scans after baseline screening was also tabulated. The LH surge day was determined based on hormone dosage (above 15 or twice as the previous day) or on day before the CL identification based on ultrasound scan. The additional luteal supports were made using 400mg intravaginal micronized progesterone from 4 to 5 days before embryo transfer.

**Results:** Among the 143 natural cycles that resulted in live births or ongoing pregnancy, 8 had no records and were removed from the study. From the remaining 135 cycles, 128 (94.8%) were performed without inducers and/or triggers (true natural cycles) and had the data analyzed. In 65.6% of cycles the LH surge was determined based on LH dosage and in 34,4% on CL identification. The LH surge day vary from 8 to 23 of cycle. In 71.3% of cases the LH surge occur between days 11-16 but in 18%, from day 17 to 20. Follicle size on the day of ovulation was different from 18 to 21mm in 32.4% of cycles. The LH and E2 most common dosage on the surge day had variance from 41 to 60 µUI/ml and from 200 to 300pg/ml respectively. In 81.7% of cycles the endometrium thickness was higher than 7mm. Regarding the transfer day, 77% of cycles the transfer was between days 17 and 23. In 76.9% of cycles the transfer was after LH+6 days and 20.7% on LH+7 and the remaining 2.5% of cycles the transfer was on days LH+4/LH+5; none of the cycles had a transfer on Sunday. Finally, the number of ultrasound scans varied from 1 to 10 and in the half of the cases performed 4 or 5 times.

**Conclusion:** The data highlights the heterogeneity of pure natural cycle markers for endometrial preparation in successful cycles of frozen-thawed embryo transfer. Although it has expected findings in standard natural cycle in relation to the day of the LH peak, LH and estradiol values, follicle size and implantation window; cycles that differs from that are acceptable. This result brings important information on biological variations on natural cycle that can result in live births. Showing that even with the difficulties of the number of ultrasounds and desirable hormone dosages, it is possible to manage a natural cycle with very satisfactory results. Importantly, this plasticity of the pure natural cycle demonstrated by transfers on day LH+6 and LH+7 possibilities a more friendly cycle for the team avoiding weekend transfer.

**P-95. Case report: use of pentoxifylline in frozen samples of patients with asthenozoospermia, who had undergone Microdissection Testicular Sperm Extraction (MicroTESE), resulting in clinic pregnancy**

**Brenda Campos Villa^1^, Thais Tiemi Higa^1^, Mariana Moraes Piccolomini^1^, Oscar Barbosa Duarte Filho^2^, Lucas Yugo Shiguehara Yamakami^2^, Renato Bussadori Tomioka^2^**

*^1^ Lab For Life - SP - SP - Brasil.*

*^2^ Vida Bem Vinda - SP - SP - Brasil.*

**Objective:** The objective of this case report is evidencing the effect of pentoxifylline in spermatic parameters of frozen MicroTESE samples and report the final results of In vitro fertilization (IVF) cycles treatment.

**Methods:** It is known that semen cryopreservation and thawing can cause decrease in motility and reduce potential of sperm fertilization. Thus, various substances had been tested to increase sperm resistance to cryopreservation process, for example, pentoxifylline. This substance is a methylxanthine derivate and a phosphodiesterase inhibitor, use to promote sperm motility.

For this case report, two IVF cycles treatment were selected, and were performed on different days in LabForLife laboratory; frozen samples from MicroTESE were used. The patients were 34 and 45 years old and their partners were 33 and 28 years old, respectively. The samples were frozen after the urological procedure and microscopic evaluation. Freezing Medium TYB (Irvine) was used, following the manufacturer's protocol, and thawing was performed on the day of oocyte retrieval. After thawing the sample, an evaluation of sperm concentration and motility was performed.

After the initial evaluation, the entire thawed volume was washed, and a new analysis were performed. In both samples, the absence of motility was verified, therefore, pentoxifylline reagent (Vitromed) was used following the manufacturer's protocol. After a period of 10 minutes of incubation, the analysis was performed in an inverted microscope and progressive motile spermatozoa were found in the samples. After selecting the best sperm, intracytoplasmic sperm injection (ICSI) was performed in the patient's oocytes.

**Results:** In the first case analyzed, 20 oocytes were injected with progressive motile spermatozoa, resulting in 8 blastocysts (6AA, 6AA, 5AA, 5AA, 4BA, 3AA, 3AA, 3BB), 5 of which were euploid. Transfers of a single embryo were performed on two separate dates. The 4BA blastocyst transfer resulted in a negative beta HCG; and the second transfer, a positive beta HCG and 1 gestational sac were obtained from a 5AA blastocyst. In the second case, 12 oocytes were injected, also with progressive spermatozoa, which resulted in 3 blastocysts (5AA, 4CB,3BC), 2 of which were euploid. A transfer of a 5AA blastocyst resulted in a positive HCG beta and 1 gestational sac. There is still no information on live births for both cases so far.

**Conclusion:** Regardless of the impacts of cryopreservation on sperm parameters, the use of pentoxifylline increased the rate of progressive motility, enabling the selection of better quality sperm in samples where initially only immobile sperm had been observed. In this case report, an improvement in the parameter of sperm motility was observed after the use of pentoxifylline, no other effect that it could cause on sperm was studied.

**P-96. The importance of Assisted Reproduction Technology beyond the knowledge of midwife nursing**

**Jeniffer Monteiro Portes de Lima^1^, Helen Patricia Camargo Kanhouche^1^, Luciana da Conceição Melo^1^, Jaqueline Sousa Leite^1^, Grazielle Viola Arronqui^1^**

*^1^ Universidade Santo Amaro - São Paulo - SP - Brasil.*

**Objective:** To characterize the importance of theoretical and scientific knowledge in nursing care for Assisted Reproductive Technology, promoting appropriate care and reducing unfamiliarity among nurse-midwives.

**Methods:** This study involves an integrative review that was conducted by taking the question "What is the importance of the knowledge of Assisted Reproductive Technology beyond Midwife Nursing?”. The review followed the PICOs strategy: P (participants): the studies examined; I (intervention): the approach to Assisted Reproductive Technology content; C (comparison): the postgraduate students; and O (outcome): the importance of the content described in this specific literature. The search was conducted by looking into the databases PubMed, Medline, Lilacs and Scielo, from 2013 to 2023, using the related descriptors “Assisted Reproductive Technology” and “Nursing”. The limitations presented in the search were the scarcity of studies that address the topic in question. Initially, 32 articles were found using the provided descriptors. The inclusion criteria were original articles available in English, Portuguese, and Spanish. Each article was evaluated by two reviewers. Subsequently, 11 articles were excluded because they were not aligned with the objective or were duplicates. In a second analysis, a full reading of 21 articles was conducted, and 8 of them which were relevant to the theme and objective of the study in question were selected.

**Results:** Assisted Reproductive Technology (ART) is a set of techniques, procedures, and biotechnological interventions on the human reproductive process^1^. Since the first birth through ART in 1978 in England, the medical scientific community has been sharing knowledge in pursuit of improving in this field. In Brazil, this milestone occurred in 1984 when the first successful case was achieved, resulting in the birth of a baby through ART^2^. The historical milestone for nursing in ART in Brazil happened in the 1990s, when guidance for infertile couples was provided by nurse-midwives, professionals who occupied this role without formal training for this purpose^3^. In recent years, the number of Assisted Human Reproduction Centers (AHRC) has been increasing. Currently, there are 193 AHRCs in Brazil4, which demonstrates a growing and promising field in the job market for nursing. A qualitative study reports the importance of the nurse professional as part of the multidisciplinary team that provides care for the couples/individuals seeking to build families5. Several international studies corroborate the importance of nursing care throughout the journey of individuals undergoing ART^6,7,8,9,10,11^. Nursing professionals who perform both clinical and managerial roles in ART face the lack of specific support for their field of practice, such as a postgraduate course^1,5^. Considering that the best practices are based on evidence, access to high-quality information becomes something indispensable to drive professional growth. By staying informed with the latest research and evidence-based guidelines, nursing professionals can enhance their skills and competencies, providing optimal care and support to individuals undergoing ART. This continuous pursuit of knowledge and adherence to evidence-based practices contribute significantly to the overall success and positive outcomes in this field.

**Conclusion:** To achieve excellent, humane, and scientifically grounded care, it is imperative that institutions promote specialized technical training programs for nursing professionals. Some postgraduate courses in nursing midwife currently cover the content of ART. However, to this date, there are no records of specific postgraduate courses in ART registered with the Ministry of Education and Culture and validated by the Federal Nursing Council. It is crucial that initiatives are developed in this direction, aiming to enhance the qualification of nurses in ART. Only then, we can ensure healthcare of high standards that are fully tailored to the needs of the scenario.

**P-97. Comparative analysis of the incidence of frozen embryos in the last three years by country region and the northeast region**

**Gilberta Guadalupe de Souza Santos^1^, Pollyanna Andreza Ribeiro dos Santos^2^, Camilla Karinne Guimarães Rosa^1^, Larissa Chaves Medeiros^3^, Cley Gabriel Lima Carvalho Dantas^4^, Fabricia Correia de Azevedo^4^, José Edeilson Dias Vitorino^4^, Gabriel Silva Morato^4^, Yasmin Casado Fortunato^4^, Héllen Néo da Rocha^4^, Emerson Santana Santos^4^**

*^1^ Universidade Federal de Sergipe - Aracaju - SE - Brasil.*

*^2^ Hospital das Clínicas da UFMG - Belo Horizonte - MG - Brasil.*

*^3^ UNIFASC - Salvador - BA - Brasil.*

*^4^ Universidade Federal de Sergipe - Lagarto - SE - Brasil.*

**Objective:** To determine the incidence of frozen embryos in the last three years by region of the country and to analyse such incidence on the northeast region.

**Methods:** Descriptive, cross-sectional and quantitative study using secondary data obtained by the National Embryon Production System. The target population were women that donated embryos in all regions of Brazil, regardless of the maternal age group, in the period from Jan/2020 to December 2022. The data obtained were reorganized and analyzed using simple descriptive statistics.

**Results:** During the year 2020 the total frozen embryos were 85,448. In the northern region there were 1,235, northeast 8,642, central-west 5,709, southeast 59,777 and south 10.085. In 2021, the total number were 108,469, with the following values for each region: North 1.084, Northeast 12.543, Central-West 8.009, South-East 73,947 and South 12.916. In the year 2022, the total number were 103,931 with the number of embryos in the North region 1,106, the Northeast 10,467, the Midwest 7,940, the Southeast 71,966 and the South 12,452. In 2021, there was a 45.41% increase in the number of frozen embryos in the northeastern region compared to the previous year. Nonetheless in 2022, there was a drop of 16.55% compared to the previous year. The states that concentrated the most actions were Bahia, Pernambuco and Ceará.

**Conclusion:** In recent decades, there has been a 255% increase in the number of frozen embryos in Brazil (SisEmbrio, 2022). This increase is largely due to the growth of assisted reproduction with improved techniques and the feasibility of access (SBRA, 2022). Our survey is corroborated by the fact that in 2021 there was a 45% increase in the number of frozen embryos in the northeastern region compared to the previous year. Meanwhile, it points to a 16% drop in 2022, compared to the previous year. Despite this regression, the rate of frozen embryos in 2022 in the northeastern region was 21% higher than 2020. As the study covers the years of the COVID-19 pandemic, which may justify lower rates. Nevertheless, the Northeast accounts for 10.62% of the national frozen embryos scenario and it is worth noting its advance in this scenario.

**P-98. Myo-inositol in follicular fluid - Oocyte quality and fertilization rate in patients undergoing In Vitro Fertilization/Intracytoplasmic Sperm Injection (IVF/ICSI)**

**Maria Carmo Borges Souza^1^, Marcelo Marinho Souza^1^, Roberto Azevedo Antunes^1^, Ana Cristina Allemand Mancebo^1^, Patricia Cristina Fernandes Areas^1^, Layna Almeida Barbosa Silva^1^, Veronica Almeida Raupp^1^, Ana Luisa Marinho Souza^1^, Flavia Fernandes Sequeira^1^, Diana Caroline Silva Bastos^2^, Tania Maria Ortiga-Carvalho^2^**

*^1^ Fertipraxis Centro de Reprodução Humana - Rio de Janeiro - RJ - Brasil.*

*^2^ Laboratorio de Endocrinologia Molecular e Translacional - Instituto de Biofisica -Carlos Chagas Filho - Rio de Janeiro - RJ - Brasil.*

**Objective:** To evaluate a possible correlation between Myo-inositol (MI) in follicular fluid (FF) and the quality of recovered oocytes/fertilization rates in patients undergoing IVF/ICSI as the follicular microenvironment is an important determinant of oocyte development. MI is an isomer of a C6 alcohol that belongs to the vitamin B complex group. Previous studies support the notion that it is involved in the regulation of the release of Ca2+, related to gamete development including oocyte maturation. The growth and development of oocytes requires communication between the granulosa cells and the FF. Thus, changes in this environment can negatively impact oocyte maturation and reproductive potential.

**Methods:** Prospective study carried out from June 2018 to April 2019, including 35 infertile women aged between 30 and 40 years, using MI for at least 8 weeks, undergoing IVF/ICSI. At the time of oocyte puncture, the FF of the first ≥ 18 mm follicle with oocyte recovered was separated to determine the MI concentration (spectrophotometric analysis). All patients received similar stimulus protocol, namely deltafollitropin or menotropin (Rekovelle^®^ or Menopur^®^, Ferring Pharmaceuticals) in an individualized SC-dose. Clinical decision led to a GnRH-antagonist (Cetrorelix acetate, Cetrotide^®^, Merck) 0,25 mg/d initiated in a flexible schedule (one follicle ≥14 mm) continued throughout the stimulation period or Dydrogesterone 10mg 8/8 hours (Duphaston^®^, Abbott) from the beginning of stimulation until the day after the trigger. Oocyte retrieval took place 36 hours after trigging. Four generalized linear models were run to test the impact of MI on the following variables: number of metaphase II (MII) oocytes, fertilization rate, recovered oocytes and follicles ≥ 15 mm on the hCG-day. The analyzes were controlled by AMH, age, BMI, and AFC, using SPSS Windows version 23 software.

**Results:** No relationships were identified between MI and the variables follicles ≥ 15 mm, number of oocytes retrieved and fertilization rate while in model 1, both the general Chi-square and the variable Inositol demonstrated predictive value for the number of recovered MII (Table1). Interestingly, the analysis of coefficient B shows an increase of 0.03 points in MII for each increase of 1 point in Inositol.

Limitations. To compensate weakness of the sample size, resampling procedures (bootstrapping) were implemented to produce greater reliability of the results and present a confidence interval (CI) of 99% for Exp B3.

**Conclusion:** Myo-inositol in follicular fluid correlated with better metaphase II oocyte rates.

**References.**

Chiu et al. Follicular fluid and Serum concentrations of myo-inositol in patients undergoing IVF: relationship with oocyte quality. Hum Reprod.2002; 17:1591-6.

Novielli et al. Effects of ά-lipoic acid and myo-inositol supplementation on the oocyte environment of infertile obese women: a preliminary study. Reprod Biology.2020;20: 541-6.

Haukoos & Lewis. Advanced Statistics: Bootstrapping Confidence Intervals for Statistics with ‘‘Difficult’’ Distributions. Acad Emerg Med.2005; 12:360-5.

**Table 1:** Generalized linear models.

Outcomes	predictors	B	Exp(B)	95% C.I.to Exp(B)		*Chi-square of Wald*	p	Chi-square (gl)	p
				Inf	Sup				
MII	(constant)	-1.52	0.21	0.01	8.94	0.64	.421	25.77(5)	.000
	**Inositol**	**0.03**	**1.03**	**1.01**	**1.06**	**6.13**	**.013**		
Fertilization rate	(constant)	7.85	4.26	1.01	2.28	3.85	.050	17.86(5)	.003
	Inositol	-0.01	0.99	0.94	1.04	0.06	.795		
Retrieved oocytes	(constant)	-3.07	0.04	0.00	3.03	2.07	.150	26.06(5)	.000
	Inositol	0.02	1.02	0.99	1.05	2.83	.092		
Follicles ≥15mm	(constant)	-1.69	0.18	0.02	1.14	3.29	.070	49.93(5)	.000
	Inositol	-0.01	0.99	0.98	1.01	0.94	.330		

Note: Models were controlled by AMH, age, BMI and AFC.

Chi-square of the model likelihood ratio. p: statistical significance.

**P-99. Comparison of the efficacy of different hormonal triggers in controlled ovarian stimulation**

**Jonathan Boncristiano^1^, Bernardo Lamonier Moura^2^, Rayssa Chaves Alvarenga^3^, Emilio Costa Garavini^4^**

*^1^ Gerar in Vitro - Itabuna - BA - Brasil.*

*^2^ Gerar in Vitro - Belo Horizonte - MG - Brasil.*

*^3^ Gerar in Vitro - Ipatinga - BA - Brasil.*

*^4^ Gerar in Vitro - Ponte Nova - MG - Brasil.*

**Objective:** The objective of this study is to compare the oocyte retrieval rate from observed follicles, the number of obtained oocytes, and their maturation between two medications used as ovulation triggers: the GnRH agonist Acetate Triptorelin and the HCG Alfacoriogonadotropin.

**Methods:** For this retrospective study, we analyzed 8,491 oocytes from 760 cycles of assisted reproductive treatment and oocyte cryopreservation conducted at four units of the Gerar InVitro Human Reproduction Institute from January 2021 to April 2023. All cycles with controlled ovarian stimulation were included and divided into three groups based on the trigger analyzed. The study group received either Alfacoriogonadotropin or Triptorelin acetate, while the control group included all triggers used. The first group aimed to analyze Alfacoriogonadotropin, considering parameters such as oocyte retrieval rate, average number of oocytes, and oocyte maturation rate. The second group aimed to analyze Triptorelin acetate, considering the same parameters. The third group served as the control group, encompassing all routine triggers and evaluating the mentioned parameters. Results from the Alfacoriogonadotropin and Triptorelin acetate groups were compared to the control group using the chi-square test.

**Results:** When comparing three groups, Alfacoriogonadotropin (group 1), Triptorelin acetate (group 2), and control, significant differences were observed. Group 1 showed no significant differences compared to the control in terms of oocyte retrieval rate, number of oocytes, and maturation. On the other hand, group 2 exhibited a higher retrieval rate (77.31% vs. 65.82% in control) and a greater average number of obtained oocytes (14.5 vs. 7.9 in control). However, there were no significant differences in oocyte maturation between group 2 and the control. These results suggest that Triptorelin acetate can be used in patients with a high number of follicles without affecting their maturation.

**Conclusion:** In conclusion, triptorelin acetate proves to be a promising option as an effective and safe trigger, without negatively affecting the proportion of mature oocytes in patients with a high number of follicles. However, further studies are needed to confirm these findings, explore underlying mechanisms, and consider individual patient factors.

**Table:** 

	Group 2		Group 3		p-value
	Triptorelin Acetate		Control group		
	N (%)	Standard Deviation	N (%)	Standard Deviation	
**Sample size**	333		133		
**Average age**	33,3	±5	36	±5	
**Recovery Rate**	77.31%	±41%	65.82%	±35.82%	0.002^**G2/G3^
**Average Oocyte Count**	14.5	±9.,8	7.9	±6.34	< 0.001^**G2/G3^
**Maturation Rate**	78.60%	±23.85%	79.23%	±19.46%	0.707^*G2/G3^

^**^ When comparing the average number of eggs collected and the recovery rate between group 2 and the control group, statistically significant differences were observed.

**Table:** 

	Group 1		Group 3		p-value
	Choriogonadotropin alfa		Control group		
	N (%)	Standard Deviation	N (%)	Standard Deviation	
**Sample size**	274		133		
**Average age**	37	±5	36	±5	
**Recovery Rate**	79.36%	±41%	65.82%	±35.82%	0.273 ^*^G1/G3
**Average Oocyte Count**	8.8	±9.7	7.9	±6.34	0.673 ^*^G1/G3
**Maturation Rate**	78.50%	±20.61%	79.23%	±19.46%	0.822 ^*^G1/G3

^*^ When we compared group 1 with the control group, no statistically significant differences were found.

**P-100. Fertilization and development potential of immature oocytes (MI) in hyperstimulated ovarian cycles subjected to intracytoplasmic sperm injection (ICSI)**

**Fernanda Cristina Souza Batista^1^, João Pedro Alves de Freitas^1^, Atila Sena Almeida^1^, Daniele Pinheiro de Freitas^1^, Erica Suzanne Soares Leal^1^, Valmira Bispo de Oliveira^1^, Rafaela Diniz Cunha^1^, Genevieve Marina Coelho^1^**

*^1^ IVI - Instituto Valenciano de Infertilidade, Salvador, BA, Brasil - Salvador - BA - Brasil.*

**Objective:** To evaluate the fertilization and development potential of immature oocytes (MI) and oocytes with late spontaneous maturation (MI/MII) obtained from controlled hyperstimulated ovarian cycles of patients undergoing intracytoplasmic sperm injection (ICSI).

**Methods:** A retrospective analysis of the embryonic development potential of immature oocytes (MI) and in vitro spontaneously matured oocytes (MI/MII) from fertilization to blastocyst stage was performed in 220 ovarian cycles between June 2022 and June 2023.The meiotic maturation of oocytes was assessed after cumulus removal by observing the presence or absence of the polar body, indicating the oocyte meiotic stage. The classification and separation of oocytes based on their maturity were performed, considering oocytes in metaphase I (MI) as immature ones - those that did not show polar body extrusion. However, during the ICSI procedure, MI oocytes were reanalyzed, and those that spontaneously showed late polar body extrusion were reclassified as MI/MII and all were injected. Cycles involving oocyte vitrification, ICSI cycles containing only completely mature metaphase II (MII) oocytes, or cycles with immature oocytes in the prophase stage (germinal vesicle - GV) were excluded from the study.

**Results:** Out of 2,228 oocytes obtained from a total of 220 retrieval cycles, 1,553 were MII oocytes, and 326 were excluded due to incompatible maturation with the study. Among the remaining oocytes, 252 (11.31%) were classified as immature oocytes (MI), and 97 (4.35%) were in vitro spontaneously matured oocytes (MI/MII). The fertilization rate for the total injected MI oocytes was 48.41% (122), and for MI/MII oocytes, it was 26.8% (26). Analyzing the embryonic development, MI oocytes achieved a blastocyst rate of 5.73% (7) with 42.85% (3) viable blastocysts, and the rate for MI/MII oocytes was 50% (13), of which 61.53% (8) completed *in vitro* development as viable embryos.

**Conclusion:** The study supports the reassessment of oocyte maturation initially considered immature during the ICSI procedure, highlighting the occurrence of this process after retrieval. The relatively low fertilization and blastocyst rates are related to the cytoplasmic immaturity of the oocytes, which may influence embryonic development. Better fertilization rates were observed in oocytes that remained MI until the ICSI procedure, but the best blastocyst rate was seen in oocytes that completed their maturation. Maintaining the ICSI procedure on immature oocytes is important to expand the pool of injectable oocytes available. This approach aims to increase the success chances of the technique, the oocyte's reproductive potential, and provide new perspectives to improve outcomes for patients facing conception challenges, especially those with low ovarian reserve. Further studies and research are needed to understand the underlying mechanisms of oocyte maturation and its effects on fertilization and embryonic development.

**P-101. Cycles with mosaic embryos: what can we learn?**

**Marília Körbes Rockenbach^1^, Carla Giovana Basso^2^, Gabriella Mamede Andrade^1^, Nilo Frantz^2^**

*^1^ Nilo Frantz Reproductive Medicine - Porto Alegre - RS - Brasil.*

*^2^ Nilo Frantz Reproductive Medicine - São Paulo - SP - Brasil.*

**Objective:** Advances in preimplantation genetic diagnosis techniques have allowed the detection of mosaic embryos. However, little is known about the factors influencing the occurrence of mosaicism in assisted reproduction cycles. Therefore, the objective of this study was to evaluate clinical and laboratory characteristics of cycles with mosaic embryo diagnosis through preimplantation genetic test for aneuploidies (PGT-A), comparing low-level and high-level mosaicism.

**Methods:** This retrospective study included all PGT-A cycles with embryos diagnosed as mosaic between January 2021 and March 2023 at Nilo Frantz Reproductive Medicine. Maternal and paternal age, fertilization rate, total and top-quality (BL1) blastocyst rate, as well as euploid (< 30% of aneuploid cells), aneuploid (> 70%) and mosaic (low-level: 30-50%; high-level: 50-70%) diagnosis were evaluated. In addition, two research groups (low-level mosaicism - LLM, and high-level mosaicism - HLM) were compared regarding blastocyst morphology (BL1 - AA, AB, or BA; BL2 - BB; and BL3 - AC, CA, BC, CB, or CC), blastocyst day (D5, D6, and D7), aneuploidy type (monosomy or trisomy), and chromosome type in mosaicism according to Denver classification (A - 156-249Mb; B - 102-145Mb; and C - 51-80Mb). Statistical analyses were performed in GraphPad Prism software (v. 6.01) through unpaired T test and Fisher’s exact test, considering *P*-values <0.05 statistically significant.

**Results:** Between the period analyzed, 67 blastocysts were diagnosed as mosaic embryos, corresponding to 4.6% of all embryos analyzed and 12.8% of all PGT-A cycles. Regarding the cycles with embryos diagnosed as mosaic, the mean maternal age was 35.02 and paternal age 38.9 years, the fertilization, total blastocyst, and top-quality blastocyst rates were 84.4%, 66.8%, and 44.8%, respectively; it was also observed that the euploid (30.3%), aneuploid (34.8%) and mosaic rates (34.9%) were similar in these cycles. Comparing LLM (n = 47) and HLM (n = 20) groups, no statistically significant differences were observed between maternal and paternal age, body mass index, anti-müllerian hormone, oocyte maturity rate, fertilization rate, morphology and day of the blastocyst, as well as chromosome type involved in mosaicism. However, it was demonstrated a higher proportion of monosomy mosaicism in HLM in comparison to LLM (60% and 29.79%, *p* = 0.0289).

**Conclusion:** The present study characterized cycles with embryos diagnosed as mosaic and demonstrated that except for aneuploidy type, no differences were observed between LLM and HLM groups. More studies with larger sample size are necessary to confirm these findings and to understand the relation between HLM and monosomy.

**P-102. Aneuploidy for chromosomes 13,18,21, X and Y in blastocysts from ICSI/PGT-A cycles**

**Daniele Pinheiro Freitas^1^, Atila Sena Almeida^1^, Valmira Bispo Oliveira^1^, João Pedro A Freitas^1^, Fernanda Cristina S Batista^1^, Erica S S Leal^1^, Rafaela G Diniz Cunha^1^, Genevieve Marina Coelho^1^**

*^1^ IVI- Instituto Valenciano de Infertilidade - Salvador - BA - Brasil.*

**Objective:** The aim of this study was to analyze the aneuploidy for chromosomes 13, 18, 21, X and Y in embryos from couples undergoing ART (Assisted reproduction techniques) with ICSI (Intracytoplasmic Sperm Injection) and preimplantation genetic test (PGT-A)

**Methods:** This observational retrospective cohort study included 206 patients that performed IVF cycle in combination with preimplantation genetic test for Aneuploidy (PGT-A) at IVI (Valencian Institute of Infertility) Salvador from January 2022 to December 2022. A total of 461 embryos obtained from this cycles were biopsied and vitrified. Embryo biopsies were performed at blastocyst stage (day 5/6). All chromosomes of the embryos were analyzed with next generation sequencing technology (NGS).

**Results:** The mean age of the patients was 38.7 years and the average number of embryos analyzed per cycle was 2.23. A total of 195 (42.3%) blastocysts resulted euploid and 266 (57.7%) were aneuploid, due to monosomy, trisomy, mosaic and complex abnormalities. Of the total number of embryos analyzed, 9 (1.95%) embryos had a result of Trisomy 21, 6 (1.3%) presented Trisomy 18, and 1 (0.21%) were defined as Trisomy 13. Turner (monosomy X) and Klinefelter (disomy X) were not found among the analyzed embryos. Evaluating the chromosomal aneuploidy, maternal age and cause of infertility, we reported that among the embryos presenting Trisomy 21, 5 were from patients aged ≤ 37 years and cause of infertility associated with male factor, and 4 embryos were from women ≥ 37 years and cause of infertility advanced maternal age. The 6 embryos that presented Trisomy 18 were from women aged ≥ 37 years and the cause of infertility associated with maternal age in 5 of them and only 1 was related to both advanced maternal age and male factor. The embryo that presented Trisomy 13 resulted from a 36-year-old patient with male factor infertility.

**Conclusion:** Different factors, such as advanced maternal age, male factor, recurrent miscarriages can affect the success of pregnancy and live births in couples around the world. With PGT-A chromosomal changes may be detected prior to embryo transfer and increase the chances of a successful pregnancy by cycle, transferring an euploidy embryo. With few exceptions, aneuploidies do not appear to be compatible with life. In fact, about 35% of spontaneous abortions are caused by trisomies. Studies show that trisomies can arise from errors of segregation in either parent and any meiotic division. The main numerical chromosomal alterations found in live births are associated with chromosomes 13, 18, 21, X and Y. Of the findings in this study, few aneuploidy embryos had trisomy 13, 18 and 21 and no aneuploidies associated with sex chromosomes were found. Several authors have questioned the practice of PGT-A, however new studies should intensify investigations about genomic medicine associated with assisted human reproduction techniques to expand knowledge in search of better results for couples who undergo IVF treatments.

**P-103. Uterine ischemia following surgical treatment of deep endometriosis as a potential cause of endomyometrial compromise and failure in Assisted Reproduction: a case report**

**Maria Cristina Barcellos Anselmi^1^, João Michelon^2^**

*^1^ Santa Casa de Misericórdia de Porto Alegre-UFCSPA - Porto Alegre - RS - Brasil.*

*^2^ FERTILITAT - Porto Alegre - RS - Brasil.*

**Objective:** To demonstrate that surgical intervention in deep endometriosis can determine structural, nervous, and especially vascular uterine risk, leading to impairment in *in vitro* fertilization outcomes

**Methods:** Case report of a 37-year-old woman who underwent surgery for deep endometriosis with an unfavorable outcome in in vitro fertilization due to probable uterine vascular impairment.

**Results:** A 37-year-old woman with pelvic pain and infertility due to deep endometriosis involving the rectum and bladder sought Assisted Reproduction service and underwent In Vitro Fertilization. She produced 12 good-quality blastocyst embryos and underwent three embryo transfers (ET), a total of 4 embryos transferred, but pregnancy was not achieved. Due to worsening clinical pain, videolaparoscopic surgery was recommended. During surgery, deep implants, the left annex, part of the bladder, and rectum were removed. Hysteroscopy was also performed, which was normal. After the procedure, she experienced amenorrhea with normal FSH levels. She underwent hysteroscopy again, which revealed a small uterine cavity, atrophic endometrium, and adhesions, which were cleared. She remained amenorrheic, and the endometrium did not respond to hormonal therapy and complementary measures. After a few months, she started to experience hypomenorrhea (scanty periods). A repeat hysteroscopy showed the same findings, and another adhesion lysis was performed. After 6 months, she restarted the ET process, and once again, the endometrium was underdeveloped, even with high doses of estrogen. On two occasions, she transferred 2 embryos. In total, she had transferred 8 blastocysts without success. Due to the critical reduction in uterine volume and the formation of adhesions, the hypothesis of uterine ischemia was raised, and an angiore sonance was indicated. The exam showed the absence of the left uterine artery and uncertainty about the origin of the right, as well as a clear reduction in vascular caliber, confirming the hypothesis of ischemia with repercussions on sub-endometrial vessels. Consequently, the transfer of the remaining embryos was suspended, and uterine replacement was recommended

**Conclusion:** The possibility of uterine vascular damage following extensive surgeries for deep endometriosis should be considered in the management of infertile patients.

**P-104. Development and chromosomal constitution of blastocysts in ICSI cycles with sequential culture until the 7^th^ day: A retrospective cohort study**

**Atila Sena Almeida^1^, Daniele Pinheiro Freitas^1^, Valmira Bispo Oliveira^1^, Joao Pedro A Freitas^1^, Fernanda Cristina S Batista^1^, Erica S S Leal^1^, Rafaela G Diniz Cunha^1^, Genevieve Marina Coelho^1^**

*^1^ IVI- Instituto Valenciano de Infertilidade - Salvador - BA - Brasil.*

**Objective:** To investigate the development and chromosomal constitution of blastocysts after sequential culturing until the 7^th^ day of embryonic development.

**Methods:** This retrospective cohort study included patients undergoing intracytoplasmic sperm injection (ICSI) cycles between January 2020 and April 2023 at the Valencian Institute of Infertility in Salvador. A total of 57 patients were included in the study. 487 metaphase II (MII) oocytes were inseminated, and the resulting embryos were subjected to extended culture to assess blastocyst formation on day 7. The following data were collected and analyzed: oocyte age, procedure performed (oocyte donation, ICSI with or without preimplantation genetic diagnosis), as well as the formation and euploidy rate of embryos that reached the blastocyst stage on the 7^th^ day of development.

**Results:** The mean age of the oocytes used in this study was 36.2 ± 2.8 years. Regarding the type of procedure performed, 53% of cycles corresponded to intracytoplasmic sperm injection (ICSI) with preimplantation genetic diagnosis, 31% were ICSI cycles without genetic diagnosis, and 16% were oocyte donation. In total, 487 metaphase II (MII) oocytes were subjected to ICSI, resulting in a fertilization rate of 77.61% with the formation of 187 blastocysts, representing 49.47% of the total number of embryos. 42.78%(80) of blastocysts were formed on the 7th day of embryonic development. For the analysis of chromosomal constitution, 33 embryos were submitted to pre-implantation genetic diagnosis, of which 60.60%(20) were considered euploid, 33.33%(11) presented aneuploidy, and 6.07%(2) still are pending the outcome of the analysis.

**Conclusion:** Based on the analysis of the data collected in this study, it was evidenced that extending the culture of slow developing embryos until the 7th day (D7), and not discarding them on the 6th day (D6), is a viable approach The results revealed a significant number of high-quality blastocysts and a favorable rate of euploidy, providing cruciais insights for selecting embryos with the potential for successful implantation, thereby improving clinical outcomes and the health of live births. Based on the results obtained it is recommended extended blastocyst culture until the 7th day of embryonic development, applying this practice into the clinical and laboratory routine to improve the results of in vitro fertilization cycles. This approach represents a significant advance in the assisted reproduction field and may contribute to the success of fertility procedures in patients undergoing such cycles. However, it is important to carry out more studies to expand understanding and improve clinical practices related to embryonic development.

**P-105. Conservative treatment in a young patient with a rare uterine tumor similar ovarian sex cord tumor diagnosed in IVF/ICSI treatment**

**Maria José Bahia Santos^1^, Maria Carmo Borges Souza^2^, Marcelo Marinho Souza^2^, Roberto Azevedo Antunes^2^**

*^1^ ProFeminina Barra / Fertipraxis - Rio de Janeiro - RJ - Brasil.*

*^2^ Fertipraxis - Rio de Janeiro - RJ - Brasil.*

**Objective:** Uterine tumors similar to ovarian sex cord tumors (UTROSCT) are divided into two groups: endometrial stromal tumors with sexual cord-like elements (Group I), which have a poor prognosis; and UTROSCT itself (group II), with more than 40% of differentiation similar to sexual cords and a smaller endometrial component, biologically less aggressive than tumors of the other group.. We report the case of a woman with UTROSCT treated by minimally invasive hysteroscopic surgery prior to embryo transfer.

**Methods:** A 31-year-old woman with a same-sex relationship undergoing in vitro fertilization (IVF) treatment, asymptomatic from the gynecological point of view, and normal previous transvaginal ultrasound examinations was submitted to outpatient diagnostic video hysteroscopy prior to embryo transfer. The examination identified a small tumor, 0.5 x 0.5 cm, suggestive of hysteroscopic level 1 submucous leiomyoma in the left anterolateral wall of the uterine cavity. Despite the minimum size, resection by surgical videohysteroscopy under sedation was indicated.

**Results:** The histopathological report was compatible with a uterine tumor similar to an ovarian sex cord tumor (UTROSCT). The immunohistochemical panel was positive for AE1/AE3, CK7, WT1, CD10, CD56 and CD 99, with a low cell proliferation index ( ki67). Smooth muscle actin, Inhibin and Calretinin were negative. The morphological and immunohistochemical findings were favorable to the diagnosis of UTROSCT. Two months after surgery, reassessment by hysteroscopy and endometrial biopsy revealed no residual endometrial disease. In three months she had an artificial cycle to prepare the embryo transfer. She received one day 5 blastocyst with no PGT-A previous study, from other 14 cryopreserved embryos ( 8 day-3, 3 other day-5 and 3 day-6 blastocysts). It resulted in a twin pregnancy and a full-term c-section of two healthy boys.

**Conclusion:** Due to the uncertain malignant potential of UTROSCTs and based on the scarce data available in the current literature, fertility-sparing surgery in young patients seems safe. However, a long-term follow-up is necessary and a quick conclusion of a patient's desire to become pregnant was advisable.

**P-106. Pregnancy from in vitro fertilization with heterologous ovum: to tell the child or not?**

**Fernanda Kunrath Robin^1^, Brenda Thamires Comandulli^2^, Milena C Rubenich^2^, Tagma Marina Schneider Donelli^2^, Nilo Frantz^1^**

*^1^ Nilo Frantz Medicina Reprodutiva - Porto Alegre - RS - Brasil.*

*^2^ Universidade do Vale do RIO dos Sinos - São Leopoldo - RS - Brasil.*

**Objective:** Analyse the intention to tell or not to tell the child about the use of a heterologous egg for conception.

**Methods:** This is a qualitative, exploratory and cross-sectional research. One hundred and nine people participated in the study, twenty-one of which were drawn for this research, among which seventeen were women and four were men. As inclusion criteria, individuals who underwent in vitro fertilization with donated eggs were selected, including heterosexual couples and single women. The instrument used was a semi-structured online questionnaire, from which one question was selected for the present study: “Until the present moment, do you think(s) at some point to tell your child about the egg donation treatment?” . The data were verified using the IRAMUTEQ software - which performs a qualitative analysis of the data and the content analysis, from which three categories were later defined: there is intention to tell the child about the use of egg donation; there is no intention to tell the child about the use of egg donation; there is indecision about telling the child about the use of egg donation.

**Results:** The results indicated that six participants intended to tell about the use of egg donation to their child. They considered it important that the child knew his story, about how he was conceived and about his origin, although they felt fearful about the possible reaction of the child to the revelation. Most participants (twelve) had no intention of telling their child. Among the reasons for maintaining secrecy are the consideration that it is irrelevant to tell, the agreements established between the couple to carry out the treatment, and the view that pregnancy is similar to a natural pregnancy, pointing to a denial of egg donation in response to the narcissistic wound that couples suffer in the face of infertility. Of the three individuals who were undecided about revealing the use of a heterologous egg to their child, the idea of not being able to deny the origin of the child is considered the frequent reason for wanting to tell, however the fear of the child's reaction to finding out, leads them to not wish to do so. In these cases, there is also concern about the existence of hereditary diseases that could be transmitted by the donor, which would lead to disclosure.

**Conclusion:** The intention of telling or not telling the child about the use of a heterologous egg is permeated by doubts and depends, largely, on the fears that the parents have about the future and on how they themselves have been elaborating the use of egg donation to conceive the son. It is important to point out that the research was carried out with couples who were pregnant or with very young children, and the theme of the secret in egg donation is more difficult for couples to think about when they still do not consider the moment to tell their child about it. Thus, the tendency is for couples to face more the question of “to tell or not to tell” as their children grow.

**P-107. Experiences of couples about keeping or not the secret about the pregnancy with egg donation**

**Fernanda Kunrath Robin^1^, Brenda Thamires Comandulli^2^, Gabriela Vaz da Rosa Viegas^2^, Tagma Marina Schneider Donelli^2^, Nilo Frantz^1^**

*^1^ Nilo Frantz Medicina Reprodutiva - Porto Alegre - RS - Brasil.*

*^2^ Universidade do Vale do Rio dos Sinos - São Leopoldo - RS - Brasil.*

**Objective:** Investigate the experiences of heterosexual couples about whether or not to keep a secret to the child about the adoption of a heterologous egg in treatment of in vitro fertilization.

**Methods:** This is a qualitative, exploratory research, with multiple case studies. Three heterosexual couples aged between thirty-six and fifty-two years participated, in which the children were conceived by in vitro fertilization with egg donation, using homologous sperm in the treatment. Data collection was carried out based on the indication of contacts and two sociodemographic forms and two semi-structured interviews were used about the experience of maternity and paternity in the context of assisted reproduction with adoption of a heterologous egg. The interviews were conducted online and synchronously, being recorded and transcribed for later analysis. The results were raised from the thematic analysis, being organized from two axes named "maintenance (or not) of secrecy in the social scope" and "desire to reveal (or not) to the children".

**Results:** Two couples had disagreements about telling or not in the social sphere and whether or not they wanted to reveal the egg donation to their child. Couple A kept the egg donation only among the nuclear family, talking only about in vitro fertilization to family members and close friends, fearing the repercussion of their decision and judgment in society. In this case, the woman had no intention of telling her daughter, while the man did, feeling secure in the presence of his genetics. Couple B revealed about the egg donation to people close to them, but they did not reach an agreement to reveal to their children about how they were conceived, having kept documents that prove the pregnancy as a way of ensuring parenthood by reaffirming it biologically. In this case, the woman questioned herself about what would be the right time to tell, while the husband explained that the revelation was not important to be carried out, since she had carried the twin children. Only couple C agreed to tell and they had already told about the egg donation, both socially and for the child, explaining that family secrets could harm the relationship with the child. In this case, both worked in the area of assisted reproduction and gave lectures on the subject, understanding that the child already knew how it had been conceived.

**Conclusion:** When experiencing infertility, a narcissistic wound opens up, weakening the trying couple. When faced with the possibility of egg donation, they embark on a new journey, filled with doubts. The question of whether or not to tell the child about the egg donation seemed controversial for some couples in this study, requiring more time and dialogue between the spouses. The way in which each couple faces the issue of disclosure is marked by the subjective experiences of each spouse along the trajectory in search of the much-desired pregnancy, making it important to listen to each case in its uniqueness.

**P-108. The impacts of human papillomavirus on sperm quality and embrionary implantation rates: a narrative review**

**Maria Eduarda Leite Simoes^1^, Fernanda Perini Concatto^1^, Emilly Brandao Schuck^1^, Caroline Wilhelmsen Martins^1^, Bruna Silva Janke^1^**

*^1^ Liga de Reprodução Humana - LIGAR - Porto Alegre - RS - Brasil.*

**Objective:** This review aims to study the influence of human papillomavirus (HPV) in human fertility, especially on sperm quality and the possible risks it can cause to the embryo implantation.

**Methods:** The present study is a narrative review of the literature searching the ScienceDirect and PubMed databases for the descriptors MeSH: "human papillomavirus", "infertile men", "assisted reproductive technologies" and "sperm motility".

**Results:** A total of 101 articles were found, removing duplicates and unavailable articles, by the eligibility criteria 9 studies remained for analyses. According to the findings for this study, human papillomavirus (HPV) is one of the most common sexually transmitted infections (STIs) in the world, affecting a large portion of sexually active people at some point of their lives, especially in their young adulthood. HPV is a double DNA strand virus, responsible for causing lesions on the oral and genital mucosa and on the skin. This sexually transmitted disease is also associated to both cancer and male and female infertility. In vitro studies show that sperm quality damage and higher spontaneous abortion rates are related to human papillomavirus infection. In situ hybridization (ISH) studies revealed that the virus DNA is allocated in the head and in the intermediate piece of the spermatozoids, that conducts the virus to the oocyte during fertilization, leading to a possible expression in the developing blastocyst. The presence of the virus was shown to increase apoptosis of the trophoblast cells and therefore reduce the embryo implantation rates, raising the risks of spontaneous abortions. Besides that, there has been a correlation between the HPV infection and the decrease of sperm quality, once the HPV-positive samples presented motility and spermatic concentration significantly reduced when compared to HPV-negative samples. The levels of superoxide dismutase (SOD), an enzyme related to the elimination of reactive oxygen species (ROS) in the seminal plasma were also analyzed and shown to be higher in HPV-positive patients.

**Conclusion:** The analyzed articles, therefore, provide evidence that the human papillomavirus is related to reduced natural and assisted pregnancy rates and increased miscarriage rates, especially when the virus is found in the male sperm cells. In light of this insight, it is important to perform HPV testing in every couple undergoing infertility treatments, once the possibility of delaying IVF procedures until the viral infection is no longer detected could be taken into consideration in order to increase the pregnancy odds.

**P-109. Full-term pregnancy in a patient with panhypopituitarism using in vitro fertilization techniques: a case report**

**Maria Eduarda Leite Simoes^1^, Leticia Quandt^1^, Laura Gazal Passos^1^, Markus Berger Oliveira^1^, Ivan Sereno Montenegro^1^, Eduardo Pandolfi Passos^1^, Paula Barros Terraciano^1^**

*^1^ Grupo de Reprodução e Farmacologia celular (REPROFARM), Centro de Pesquisa Experimental, Hospital DE Clinicas de Porto Alegre - Porto Alegre - RS - Brasil.*

**Objective:** Description of a case report about a 24-year-old female previously diagnosed with panhypopituitarism (PHP), who was referred to the Gynecology and Obstetrics department at the General Clinical Hospital in Porto Alegre - Rio Grande do Sul, Brazil, for treatment of secondary infertility. She expressed her desire to become pregnant and was included in our Public Health Program in Assisted Reproduction. The patient had already been under periodic endocrinological follow-up since childhood at the same hospital to treat the PHP condition, which was a consequence of pituitary stalk agenesis.

**Methods:** When started in the Assisted Reproduction Program, the patient was receiving continuous treatment with levothyroxine (Euthyrox^®^ - 125 mcg/day), estradiol (System^®^ 25 - 1 patch every 2 days), medroxyprogesterone (Provera^®^ 2.5 mg - (from day 13-23 of menstrual cycle), and prednisone 2.5 mg/day. Her endocrinologist recommended increasing the Provera^®^ dosage to 5 mg (from day 13-23). However, she had stopped taking GH replacement four months before starting the procedure, on her own. The patient's spouse provided a semen sample for a spermogram, and all parameters were within normal limits. Despite this, the first ovulation induction cycle for fertilization in the laboratory was unsuccessful. A year later the patient attempted another ovulation induction. This time, human menopausal gonadotropin (HMG) was used at a dose of 150 IU/day, starting two days after the initial menstrual bleeding. HMG treatment continued for six days. The patient's Somatomedin C (IGF-1) levels were measured at 155 ng/mL, falling outside the recommended reference levels for her age. As a result, the patient was advised to discontinue GH replacement. Moreover, the ultrasound revealed another poor response to ovulation induction, leading to the termination of the induction cycle. Three months later, the patient made a third attempt at ovulation induction. This time, the HMG dose was increased to 225 IU/day, starting three days after the onset of menstrual bleeding. The HMG treatment continued for six days, and ovulation control ultrasound was performed on the sixth day. Additionally, IGF-1 levels were determined again and resulted in 205 ng/mL, within the reference value. Ultrasounds were performed every two days from the ninth day of the cycle to monitor follicular growth until the seventeenth day, with the same medication dose.

**Results:** The follicles were punctured on the twentieth day of the cycle, resulting in 15 collected oocytes. Among them, 13 were mature, and 2 degenerated. Considering that the seminal parameters of the spouse were within normal limits, with a total motility of 90% and a concentration of 150x106 spermatozoa, classic in vitro fertilization (IVF) was used for oocyte fertilization. From the 13 inseminated oocytes, 11 embryos were obtained on D3, with four having embryonic classification B and seven having embryonic classification C. Two healthy embryos (classification B) were freshly transferred to the patient, and the remaining nine embryos were cryopreserved. After 15 days, Beta HCG levels were determined, confirming a single embryo pregnancy. Given the high-risk pathology, the patient was referred to a specialized obstetric center for careful follow-up throughout the entire gestational period. The birth occurred at 38 weeks' gestational age, through cesarean delivery.

**Conclusion:** In women with PHP, spontaneous pregnancy, even if rare or exceptional, is associated with a high risk of miscarriages or fetal and maternal mortality. Thus, IVF techniques may be the only option for PHP patients to have a safe full-term pregnancy.

**P-110. Beyond Technique: Welcoming in the Patient Treatment Process in Assisted Human Reproduction**

**Queila Silva Raimundo^1^**

*^1^ Instituto Ideia Fértil de Saúde Reprodutiva - Santo Andre - SP - Brasil.*

**Objective:** This article aims to delineate the importance of patient welcoming in the context of assisted human reproduction treatment. It examines professionals who adopt a personalized approach, taking into consideration each patient's individual circumstances, and offer more effective and tailored support as required during the treatment process. Empathetic communication, teamwork, clear information provision, continuous emotional support, and personalized care are factors contributing to a more humane, understanding, and successful treatment.

**Methods:** This study is based on a literature review of fourteen scientific articles and literary works. The specific theme of the article is "The importance of patient welcoming in the context of assisted human reproduction treatment." A search was conducted in reliable databases such as PubMed and Google Scholar using relevant keywords. Scientific articles, reviews, and studies related to the topic were sought. The articles relevant to the theme were selected. The objectives of the studies, methodologies employed, results, and conclusions were analyzed. A critical analysis was performed to assess the study's quality, the validity of the information presented, and the relevance of patient welcoming in assisted human reproduction treatment, as it can lead to significant changes in patients' lives.

**Results:** Among the fourteen articles read, eleven agreed that patient welcoming in human reproduction can lead to various positive outcomes. Three articles pointed out that welcoming is practiced in conjunction with other aspects, making the results less explicit. There is still much to be studied, but the results are promising. Patients who receive a welcoming approach tend to have a more positive treatment experience, leading to increased adherence to the procedures. Additionally, the emotional support provided by professionals reduces stress and anxiety, thus improving patients' mental well-being. The professional-patient relationship is strengthened, resulting in higher patient satisfaction with the treatment. Emotional support is also crucial in cases of treatment failure, assisting patients in coping with grief and in pursuing new attempts and alternatives. As a result, patients tend to approach the treatment with fewer expectations and greater resilience and emotional balance, contributing to a better quality of life throughout the assisted human reproduction process.

**Conclusion:** Welcoming patient treatment in assisted human reproduction plays an essential role in providing emotional support, relevant information, and overall well-being to patients. Collaborative efforts among doctors, social workers, psychologists, nurses, and other professionals are crucial in helping patients face infertility with greater resilience, thereby enhancing their chances of treatment success and overall quality of life. Empathetic and human welcoming is a powerful tool that can make the treatment process less burdensome and more hopeful for those pursuing the dream of having a child.

**P-111. Elevated progesterone on trigger day has no impact on blastulation rate**

**Luiza da Silva Rodrigues^1^, Lisiane Knob de Souza^1^, Bruna Campos Galgaro^1^, João Sabino Lahorgue Cunha Filho^1^**

*^1^ Insemine - Porto Alegre - RS - Brasil.*

**Objective:** To evaluate the effect of serum progesterone level on the hCG trigger day on blastulation rate.

**Methods:** This retrospective analysis included IVF/ICSI cycles performed from January 2022 to June 2023 that underwent extended culture to the blastocyst stage. Patients were divided into two groups according to the serum progesterone level on the hCG trigger day: control (<1,5 ng/mL) and elevated progesterone (study group, ≥1.5 ng/mL). The primary outcome of this study was blastulation rate. Antral follicle count, AMH, number of oocytes retrieved and fertilization rate were also compared.

**Results:** A total of 81 IVF/ICSI cycles were included in this study. Mean serum progesterone level in the control group (n=58) was 0.86 ng/mL and 2.178 ng/mL in the study group (n=23). As expected, since AMH and progesterone are both products of the ovarian granulosa cells, AMH was higher in the elevated progesterone group [4.20 ng/mL ± 3.17 vs 2.45 ng/mL ± 1.89 (*p*=0.003)], as well as the antral follicle count [14.3 ± 5.0 vs 11.8 ± 6.1 (*p*=0.052)] and the number of oocytes retrieved [16.0 ± 8.5 vs 10.4 ± 6.5 (*p*=0.001)]. There was no statistical difference in the fertilization rate [88.5% ± 0.16 vs 88.5% ± 0.15 (*p*=0.715)], nor in the blastulation rate [47.8% ± 0.35 vs 41% ± 0.28 (*p* = 0.498)] between control and study groups, respectively. We also performed a multivariable analysis, which confirmed our findings that blastulation and increased progesterone on the hCG day are not associated.

**Conclusion:** Our results indicated that serum progesterone above 1,5 ng/mL on hCG trigger day has no association with blastulation rate.

**P-112. Levels of estradiol and progesterone in the middle luteal phase (Day 6 after transfer) and correlation with ongoing and live birth rates with transfer of thawed blastocysts in artificial cycles**

**Rodopiano Souza Florêncio^1^**

*^1^ Humana Medicina Reprodutiva - Goiânia - GO - Brasil.*

**Objective:** In recent years, there has been a growing interest in progesterone (P4) levels measured on the day of embryo transfer or 1 day before in artificial cycles, as there could be a cutoff point in P4 levels above which the chances of pregnancy increase. Few studies have measured P4 levels early, specifically on Day 6 after blastocyst transfer (DT6) and correlation with ongoing or live birth rates (ONG/LBR). In this study, we evaluated pregnancy chances in relation to estradiol (E2) and P4 levels measured on DT6 of viable blastocysts in cycles without the presence of corpora lutea (artificial cycles).

**Methods:** Patients from a private clinic who underwent frozen embryo transfer (FET) between January 2017 and December 2021, using artificial cycles (6mg/day E2 valerate and occasionally adding transdermal gel in the endometrial proliferative phase, and 600mg/day vaginal or 50mg/day intramuscular micronized P4), that were followed by the author. E2, P4, and beta-hCG levels were measured on DT6 (transfer day, Day 0). In this period 227 transfers were performed by the author. Of these,73 cycles were excluded for different reasons, remaining 154, which met all the criteria. We evaluated the cutoff point on DT6 for these two hormones using the ROC curve and area under the curve (AUC) to determine a threshold for assessing ONG/LBR above and below the cutoff point, determined by sensitivity and specificity. We also assessed ONG/LBR by range of progesterone levels. Furthermore, we compared the mean P4 levels between the vaginal and intramuscular groups and their ONG/LBR.

**Results:** The ROC curve for E2 showed the highest sensitivity/specificity for ONG/LBR with a dosage of 158.5 pg/ml and a range of 49.8 to 1149 pg/ml and odds bellow and above 158.5 pg/ml were 21,43% and 36.51% respectively, and *p*=0.1843. Then, we had a curve flattened with an AUC of 0.5169, *p*=0.7334. The ROC curve for P4 showed the highest sensitivity/specificity with 9.6 ng/ml and a range of 3.9 to 47 ng/ml, with an AUC of 0.5901, *p*=0.0678. The pregnancy rates for ONG/LBR below and above the 9.6 cutoff point were 50% and 25.9% and OR 0.3506 (0.1771 - 0.7132), *p*= 0,0038. The odds for ONG/LBR by P4 bands were A - 3.9 to 7 ng/ml (41,1%), B - 7.1 to 10 ng/ml (50%), C - 10.1 to 20 ng/ml (25%) and D - > 20 ng/ml (29,7%). The p values were p.a/b= 0.5742, p.b/c= 0.0170 and p.c/d= 0.6452 (table 1). The mean P4 dosage was 12.7 ng/ml for vaginal and 22.2 ng/ml for intramuscular use, with pregnancy chances for ONG/LBR at 37,1% in the vaginal group and 26.5% in the intramuscular group, OR 0.6111 (0.2967 - 1.291), *p*=0.2070.

**Conclusion:** Our data show no predictive value for pregnancy ONG/LBR with E2 dosage and low prognostic capacity for pregnancy ONG/LBR with P4 measurements. Determining a cutoff showed no utility for E2, and the P4 showed higher chances of ONG/LBR below 9.6 ng/ml. The mean P4 dosages with vaginal or intramuscular use were different as expected, but ONG/LBR were similar.

**Table 1:** Progesterone ranges on days 6-7 and pregnancy ongoing/live birth rates.

Range PRG (ng/ml)	ONG (positive)	ONG (negative)	% ONG	p
A - 3.2 - 7	07	10	41,1	
B - 7.1 - 10	19	19	50	a/b=0.5742
C - 10.1 - 20	15	47	25	b/c=0.0170
D - > 20	11	26	29.7	c/d=0.6452

**P-113. Severe ovarian hyperstimulation syndrome associated with long-acting GnRH agonist after oocyte retrieval in oncofertility patient: a case report**

**Yahn Rezende Abreu^1^, Ana Luiza Pereira Saramago1, Camila Toffoli-Ribeiro^1^**

*^1^ Universidade Federal de Uberlandia - Uberlândia - MG - Brasil.*

**Objective:** To report a case of severe ovarian hyperstimulation syndrome (OHSS) in oncofertility patient receiving chemoprotective long-acting GnRH agonist (GnRHa_dep) after controlled ovarian hyperstimulation (COH).

Methods: Medical record and literature review about OHSS in oncofertility patients.

**Results:** A 28 year-old woman with luminal breast cancer presents to medical consult for fertility preservation. Random start COH for egg retrieval was indicated. Basal antral follicle count was 32 and there was a 15mm follicle at the time COH was started (D1). The adopted protocol was follitropin delta (72mg), combined to urofollitropin (450UI). Clomifene citrate and letrozole were associated for ovulation inhibition and to minimize impact on cancer cells. Ovulation was detected on D6. In D9 double triggering was performed with 2500 UI human gonadotrofin (hCG) and 0,1mg triptorrelin subcutaneously. Thirty-three oocytes were aspirated, and 24 were frozen in M2 stage. The patient presented abdominal distension, light pain, reduced appetite, nausea and constipation from the day after the procedure. Support measures and telemonitoring were thoroughly adopted. Five days after follicle aspiration there was worsening of the symptoms. From sixth day on there was a slow but steady improvement, as menstrual flow began. On the eigth day post oocyte retrieval (OR) she was feeling significantly better, when chemotherapy was iniciated. GnRHa_dep (10,8mg goserelin) was prescribed by the oncologist for fertility preservation despite recent OR. Forty-eight hours after GnRHa_dep there was major worsening of OHSS, with detection of dyspnea, resurgence of ascites, tachycardia (124 bpm), vomiting and dehydration. Pacient was hospitalized for intensification of hydration, analgesia, profhylatic heparinization. Significant ascites and small pleural effusion were observed and 3 liters of serosanguineous peritoneal fluid were aspirated by ultrasound guided paracentesis. Total lenght of hospitalization was 10 days. OHSS is a major complication associated with assisted reproductive technology (ART) and occurs in approximately 1-5% of treatment cycles. It is characterized by ovarian enlargement, ascites, hemoconcentration, hypercoagulability, and electrolyte imbalances. The cause of OHSS is believed to be massive luteinization of granulosa cells induced by hCG with production of vascular endotelial growth factor (VEGF) which increases vascular permeability. The gold standard strategy for fertility preservation in female cancer patients is oocytes or embryo cryopreservation. GnRHa_dep is also supposed to exert a chemoprotective effect on the ovaries and long acting formulations have come into common usage as co-treatment during chemotherapy. Short after GnRHa_dep is given, there is a gonadotropin “flare” phase resulting in a rise in FSH, LH, and estradiol, which usually lasts for 7-14 days. It is believied that sustained gonadotropin elevation during the flare phase of GnRHa_dep may mimic the effect of hCG and contribute to severe OHSS cases as the one reported here. In a previous study, the authors demonstrated that the function of human corpus luteum can be rescued and function normally after seven days of deprivation from gonadotrophin stimulation in patients with hypogonadotropic hypogonadism. Therefore, we may assume that the risk of rescuing corpus luteum (and potentially causing OHSS) with GnRHa_dep injection remains at least until the seventh-eighth day after OR. Candidates to hormonal chemoprevention might not receive GnRHa injection for this period (or even more) to avoid corpus luteum rescuing. Severe OHSS may be especially catastrophic for patients undergoing chemotherapy as this can result in delays in initiating cancer treatment as well as place the patient at risk for significant complications.

**Conclusion:** Risk of severe OHSS may be increased when a GnRHa_dep is used for chemoprotection following oocyte retrieval. The timing for GnRHa_dep administration should be established between oncologist and gynecologist in order to avoid risks.

**P-114. Seminal capacitation techniques for intracytoplasmic sperm injection based on BHCG results in the year 2021: A retrospective cohort study**

**Rafaela G Diniz Cunha^1^, Valmira Bispo Oliveira^1^, Atila Sena Almeida^1^, Fernanda Cristina S Batista^1^, Daniele Pinheiro Freitas^1^, Joao Pedro A Freitas^1^, Erica S S Leal^1^, Genevieve Marina Coelho^1^**

*^1^ IVI- Instituto Valenciano de Infertilidade - Salvador - BA - Brasil.*

**Objective:** Analyze the seminal capacitation techniques used for intracytoplasmic sperm injection in 2021 and compare them with the BHCG results.

**Methods:** Retrospective cohort study in which 503 BHCG results of patients who underwent Intracytoplasmic Sperm Injection (ICSI) at the IVI Salvador clinic during the period from January to December 2021 were analyzed, relating them to their respective seminal capacitation techniques. The patients were separated according to the chosen sperm selection technique and the results were divided into positive and negative. The methods considered were Discontinuous Density Gradient, Swim up and Simple Wash.

**Results:** In the 92 patients in whom the seminal capacitation technique was the Density Discontinuous Gradient, 54.35% (50) of the results were positive, and 45.65% (42) were negative. In 298 patients whose chosen technique was Swim up, 59.73% (178) of the cases developed a positive result, while 40.27% (120) had a negative result. When we talk about the Simple Wash technique, performed on 113 patients, 57.85% (63) of the patients had a positive result, while 42.14% (50) had a negative result.

**Conclusion:** Based on the study's results, it is possible to conclude that, in the year 2021, the sperm capacitation techniques selected for intracytoplasmic sperm injection (ICSI) performed at the IVI Salvador clinic obtained positive results in different proportions. Therefore, no significant difference demonstrates the influence of seminal capacitation techniques on BHCG results. However, we emphasize that the analysis was performed on a heterogeneous population sample, where the results cannot be applied to the general population. It is also important to highlight that the choice of sperm selection technique must be personalized, taking into account the specific characteristics of the couple, thus making individual cases to obtain better results.

**P-115. Successful Pregnancy Achievement after Transfer of Euploid Embryo Originating from Oocyte Fertilized with 3 Pronuclei (3PN)**

**Rafaela Aguiar^1^, Diana Caroline da Silva Bastos^1^, Julia Gonçalves do Carmo^1^, Edson Guimaraes Lo Turco^2^, Fernando Prado Ferreira^3^, Raquel Mitie Koike^1^, Alexandre Akio Takara Gusukuma^1^, Eliana Junko Morita^1^**

*^1^ CITI Hinode - SP - SP - Brasil.*

*^2^ Departamento de Ginecologia, UNIFESP - SP - SP - Brasil.*

*^3^ Neo Vita - SP - SP - Brasil.*

**Objective:** In this case report, the successful pregnancy was accomplished following the transfer of a chromosomally normal embryo resulting from fertilization of an oocyte with three pronuclei (3PN), which underwent comprehensive ploidy analysis prior to implantation.

**Methods:** In this study, a 40-year-old patient underwent in vitro fertilization (IVF) using donated oocytes and donor sperm, and 12 mature oocytes were allocated for the treatment. Fertilization assessment occurred 19 hours after intracytoplasmic sperm injection (ICSI), and subsequent embryo development was individually monitored in continuous culture medium. Assessments were conducted on day 3, with assisted hatching, and on days 5, 6, and 7 at the blastocyst stage. Blastocysts that reached the expanded blastocyst stage underwent biopsy followed by freezing, and the biopsied cells were sent to the genetics laboratory for pre-implantation genetic testing for aneuploidies (PGT-A) and ploidy analysis for the potential transfer, thereby augmenting the likelihood of a successful pregnancy outcome.

**Results:** Among the 12 injected oocytes, all showed signs of fertilization, with 8 of them having 2 pronuclei, 1 with 1 pronucleus, 1 with 3 pronuclei, and 2 with 2 polar bodies. On the third development day, the 3PN-derived embryo displayed 14 cells with <10% fragmentation. On the fifth day of development, four embryos reached blastocyst level for biopsy, including one originating from a 3PN fertilized oocyte. This embryo received a 5AB grading according to Gardner's criteria, indicating the best morphological classification compared to other blastocysts. Genetic analysis was performed on samples from two blastocysts, confirming their euploid status. Notably, one of the euploid embryos was derived from a 3PN oocyte, and genetic analysis revealed it to be a diploid embryo with a mitoscore of 19.01 The decision to transfer this specific euploid embryo was based on its superior quality compared to the others. Subsequently, the 3PN-derived euploid embryo underwent freezing and was transferred to a surrogate uterus in the following month. The following month, the embryo was thawed and transferred to a surrogate uterus, given the patient's history of pregnancies with early growth restriction, leading to fetal demise due to uterine conditions. Two hours before the transfer, the embryo was thawed and was fully expanded at the time of transfer. Monitoring the patient's hormone levels through beta-HCG measurements revealed a consistent increase, with 349.65 mIU/mL at nine days post-transfer, 916.70 mIU/mL on day 11, and 2,237.06 mIU/mL on day 14. Ultrasound examinations confirmed the pregnancy at 6 weeks and 12 weeks, showing a healthy, viable fetus with normal development. At 12 weeks, the patient underwent a morphological ultrasound, revealing a single, viable fetus with a crown-rump length of 5.4 cm, a nuchal translucency of 1mm, and a visible nasal bone. Currently, at 15 weeks of gestation, the patient is undergoing uncomplicated prenatal follow-up.

**Conclusion:** Further research and larger studies are warranted to validate the findings of this case report and to elucidate the potential implications of 3PN-derived euploid embryos on overall IVF success rates and reproductive outcomes.

**P-116. Impact of Paternal Age on Embryo Development: A Case Report of Thawed Oocytes Inseminated with PESA-Derived Sperm in a 73-Year-Old Male**

**Rafaela Aguiar^1^, Diana Caroline da Silva Bastos^1^, Julia Gonçalves do Carmo^1^, Edson Guimaraes Lo Turco^2^, Fernando Prado Ferreira^3^, Raquel Mitie Koike^1^, Alexandre Akio Takara Gusukuma^1^, Eliana Junko Morita^1^**

*^1^ CITI Hinode - SP - SP - Brasil.*

*^2^ Departamento de Ginecologia, UNIFESP - SP - SP - Brasil.*

*^3^ Neo Vita - SP - SP - Brasil.*

**Objective:** This case report describes a fertility intervention utilizing percutaneous epididymal sperm aspiration (PESA) in a male patient aged 73 years, aiming to present the treatment cycle and its outcomes

**Methods:** In this study, a 73-year-old male patient with a history of vasectomy 26 years ago underwent a procedure known as Percutaneous Epididymal Sperm Aspiration (PESA). PESA involved making two punctures on the right epididymis to retrieve motile sperm for use in Intracytoplasmic Sperm Injection (ICSI). Thirteen donated oocytes (egg cells) were thawed for this particular case, and all of them survived the thawing process. Out of these oocytes, seven were subjected to ICSI using sperm obtained through PESA, while the remaining six oocytes were injected with sperm from a previously frozen semen sample acquired from a sperm bank. Fertilization assessment was conducted 19 hours after the ICSI procedure to determine the success of sperm-egg fertilization. On the fifth and sixth days of embryonic development, Preimplantation Genetic Testing for Aneuploidy (PGT-A) was performed on the resulting embryos.

**Results:** All injected oocytes exhibited fertilization, with six fertilized by the semen bank sample displaying two pronuclei (2PN). Among the seven oocytes fertilized with PESA sperm, four showed 2PN, one displayed 1PN, and two exhibited 2 polar bodies. During embryo development, ten blastocysts were biopsied and cryopreserved-five from oocytes fertilized with the semen bank sample and five from PESA-fertilized oocytes. The PESA group achieved a 71% blastocyst formation rate relative to injected oocytes, while the semen bank group achieved 83% blastocyst formation. On the fifth day of embryo development from PESA-originated embryos, we observed the classifications 5AB, 5BB, and 3BB, based on Gardner's grading system. On the sixth day, two blastocysts were classified as 5BB. Genetic analysis was only performed on PESA-derived embryos, revealing two euploid blastocysts, 5AB and 3BB on D5, and three aneuploid blastocysts. The 5AB PESA blastocyst was thawed and transferred. Nine days after the transfer, the beta-HCG level was measured at 373.7. The beta-HCG values demonstrated expected growth, doubling every two days during monitoring. The patient's pregnancy is progressing without complications.

**Conclusion:** In conclusion, this case report underscores the feasibility of attaining optimal-quality embryos with euploid genetics using PESA-derived sperm from patients aged above 70 years.

**P-117. Do time-lapse incubator systems improve results in embryo development?**

**Priscila Carvalho^1^, Ivan Henrique Yoshida^1^, Rodrigo Angelo Souza^1^, Caroline Zulim Berton^1^, Emerson Barchi Cordts^1^, Caio Parente Barbosa^1^**

*^1^ Instituto Ideia Fértil de Saúde Reprodutiva - Santo André - SP - Brasil.*

**Objective:** Evaluate the fertilization, embryo quality, blastulation, and ploidy rates of embryos cultivated in k-systems benchtop incubators (KS) versus embryoscope (ES) time-lapse systems.

**Methods:** A retrospective study was conducted, including 152 cycles of ICSI + PGTa from January to May 2023. Among them, 116 patients underwent embryo culture in k-systems incubators, and 36 patients were cultivated in embryoscope incubators. A total of 996 zygotes were evaluated, with patients distributed into three groups based on age: Group 1 - patients up to 35 years old, with 23 patients in KS and 9 in ES. Group 2 - patients aged 36 to 39 years, with 46 patients in KS and 10 patients in ES. Group 3 - patients aged 40 or older, with 47 patients in KS and 17 patients in ES. The same protocol and unique culture medium were used for both incubators. The following variables were assessed: total fertilization rate; 2PN fertilization; top-quality embryos on days 3, 5, and 6; blastulation; and results of embryo genetic analysis (PGT-a). All cycles were subjected to extended culture, and embryo biopsy was performed on the 5th or 6th day of development. Preimplantation genetic testing (PGT-a) was performed using next-generation sequencing (NGS), and embryos were categorized as euploid or aneuploid. Statistical analysis was performed using Pearson's chi-square test or Fisher's exact test, considering a p-value <0.05.

**Results:** Out of 996 zygotes, 705 were cultured in KS incubators and 487 evolved to blastocyst stage (69.0%); in ES incubators, 291 zygotes were cultured, with 202 reaching blastocyst stage (69.0%). When splited by age, the results were as follows: Group 1: In KS, 177 zygotes and 132 blastocysts were formed (75%); in ES, 84 zygotes and 63 blastocysts (75%). Group 2: In KS, 308 zygotes and 229 blastocysts (74%); in ES, 64 zygotes and 54 blastocysts (84%). Group 3: In KS, 220 zygotes and 126 blastocysts (57%); in ES, 143 zygotes and 79 blastocysts (55%). Regarding the rates of total fertilization and top-quality embryos (D3/D5/D6), there was no statistical difference between the groups. However, in the evaluation of the 2PN fertilization rate, there was a statistical difference (p = 0.024) in Group 3, with ES showing a higher rate compared to KS (78% and 69%, respectively). Also, in the blastulation analysis, there was a statistical difference in Group 2 (p = 0.040), with ES outperforming KS (79% and 67%, respectively). In the comparison between groups about genetic analysis, the evaluated parameters did not show significant statistical differences.

Conclusion: The results obtained from this study support the understanding that embryo culture in embryoscope time-lapse systems is equivalent and, in some aspects, even superior to those obtained in benchtop incubators.

**Conclusion:** Our results indicated that serum progesterone above 1,5 ng/mL on hCG trigger day has no association with blastulation rate.

**P-118. Maternal and neonatal outcomes after frozen embryo transfer with and without PGT**

**Mariangela Badalotti^1^, Vanessa Trindade^1^, Marta Ribeiro Hentschke^1^, Isadora Badalotti-Teloken^2^, Ricardo Azambuja^1^, Fabiana Mariani Wingert^1^, Natália Fontoura Vasconcelos^2^, Victoria Campos Dornelles^2^, Alvaro Petracco^1^**

*^1^ Fertilitat Centro de Medicina Reprodutiva - Porto Alegre - RS - Brasil.*

*^2^ Pontifícia Universidade Católica - PUCRS - Porto Alegre - RS - Brasil.*

**Objective:** It is known that children born after frozen embryo transfer (FET) and in vitro fertilization have some differences in neonatal outcomes. However, there is no sufficient data in the literature comparing these parameters in pregnancies and births after FET with or without preimplantation genetic test (PGT) biopsy. Thus, the objective of this study was to analyze if there is any difference regarding obstetric and perinatal outcomes after FET with/without PGT.

**Methods:** Retrospective, observational, cross-sectional study. A total of 897 pregnancies were analyzed between 2016 and 2021. The sample was divided into two groups: G1 - did not perform PGT (n=629 pregnancies, 546 singletons, and 83 twins), and G2 - performed PGT (n=268 pregnancies, 247 singletons, and 21 twins). All embryos were submitted to hatching after thawing; therefore, the embryos submitted to PGT received two hatchings, according to the clinical protocol. The variables regarding maternal and neonatal outcomes were analyzed separately for single or twin pregnancies. Statistical analysis: Student's t-test, chi square, and Fisher's exact test, considering *p*<0.05.

**Results:** Data were compared between G1 vs. G2, respectively: twins rate after one embryo transfer (1.6% vs. 3.8%, *p*=0.064); and after two embryo transfers (25.4% vs. 35.3% *p*=0.157). Single pregnancy results (G1 vs. G2): gestational age (GA), wks (38.2 vs. 38.1, p=0.382); prematurity, %: (12.0 vs. 14.6, *p*=0.315); birthweight, g (3259.8±561.0 vs. 3209.0±587.9, *p*=0.245); small for GA,% (4.2 vs. 3.6, *p*=0.705); low birthweight, % (4.4 vs. 3.2, *p*=0.447); Apgar 5' >7,% (96.7 vs. 97.2, p=0.743); neonatal UCI admission,% (4.9 vs. 6.9, *p*=0.187); maternal hypertension, % (5.6 vs. 6.1, *p*=0.629); gestational diabetes, % (4.0 vs. 3.2, *p*=0.692). Twin results (G1 vs. G2): number of fetuses (166 vs. 42), GA, wks (35.1 vs. 36.1, *p*=0.101); prematurity, % (71.8% vs. 66.7%, *p*=0.647); weight, g (2321.8±554.4 vs. 2463.7±346.5, *p*=0.139); Apgar 5th >7, % (92.8 vs. 95.2, *p*=0.741); there were no differences in maternal and fetal complications or chromosomal anomalies.

**Conclusion:** There were no statistically significant differences considering maternal and perinatal outcomes regardless if the embryo was biopsied in frozen embryo transfers. The biopsy seems safe for the newborn and does not increase maternal complications. There is a tendency for a higher twinning rate when one biopsied embryo is transferred, and the twin pregnancy rate is extremely high when two embryos are transferred in both groups.

**P-119. Next-generation of Oocyte In Vitro Maturation: Potential 3D Matrix using Bovine Model**

**Sarah Gomes Nunes^1^, Raquel Zaneti Puelker^2^, Bruno Carrino Suave^3^, Alan Brunholi Giroto^3^, Thaisy Tino Dellaqua^1^, Vitor Andrade Ferreira^3^, Janice Koepp^4^, Guilherme Colla^4^, Anthony Cesar de Sousa Castilho^4^**

*^1^ São Paulo State University (UNESP) - Botucatu - SP - Brasil.*

*^2^ Blastocell Biotechnologies - Botucatu - SP - Brasil.*

*^3^ University of Western Sao Paulo States (UNOESTE) - Presidente Prudente - SP - Brasil.*

*^4^ Biocelltis Biotecnologia - Florianópolis - SC - Brasil.*

**Objective:** Despite advances in assisted reproductive technologies (ART), current conditions in human medicine and animal production desired standards. Abnormalities in oocytes matured under in vitro maturation (IVM) conditions can negatively affect embryonic development. In comparison, oocytes matured in vivo produce more and better embryos with differences in morphology, metabolism, and gene expression than in vitro embryos. The culture environment has a significant impact on bovine blastocyst development. Traditionally, IVM has relied on two-dimensional (2D) culture systems that cannot fully reflect the complexity of the in vivo environment. Consequently, oocytes matured in 2D systems may be of lower quality, resulting in a lower rate of successful embryo development. In contrast, three-dimensional (3D) culture systems provide a more biomimetic environment by using matrices or scaffolds that closely resemble the extracellular matrix in vivo. These matrices support interactions between oocyte and cumulus cells, promote a more natural cell-cell communication, and provide a microenvironment that better resembles the ovarian follicle. In this study, we present an innovative 3D-IVM approach using a custom 3D matrix to enhance oocyte maturation by preventing cumulus-oocyte complex (COC) flattening and preserving its structural and functional integrity. We investigated meiotic progression in oocytes and blastocyst yield using this 3D matrix.

**Methods:** COCs were collected from cattle ovaries at a slaughterhouse (n= 50 COCs/group; with 10 replicates) and subjected to either the new 3D-IVM system or a control group using 2D-IVM for 24 hours in a basic medium. The maturation medium consisted of TCM199 with bicarbonate and Earle's salts, supplemented with recombinant follicle-stimulating hormone (FSH - 0.1 IU/mL), sodium pyruvate (22 µg/mL), amikacin (75 µg/mL), and bovine serum albumin without fatty acids (BSA; 4 mg/mL). After the IVM, matured COCs were denuded and the meiotic progress was examined under a microscope to determine the meiotic stage (Hoechst 33342 protocol). In a second experiment, the matured COCs were subjected to regular in vitro fertilization (IVF) and in vitro culture (IVC).

**Results:** In our 3D-IVM system, COCs did not adhere to the surface of the plate, enhancing their exchange surface area with the culture medium. Although the meiotic progress among 2D-IVM and 3D-IVM was not affected by the new system (*P*= 0.76; 86.45 ± 2.65 and 85.77 ± 2.08, respectively) the results suggest a brief reduction in degenerated oocytes using our 3D-IVM (*P*= 0.06; 2.30 ± 0.56; 0.81 ± 0.54, for 2D-IVM and 3D-IVM, respectively). Furthermore, our system was able to improve the blastocyst yield (*P*= 0.04; 37.20 ± 1.99) compared to the 2D system (27.73 ± 3.56), which may have a beneficial effect on early embryonic development.

**Conclusion:** The proposed 3D-IVM approach represents a groundbreaking and highly promising advancement in the field of assisted reproduction. 3D maturation offers a paradigm shift by providing a more biologically relevant and physiologically accurate microenvironment for oocyte development. As research and technology in the field advance, the adoption of 3D-IVM has the potential to revolutionize reproductive technologies, leading to increased success rates and improved fertility treatments in both human medicine and animal reproduction programs. This study was financially supported by CAPES and FAPESP- Pipe (nº 2022/06068-5).

**P-120. The role of BRCA gene mutation in infertility patients**

**Mariana Kasuga Morya^1^, Isabela Clarassoti Simionato^1^, Rafaela Pereira Lemes^1^, Gabriela Mariana Gouveia^1^, Stephanie Tasselli Alencar da Assunção^1^**

*^1^ Universidade Santo Amaro - São Paulo - SP - Brasil.*

**Objective:** The objective of the present study was to assess existing scientific evidence of the impacts of mutation in the BRCA gene and infertility.

Methods: This bibliographic review was elaborated through a search in the PUBMED database from the descriptors “fertility”; “infertility”, “BRCA” and “ovarian reserve”, from the last 5 years, which included reviews, clinical trials, and randomized controlled trials.

**Results:** Evidence demonstrates that when compared with controls, women who carry a mutation in the BRCA1 gene produced fewer oocytes 7.4 (95% CI, 3.1-17.7) versus 12.4 (95% CI, 10.8-14.2); *p* = 0.025), indicating that the BRCA gene may be related to gametogenesis. It has also been shown that their oocytes not only have a higher frequency of impaired repair mechanisms, but also accelerated DNA double strand breakage. This condition may be associated with a decreased ability to neutralize genotoxic stress, leading to an accelerated loss of ovarian reserve after the accumulation of these DNA breaks in the oocyte, and consequently, premature ovarian failure. Furthermore, after adjusting for age and BMI, significantly lower levels of anti-Mullerian hormone (AMH), a biomarker that represents a woman's reproductive competence, were found in carriers of the mutated BRCA1 gene (*P*=0.02), but not in carriers of the mutated BRCA2 gene (*P*=0.94) when compared to controls. However, the study states that low serum concentrations of AMH have not been shown to affect natural fecundity and fertility in BRCA mutation carriers below 30 years of age, affecting only older patients. With regard to preservation of fertility, it has been shown that after ovarian stimulation, carriers of the mutation in the BRCA gene had a lower number of mature oocytes and follicular reserve. It was also demonstrated that the average number of oocyte production in these patients is lower when compared to non-mutation carriers. Furthermore, studies show that BRCA mutation carriers have higher rates of poor ovarian response compared to BRCA mutation negative patients undergoing ovarian hyperstimulation. It is also important to mention that studies point to ovarian aging induced by chemotherapy, which occurs by inducing the breakage of DNA double strand repair in primordial follicular oocytes and thus triggers massive apoptotic death. Despite this, it is noteworthy that recent studies have shown that many follicles can remain free of apoptosis, suggesting that there is reversal of chemoinduction.

**Conclusion:** In conclusion, it was observed that women with a BRCA1 gene mutation produced smaller numbers of oocytes and faster double-strand DNA breaks, which resulted in loss of ovarian reserve after the accumulation of these breaks and consequently premature ovarian failure. The low concentrations of AMH have not been shown to affect natural fecundity and fertility in patients with a BRCA mutation younger than 30 years old, affecting only patients with more advanced age. Moreover, it has been shown that after ovarian stimulation, patients with a mutation in the BRCA gene have a lower number of mature oocytes and follicular reserve. However, it is important to understand the limitations of studies in healthy women with BRCA mutations, as they may represent only a small part of a larger, complex problem. Women who develop cancer as a result of a mutation in the BRCA gene and receive chemotherapy have ovaries aging due to induction of DNA double-stranded repair breakage in primordial follicular oocytes resulting in massive apoptotic death.

**P-121. The difficulties in preserving fertility in young patients diagnosed with cancer**

**Gabriela Wroblewski^1^, Isabela Clarassoti^1^, Lara Bitar Novazzi^1^, Mariana Kasuga Morya^1^, Gabriela Gouveia^1^**

*^1^ Universidade Santo Amaro - São Paulo - SP - Brasil.*

**Objective:** The aim of the following study was to evaluate the various methods used for fertility preservation in pediatric patients diagnosed with cancer and to identify the various difficulties that each intervention poses.

**Methods:** A systematic bibliographical review was carried out with a search in the PUBMED database (Medline) from the descriptors “fertility preservation”, “cancer” and “pediatrics”. Only review articles published in the last 10 years (from 2013 to 2023) were selected. Works that reported male fertility only were excluded from the study.

**Results:** Based on the data collected from the articles, it was noticeable that fertility preservation procedures, such as cryopreservation, can often cause irreversible damage to the patient's health. Cancer treatments, including radiotherapy and chemotherapy, cause late effects such as gonadotoxicity with permanent azoospermia or premature ovarian failure, resulting in difficulty conceiving or even sterility. Patients who opt for the fertility preservation process will face several challenges that can culminate in physical and emotional harm. Some of these damages include: multiple daily injections, side effects of ovarian stimulation, risks associated with anesthesia and laparoscopy, delay in starting cancer treatment and all the consequences linked to the emotional immaturity of children. The method of choice for fertility preservation depends on several factors, such as age, the chosen cancer treatment and the patient's level of maturity. In addition to leaving an excessive emotional and physical impact on patients who choose to undergo the preservation process, none of the methods offer a clinical pregnancy rate of approximately 100% for women after a gonadotoxic regimen. According to the American Society for Reproductive Medicine, oocyte cryopreservation offers a clinical pregnancy rate of only 4-12% per oocyte (or 36-61% per embryo transfer), while ovarian tissue cryopreservation offers a rate of 57%, both rates being seriously low.

**Conclusion:** The American Society of Clinical Oncology updated its guideline in 2013 to emphasize the importance of addressing gonadotoxicity and preserving fertility in all patients of reproductive potential. This is crucial when thinking about planned parenthood for cancer patients that might be interested in the future. There is a need for new research and development of guidelines that evaluate preservation methods with high pregnancy rates that provide less sequelae for patients. Although fertility preservation is recommended for all patients undergoing cancer treatment, there are still many barriers and ethical considerations to achieving preservation.

**P-122. In vitro fertilization laboratory practice for handling a large number of oocytes in Brazilian reproductive medicine clinics. Which is the most common practice and its consequences?**

**Christina Rumi Morishima^1^, Amanda Setti^2^, Nilka Fernandes Donadio^3^, Luiz Henrique Gebrim^3^, Tatiana Carvalho Souza Bonetti^4^**

*^1^ Departamento Ginecologia - Escola Paulista de Medicina - Universidade Federal de São Paulo (EPM-UNIFESP) - São Paulo - SP - Brasil.*

*^2^ Fertility Medical Group - São Paulo - SP - Brasil.*

*^3^ Setor de Reprodução Humana, Hospital da Mulher - São Paulo - SP - Brasil.*

*^4^ Departamento Ginecologia - Escola Paulista de Medicina - Universidade Federal de São Paulo (EPM-UNIFESP) - São Paulo - SP - Brasil.*

**Objective:** The increase demand for assisted reproduction techniques (ART) leads to a constant improvement in the technologies with the aim of achieving higher pregnancy success rates. The controlled ovarian stimulation (COS) sometimes results in many collected oocytes, which in turn results in a significant number of cryopreserved oocytes and/or embryos. The management of cryopreserved specimens is still uncertain, especially for embryos, and involves ethical, religious, legal, moral, financial, and personal issues. This scenario has generated a significant number of embryos cryopreserved in the ART clinics, which brings discussion leading to a series of discussions about behaviors to reduce surplus embryos. The aim of this study was to assess the profile of in vitro fertilization (IVF) laboratories regarding the handling of oocytes and surplus embryos from women undergoing IVF cycles in Brazil.

**Methods:** This study was a field research through the application of a multiple-choice questionnaire using an online survey platform (SURVIO). All ART centers, whose laboratories were registered in the National Embryo Production System (SISEMBRIO, 2018) were invited to participate. The questionnaire was developed by the research group and validated before the start of the study. The questions addressed (i) productivity and activity time of the laboratory, (ii) the technical-operational procedures, and (iii) the use and cryopreservation time of the material. The final version, composed of 72 multiple-choice questions pertinent to the understanding of laboratory conduct was sent to 94 IVF laboratories between November 2020 and May 2021. A total of 57 ART centers agreed to participate in the study, and after signature of a consenting term, answered the questionnaire. This study evaluated part of the questionnaire, focusing on technical-operational procedures of IVF laboratories, in the face of ovarian hyper-responsiveness.

**Results:** Most of the participating ART centers were private (92.9%) and about half of them (47.4%) were considered medium or large, performing at least 30 IVF cycles per month. ART clinics were asked what their concept of ovarian hyper-responsiveness was, and 76% answered that more than 15-20 oocyte collected after a COS. They then answered what was the main conduct when faced with ovarian hyper-responsiveness; 53% reported injecting all mature oocytes and vitrifying all developed blastocysts, 35% reported a fragmental practice of freezing half of the oocytes and the developed blastocysts after injecting half of the oocytes, and 9% alternate between those two protocols. We performed a review of the average cost of IVF laboratory procedures for 15 oocytes. The most common practice of injecting all mature oocytes and vitrifying all developed blastocysts has an average cost of R$4700.00. The approach of fragmental practice has an average cost approximately 40% higher (R$6700.00), while it supposedly could reduce the number of cryopreserved blastocysts.

**Conclusion:** This study showed that the most commonly used approach in managing a large number of collected oocytes is injection of all oocytes followed by cryopreservation of the developed blastocysts. Despite of that practice has a lower cost, probably results in a higher number of embryos cryopreserved. Moreover, costs may not be the only reason for this choice, but the uncertainty of the yield of oocyte vitrification may contribute to this decision. Given the relevance of this topic and the data presented, it is evident the need to update the consensus belongs conducts regarding the production and management of oocytes and embryos, considering the responsibly and benevolently the parties involved. Guidelines in reproductive medicine practices are essential. Moderating ovarian stimulation and encouraging oocyte cryopreservation are option to avoid high embryo production, besides of outlining issues such as embryo discard and abandonment.

**P-123. Morphology and embryo development day evaluation as a criteria in single euploid blastocyst transfer cycles**

**Maythe Alves Fanasca^1^, Caroline Christina Varela Brogliato^1^, Ivan Henrique Yoshida^1^, Rodrigo Angelo Souza^1^, Karla Melo Pacheco^1^, Isabella Mendes Alves^1^, Caroline Zulim Berton^1^, Emerson Barchi Cordts^1^, Caio Parente Barbosa^1^**

*^1^ Instituto Ideia Fértil de Saúde Reprodutiva - Santo André - SP - Brasil.*

**Objective:** Evaluate if embryo development day (D+5 or D+6) and its morphology impact on clinical pregnancy rates in single blastocyst transfer cycles.

**Methods:** Cross-sectional study performed from January 2019 to December 2022 including couples that went through assisted reproduction treatments associated with embryo genetic study (PGT-A) with single blastocyst transfer. Blastocysts were evaluated according to: day of development - Day 5 (D+5) or Day 6 (D+6); trophectoderm morphological score (top quality - AA, AB, BA; intermediate - BB and poor - BC, CB, CC), considering grade 3 or superior expansion. 365 embryos were studied and submitted to 3 different comparatives analysis. 1: poor quality blastocysts achieving on D+5 (n=183) versus D+6 top quality (n=43); 2: D+5 poor quality embryos (n=183) versus D+6 intermediate quality (n=30) and 3: D+5 intermediate quality embryos (n=109) versus D+6 top quality (n=43). The endpoint of this study was clinical pregnancy rate evaluation.

**Results:** The clinical pregnancy rate found when groups were compared was 45% and 50% respectively (*p*=0.754) in analysis 1; 45% and 40% (*p*=0.584) in analysis 2 and 54% and 50% (*p*=0.785) in analysis 3. There were no statistically significant differences on those analysis.

**Conclusion:** Embryo chromosomal status is still the gold standard criteria for embryo selection. When D+5 top quality euploid embryos are available, these are still the priority choice for embryo transfer. However, to couples that don’t dispose better quality blastocysts, the results obtained in this study showed no difference in clinical pregnancy rates when embryo development day is compared with poor quality embryos. The authors understand that complementary studies with higher casuistry are appropriate to solidify the statistical relevance.

**P-124. An infertile man with Kallmann Syndrome: a case report**

**Maria Eduarda Leite Simoes^1^, Ricardo Madalozo^1^, Rodrigo Moser Uliano^1^, Ivan Sereno Montenegro^1^, Isabel Durli^1^, Markus Berger Oliveira^1^, Paula Barros Terraciano^1^, Eduardo Pandolfi Passos^1^**

*^1^ Grupo de Reprodução e Farmacologia Celular (REPROFARM), Centro de Pesquisa Experimental, Hospital DE Clínicas de Porto Alegre - Porto Alegre - RS - Brasil.*

**Objective:** Kallmann Syndrome (KS) is a congenital association between hypogonadotropic hypogonadism (HH) and anosmia or hyposmia. It is a rare condition that is clinically characterized by decreased levels of testosterone, luteinizing hormone, and follicle-stimulating hormone. Additionally, this genetic disease leads to incomplete sexual maturation and the absence of secondary sexual features, such as face and body hair growth. HH is also known as central non-obstructive azoospermia, therefore, Kallmann Syndrome diagnosed men are considered to be infertile. The main objective of this case report is to describe the importance of early diagnosis of KS for the best treatment and advising and treatment of reproductive care.

**Methods:** A male patient, 39 years old, was referred in 2022 to the Infertility and Human Reproductive service in Hospital de Clínicas de Porto Alegre (HCPA) due to a male infertility factor, presenting a spermogram with azoospermia (2021). The patient was diagnosed with KS in 2012, presenting poorly developed secondary characters, hyposmia, reduced testicular volumes on ultrasound, and other classic features of HH, such as low libido and hypothyroidism. In males diagnosed with KS, success in inducing spermatogenesis is around 90 - 95% with the traditional use of gonadotropins (hCG associated or not with recombinant FSH). The patient started to use hCG associated with recombinant FSH in 2022, with a good response on hormone profile (01/20/23: Total testosterone: 927; E2 28; FSH 3,13; LH<0,12), nevertheless with the maintenance of azoospermia on the spermogram. In this way, the Assisted Reproduction team has indicated microTESE (microscopic testicular sperm extraction) surgery, which aims to recover sperms in non-obstructive azoospermia through the extraction of seminiferous tubules visualized under the microscope. The surgery was performed by our team on 02/10/2023, in both testicles under visualization with microscopy, with a multidisciplinary team (gynecologist, urologist, and embryologist).

**Results:** The surgery performed in February 2023 showed a reduced volume of testicles, with the right testicle smaller than the left side, as shown by the ultrasound exam. The microscopy surgery showed a few dilated seminiferous tubules, removed and subsequently delivered to the embryologists. It was evidenced by immotile spermatozoa in the sample, which was cryopreserved according to the protocols. A sample from both testicles was also sent for anatomopathological examination, which showed testicular parenchyma with loss of maturation in 50% of the seminiferous tubules. The patient has evolved well and without any complications postoperatively, and is still following up with the human reproductive service in HCPA.

**Conclusion:** Although rare in men (1/10.000), Kallmann Syndrome is a congenital genetic disease that represents a condition with hypogonadotropic hypogonadism (HH) that affects health and can affect fertility in many ways. This case report describes an adult with a delayed diagnosis of Kallmann Syndrome associated with male infertility, with an adequate hormone response for clinical treatment but maintenance of azoospermia in spermogram. The indication for the microTESE surgery has successfully retrieved sperm and increased the couples chance of achieving a pregnancy through in vitro fertilization. Therefore, it is essential that patients with Kallman syndrome are diagnosed early and followed up with multidisciplinary teams to have better outcomes in reproductive care.

**P-125. Pregnancy results using oocytes cryopreserved for fertility preservation: A single center’s experience**

**Mariangela Badalotti^1^, Marta Ribeiro Hentschke^1^, Isadora BadalottI-Teloken^2^, Ricardo AZAMBUJA^1^, Natália Fontoura Vasconcelos^2^, Victoria Campos Dornelles^2^, Fabiana Mariani Wingert^1^, Alvaro Petracco^1^**

*^1^ Fertilitat Centro de Medicina Reprodutiva - Porto Alegre - RS - Brasil.*

*^2^ Pontifícia Universidade Católica - PUCRS - Porto Alegre - RS - Brasil.*

**Objective:** Most data in the literature deals with IVF cryopreserved surplus oocytes, and little information is available on pregnancy results when the indication is exclusively fertility preservation. Then, this study aims to evaluate the results of IVF cycles with oocytes cryopreserved with the objective of preserving fertility.

**Methods:** Retrospective study. Patients who underwent oocyte cryopreservation (OC) for fertility preservation and later thawed the oocytes for their own use by the end of 2022 were included. Therefore, patients who inseminated and cryopreserved parts of their oocytes were excluded. Laboratory results and cumulative pregnancy rates according to age at the time of cryopreservation and the number of cryopreserved oocytes were evaluated. Student's t, Chi-square and Fisher's exact tests were used for statistical analysis, considering *p*<0.05.

**Results:** The cryopreservation cycles occurred between 2004 and 2021, and the thawing cycles between 2010 and 2022. Oocytes from 46 patients, originating from 61 OC cycles (two slow freezing cycles and 59 vitrification cycles), were thawed which resulted in 50 cycles of IVF with fresh transfer (FOT) and eight cycles of cryopreserved embryo transfer (FET). The mean age of the patients at the time of cryopreservation was 37.5 ± 2.4 years, and at thawing, 40.7 ± 3.7 years. The total number of thawed oocytes was 382; the survival rate was 80.7% (n=308) and the fertilization rate was 79.2% (n=244). In 31 cycles, the embryo culture lasted 5 days and the blastulation rate was 40.7% (77/189). The cumulative rates were 48.9% for total pregnancy, 40,0% for clinical pregnancy, and 31,1% for birth/ongoing pregnancy, with 22.2% miscarriage. The cumulative clinical pregnancy rate was 60.0% when the age at cryopreservation was < 35 years (n=5), 47.6% when between 35-37 years (n=21), and 29.4% when between 38-40 years old (n=17); patients with >40 years (n=3) did not get pregnant (*p*=0.444). The cumulative clinical pregnancy rate was 45.8% when 4 to 9 eggs were frozen and 45.5% when 10 to 15 eggs were frozen; patients with <4 oocytes did not get pregnant. Four patients who did not become pregnant still have eight cryopreserved embryos.

**Conclusion:** The search for OC to preserve fertility has been growing, both in cancer patients and mainly, due to the delay of motherhood. In our experience, the number of procedures has quadrupled in the last decade, with a growth of 20% per year in the previous 5 years. However, the number of cases of patients who sought the clinic to try to conceive with their oocytes is still low. But, even so, these results suggest that patients can freeze their eggs even after 35 years old but before 40, and have more than three frozen eggs. This data shows the importance of offering patients such an opportunity.

**P-126. Selective serotonin reuptake inhibitors (SSRIs) and their influence on male fertility**

**Raisa Arruda de Oliveira^1^, Suellen Casado dos Santos^2^, Vera Lúcia de Menezes Lima^2^, Luana Nayara Gallego Adami^1^**

*^1^ Embriológica - São Paulo - SP - Brasil.*

*^2^ Universidade Federal de Pernambuco - Recife - PE - Brasil.*

**Objective:** We aimed to discuss the main findings related to adverse effects of SSRI drugs on male fertility, considering hormone levels, sexual function, seminal parameters and sperm function.

**Methods:** This study is a systematic review about the research question: "What adverse effect(s) and influence(s) do the main SSRI have on male fertility?". Search strategy included: (male infertility) OR (male fertility) AND (serotonin uptake inhibitor) OR (antidepressant agent) OR (sertraline) OR (fluoxetine) OR (paroxetine) OR (citalopram) OR (escitalopram) OR (fluvoxamine) OR (vilazodone) AND (semen analysis) OR (DNA damage) OR (spermatogenesis) OR (sexual dysfunction), with addition of filters from the last ten years and types of studies for randomized controlled trial, clinical study, clinical trial, case report, prospective study, retrospective study, animal experiment and systematic review. The databases chosen were EMBASE and LILACS/BVS. For the selection of studies, the inclusion criteria were carried out, considering the English language, studies that were exclusively with the main drugs of the class of the SSRI, work done with mice and rats, as well as studies with humans. In terms of exclusion, other alternative models, literature reviews, editorials, as well as other drugs in the class that were not in the search strategy were disregarded.

**Results:** A total of 125 studies were found (8 LILACS/BVS and 117 EMBASE), 7 of which were considered duplicates by PICOportal. By screening the reading of the abstract, by two independent researchers, 18 were included and 100 were excluded, following the exclusion and inclusion criteria. Finally, for full text, 13 publications were considered. All analyzes followed the PRISMA Statement 2020. Of the compiled articles, vilazodone, fluvoxamine and escitalopram were not cited, most studies point to fluoxetine as the drug most used in research on the subject. Most studies are experimental in mice or rats. The authors worked with biochemical markers, comparing them with the main drugs chosen in the present research, in a way that demonstrated gonadotoxic effects, ejaculatory dysfunction, failures in spermatogenesis, in morphology, decrease in sperm production and progressive motility, in addition to questioning sertraline, fluoxetine, citalopram and paroxetine, as possible oxidative stressors. However, other studies have pointed out that the use of SSRIs do not affect sperm DNA, on the contrary, they would act as antioxidants, bringing variability and making it difficult to reach a real consensus on the influence of these therapies. The use of extracts or natural products can corroborate the decrease in the impact caused by paroxetine, thus being relevant for the control of hormonal parameters and the adverse effects caused by SSRIs. In addition, the dosage of drugs is quite varied, but all claim that high doses of SSRIs decreased hormone levels (LH, FSH, Testosterone). The use of those causes even greater effects with increasing age.

**Conclusion:** Considering that this drug therapy may have a longer duration, their effects on fertility need to be considered. On the other hand, even though studies point to adverse effects of the use of SSRIs, more randomized and experimental clinical research is needed to evaluate the mechanisms of spermatogenic failure, as well as to investigate the criteria underlying these effects, in male fertility. Proposing tools for therapeutic monitoring of these drugs in the seminal fluid, or other parameters, can be good allies in the investigation, minimizing the possible risks.

**Table 1:** Comparison between Groups 1 and 2, according to KIDScore, and clinical parameters.

	G1 (KIDScore <7) n=32	G2 (KIDScore ≥7) n=34	p
KIDScore	5.2±1.4	8.1±0.7	-
MALE AGE (years)	40.6±8.5	39.2±5.7	0.440
OOCYTE AGE (years)	28.9±5.7	30.4±6.2	0.307
GESTATIONAL AGE (weeks)	37.4±2.2	37.2±2.1	0.703
BIRTH WEIGHT (g)	3158.0±530.4	2873.7±598.5	**0.047**
BIRTH LENGTH (cm)	47.7±3.3	47.6±2.5	0.911
5`APGAR	9.4±0.6	9.3±0.8	0.639
PREMATURITY	6/32 (18.8%)	11/34 (32.4%)	0.207

**Table 2:** Comparison between Groups 1 and 2, according to KIDScore, and weight percentile.

	G1 (KIDScore <7) n=32	G2 (KIDScore ≥7) n=34	p
WEIGHT PERCENTILE SGA AGA LGA	1/32 (3.1%) 30/32 (93.8%) 1/32 (3.1%)	8/33 (24.2%) 25/33 (75.8%) 0/33 (0%)	**0.032**

SGA: small for gestational age; AGA: adequate for gestational age; LGA: large for gestational age.

**P-127. Automatic embryo scoring using the KIDscore algorithm and neonatal outcomes**

**Marta Ribeiro Hentschke^1^, Victoria Campos Dornelles^2^, Ricardo Azambuja^1^, Fabiana Mariani Wingert^1^, Isadora Badalotti-Teloken^2^, Natalia Fontoura Vasconcelos^2^, Alvaro Petracco^1^, Mariangela BadalottI^1^**

*^1^ Fertilitat Centro de Medicina Reprodutiva - Porto Alegre - RS - Brasil.*

*^2^ Pontifícia Universidade Católica - PUCRS - Porto Alegre - RS - Brasil.*

**Objective:** Time lapse technology is bringing new perspectives to the relationship between embryos' morphokinetics and implantation rates after assisted reproduction techniques (ART). The KIDscoreTM D5 (KS5^®^) algorithm has reported that a higher KIDScore is related to higher euploid embryos, which could lead to higher chances of implantation. However, data relating neonatal outcomes and the KS5 algorithm are scarce. Thus, the objective of the present study was to analyze if there is any association between KIDScore and neonatal outcomes.

**Methods:** Retrospective observational study performed at a reproductive medicine center, using data collected between 2020 and 2022. A total of 66 live births resulting from 197 single fresh blastocysts transferred cycles were included for analysis. All embryos were cultivated in a time-lapse incubator (Embryoscope^®^, Vitrolife^®^) and received a KS5. The sample was stratified by KS5: G1 (1-6.9) and G2 (7.0-9.9). T-test and chi-square test were used for analysis, considering *p*<0.05.

**Results:** Results regarding the comparison between G1 vs. G2, are presented in Table 1 and table 2.

**Conclusion:** It was observed that when the KS5 was equal or higher than 7, the birthweight was lower, and the percentage of SGA was higher. However the lower birth weight may have other parameters related like endometrium, maternal endothelial disease, and so, the data could be better analyzed with large samples and maternal characteristics included. Besides the birthweigh and percentile, there were no longer differences between groups.

**P-128. The role of the father in IVF treatment begins prior to conception**

**Victoria Barchi Cordts^1^, Giovanna Pires de Lima Martins^1^, Gabriel Monteiro Pinheiro^1^, Gabriela Gouveia^1^**

*^1^ UNISA - São Paulo - SP - Brasil.*

**Objective:** The objective of this present study is to understand the father's role in IVF, if it should begin even before conception and how it impacts the treatment´s outcomes.

Methods: Eighteen articles on the topic were analyzed. Out of these, eight were chosen for portraying the man's experience during the IVF treatment more effectively. This study is a narrative review of articles published in English and Portuguese on PubMed, Scielo and assisted reproduction journals between 2006 and 2020. Works that do not report male involvement and gestational success were excluded. The keywords mentioned were: fatherhood, gestational success, success rate in IVF, and IVF.

**Results:** Based on the data collected from the chosen articles, it was noticeable that the most discussed topic among men in couples undergoing IVF treatment was the feeling of loss of masculinity, especially when infertility is caused by a male factor. Besides that, male and female participants acknowledged the sensitive nature of fertility as a women's health issue, posing challenges for men to discuss. This underscores the need for increased male involvement in fertility studies to improve our understanding of male factors and promote education and communication on male fertility and reproductive health. Petterson B. D, et Al, 2006, concluded that women use more coping strategies than men, and distancing as a coping strategy was related to decreased infertility stress in both men and women and decreased marital adjustment for men. Furthermore, female and male infertility stress was explained by participants coping strategies related to decreased infertility stress, such as seeking social support and planful problem-solving. These coping strategies should be identified and encouraged. D.K. Ondieki, et Al, 2015, concluded through his study that of the 163 women enrolled in the study, 114(69.9%) were ever accompanied while 30.1% (49) were never accompanied by their spouses. Of the ever accompanied women, 24(14.7%) were always accompanied. The unaccompanied women give as a reason for their spouses not attending the fertility clinic that their male partners were busy. Most of the women, 158(96.9%), wanted their spouses to accompany them to the clinic. Besides that, all the men 34 (100%) and all the women 163 (100%) felt that male participation would add value to the overall couple's care.

**Conclusion:** Since ancient times, women have been revered for their ability to bring another being into the world and provide them with sustenance through a special nourishment that only they could provide: breast milk. The notion of a man with constant erectile ability and unending sexual desire is a myth that appears in tales such as the Labors of Hercules and the famous harems that perpetuated the association of virility with the ability to conceive children (fertility). As male chauvinism remains deeply ingrained in our society, despite causing half of infertility difficulties, many men don't actively join the treatment process. Consequently, cultural and gender norms reinforce the notion that pregnancy is predominantly a woman's domain and discourage men's active engagement in the preconception process. Given that a significant number of studies demonstrate that partner support plays a crucial role in reducing fertility related stress, it is essential that fertility clinics prioritize involving men in procedures, since their involvement during fertility treatment has been shown to have positive impacts on IVF outcomes.

**P-129. The role of in vitro gametogenesis from pluripotent stem cells in assisted human reproduction**

**Jamille Ribeiro de Santana^1^, Andrea Giannotti Galuppo^2^, Jonathas Borges Soares^2^**

*^1^ Escola Bahiana de Medicina e Saúde Pública - Salvador - BA - Brasil.*

*^3^ Projeto Alfa - São Paulo - SP - Brasil.*

**Objective:** The objective was to analyze the role of in vitro gametogenesis (IVG) from the differentiation of pluripotent stem cells as a support tool for assisted human reproduction procedures.

**Methods:** An integrative literature review was carried out considering the last 5 years, from the following databases: National Library of Medicine National Institutes of Health of the USA (PubMed), Virtual Health Library (BVS), Latin American and Caribbean Literature/ Bibliographic Index Español en Ciencias de la Salud (Lilacs) and Medical Literature Analysis and Retrieval System Online (Medline). Experimental and opinion articles were selected, only in English and with IVG as their central theme. From the analysis of the 17 articles selected, it was identified that much has been studied since the first report of success in obtaining in vitro gametes derived from mouse embryonic stem cells.

**Results:** This process was initially described in 2011 by Professor Katsuhiko Hayashi's group and was called in vitro gametogenesis (IVG). Gametes can be produced *in vitro* from two different cell types; they can be derived from induced pluripotent stem cells (iPSCs) or pluripotent stem cells (ESCs) derived from cloned embryos or embryos produced by IVF. Regarding its clinical use, three broad applications of IVG can be distinguished: in heterosexual couples, homosexual couples, and individual reproduction. Specifically in the case of using iPSC, it would be possible to generate functional autologous gametes from the couple's somatic cells, eliminating the need to resort to a gamete or embryo bank. It was also described that iPSCs use for IVG have limitations, there are findings that iPSCs retain residual epigenetic memory, typical of parental somatic cells, which can lead to a bias in their propensity to differentiate into different lineages. However, one of the main challenges of IVG is the reconstitution of the complex processes that involve gametogenesis, culminating in the production of a viable and functional sperm or oocyte. Also, before reaching the threshold of differentiation of iPSCs or primordial germ cells, it is necessary to be able to evolve *in vitro* growth and in vitro maturation of gametes. Furthermore, the development of gamete production *in vitro* brings with it, in addition to helping infertile couples, the possibility of genetic manipulation of these cells. It would be possible to select cells free of genetic diseases, specific characteristics of the offspring, in addition to use in research such as analysis of the differentiation process of embryonic stem cells, X chromosome inactivation, fertilization, early embryonic development, germ cell tumors and gene editing (CRISPR/Cas9). This wide range of possibilities raised questions about the idea of applying regenerative and reproductive medicine to humans in practice and brought legal and ethical issues into focus.

**Conclusion:** IVG is a technique capable of reconstituting spermatogenesis or oogenesis in vitro from iPSCs, promoting their differentiation into viable gametes, which have the same genetic as the infertile patient. The gametes can be fertilized using IVF techniques and do not require the donation of gametes and embryos, making it possible to have a child with a genetic from both partners. It is a promising methodology that may be used as a tool in the treatment of infertility, particularly in cases of germ cell aplasia.

Therefore, the development of IVG may help in understanding not only gametogenesis, but also factors related to infertility and genetic diseases. Although it is a promising technique for both reproductive and regenerative medicine, many discussions have been held about the ethical aspects and social repercussions of this technique, such as the possibility of gene manipulation.

**P-130. Comparative analysis of embryo classification by KIDscore using seminal parameters**

**Ana Karoline Machado Rosa^1^, Ricardo Azambuja^2^, Fabiana Mariani Wingert^2^, Marta Ribeiro Hentschke^2^, Victoria Campos Dornelles^3^, Isadora Badalotti-Telöken^3^, Alvaro Petracco^2^, Mariangela Badalotti^2^, Claudio Telöken^1^**

*^1^ Universidade Federal de Ciências da Saúde de Porto Alegre - Porto Alegre - RS - Brasil.*

*^2^ Fertilitat Centro de Medicina Reprodutiva - Porto Alegre - RS - Brasil.*

*^3^ Pontifícia Universidade Católica - PUCRS - Porto Alegre - RS - Brasil.*

**Objective:** To evaluate seminal parameters according to stratified Kidscore groups.

**Methods:** A retrospective cohort study was performed in a Reproductive Medicine Center from January 2020 to December 2022. The embryos (n=3437) were cultivated in a time-lapse incubator (Embryoscope^®^, Vitrolife^®^) and were stratified into three groups according to Kidscore: G1 (1.0 - 4.0), G2 (4.1 - 7.0) and G3 (7.1 - 9.9). Male and seminal parameters (age, concentration, morphology and motility) were analyzed between groups. Frozen oocytes and preimplantation genetic testing (PGT) embryos were excluded as well as all the percutaneous epididymal sperm aspiration (PESA) and testicular sperm aspiration/extraction (TESA/TESE). The data were presented as mean and standard error (SE). ANCOVA test was applied, and data were adjusted for female age.

**Results:** The comparative analysis of seminal parameters between the three groups are shown in Table 1.

**Conclusion:** When the seminal parameters were compared according to the embryo score, no statistical difference was observed. Thus, in this study, seminal parameters seem not to have an impact on embryo quality evaluated through time lapse. More studies considering other variables should be performed for further conclusions regarding the male impact possibility on embryo quality.

**P-131. Seminal parameters in single fresh embryo transfers: are they better in positive clinical pregnancy?**

**Ana Karoline Machado Rosa^1^, Ricardo Azambuja^2^, Fabiana Mariani Wingert^2^, Marta Ribeiro Hentschke^2^, Victoria Campos Dornelles^3^, Isadora Badalotti-Telöken^3^, Alvaro Petracco^2^, Mariangela Badalotti2, Claudio Telöken^1^**

*^1^ Universidade Federal de Ciências da Saúde de Porto Alegre - Porto Alegre - RS - Brasil.*

*^2^ Fertilitat Centro de Medicina Reprodutiva - Porto Alegre - RS - Brasil.*

*^3^ Pontifícia Universidade Católica - PUCRS - Porto Alegre - RS - Brasil.*

**Objective:** To evaluate the association between seminal parameters and the presence of pregnancy in single fresh embryo transfers.

**Methods:** A retrospective cohort study was performed in a Reproductive Medicine Center from January 2020 to December 2022. A total of 194 single fresh embryo transfers were evaluated. The embryos were cultivated in a time-lapse incubator (Embryoscope^®^, Vitrolife^®^) after Intracytoplasmic sperm injection (ICSI). The samples were divided into two groups (pregnancy versus non-pregnancy). Seminal parameters (sperm motility, concentration, morphology abnormal and abnormal head, midpiece and tail morphology) were analyzed between groups. Frozen oocytes and preimplantation genetic testing (PGT) embryos were excluded as well as all the percutaneous epididymal sperm aspiration (PESA) and testicular sperm aspiration or extraction (TESA/TESE). The data were presented as mean and standard error (SE) and as mean and standard deviation (SD). ANCOVA and Student t tests were applied, and data were adjusted for female age.

**Results:** The mean male age according to pregnancy versus non-pregnancy was, respectively, 39.7±6.2 and 39.9±6.9 years (p=0.823). When comparing seminal parameters in both groups, no statistical difference was found. The comparative analysis of seminal parameters between the two groups are shown in Table 1.

**Conclusion:** When the maternal age is adjusted, none of the seminal parameters seem to influence the pregnancy outcomes. Perhaps the low number of embryos transferred have limited the outcome of the results. More studies with larger samples should be conducted in order to identify any possible significant difference.

**Table 1:** Comparative analysis of seminal parameters according to Kidscore groups.

Blastocysts (n=3437)	G1 (1,0 - 4,0)	G2 (4,1 - 7,0)	G3 (7,1 - 9,9)	p-value^*^
**Male age, Yo**	39.1±0.12	39.1±0.13	39.1±0.21	0.951
**Sperm motility,%**	53.5±0.50	53.1±0.48	51.7±0.77	0.135
**Concentration (million/mL)**	58.3±1.42	58.7±1.36	60.0±2.19	0.804
**Abnormal Morphology, %**	95.4±0.25	95.5±0.23	95.8±0.36	0.660
**-Abnormal head, %**	47.2±0.82	45.9±0.74	44.8±1.18	0.231
**-Abnormal midpiece,%**	27.0±0.50	27.5±0.45	27.7±0.72	0.686
**-Abnormal tail,%**	21.2±0.69	22.1±0.62	23.4±0.99	0.181

^*^Ancova. SE, Standard Error.

**P-132. The presence of endometriomas is associated with post-oocyte retrieval complications in patients undergoing IVF treatment**

**Larissa Matsumoto^1^, Nathalia Reggi Reis Silva Malandrino^1^, Sergio Conti Ribeiro^2^, Thais Tiemi Higa^3^, Renato Bussadori Tomioka^1^**

*^1^ Clínica VidaBemVinda - São Paulo - SP - Brasil.*

*^2^ Hospital Sírio Libanês - Sao Paulo - SP - Brasil.*

*^3^ LabForLife - Sao Paulo - SP - Brasil.*

**Objective:** This review aims to evaluate whether the presence of endometriomas is a risk factor for post-oocyte retrieval complications, such as pelvic infections, vaginal or peritoneal bleeding, and pelvic pain in women undergoing in vitro fertilization (IVF) treatment for infertility.

**Methods:** This literature review covers 55 original articles, case reports and reviews in English published on Pubmed between January 1983 and December 2022. The MESH terms used for the search were: endometriosis, endometrioma, oocyte retrieval, hemoperitoneum, pelvic infection, pelvic inflammatory disease, vaginal bleeding, peritoneum injuries, and postoperative complications.

**Results:** Anatomical distortion may impair oocyte retrieval in patients with endometriomas. In one of the studies included it was found that incomplete aspiration of the follicles occurred in 14% of women as a result of limited needle access to the ovaries. Pelvic pain in women with endometriomas is one of the most common complications after the oocyte pick-up. In another study, the percentage of women with pain that needed to go to hospital was 7%. Endometriosis and the presence of an endometrioma are associated with a higher risk of post-oocyte retrieval pelvic infection, as a consequence of various factors: immunological changes, the fact that endometriomas are an excellent culture medium, the frequency of endometrioma puncture and pelvic adhesions. Most critically, endometrioma puncture during the oocyte retrieval is the risk factor more associated with pelvic infection post-egg retrieval. In this context, endometriomas larger than 27 mm were inadvertently punctured in 22% of cases, and in patients with previous laparoscopic approaches, this percentage increased to 24%. Pelvic infection after transvaginal oocyte retrieval occurred in 0.2-0.5% of the cases. In 14 endometrioma infections cases in the literature, 11 of them received antibiotic therapy after the procedure, as prophylaxis, and 7 had the endometrioma accidentally punctured during the retrieval. Vaginal bleeding is one of the most common complications after oocyte retrieval, mild cases could occur in 18.8% and be solved with local compression. Its main risk is associated with the intensity of pressure exerted by the endovaginal ultrasound probe. Peritoneal bleeding occurs less frequently, just in 0.2/1000 to 0.5/1000 of the oocyte retrievals. Therefore, the presence of endometriomas was not associated with vaginal and/or peritoneal bleeding. Previous laparoscopic procedures could be a risk factor for hemoperitoneum in women with endometriosis, together with ovarian scars. In a case series report that included 54 patients with post-oocyte retrieval pelvic bleeding, 33.33% reported having had laparoscopic surgery before the IVF treatment.

**Conclusion:** Therefore, endometriosis and the presence of endometriomas are risk factors for pelvic infection after ultrasound-guided oocyte retrieval, but the literature does not suggest they are risk factors for peritoneal and vaginal bleeding.

**P-133. Enhancing IVF Success Rates: Exploring the Efficacy of Acupuncture and the Paulus Protocol**

**Eliana Junko Morita^1^, Raquel Mitie Mitie Koike^1^, Mary Clea Zip Lem Gun^1^, Barbara Arrivetti de Andrade^1^, Amanda Altoé Satlher^1^, Suellen Santos Mendes^1^, Pedro Avanci Fontenele^1^, Gabriela Marcilio^1^, Alexandre Akio Takara Gusukuma^1^, Edson Guimarães Lo Turco^2^, Fernando Prado^3^, Diana Caroline da Silva Bastos^1^, Rafaela Aguiar^1^**

*^1^ CITI Hinode - SP - Brasil.*

*^2^ Departamento de Ginecologia, UNIFESP - SP - Brasil.*

*^3^ Neo Vita - SP - Brasil.*

**Objective:** Acupuncture, an ancient Chinese medicine technique, has been integrated into human reproduction treatments. Studies suggest that combining acupuncture with traditional in vitro fertilization (IVF) procedures may enhance success rates. Acupuncture activates the autonomic nervous system, promoting better blood circulation to reproductive organs and regulating the hypothalamic-pituitary-ovarian axis through the sympathetic nervous system. Neurotransmitter release, such as serotonin and gamma-aminobutyric acid (GABA), also induces anxiolytic effects, reducing stress during treatment. These factors create a favorable uterine environment for embryo implantation and increase the likelihood of clinical pregnancy. The renowned Paulus Protocol, developed by Dr. Wolfgang Paulus and his team, involves two 25-minute acupuncture sessions (pre and post embryo transfer) during IVF treatment. Sterile, disposable needles and stainless materials are used in specific acupoints, aiming for the "Deqi" sensation (pain, numbness, or distension). Pre-embryo transfer points include PC6, SP8, LR3, GV20, and S29, while post-embryo transfer points are S36, SP6, SP10, and LI4. Additionally, the following ear acupuncture points were placed: ear point 55 (ShenMen), ear point 58 (Zhigong), earpoint 22 (Neifenmi) and earpoint 34 (Naodian). This study seeks to demonstrate the impact of acupuncture and its potential benefits in IVF treatment.

**Methods:** A retrospective study was conducted at CITI Hinode, São Paulo, analyzing 284 embryo transfer cycles between January 2022 and June 2023. The in-house team of physicians (gynecologists and acupuncturist) assessed the data. The acupuncture group (141 patients) followed the Paulus Protocol, while the control group (143 patients) received embryo transfer without adjuvant treatment. SPSS 26 software, chi-square test, and T-test analyzed the data for differences between groups. A P value < 0.05 indicated statistical significance. Criteria evaluated: patient age, embryo quality percentage, transferred embryo count, pregnancy rate, embryonic loss rate, and evolutionary pregnancy rate.

**Results:** No notable age differences were observed between patient groups Controls vs acupuncture (40.17 ± 4.65 and 40.02 ± 6.02, p = 0.800), along with no significant variations in the number of transferred embryos (1.37 ± 0.53 and 1.38 ± 0.50) and embryonic quality (81.8%± 38.8 and 81.8%± 38.8) (*p* = 1.000). Analyzing plasma B-hCG on the 9th day after transfer, positive results were seen in 76.6%±42.5 with acupuncture and 67.1%±47.1 in controls, but the difference lacked statistical significance (*p* = 0.076). Although, the gestational losses not present significantly difference between groups, 13.0%± 32.7 in acupuncture and 25.0%± 43.5 in controls, the Paulus Protocol acupuncture during embryonic transfer showed significant benefits for evolutionary pregnancy 87.7%± 33.0 in acupuncture and 74.2%± 44.0 in controls, indicated by the presence of a gestational sac and embryo with heartbeat on ultrasound, compared to the controls.

**Conclusion:** Acupuncture, particularly the Paulus Protocol, shows promise in enhancing assisted human reproduction success. Analyzed data reveals positive correlation, boosting IVF success rates, reducing losses, and increasing evolving pregnancies. Larger, rigorous studies will further validate acupuncture's benefits in IVF treatment.

**P-134. Impact of SARS-CoV-2 on seminal parameters of men treated at an Assisted Reproduction Center in Rio Grande do Norte, Brazil**

**Fernanda Karoline Oliveira^1^, Amanda Domingues Fonseca^1^, Danielle Barbosa Morais^1^**

*^1^ Universidade Federal do Rio Grande do Norte - Natal - RN - Brasil.*

**Objective:** The objective of this study was to know the impacts of SARS-CoV-2 infection on the seminal parameters of men undergoing assisted reproduction treatment at a maternity school, in a public assisted reproduction center located in Rio Grande do Norte, Brazil.

**Methods:** After approval by the ethics committee of Universidade Federal do Rio Grande do Norte (number 64207622.5.0000.5537), men who reported having received a positive diagnosis for COVID-19, before or after starting treatment at the assisted reproduction center of Maternidade Escola Januário Cicco (Natal-RN), between the years of 2022 to 2023, were invited to participate in this research. Data were collected from medical records and spermograms of 50 men who agreed to participate in the research, by signing the informed consent form. The medical records were analyzed and important data collected, such as: 1. socio-economic profile of the patient; 2. sperm parameters; 3. reported sperm alterations. The data analysis used a case-control design, in which the control group data was performed according to WHO laboratory manual 6th ed. for the examination and processing of human semen.

**Results:** A total of 50 patients were included with a mean age ± SD (range) of 36.7 ± 8.94 (29-50) years. Among these, 64% (32/50) performed physical activity one or more times a week. At the beginning of treatment, 80% had no children and 20% had 1 or more children. Regarding sperm parameters, 52% (26/50) had a mean liquefaction time of 20 minutes, 10% (5/50) with a mean time of 40 minutes, 2% (1/50) with 45 minutes and 36% (18/50) with a mean time of 60 minutes. According to WHO, this process usually occurs until 20 minutes, and finishes until 60 minutes after semen collection. The mean semen volume was 2.9 ml, in which 36% (18/50) samples presented volume between 0.5 to 1.5 ml and 16% (8/50) presented volume above 5.0 ml. According to WHO laboratory manual, 1.5 ml is the minimum acceptable value for normospermia, while values above 5 ml characterize hyperspermia. The presence of leukocytes (10^6/ml) was on average 0.1% for the 50 samples, being within the expected value. Linear progressive motility (LPM) value was on average 19%, non-linear progressive motility (NLPM) showed a mean value of 20%, and non-progressive motility (NPM) showed mean value of 4.9%. According to WHO, a value above 32% of LPM + NLPM, and above 40% of PM + NPM is preferred for normal fertility. In this study, respectively, 52% (26/50) and 56% (28/50) of the patients showed sperm motility below the expected values for the sum of LPM + NLPM or PM + NPM, characterizing asthenozoospermia. Immotile spermatozoa value was on average 21%. As for morphology, 22% (11/50) of the sperm had less than 4% of normal morphology. According to WHO, the lower reference limit is 4%. Of these, 65% (33/50) had no other associated diseases and 35% (17/50) had associated diseases, varicocele being the most common (8/17).

**Conclusion:** It can be concluded that the exposition of SARS-CoV-2 can affect the seminal parameters by reducing the expected values for normospermic men, especially on sperm motility. The existence of previous pathologies does not seem to influence the severity of the condition. This study therefore reinforces the importance of post-COVID-19 seminal evaluation, aiming to increase pregnancy rates in infertile patients, or with marital infertility.

**P-135. Pregnancy after treatment of hyperprolactinemia: a case report**

**Maria Eduarda Motta Pagnoncelli^1^, Mariana Cleffi Alves^1^, Gabriela Gouveia^1^, Gabriel Monteiro Pinheiro^1^**

*^1^ UNISA - SÃO PAULO - SP - Brasil.*

**Objective:** The aim of this study was, in general, to document a case of idiopathic hyperprolactinemia associated with a desire to become pregnant. Thus, the relationship between hyperprolactinemia and pregnancy and the possible risks of treating this pathology during pregnancy were evaluated; we sought to understand which conduct is safer and more appropriate for patients with this condition.

Methods: This research is a quantitative case report study with a descriptive observational design. For this study, the patient's medical record was studied, attended at the Dr. Wladimir Arruda, during the years 2022 and 2023. Thus, the results obtained were analyzed and discussed in association with the current literature.

**Results:** Hyperprolactinemia is a pathology associated with the presence of supraphysiological levels of prolactin that can lead to changes in the menstrual cycle, reduced libido, galactorrhea and even infertility. Since it is considered drug treatment, it is known that cabergoline is the most used medication, due to its effectiveness and excellent tolerability. However, when dealing with women who wish to become pregnant, the evaluation must be very careful, since there is no clear recommendation for the treatment of idiopathic hyperprolactinemia. At first, when carrying out a thorough analysis of the current literature, it was seen that most of the research carried out regarding the use of cabergoline in pregnant women and/or women with a desire to become pregnant, did not demonstrate the appearance of complications arising from the use of this medication, both in the pregnant woman and in the fetus; concluded that there was not a higher frequency of complications such as abortions, Congenital Abnormalities, among other complications. In the present case, patient X, 24 years old, arrived at the outpatient clinic in August 2021, complaining of galactorrhea and unsuccessful attempts to conceive for three years, since the diagnosis of primary infertility due to idiopathic hyperprolactinemia, a treatment with cabergoline was started; despite having difficult-to-control serum prolactin levels throughout the consultations, the patient managed to get pregnant approximately one year after her first consultation. Thus, she continued using the medication until the 12th week of pregnancy; during pregnancy, she has high levels of blood glucose for large period, but she did not use any control medication. In March 2023, the pregnancy was completed, at 33 weeks and 3 days; the patient was admitted with decompensated blood glucose and non-reassuring cardiotocography, but continued with an uneventful cesarean delivery; considering the data of the newborn, in addition to being premature, he presented an newborn vitality index (APGAR) of 9 at the fifth minute and progressed with respiratory distress syndrome and hypoglycemia at birth, after 16 days in hospital he advanced with weight gain and was discharged. Finally, in the last consultation, in June 2023, she was still breastfeeding and both she and the baby, on physical examination, did not present any abnormality and, when questioned, did not report any complaints relevant to the case.

**Conclusion:** In the present case, it was seen that, despite the resolution of the infertility complaint after the use of cabergoline, there were external gestational intercurrences (gestational diabetes) that hindered an accurate assessment regarding the safety of treatment with this medication in pregnant women. On the other hand, in the other analyzed studies, most of them concluded that the drug under discussion is not the responsible for the presence of gestational and neonatal intercurrences. Finally, it is worth emphasizing the need for further studies to elucidate, standardize and firmly validate a medical approach in the face of this situation.

**P-136. The Scope and Limits of the Psychologist and the Nurse in IVF Cases with the Use of Heterologous Oocytes**

**Fernanda Kunrath Robin^1^, Flávia Giacon^2^**

*^1^ Nilo Frantz Medicina Reprodutiva - Porto Alegre - RS - Brasil.*

*^2^ Mater Prime Clínica de Reprodução Humana - São Paulo - SP - Brasil.*

**Objective:** The main objective of this research is to understand the role of nurses and psychologists in assisted human reproduction treatments that are carried out from heterologous oocytes.

**Methods:** A bibliographic review was carried out using the Medline and Scielo databases. The following descriptors were used: egg donation, nurse, and phycologist. The search was carried out as follows: egg donation and nurse or phycologist. As inclusion criteria, it was determined that the research would only be carried out by scientific articles from the last ten years that had been written in Portuguese, English, or Spanish. As an exclusion criterion, articles whose full texts could not be accessed were removed. In Medline, 07 articles were obtained, and in Scielo, 01 article.

Results: After a rigorous reading of the articles, it is clear that none of them covers the topic researched, especially concerning the care provided by nursing and psychology professionals, in an attempt to understand their roles during these treatments. We point to the importance of conducting research related to the theme of this work, considering interdisciplinarity as a promoter of the well-being of the couple and/or person receiving heterologous oocytes.

**Conclusion:** As experts in the field, we contacted in daily practice, the importance of broadening the discussion of the subject with the interdisciplinary team, considering that this should pay attention to the provision of excellent care to patients receiving heterologous oocytes. The team must be attentive to the specific demands of each patient, offering them comprehensive care, and considering aspects of their physical and emotional health. Finally, we emphasize the importance of defining the roles of both the nurse and the psychologist in cases of IVF with the use of heterologous oocytes. This definition provides more clarity for all members of the multidisciplinary team, which significantly contributes to the entire process. This lack of definition of roles may represent a gap and open the way for clinics to interfere with these functions and role delimitations. Delimitations must be completed by the referred professionals, based on their codes of ethics and what is incumbent on the exercise of their profession, defining from there, the scope and limits of their performance.

**P-137. Is embryo morphology at day 3 or at the day of biopsy an important point to pay attention in PGT-A cycles?**

**Sabrina Maria Rodrigues Jacinto Costa^1^, José de Melo Bomfim Neto^1^, Thamara Matos dos Santos^1^, Iuri Donati Telles de Souza^1^, Andrea Porto Pinheiro Telles^1^**

*^1^ Fertilità - Aracaju - SE - Brasil.*

**Objective:** Every blastocyst could be biopsied, but that does not mean that they all should. Discussions about the decision point about performing or not a biopsy in certain embryos is still up for debate, and there is not a consensus among countries, clinics, and even among physicians and embryologists of the same practice. The objective of this retrospective case-control study was to analyze the morphology of embryos at day 3 and on biopsy day, and compare these observations between euploids and aneuploids blastocysts.

**Methods:** For this study, 125 cycles of IVF and PGT-A were enrolled, analyzing a total of 458 blastocysts. Two groups were defined depending on embryo ploidy: euploid group, with 163 blastocyst, and aneuploid group with 295 embryos. With the purpose of understanding the impact of morphology and days of culture, and to evaluate the possibility of a ploidy prediction at different stages of embryos, the development and morphology of these embryos were assessed at day 3, 5, 6 and 7 or until the biopsy was performed. For day 3 embryos, the quality was determined by using a grade classification where grade 1 (G1) contemplated embryos with 8 cells, < 10% of fragmentation, symmetric cells and without multinucleation; grade 2 (G2) was composed by embryos with 6-7 or 9-10 cells, 10-25% of fragmentation, asymmetric cells and without multinucleation; and grade 3 (G3) contemplated embryos with < 6 or > 10 cells, > 25% fragmentation and asymmetric and multinucleate cells. The day of biopsy, as well as trophectoderm and ICM morphology, were assessed following Gardner classification. Blastocysts were split regarding morphology; good quality embryo (embryos with grade A and/or B) and poor-quality embryos (when ICM or trophectoderm received a grade C). Data were compared using Chi square and Fisher’s exact test with p value ≤ 0.05 considered significant.

**Results:** On day 3 of culture, for the euploid group (n= 163 embryos), 42.94% embryos were classified as G1, 45.40% were G2 and 11.66% were G3. Among aneuploid embryos (n=295 embryos), 39.66% were in G1, 45.42% in G2 and 14.92% in G3. No alterations were detected on day 3 embryos regarding their morphology and ploidy. As expected, most biopsies were performed on day 6 (76.07% and 77.97% in euploid and aneuploid groups, respectively), in comparison to day 5 (19.63% and 14.58% in euploid and aneuploid group, respectively) and day 7 (4.29% of euploid embryos and 7.46% of aneuploid). However, no difference was detected between the day of biopsy and ploidy. Interestingly, a significant difference (p=0.0071) was detected regarding blastocyst morphology: blastocyst with poor quality were more prevalent in the aneuploid group (16.95%) in comparison to embryos from euploid group (7.98%).

Conclusion: Poor quality blastocysts, classified as C in ICM and/or trophectoderm, have higher chances to be aneuploid. And for this reason, the possibility to biopsy these embryos should be very well evaluated. Our data also suggest that morphology assessment of day 3 embryos do not predict the ploidy status of the resulting blastocyst.

**P-138. Epidemiology of Infertility Among Patients Referred to a Public Secondary Medical Care Center**

**Bruna Costa Queiroz^1^, Luisa Silva de Carvalho Ribeiro^2^, Ines Katerina Damasceno Cavallo^2^, Daniela de Paiva Moreira^2^**

*^1^ Prefeitura de Belo Horizonte - Belo Horizonte - MG - Brasil.*

*^2^ Hospital das Clinicas da UFMG - Belo Horizonte - MG - Brasil.*

**Objective:** This study aims to present epidemiological data from a public secondary medical care center specialized in infertility.

**Methods:** A cross-sectional study was conducted, analyzing data from 1217 patients referred to this public secondary care center for infertility between March 2022 and July 2023. These patients were firstly referred from public primary care centers and, if necessary, would be referred to a public tertiary center for assisted reproduction treatments (ART). Data were manually collected from electronic medical records and results are presented as percentages.

**Results:** The study included 1217 female patients. The majority of patients (61%; n=741) was over 35 years old, with almost half of these women over 40 years old (n=369; 30%), including 171 patients over 43 years old. Additionally, 16% of patients were younger than 30 years old, and 23% fell within the age range of 30-35 years. Primary infertility accounted for 60% of cases. Among couples, 15% (n=180) had been trying to conceive for more than 10 years, while 21% (n=252) experienced infertility for less than 3 years. The cause of infertility could not be determined for approximately 20% of patients (n=207) due to ongoing workup or loss to follow-up. Among the 1010 patients with a stablished cause of infertility, the most common causes identified during the initial work up were tubal factor (21%; n=211), male factor (20%; n=197), ovulatory function abnormalities (17%; n=170), endometriosis (13%; n 132), and unexplained infertility (11%; n=108). Combined factors were present in 3% of couples, and a small proportion of patients (less than 3%; n=31) were in a same-sex relationship or seeking independent reproduction. Uterine factor and advanced maternal age were also identified as causes of infertility, while 5% of couples were referred due to recurrent pregnancy loss.

**Conclusion:** The predominance of patients over 35 years old among those referred to a secondary center aligns with existing literature, which highlights a higher occurrence of infertility between the ages of 35 and 44 in women. The prevalence of tubal factor, ovulatory function abnormalities, and unexplained infertility in this study is consistent with findings in the literature. However, male factor and combined causes were less frequent in this study compared to general reports. World Health Organization emphasizes the importance of identifying, preventing, and treating infertility to promote individual’s overall health. Given that over 70% of the Brazilian population relies on the public health system (SUS) and very few ART centers are available through SUS and the medications used for treatment are not provided by the system at all, studying epidemiological data from these scarce centers specialized in human fertility is crucial for future improvements in public health policies aimed at expanding population’s access to ART.

**P-139. Immediate oocyte denudation or after two hours of cumulus-oocyte complexes in vitro culture: does it really matter?**

**José de Melo Bomfim Neto^1^, Sabrina Maria Rodrigues Jacinto Costa^1^, Thamara Matos dos Santos^1^, Beatriz Tavares Lemos^1^, Andrea Porto Pinheiro Telles^1^, Iuri Telles Donati de Souza^1^**

*^1^ Fertilità - Aracaju - SE - Brasil.*

**Objective:** The intense cell communication that occurs in cumulus-oocyte complexes (COCs) is limited after ovulation trigger and at time of ovum-pick-up (OPU). However, it is still uncertain if the remained communication, such as through paracrine molecules, is important during this period until in vitro fertilization. For the ICSI procedure, oocyte denudation could be performed immediately after OPU or after 2-3 hour of in vitro culture of the COCs, but there is no consensus about the optimal denudation time. The aim of this study was analyze the effect of the time until oocyte denudation on fertilization and cleavage rates.

**Methods:** In this retrospective cohort study, 631 ICSI cycles between 2018 and 2023 were split into two groups; 281 cycles in which denudation was performed immediately after OPU (IM group) and 350 cycles with denudation after 2 hours of COCs in vitro culture (2h group). The excluding criteria were male factor, receptors and cryopreserved sperm cycles. In IM group the ICSI was performed 2 hours after OPU, and in 2h group 3 hours after. The fertilization was checked on day 1 and the cleavage stage of embryos was assessed on day 2 and 3. The age of both groups was compared, but for better understanding of denudation time influence, cycles were also separated by maternal age (< 31 years old, 31-34 years old, 35-37 years old, 38-39 years old, 40-42 years old and > 42 years old). The fertilization (2PN embryos / injected oocytes) and cleavage rates (cleavage embryo / 2PN embryos) between both groups, as well as data from age related subgroups, were compared using Chi square and Fisher’s exact tests with p value ≤ 0.05 considered significant.

**Results:** Although fertilization rate was higher in 2h group (79.53%; 2,051/2,579) when compared to IM group (74.46%; 1,478/1,985), cleavage rate was improved in IM group (IM group; 96.48% (1426/1478) and 2h group; 77.18% (1583/2051)). The subgroup analysis showed that only cycles with maternal age in the ranges 31-34 and 35-37 years old presented an increase in fertilization rate in the 2h group (80.49%; 590/733 and 81.78%; 552/675, respectively) comparing to cycles with immediate denudation (78.48%; 175/223 and 70.77%; 368/520). Interestingly, among all subgroups, the cleavage rate was increased in IM groups (< 31 years old; 98.86% (173/175), 31-34 years old; 95.39% (414/434), 35-37 years old; 98.64% (363/368), 38-39 years old; 96.32% (183/190), 40-42 years old 92.77% (231/249) and > 42 years old; 100% (62/62)) when compared to 2h groups (< 31 years old; 75.98% (174/229), 31-34 years old; 79.83% (471/590) , 35-37 years old; 68.30% (377/552), 38-39 years old; 85.97% (288/335), 40-42 years old; 79.30% (226/285) and > 42 years old; 78.33% (47/60)).

**Conclusion:** Overall, our data suggest that immediate oocyte denudation after OPU, and consequently shorter the time between oocyte recovered and ICSI process, improves cleavage rate, independently of maternal age.

**P-140. Treatment for endometriosis before in vitro fertilization in infertile women who wish to become pregnant: A systematic review**

**Andréa Fortes Carvalho Barreto^1^, Nathalie da Cunha Caldas^1^, Flávia Gabriela Tojal Hora^1^, Ana Flávia Faro Passos^1^, Laís Viana Aragão Almeida^1^, Paloma Lisboa de Souza^1^**

*^1^ Universidade Tiradentes - Unit - Aracaju - SE - Brasil.*

**Objective:** To evaluate treatment for endometriosis prior to IVF in infertile women who wish to become pregnant.

**Methods:** PUBMED search, conducted in May 2023, with the MESH descriptors "Endometriosis" and "Fertilization in Vitro", articulated with the Boolean operator "AND". We found 1451 publications and, after the filters "randomized controlled trials" and "last 5 years", there were a total of 16 articles. With reading of the titles, 8 studies were selected and, after evaluation of the abstracts, there were a total of 7 articles.

Results: Endometriosis is considered the most intractable cause of female infertility. From the analysis of the articles, it was observed that the performance of treatment for endometriosis before in vitro fertilization and embryo transfer (IVF-ET) is an important strategy to improve the results of ET-IVF for infertile women with endometriosis. In this context, it was found that the performance of treatment with dienogest (DNG) immediately before IVF-ET did not provide any benefit to improve the clinical outcomes of infertile women with endometriosis, since the number of growing follicles, recovered oocytes, fertilized oocytes, blastocysts, fertilization rates, cumulative pregnancy rate and live birth rate were lower in the group that used DNG than in the control group. In addition, it was found that three months of treatment with gonadotropin-releasing hormone (GnRH-a) agonists before in vitro fertilization promotes lower concentration of cytokines in the follicular fluid and a higher fertilization rate, but the implantation rate, embryonic quality and clinical pregnancy rate in infertile women with endometriosis had no statistically significant difference from the group that did not receive GnRH-a, so it is a questionable method, and may or may not improve the success rate of pregnancy. It was also evaluated that the effect of vitamin C (VitC) supplementation on the results of *in vitro* fertilization-embryo transfer (IVF-ET) in women with endometriosis did not affect markers of oxidative stress in these patients, only improved serum VitC levels. Finally, we observed the efficacy of different progestogens in women with advanced endometriosis undergoing controlled ovarian hyperstimulation for in vitro fertilization and it was found that the three protocols of medroxyprogesterone acetate plus human menopausal gonadotropin (hMG), didrogesterone plus hMG and progesterone plus hMG are equivalent in terms of fertilization and pregnancy outcomes for women with advanced endometriosis, thus, this protocol may be an alternative choice for women with severe endometriosis and normal ovarian reserve undergoing IVF/ICSI.

**Conclusion:** The correct follow-up of patients undergoing endometriosis treatment who wish to regain fertility and become pregnant is essential, but it is known that the use of dienogest did not generate benefit, that the use of gonadotropin-releasing hormone agonists before in vitro fertilization is questionable, that the use of vitamin C in the results of in vitro fertilization of embryos did not affect markers of oxidative stress and that the three different protocols with progestogens are equivalents and may be an alternative for women with severe endometriosis. But even so, it is concluded that none of these methods was totally effective and safe.

**P-141. The influence of obesity on in vitro fertilization results: A systematized review**

**Ana Flavia Faro Passos^1^, Lais Viana Aragão Almeida^1^, Andréa Fortes Carvalho Barreto^1^, Paloma Lisboa Souza^1^, Nathalie da Cunha Caldas^1^, Flávia Gabriela Tojal Hora^1^**

*^1^ Universidade Tiradentes - Unit - Aracaju - SE - Brasil.*

**Objective:** To systematically analyze in the literature, the influence of obesity on the results of in vitro fertilization.

Methods: For the study, the BVS and PUBMED databases were consulted. In the selection of articles, the keywords “Obesity” and “IVF” were used, articulated with the Boolean operator “AND”. The filter “the last 5 years” was used. In BVS, 96 articles were found and in PUBMED, 250 were found. 4 articles from PUBMED and 3 from BVS were read according to the greatest alignment with the research proposal. The conduct used for data extraction was the analysis of the results of the searches carried out in the databases, this analysis was made from the reading of the articles.

**Results:** Obesity in IVF influences the reduction of live birth rates and increased abortion rates when compared to non-obese women. This occurs because, when comparing the groups, there is a reduction in the quality of oocytes and a lower endometrial receptivity in women with obesity. At first, it was believed that abortions in obese patients could be caused by greater numbers of aneuploid embryos, but through the study it was found that the PGT-A cycles, days of stimulation and number of collected MII oocytes do not show significant changes between obese and non-obese women, with changes only in the amount of gonadotropins, which in obese women require higher dosages. This finding demonstrates that the origin of the increased rate of pregnancy loss after IVF, in these women with euploid embryos, may be related to epigenetic or mitochondrial disturbances of oocytes and embryos, or be secondary to an abnormal uterine environment, fed by an environment inflammation characteristic of obesity.

**Conclusion:** Therefore, it is noticeable that obesity and high body mass indexes (BMI) negatively influence the implantation and development of the embryo in women undergoing in vitro fertilization. In these cases, the articles read demonstrated an increase in the rates of abortion and stillbirths in women with obesity and a reduction in the success of the IVF procedure.

**P-142. The relationship between sperm DNA fragmentation and morphology abnormalities**

**Thamara Matos dos Santos^1^, Sabrina Maria Rodrigues Jacinto Costa^1^, José de Melo Bomfim Neto^1^, Helen Ingrid Santos Cardoso^1^, Emilly Freitas Oliveira^1^, Andrea Porto Pinheiro Telles^1^, Iuri Donati Telles de Souza^1^**

*^1^ Fertilità - Aracaju - SE - Brasil.*

**Objective:** It is well known that high levels of sperm DNA fragmentation could negatively impact IVF cycles, leading ultimately to higher miscarriage rates. On the other hand, some sperm morphology alterations could lead to fertilization failure, often leading to higher abortion rates. However, how the DNA fragmentation and morphology abnormalities are related in IVF outcome is still unknown. For that, the aim of this work was to study the association between high sperm DNA fragmentation and sperm morphology abnormalities.

**Methods:** This was a retrospective cohort study that analyzed 80 sperm samples from patients that underwent IVF cycles for DNA fragmentation by CANfrag test and sperm morphology. As recommended, 28 samples that presented more than 30% of DNA fragmentation were considered as high fragmentation samples (High group) and 62 samples with fragmentation rates lower than 30% composed the Control group). Morphology parameters were analyzed as recommended by WHO. Briefly, sperm head (macrocephaly, microcephaly, vacuoles, misshapen, elongated, fusiform, bicephalous, acephalous, round-head and piriform) acrosome (defective or absent), intermediate piece (drops, eccentric piece and vacuoles) and tail (short, curly, doble-tail and absence of tail) abnormalities were analyzed, as well as pleomorphic and amorphous sperms. Samples with 4% of normal sperm morphology were considered normal. The patients’ age was compared between groups. The fragmentation, total sperm abnormality and each sperm abnormality type were compared between groups using Chi square and Fisher’s exact test with p value ≤ 0.05 considered significant.

**Results:** Samples of the High group showed an increase in abnormal sperm rate (98.36%; 2754/2800) when compared to Control group (97.81%; 6064/6202). Acrosome and tail abnormalities were similar between groups (0.13% and 0.11%, and 0.28% and 0.11% for acrosome and tail abnormalities between Control and High groups, respectively). Total head abnormalities were decreased in high fragmentation samples (22.55% in High group and 25.46% in Control) and, among head abnormalities, microcephaly (8.70%) and round-head (3.54%) sperms were decreased, and acephalous (5.96%) and piriform (1.13%) sperm rates were increased in High group, comparing to Control (microcephaly; 13.67%, round-head; 6.15%, acephalous; 3.82% and piriform; 0.32%). Although the total number of intermediate piece abnormalities did not differ between groups (0.3% in Control and 0.11% in High group), among them, the presence of drops was decreased and eccentric piece rate was increased in samples with higher DNA fragmentation, comparing to control samples (drops presence; 72.22% in Control group versus 0% in High group and eccentric piece rate of 22.22% in Control versus 100% in High group). Regarding pleomorphic spermatozoa, High group showed lower rate (73.83% in Control versus 32.18% in High group). Sperm with head and acrosome abnormalities were less common in High group, with rate of 13.28% versus 23.34% in Control. Moreover, when head and tail abnormalities and 3-piece abnormalities where compared, a higher rate in high fragmentation samples (8.62% and 25.80%, respectively) comparing with control samples was observed, with 5.07% of total abnormalities with head and tail combined and 23.12% with 3-piece abnormalities.

**Conclusion:** Our data indicate that sperm DNA fragmentation and some sperm morphology abnormalities could be related. However, more studies are necessary to understand the impact of this association in IVF cycles outcomes.

**P-143. Treatment of assisted reproduction by the united health system in Brazil**

**Gabriel Monteiro Pinheiro^1^, Gabriela Gouveia^1^, Vitória Luiza Batalhoti Brogiato^1^**

*^1^ UNISA - São Paulo - SP - Brasil.*

**Objective:** This study aims to evaluate the distribution of Assisted Reproduction Treatments (ART) in private and public networks throughout Brazilian territory.

**Methods:** It is a bibliographic review based on indexed scientific articles in Portuguese and English, from the years 2010 to 2023, obtained from databases such as PubMed, Scielo, and the Ministry of Health. The search was conducted using keywords such as "Infertility," "Sistema Único de Saúde or SUS" (Brazil's Public Health System), "Assisted Reproduction Techniques or ART," and "Reproductive Rights." Inclusion criteria comprised studies conducted in Brazil, those that assessed the assistance rates provided by the SUS, and studies related to ART within the SUS. Eight articles were gathered, of which 2 were excluded due to duplicates, title and abstract reading, resulting in 6 selected articles.

**Results:** Article 226 of the Brazilian Federal Constitution guarantees access to scientifically accepted and safe conception and contraception methods, leading to the creation of Ordinance no 426/GM in 2005, which established the National Policy of Integral Care in Assisted Reproduction within the SUS, aiming to provide comprehensive assistance to infertile couples at all levels [1]. A qualitative study from 2014 evaluated the availability of ART in Brazil, indicating that only 5% of consultations are conducted through the SUS, and it highlighted the waiting time for patients, ranging from 6 months to 4 years [2]. Another study emphasized that the coverage of assistance expenses is not comprehensive [3]. Various authors point out that ART is not prioritized within public health [2;3;4;5]. In 2022, the 14th Report of the National Embryo Production System - SisEmbrio was published, indicating 80,575 cycles performed in 2020 and 2021, in 170 Assisted Human Reproduction Centers, showing a total of 181 centers across Brazil [6]. Studies show that only 13 of these available clinics offer treatments through the SUS in Brazil (image 1), and both private and public centers are unevenly distributed throughout the territory [5,6].

**Conclusion:** Given this, it is evident that the availability of these services within the SUS is still limited, and many couples do not have access to such treatments. Strengthening and expanding the policy of integral care in assisted reproduction within the SUS is necessary to reduce geographic and financial inequalities in treatment access. Moreover, it is crucial to invest in research and public policies prioritizing assisted reproduction as a relevant public health issue. Awareness about the importance of reproductive rights and the need for accessible and quality treatments for infertile couples should be promoted both in the context of public health and society at large.

**P-144. Influence of sexually transmitted infections on female fertility**

**Gabriel Monteiro Pinheiro^1^, Gabriela Gouveia^1^, Karolyne Vale de Sá^1^, Vitória Luiza Batalhoti Brogiato^1^**

*^1^ UNISA - São Paulo - SP - Brasil.*

**Objective:** This study aims to assess the influence of late diagnosis and treatment of Sexually Transmitted Infections (STIs) on female fertility.

**Methods:** It is a bibliographic review of articles published in English and Portuguese within the last 5 years, using PubMed and SciELO databases. The search was conducted using the following keywords: "Sexually transmitted diseases (STD)," "sexually transmitted infections (STI)," "Pelvic Inflammatory Disease (PID)," and "female fertility," which resulted in 28 articles. The inclusion criteria were studies involving women aged 18 to 40, with infertility complaints due to a history of STIs, and a correlation between STIs and female infertility. After removing duplicates and reviewing titles and abstracts, 9 articles were utilized for this study.

**Results:** Different authors point out that STIs negatively impact around 30% of female fertility. For example, bacterial infections like Chlamydia (Chlamydia trachomatis) and Gonorrhea (Neisseria gonorrhoeae) have adverse effects on pregnancy, even when asymptomatic, and may lead to Pelvic Inflammatory Disease (PID) affecting structures such as the uterus, fallopian tubes, and ovaries. Infections in the upper genital tract cause inflammation that can result in adhesions and obstruction of the fallopian tubes, conditions associated with female infertility due to tubal factors and ectopic pregnancy, caused by impaired egg transport. Different studies show that tubal factors account for 25-35% of female infertility causes, and in women with recurrent PID (more than 3 episodes), over 50% may experience tubal dysfunction. Chlamydia infections have also been associated with chronic pelvic pain, spontaneous abortion, and other adverse pregnancy events. Systematic reviews indicate that Chlamydia infection is primarily linked to stillbirths (OR = 5.05) and spontaneous abortion (OR = 1.30), with approximately 76% of published studies supporting these associations. Furthermore, the research indicates that positive associations between STIs, spontaneous abortion, infertility, and ectopic pregnancy are more frequent in low-to-middle-income countries compared to high-income countries. The treatment for PID involves broad-spectrum antibiotic therapy, best initiated as early as possible, and including partner treatment to prevent reinfections. Despite a good response to treatment, recurrent infections increase the risk of developing tubal infertility, making the investigation of STI history crucial in the assessment of infertile couples. Treatments for tubal infertility resulting from PID, besides treating the infection, include laparoscopic or hysteroscopic removal of adhesions and in vitro fertilization.

**Conclusion:** Therefore, it is evident that STIs, particularly Chlamydia and Gonorrhea, significantly reduce female fertility. Healthcare professionals must conduct early diagnoses and provide appropriate treatments for pelvic inflammatory diseases to prevent severe consequences on reproductive health. Additionally, providing adequate medication for both the patient and their partner, with special attention to cases of reinfections, is essential. With these measures, the negative effects of STIs on female fertility can be mitigated, enhancing the reproductive health of women of reproductive age.

**P-145. Correlation between embryo’s morphological evaluation, euploidy and implantation rate**

**Mariana Rodrigues Tolentino^1^, Rivia Mara Lamaita^2^, Pedro Henrique Martins Marques^2^**

*^1^ Mater Dei - Belo Horizonte - MG - Brasil.*

*^2^ Universidade Federal de Minas Gerais - Belo Horizonte - MG - Brasil.*

**Objective:** The aim of this study was to determine the relationship between morphological parameters and the incidence of chromosomal abnormalities and to evaluate the potential for implantation of euploid embryos.

Methods: This is a retrospective observational study based on the analysis of medical records from 96 patients who underwent in vitro fertilization (IVF) cycles, totaling 323 biopsied blastocysts. Data were collected from a private Assisted Reproduction clinic in Belo Horizonte. Embryonic morphology assessment was performed according to the Gardner and Schoolcraft system, wherein the inner cell mass (ICM) and trophectoderm (TE) cells were scored based on their number and cohesion into three different grades (A, B, C), with A representing the highest grade. Finally, the incidence of euploid and aneuploid cells in trophectoderm biopsies was compared with embryonic morphological quality and pregnancy rate.

**Results:** The rate of euploidy was significantly higher in embryos with faster growth, with 41.92% of embryos reaching the blastocyst stage on the fifth day being euploid, compared to 22.58% on the sixth day of culture. Regarding morphological grades, the rate of aneuploidy increased significantly in embryos with poorer ICM evaluations, as the euploidy rate was 42.57%, 35.2%, and 23.07% for grades A, B, and C, respectively. As for the TE, the euploidy rates for embryos with grades A and B were comparable, with euploidy rates of 39.28% and 41.29%, respectively. These values were higher than the euploidy rate found in embryos with TE grade C, which showed a rate of only 28.57%. Regarding the potential for implantation of euploid embryos, only 34% of cases resulted in a clinical pregnancy, compared to 63% of cases where no pregnancy occurred. Additionally, a 2% rate of ectopic pregnancy was observed. Despite different morphologies, the pregnancy rates were similar for embryos classified with grade A and B ICM (50%). However, when evaluating the TE, embryos classified as grade A had a clinical pregnancy rate of 30%, compared to a rate of 60% for embryos classified with grade B and 10% for grade C embryos.

**Conclusion:** The selection of embryos with the highest potential for implantation remains one of the most significant challenges in assisted reproduction. Several studies have demonstrated a positive correlation between embryonic morphological appearance and clinical outcomes, suggesting that better morphological aspects may lead to improved chances of successful implantation. However, the complex relationship between embryonic morphology and chromosomal constitution has led to the introduction of preimplantation genetic testing as a complementary tool aimed at selecting euploid embryos and enhancing the delivery rate per transfer. This study confirmed a moderate association between euploidy and blastocyst morphological characteristics. An increased rate of aneuploidy was observed among blastocysts with poorer morphological scores, while embryos with better scores showed a higher likelihood of euploidy. Thus, selection based on morphology appears to be an acceptable parameter for increasing the probability of transferring chromosomally normal embryos. However, it is important to note that this methodology still presents discrepancies in results, with euploid embryos having poor scores and vice versa. For this reason, selection based on embryonic morphology cannot be used as an alternative to genetic testing to minimize the risk of transferring chromosomally abnormal embryos.

Through the results, it was also possible to investigate the role of morphological evaluation in predicting the chance of successful implantation, excluding chromosomal abnormalities from the analysis as a confounding factor. And the morphology appears not to be an important parameter to consider which euploid embryos should be transfer, since better outcomes were observed in embryos with lower quality trophectoderm. Also, euploid embryos with poorer quality ICM produced the same implantation rate as morphologically better evaluated blastocysts.

**P-146. Influence of genetic mapping nowadays**

**Gabriel Monteiro Pinheiro^1^, Gabriela Gouveia^1^, Fernanda Perez Cardim^1^, Giovanna Bonatto Luca^1^**

*^1^ UNISA - São Paulo - SP - Brasil.*

**Objective:** The objective of the present study is to analyse the influence of genetic mapping nowadays.

**Methods:** It is a narrative review of 10 articles published in English and Portuguese on (PubMed, Scielo, LILACS) platforms between 1995 and 2023.

**Results:** The Human Genome Project was formally initiated in 1990 and ended in April 2003, with the aim of sequencing the 3.1 billion nitrogenous bases of the human genome, which characterizes the set of DNA of a living being. DNA is composed of a sequential bond of nucleotides formed by a chemical bond between deoxyribose and a nitrogenous base, which can be an Adenine, Guanine, Thymine or Cytosine. The sequential order of these bases differentiates the molecular content that will be expressed in translation and transcription, which has a majority importance for the overall functioning of an organism. In this context, the discovery of the sequencing of the bases by the project, manifested enormous scientific advance for a deeper understanding of the human genetic code, through genetic mapping. Genetic mapping, or genetic sequencing, consists of the analysis of DNA extracted from a person, to genotypically, discover changes that can be expressed in the phenotype of the individual. Understanding chromosomal and gene structure provides the possibility of diagnosis, treatment, and prevention of genetic diseases. In addition, genetic mapping is extremely relevant for the identification of variations associated with hereditary diseases, which may present late manifestation. This provides a more accurate and sometimes early diagnosis, which presents to the patient and their family the possibility of genetic counseling and taking early precautions in some cases. In addition, genetic mapping has been a remarkable tool for the development of personalized therapies. By doing the analysis of the genotypic profile of the patient, professionals can present to which, the most effective treatments and the risks according to the genome. This, in addition to enabling a more targeted therapeutic approach to the treatment of several diseases, demonstrates a more hopeful path for the future of treatments. On the other hand, another considerable aspect is the fact that once the genetic sequencing of populations is done, it is possible to analyze the genetic profile of distinct people, who share similarities, identifying genetic patterns more prevalent in certain regions and ethnic groups. The collection of this information is essential in helping to understand genetic diversity, susceptibility to diseases, responses to drugs and the evolutionary theory of the human being. Similarly, studies related to how lifestyle and environmental conditions interact with human genetics have benefited from genetic mapping. This area of study related to how different environments generate the activation or deactivation of genes is called functional genomics. With this, it becomes possible to adopt personalized preventive measures for each individual based on their genetic profile, such as lifestyle changes, generating health benefits.

**Conclusion:** In short, it is possible to affirm that genetic mapping plays a fundamental role today, to allow a detailed understanding of the genetic constitution of the human being. Genetic sequencing was able to generate advances in scientific and technological research, and especially in the context of the medical sphere and in the understanding of the complexity of the health-disease process. Due to the potential for personalized therapies, prevention, early discovery of diseases, more accurate diagnoses, it is possible to affirm that genetic mapping is the beginning of the path to the future of treatments and an approach focused on the needs of each patient, in an individualized and effective way in health care.

**P-147. Effects of Clomiphene Citrate on Hormonal and Seminal Parameters in Men Diagnosed with Oligozoospermia**

**Bruno Hallan Meneses Dias^1^, Tiago Magalhães^1^, Mayara Lobato Lourenço^1^, Amanda Pereira Tomás^2^, João Erick Pompeu^2^, Ana Beatriz Guedes^2^, Eduardo de Paula Miranda^1^**

*^1^ Sollirium Health Group - Fortaleza - CE - Brasil.*

*^3^ Unichristus - Fortaleza - CE - Brasil.*

**Objective:** This study aimed to assess the therapeutic response to clomiphene citrate (CC) in men diagnosed with oligozoospermia at a human reproduction center

**Methods:** A retrospective analysis of medical records was conducted, focusing on the hormonal profile (total testosterone - TT, estradiol - E2, follicle-stimulating hormone - FSH, luteinizing hormone - LH) and semen analysis before and after CC treatment. The variations in values were expressed as ∆% = [(final value - initial value) / initial value] x 100

**Results:** Semen analyses analysis revealed that 45.5% of patients exhibited isolated improvements in sperm concentration, while 31.8% demonstrated enhanced progressive motility. Hormonal profile analysis indicated a 90% increase in total testosterone after treatment, with a mean variation (∆TT) of 68.6%. Among patients with elevated testosterone levels, 22.2% showed a ∆TT less than 20%. Estradiol levels increased in 87.5% of patients, with a ∆E2 of 43.3%. A decline in the total testosterone/estradiol ratio was observed in 25% of patients; however, only 16.7% had ratio values below 10 after treatment. FSH levels were elevated in 72.7% of patients, with a ∆FSH of 45.5%. LH levels were increased in 81% of patients, with a ∆LH of 58.7%. Among the 19% of patients who showed a reduction in LH after treatment, no evidence of a decrease in testosterone levels was observed.

**Conclusion:** Clomiphene citrate represents a cost-effective, well-tolerated, and user-friendly option that leads to improvements in seminal parameters in semen analysis for men diagnosed with oligozoospermia.

**P-148. Is there any relation of Assisted Reproduction Techniques and congenital anomalies?**

**Gabriel Monteiro Pinheiro^1^, Gabriela Gouveia^1^, Esther Gomes de Melo^1^, Giovanna Bonatto Luca^1^, Vitória Luiza Batalhoti Brogiato^1^**

*^1^ UNISA - São Paulo - SP - Brasil.*

**Objective:** Therefore, this study aims to investigate the incidence of CA in live births from assisted human reproduction.

**Methods:** This is a bibliographic review made from scientific articles indexed in Portuguese and English, between the years 2006 to 2023 in the databases available for access to PubMed, Scielo and the Health System; The search was done through the key words "congenital anomaly" and "assisted reproduction". Publications from all areas of knowledge were selected, which resulted in 14 articles, 8 articles remained, of which they were used in the course of this work.

**Results:** According to the Health System, CAs are a group of structural or functional changes that occur during intrauterine life and that can be detected before, during or after the birth of the fetus, these changes are mainly of genetic origin, in addition to possible infectious, nutritional or environmental causes [2]. The Information System on Live Births (SINASC) points out that about 25 thousand Brazilian live births are registered annually with some type of CA, for this reason Law No. 13,685, of June 25, 2018 regulates, in Brazil, the CAs as compulsory in SINASC [3]. A study done by the State Medical University of Botucatu with the Child Development Monitoring Center of Ribeirão Preto compared the rates of live births conceived by intracytoplasmic sperm injection (ICSI) with the general population that has some of the CAs and found that about 2.9% of this first group progressed with some major congenital malformation (MCM), This value when compared with the second group shows references close to the expected of 2.6% [4]. In agreement with this, a study conducted in 2012 demonstrated that babies conceived by ART have a significant risk of presenting some congenital anomaly when compared to children who are conceived naturally, with an OR of 1.37; this same study showed that between in vitro fertilization (IVF) and ICSI, there is no significant difference in the rates of CAs [5]. In 2012 the American Association of Pediatrics pointed out that about 9% of IVF cases had CAs, which showed an increase of 2.3% in relation to natural pregnancies where 6.6% of children in this same profile were found [6]. A 2019 study in India compared the prevalence of CAs in ART pregnancies with those of spontaneous conceptions and pointed out that births resulting from IVF or ICSI pregnancies do not tend to have a higher rate of birth defects [7]. In 2021 epidemiology and health services released a list of priority CAs for surveillance, pointing out as such: neural tube defects, microcephaly, congenital heart defects, oral clefts, genital organ anomaly, limb defect, abdominal wall defect and Down syndrome, respectively [2;8].

**Conclusion:** Different studies are still conflicting regarding the differentiation of the rates of congenital malformation in groups submitted to ART. The slightly increased rates in certain studies may come from the surveillance modality adopted in the country, in addition to early detection in pregnancies after IVF or ICSI; in Brazil, compulsory notification and greater monitoring of epidemiological surveillance have been shown to be important to establish public policies for care, education and health care. Given these points, a study with a larger sample size is necessary for a better understanding of the prevalence, incidence and relation of ART with CAs.

**P-149. The incidence of sexually transmitted infections in women of reproductive age in the hospital outpatient Wladimir Arruda School (HEWA)**

**Gabriel Monteiro Pinheiro^1^, Gabriel Napolitani^1^, Fernando Oliveira^1^, Luiz Henrique^1^, Marina Tiemi^1^, Gabriela Gouveia^1^**

*^1^ UNISA - São Paulo - SP - Brasil.*

**Objective:** To estimate the incidence of sexually transmitted infections in women of reproductive age, between 15 and 45 years old, attending the outpatient clinic of Hospital Escola Wladimir Arruda (HEWA).

**Methods:** The research was conducted with 107 women between 15 and 45 years old, who underwent oncotic cytology and real-time Polymerase Chain Reaction (PCR) with specific primers from November 2020 to December 2021 for screening and diagnosis of urogenital infections caused by the bacterium Chlamydia trachomatis, according to sexual frequency, number of partners in the last 6 months, frequency of sexually transmitted infections, and the number of pregnancies.

**Results:** Out of the 107 interviewed women, 17 (18.19%) tested positive for Chlamydia trachomatis. All positive cases were sexually active, with only 1 (0.17%) having sexual relations with 4 partners in the last 6 months of the collection period, 5 (0.85%) had not been pregnant, and all positive cases (18.19%) reported using contraceptive methods, with only 1 (0.17%) having had other sexually transmitted infections. According to current literature, we know that the occurrence of conjugal infertility due to tubo-peritoneal factors is approximately 30% to 40% of the causes of this pathology, and that tubal damage can be induced by a Sexually Transmitted Infection (STI), where the main infectious agent is Chlamydia trachomatis.

**Conclusion:** Based on the results, we conclude that 17 women of reproductive age, or 18.19% of the total studied, were infected with Chlamydia trachomatis by the PCR method. The frequency of infection indicates that even with a fixed partner, the risk of contamination without contraceptive methods is high, and the association with other STIs does not facilitate contamination by this infectious agent. Early diagnosis and treatment with antibiotics reduce the risks of developing infection and inflammation of the upper genital tract, being a subclinical disease, thereby reducing the occurrence of conjugal infertility due to tubal obstruction.

**P-150. The impact of sperm morphology on human reproduction**

**Gabriel Monteiro Pinheiro^1^, Gabriela Gouveia^1^, Giovanna Bonatto Luca^1^, Juliana Neves Martins^1^**

*^1^ UNISA - São Paulo - SP - Brasil.*

**Objective:** The objective of the present study is to analyse the influence of genetic mapping nowadays.

**Methods:** It is a narrative review of 9 articles published in English and Portuguese on (PubMed, Scielo, LILACS) platforms between 1999 and 2023.

**Results:** Sperm morphology is considered extremely important in cases where there is reproductive difficulty as it is directly related to male fertility. It is one of the evaluations carried out in a spermogram test, which measures several semen parameters to assess a man's ability to generate a pregnancy. Morphological analysis has the objective to analyze the shape and structure of sperm where sperm with a morphology normally have a higher probability of successfully fertilizing the egg. On the other hand, morphological abnormalities can indicate problems in sperm function and affect fertilization capacity A high percentage of morphologically normal sperm is considered a positive indicator of male fertility. If most of the sperm show abnormalities, this could indicate a reduced ability to fertilize and increase difficulties in conceiving. Sperm morphology is just one of many factors evaluated in the spermogram, along with sperm count, motility, and other parameters. All of these aspects are important to get a complete picture of male reproductive health. In addition, analysis of sperm morphology can also help identify possible causes of infertility, such as genetic problems, infections, injuries or exposure to toxins. This information can guide doctors in choosing the most appropriate treatment to help a couple conceive. Playing a crucial role in assessing male fertility. The analysis of the shape and structure of the sperm provides important information about its fertilizing capacity. Detection of morphological abnormalities can indicate problems with sperm function and affect the ability to conceive. Furthermore, sperm morphology is just one of many parameters evaluated in the spermogram, along with sperm count, motility and other factors, which together provide a complete picture of male reproductive health.

**Conclusion:** In summary, sperm morphology is important because it provides crucial information about sperm quality and function, helping to assess male fertility and identify possible causes of infertility.

Looking to the future, it is expected that technological advances will drive the improvement of sperm morphology. Automation, computational analysis and three-dimensional approaches are being developed to accelerate and improve sperm morphological analysis. Additionally, research is exploring specific molecular markers and the use of artificial intelligence to improve the accuracy and efficiency of diagnoses. Integrating morphological assessment with functional testing offers a more comprehensive perspective of sperm quality.

**P-151. Functional ovarian reserve in women with sickle cell disease: A systematic review**

**Caroline Santos Silva^1^, Nathália Muraiviechi Passos^2^, Raquel Rodrigues Mattos^3^, Evangelista Torquato^4^, Eduardo de Paula Mirada^3^, Jose Bessa Junior^1^**

*^1^ Universidade Estadual de Feira de Santana - Feira de Santana - BA - Brasil.*

*^2^ Instituto Gonçalo Moniz - FIOCRUZ - Salvador - BA - Brasil.*

*^3^ Unichristus - Fortaleza - CE - Brasil.*

*^4^ Clínica Evangelista Torquato - Fortaleza - CE - Brasil.*

**Objective:** To carry out a systematic review on the evaluation of functional ovarian reserve in women with sickle cell disease.

**Methods:** The protocol for this systematic review was previously registered in PROSPERO, under registration number CRD42023432505. A search was performed in Pubmed, Science direct, SciELO, Lilacs, Clinical Trials, and CAFe databases, in addition to preprint servers and reference lists of selected publications, to locate studies that evaluated the ovarian reserve of women with SCD. Relevant original studies that used methodologies such as: cohort, case-control, cross-sectional study, and clinical studies, regardless of publication status, were eligible for inclusion. There were no restrictions on languages and date of publication. Two independent reviewers carried out the search, and the risk of bias in the selected studies was assessed using the Newcastle-Ottawa scale.

**Results:** 1,086 records were initially retrieved after consulting the selected databases, and one article was identified after consulting the reference lists of the screened articles, only four articles met the eligibility criteria. The quality of evidence was rated very low due to the design of the studies, however, the risk of bias was considered low. These are recent studies published between 2015 and 2021, as for methodology, three cross-sectional studies and one case-control study were carried out, including a total of 229 adult women with SCD (HbSS, HbSC, and HbSβ). Two studies included a control group, totaling 156 women without SCD, matched by age and/or race. All studies reported lower levels of anti-Müllerian hormone (AMH) in women with SCD when compared to reference values for women of the same age without the disease. Although lower, AMH levels were within the normal range in young women with SCD only with supportive care, in those who used hydroxyurea (HU), a more significant decrease in ovarian reserve was observed and, in the participant, undergoing hematopoietic stem cell transplantation (HSCT), AMH levels indicated primary ovarian failure (undetectable AMH).

**Conclusion:** There is insufficient evidence to support a causal relationship between SCD and reduced ovarian reserve. From anti-mullerian hormone values, there appears to be a trend towards lower levels in women with SCD compared to healthy women, as reported in the four studies evaluated. Studies that analyze the ovarian reserve based on imaging and biochemical parameters are an important future focus.

**P-152. Is there any difference between neonatal outcomes from twin pregnancies according to its conception method?**

**Victoria Campos Dornelles^1^, Marta Ribeiro Hentschke^1^, Isadora Badalotti-Teloken^1^, Fabiana Wingert^1^, Julia Dallanora^2^, Kassia Merchioratto^2^, José Luiz Petersen Krahe^2^, Jorge Alberto Bianchi Telles^2^, Alvaro Petracco^1^, Mariangela Badalotti^1^**

*^1^ Fertilitat - Reproductive Medicine Center - Porto Alegre - RS - Brasil.*

*^2^ Hospital Materno Infantil Presidente Vargas - Porto Alegre - RS - Brasil.*

**Objective:** To compare neonatal outcomes from twin pregnancies according to reproductive techniques and spontaneous pregnancies (SP).

**Methods:** A retrospective observational study was performed in a reproductive medicine center and in a maternal child public hospital, both located in South of Brazil. Data regarding reproductive techniques were collected from 1990-2022 and regarding SP from 2021-2022. A total of 875 live births' twin pregnancies resulting from reproductive techniques were included in addition to 80 live birth's twin pregnancies resulting from SP, all divided into 4 groups according to conception: G1, In vitro fertilization embryo transfer (IVF-ET; n= 635) vs. G2, frozen embryo transfer (FET; n= 218) vs. G3, ovulation induction/artificial insemination (OI/AI; n= 22) vs. G4, SP (n=80). Regarding reproductive techniques, all cycles with embryo biopsy, freezing oocytes or received donated oocytes were excluded to avoid bias. Considering an alpha 0.05, the total sample was deemed with 80% of statistical power. Variables regarding neonatal outcomes were compared between groups, expressed as mean±SD, median[IQR] or n(%), as appropriate. Anova and Kruskal-Wallis tests were applied, considering *p*<0.05.

**Results:** Comparing groups 1 vs. 2 vs. 3 vs. 4, the following results were found, respectively: maternal age, yo (33.8±3.8 vs. 34.3±3.7 vs. 32.3±3.4 vs. 26.8±7.2, *p*=0.000); first newborn's weight, g (2262.8±559.5 vs. 2336.6±592.7 vs. 2371.5±603.3 vs. 2112.3±615.9, *p*=0.030); second newborn's weight, g (2197.8±548.1 vs. 2318.4±568.8 vs. 2181.3±478.5 vs. 2002.4±681.6, *p*=0.000); newborn's birth weight difference, g (235 [100-420] vs. 250 [100-410] vs. 217.5 [100-470] vs. 310 [125-470], *p*=0.114); n%: prematurity, % (66.5 vs. 69.3 vs. 54.6 vs. 81.3, p=0.027); first newborn's Apgar 5thmin≥7, % (97.4 vs. 95.4 vs. 95.4 vs. 78.7, *p*=0.042); second newborn's Apgar 5thmin≥7, % (95.9 vs. 95.4 vs. 95.4 vs. 76.2, *p*=0.030); C-section, % (96.8 vs. 99.0 vs. 95.4 vs. 77.5, *p*<0.001).

**Conclusion:** Previous studies have already shown differences in singleton pregnancies' neonatal outcomes regarding reproductive technique, but little is known when considering twin pregnancies or SP comparison. In this study, the singleton pregnancy pattern was not observed in twin pregnancies, since there was no difference in neonatal outcomes according to reproductive technique. However, twin pregnancies from SP were associated with higher prematurity rate, lower birth weight in both newborns, lower Apgar 5thmin index, even considering its lower sample size. This could be related to social economical differences among reproductive treatments' patients and SP patients from public health service, which has little prenatal care assistance. The higher C-section rate in reproductive patients also reflects this pattern, since the public health system does not allow patients to choose for elective C-section delivery, which is more associated with private patients in Brazil. Furthermore, it is important to emphasize that the public hospital included in this study is considered a local high risk pregnancy reference center, receiving most of the complicated twin pregnancies from primary health care. Thus, these results highlight the importance of better understanding the physiopathological mechanisms involved in neonatal outcomes from reproduction treatments.

**P-153. Expanding the Human Seminal Virome Panel: Latest Findings and Implications**

**Beatriz Helena Dantas Rodrigues Albuquerque^1^, Daniel Carlos Ferreira Lanza^1^**

*^1^ Federal University of Rio Grande do Norte - Natal - RN - Brasil.*

**Objective:** In 2022, we published the first panel to analyze the human seminal virome. In this study, we provide a comprehensive update covering subsequent years (starting from May 8, 2021) to assess the available evidence for the presence of viruses in human semen and their potential impacts on male fertility. Additionally, we revised the panel for the detection of clinically significant viruses in seminal samples.

**Methods:** To characterize the human seminal virome, we initially identified all studies published from May 8, 2021, to July 25, 2023, as available in the PubMed database without language restrictions. The search was conducted using the following parameters: ("virology"[MeSH Subheading] OR "virology"[All Fields] OR "viruses"[All Fields] OR "viruses"[MeSH Terms] OR "virus"[All Fields] OR "viruses"[All Fields] OR "virus"[All Fields]) AND ("semen"[MeSH Terms] OR "semen"[All Fields] OR "semen s"[All Fields] OR "semens"[All Fields] OR ("sperm s"[All Fields] OR "spermatozoa"[MeSH Terms] OR "spermatozoa"[All Fields] OR "sperm"[All Fields] OR "sperms"[All Fields]) OR "seminal"[All Fields]). The titles, abstracts, and full texts of the identified articles were carefully examined. Only articles discussing the presence of viruses in semen through isolation, amplification, or detection of nucleic acids or specific antigens were considered in this review. Reviews, meta-analyses, and other publications that did not report original clinical data were excluded. Studies conducted in vitro or in animal models and those with unavailable full texts were also not considered. Once the main viruses were identified, the search was expanded to include previously published articles and animal studies to provide more comprehensive insights for each case.

**Results:** A total of 484 articles were initially identified. After screening by titles, abstracts, and full texts, 55 articles were included. The analysis of selected articles revealed that 23 virus species were identified in human semen during May 2021 and July 2023. The monkeypox virus (MPXV) was the most frequently cited, followed by the human immunodeficiency virus (HIV) and human papillomavirus (HPV). Some of these viruses have been associated with abnormalities in seminal parameters, such as low sperm count, sperm DNA damage, and increased leukocytospermia. Eight viruses were newly identified and were not previously reported among the 27 viruses in the last study. These newly identified viruses include MPXV, Coxsackievirus B (CVB), Toscana virus (TOSV), Kyasanur Forest disease virus (KFDV), Enterovirus 71 C2 (EV-A71), Merkel cell polyomavirus (MCPyV), and other polyomaviruses. Based on this data, it is possible to propose an update to the panel of viruses that affect seminal quality. The updated panel includes DNA viruses: MPXV, HPV, Hepatitis B virus (HBV), Herpes simplex virus type 1 and 2 (HSV-1/2), Human cytomegalovirus (HCMV), Human herpesvirus 6 (HHV-6) and Varicella zoster virus (VZV), and RNA viruses: HIV, Zika virus (ZIKV), Severe acute respiratory syndrome coronavirus 2 (SARS-CoV-2), Hepatitis E virus (HEV), Hepatitis C virus (HCV) and Ebola virus (EBOV). Additionally, adjustments can be made to the panel depending on the epidemiological scenario in each region.

**Conclusion:** The number of studies investigating viruses that occur in human semen has significantly increased. Through the joint analysis of all these studies, we list the new viruses that were not identified in the previous study. Additionally, we propose an updated panel of viruses recognized for their potential impact on male fertility and health, now including the monkeypox virus. This panel can assist in evaluating semen quality and represents a valuable investigative tool in cases of infertility. Moreover, it can potentially be adapted for routine pathogen detection, tailored to specific geographic or epidemiological scenarios.

**P-154. Fertility preservation in oncological patients: methods and applications**

**Beatriz de Araújo Cruz^1^, Danielle Barbosa Morais^1^**

*^1^ Universidade Federal do Rio Grande do Norte - Natal - RN - Brasil.*

**Objective:** Conduct an overview of the existing fertility preservation techniques for characterize the most frequently used in cancer patients and the benefits applied to the clinical condition of each patient, differentiating between men and women. It was aimed tracking the success rates of germ cells and tissues reimplanted in patients after preservation of fertility and finished the oncological treatment, as well as providing subsidies for assisted human reproduction professionals to select the most appropriate technique for each patient.

**Methods:** A integrative review of literature was made by using two databases, PubMed and Web of Science, selecting clinical studies through two search strategies: “Fertility preservation” OR oncofertility AND “ovar*” for female patients, and "Fertility preservation" OR "oncofertility" OR "cancer" AND "sperm*" OR "testicle" for male patients, resulting in 9 and 6 selected results, respectively. The PRISMA method was used to select the articles, including only clinical studies, reporting oncological patients.

**Results:** The results pointed to oocyte cryopreservation as the most cited technique for female patients, with breast cancer being the one with the highest incidence in the studies; and semen cryopreservation as the most cited technique for male patients, with testicular cancer having the highest incidence in the evaluated patients. It is also highlighted the importance of hormonal stimulation previously to the capture of oocytes, despite also drawing attention to possible adverse effects on patients with hormone-dependent cancers.

**Conclusion:** Finally, the importance of associating assisted reproduction techniques with the preservation of fertility and well-being of cancer patients was concluded, aiming to reduce the impacts suffered by gonadotoxic treatment and provide positive future perspectives to patients. It is also important to highlight the difficulty in following up the patients for a long time, which may explain the occurrence of different outcomes in the face of the application of different techniques, when trying to track their success rates in obtaining a pregnancy at the end of the oncological treatment. It is evident, therefore, the importance of associating assisted reproduction techniques with fertility preservation, aiming to reduce the impacts suffered by the cancer patient during a potentially gonadotoxic treatment and to provide well-being and positive future perspectives to these patients, regarding the desire to constitute a family from their own gametes. The progress being made in gonadal tissue cryopreservation techniques represent a good outlook principally about young pre pubertal kids, demonstrating the responsibility to improve de research and consolidate protocols to use widely.

**P-155. 10 years of Endometriosis in Brazil and our relation with infertility: a epidemiologic analysis**

**Nathalie da Cunha Caldas^1^, Andrea Fortes Carvalho Barreto^1^, Flavia Gabriela Tojal Hora^1^, Ana Flávia Faro Passos^1^, Lais Viana Aragão Almeida^1^, Paloma Lisboa de Souza^1^**

*^1^ Universidade Tiradentes - Aracaju - SE - Brasil.*

**Objective:** Investigate the epidemiology of hospitalizations due to endometriosis in Brazil in the last decade and the impact on female infertility.

**Methods:** The numbers of hospitalizations due to endometriosis recorded in the IT department of the Unified Health System, DATASUS, using the TABNET tool, from 2013 to2023 in each region of Brazil were used for prevalence statistics. For being a study with a public database, the approval of the Research Ethics Committee was not required.

**Results:** In the last 10 years, there were 125.262 hospitalizations for endometriosis in Brazil, and de prevalence was 12% in 2013, 11.93% in 2014, 9.98% in 2015, 9.54% in 2016, 8.82% in 2017, 9.83% in 2018, 9.61% in 2019, 5.83% in 2020, 6.49% in 2021, 11.2% in 2022 and 4.5% until may of 2023. The results for each region was that the Southeast Region represents 41,87% with 52.429 cases, Northeast Region 26.95% with 33.567 cases, South Region 18.2% with 22.821 cases, Midwest Region 6.98% with 8.746 cases, and North Region 6.12% with 7.669 cases.

**Conclusion:** It was possible to observe that the region with more cases of hospitalization for endometriosis was the Southeast region, and the year with most admissions was 2012. An important information is that from 2021 to 2022 almost duplicated the number of admissions after a time of less notification. This reality reflects the impact of the gravity of this disease in Brazil nowadays, and because of that fertility complications should receive greater attention, given that between 30-50% of people with endometriosis may experience infertility for many reasons, such as a complex network of humoral and cellular immunity factors that modulate growth and inflammatory behavior of ectopic endometrial implants and affects embryo implantation, or endocrine and ovulatory disorders.

**P-156. Male factor and its impact on IVF/ICSI treatment: a retrospective cohort study of 731 cycles**

**Thais Tieml Higa^1^, Conrado Alvarenga^2^, Bruno Nicolino Cezarino^2^, Eduardo Hideki Miyadahira^2^, Larissa Matsumoto^2^, Mariana Moraes Piccolomini^1^, Oscar Barbosa Duarte-Filho^2^**

*^1^ LabForLife - São Paulo - SP - Brasil.*

*^2^ Clínica VidaBemVinda - São Paulo - SP - Brasil.*

**Objective:** This study aims to evaluate the male factor impact on the intracytoplasmic sperm injection (ICSI) treatment outcomes following preimplantation genetic testing (PGT).

**Methods:** This retrospective, observational, longitudinal cohort study involved 731 ICSI cycles performed at LabForLife Reproductive Medicine from January 2019 to June 2023. The female partner’s age was limited to under 35 years old. The cohort was divided into five groups according to the male partner's sperm parameters, based on the fifth percentile of the World Health Organization (WHO) semen manual 6th edition (2021). According to the male partners, the groups were: 1) Normozoospermic, 2) Severe oligozoospermic (sperm number <5 million/mL), 3) Epididymal sperm (PESA - Percutaneous Epididymal Sperm Aspiration and MESA - Microscopic Epididymal Sperm Aspiration), 4) Testicular sperm (TESE - testicular sperm extraction and microTESE - microdissection testicular sperm extraction) and 5) Surgical sperm retrieval (group 3 and 4 together). The data distributions were previously verified using the Shapiro-Wilk test. Data on fertilization rate, blastulation rate (blastocysts per fertilized oocyte), and euploidy rate were analyzed using Kruskal-Wallis test followed by Dunn's post hoc tests. Results were presented as median (95% CI), *p*<0.05 was considered statistically significant.

**Results:** A total of 8072 metaphase II oocytes were inseminated. The fertilization rate was significantly reduced (H4= 52.79; *P*<0.001) in MESA/PESA 73.2% (68.4, 80.3; *P*=0.003), TESE/microTESE 54.2% (48.6, 70.2; *P*<0.001) and surgical sperm retrieval 67.4% (65.1, 75.8; *P*<0,001) when compared with Normozoospermic 86.7% (82.7, 85.1), Interestingly, there was not significantly difference when compared to Severe oligozoospermic patients 82.8% (76.9, 83.8; *P*=0.207). Blastulation rate was significantly reduced (H4= 42.40; *P*<0.001) in Severe oligozoospermic 50% (46.0, 55.5; *P* < 0.001), TESE/microTESE 29.2% (10.2, 48.9; *P*<0,001) and surgical sperm retrieval 51.7% (39.7, 56.0; *P* < 0.002) when compared with Normozoospermic 66.7% (59.7, 63.7). Euploidy rates were not significantly different (H4= 0.38; *P*= 0.984) in the male factor groups compared to Normozoospermic patients.

**Conclusion:** This study demonstrates that severe male factor, mainly when it is used epididymal and testicular spermatozoa, impairs embryo developmental competence, regarding fertilization and blastulation rate. When severe oligozoospermic patients were analyzed, despite the fertilization rate was not affected, the blastulation rate significantly decreased. Perhaps the sperm-oocyte initial interaction and the DNA repair mechanisms of the oocyte could compensate the male factor, which is reinforced by the fact that only female partners under 35 years old were included in the study. It was also observed that the euploidy rate was not affected by the male factor, independently of its gravity. This finding corroborates previous studies, and it raises the discussion of whether PGT should be advised to patients with the male factor as the sole cause of infertility.

**P-157. Orange June - An Action of All AHR Segment Second Year of the National Congress Illumination**

**Hitomi Miura Nakagawa^1^, Viviã Sousa Oliveira^2^, Flávia Giacon^3^**

*^1^ Genesis - Centro de Assistência em Reprodução Humana Ltda, Brasília-DF - Brasil.*

*^2^ Revista Evolution - Brasília - DF - Brasil.*

*^3^ Mater Prime Clínica de Reprodução Humana - São Paulo - SP - Brasil.*

**Objective:** For the second consecutive year, the National Congress was illuminated with orange to raise the awareness of all civil society and sensitize parliamentarians, drawing attention to the suffering of millions of Brazilians diagnosed with infertility. Among the actions pleaded with the Executive and Legislative Powers is the implementation of policies that expand the population's access to fertility treatments paid for by the public network. The objective of this work was to describe to professionals in the area of Assisted Human Reproduction the illumination of the National Congress, which took place for the second consecutive year, and is considered a milestone for the entire sector and goes along with the campaign headed by the Brazilian Association of Assisted Reproduction (SBRA) started in 2016, "Fertility Movement". The Orange June, an important action, since the beginning, has the support of the main assisted reproduction medical societies and has been gaining more and more strength and adhesion of many clinics and professionals in the area, who throughout the month updated their profiles with the orange ribbon, referring to this Evolution Magazine campaign. According to a new report published by the World Health Organization (WHO), many people are affected by infertility throughout their lives. About 17.5% of the adult population - 1 in 6 worldwide - suffers from infertility, showing the urgent need to increase access to high-quality care, as well as the need to preserve fertility. Being well-informed about this topic is essential for anyone who faces the problem or is close to those who deal with it. Between 50 and 80 million people worldwide are affected by infertility, estimates the WHO, which recognizes June as Infertility Awareness Month across the planet.

**Methods:** The methodology used was a brief internet search using the descriptors: family planning, reproductive treatments, fertility preservation, and World Infertility Awareness Campaign. The present description, reported in this congress, can be used for the indexing of other scientific works that will contribute to future research in this area.

**Results:** The results of this article indicate that the illumination of the National Congress opens space for important dialogues between the public and private spheres. It is an action that adds considerable effort to inform the entire civil society about infertility, the importance of early diagnosis, and the need to expand access to specialized services for those who wish to have children. It draws attention to the processing of important Bills that can benefit millions of Brazilians concerning the effectiveness of family planning. It encourages the sum of efforts between professionals, clinics, and institutions; draws attention to the emotional suffering involved; reaffirms that infertility is treated as a public health issue by the competent bodies; encourages educational actions that reach general gynecologists about family planning and the importance of paying attention to the assessment of fertility risks in routine consultations. Finally, it must be recognized that the sum of collaborative efforts is a promising path for everyone who has an interface with the AHR area for the benefit of infertile patients.

**Conclusion:** All actions have different complexities and demand different work fronts. By illuminating the National Congress, considering the visibility achieved, the theme of infertility and reproductive treatments may be guided in the agenda of discussions of the National Congress, enabling resolute actions that are mainly of interest to Assisted Reproduction patients.

**P-158. Efficiency of different seminal processing methods in reducing sperm DNA fragmentation**

**Thais Serzedello de Paula^1^, Bruna de Aguiar Martins^2^, Fernanda Rodrigues Bernarde^2^, Angela Kim^1^, Gustavo Baucke dos Santos^1^, Rafael Portela^2^, Matheus Teixeira Roque^2^, Gustavo Nardini Cecchino^2^**

*^1^ CRIO Brasil Serviços e Comércio LTDA - São Paulo - SP - Brasil.*

*^2^ Mater Lab Reproductive Medicine - São Paulo - SP - Brasil.*

**Objective:** To demonstrate whether distinct sperm sorting methods improves the capacity of selecting spermatozoa with lower DNA fragmentation.

**Methods:** This observational prospective study comprised 22 seminal samples from patients undergoing in vitro fertilization treatment. The seminal samples were evaluated according to the sixth edition of the World Health Organization (WHO) Manual, and only those with a sperm concentration above 5 million sperm/mL and a volume greater than 2.5 mL were included. DNA integrity assessment was performed using the terminal deoxynucleotidyl transferase dUTP nick end labelling (TUNEL) assay under fluorescence microscopy. The samples were evaluated before and after using three different sorting methods: swim-up, density gradient centrifugation, and the ZyMōtTM Multi microfluidic device. Statistical analysis was carried out using the repeated-measure ANOVA with Bonferroni correction for paired data with normal distribution, and the Friedman test for paired data with non-normal distribution.

**Results:** The mean age was 37.9 years (ranging from 24 to 52 years). Infertility factors such as varicocele, obesity, smoking, and others were not considered in the patient selection process. The ZyMōtTM device (10.20 ± 5.13 vs 4.84 ± 4.23; *p* < 0.05) and the Swim-Up technique (10.20 ± 5.13 vs 4.90 ± 2.40, *p* < 0.05) significantly reduced sperm DNA fragmentation levels when compared to the pre-processing fragmentation rates. Conversely, the density gradient centrifugation technique did not lead to a decrease in DNA fragmentation levels, maintaining values very similar to the pre-processing DNA fragmentation index (10.37 ± 5.60 vs 10.20 ± 5.13, *p* > 0.05). No significant differences were found between the ZyMōtTM device and the Swim-Up technique. Sperm morphology showed no significant differences between the three techniques.

**Conclusion:** Currently, the microfluidic device for sperm sorting is gaining increasing attention as it pledges to recover sperm with higher quality and lower DNA fragmentation compared to other conventional techniques. In this study, both the Swim-Up and the ZyMot Multi device improved sperm selection regarding to the rates of DNA fragmentation. However, no differences were found in the rate of improvement between the two methods. Nevertheless, caution should be used when interpreting this data once this is a preliminary proof-of-concept study with a small sample size. Further research is needed to evaluate larger sample sizes and the effect of different seminal processing techniques in both the IVF laboratory and reproductive outcomes.

**P-159. Epidemiological study about costs related to infertility in Brazil from 2018 to 2022**

**Carlos Mathias de Menezes Neto^1^, João Otávio Marques de Souza^1^, Eduarda dos Santos Lima^1^, Claudio Guerra de Lima^1^, Gabriel Alves de Souza Magalhães^1^, Maria Fernanda Santana Barroso^1^**

*^1^ Universidade Tiradentes - Aracaju - SE - Brasil.*

**Objective:** To epidemiologically analyze the costs related to infertility in Brazil from 2018 to 2022.

**Methods:** This is a cross-sectional, descriptive study with a quantitative approach, based on data collected from the Hospital Information System of the Unified Health System (SIH/SUS) linked to DATASUS, considering variables such as "total value," regions, age groups, and ethnicity. The data analyzed were limited to expenses related to infertility within the period between January 2018 and December 2022 in Brazil. Decimal places were excluded from the percentage calculations.

**Results:** It was found that a total of 688,034 Brazilian reais were invested in infertility within the public healthcare services of the country. Furthermore, the cost in each region was analyzed, yielding the following results: 310,854 (45.18%) reais in the Northeast region, 178,291 (25.91%) in the Southeast region, 131,068 (19.04%) in the South region, 40,654 (5.9%) in the North region, and 27,165 (3.94%) in the Central-West country's region. In addition, regarding ethnicity, excluding the 82,162 (11.94%) cases with missing ethnicity information, it was observed that Pardo individuals had the highest number, with 340,851 (49.53%), followed by White individuals with 196,941 (28.62%), Black individuals with 38,892 (5.65%), Yellow individuals with 28,494 (4.14%), and Indigenous individuals with 692 (0.10%). Lastly, concerning age groups, the child-adolescent group (0 to 19 years) accounted for 14,027 (0.7%), the adult group (20-59 years) for 668,879 (97.21%), and the elderly group (60 or more years) for 5,122 (0.7%) of the expenses.

**Conclusion:** Therefore, a higher amount of expenses was observed in the Southeast and Northeast regions, potentially correlated to their higher demographic densities. Regarding ethnicity, a higher prevalence was noticed among Pardo and White individuals, which together accounted for more than three-quarters of the total expenses. In conclusion, concerning age groups, it was evident that women in the adult phase represented practically the entire expenditure.

**P-160. Live births following sperm selection based on the hypo-osmotic swelling test and preimplantation genetic testing from chromosomal translocation carriers: a report of two cases**

**Thais Tiemi Higa^1^, Brenda Campos Villa^1^, Mariana Moraes Piccolomini^1^, Larissa Matsumoto^2^, Oscar Barbosa Duarte-Filho^2^, Lucas Yugo Shiguehara Yamakami^2^**

*^1^ LabForLife - São Paulo - SP - Brasil.*

*^2^ Clínica VidaBemVinda - São Paulo - SP - Brasil.*

**Objective:** The aim of this case report is to demonstrate that the hypo-osmotic swelling test (HOST) could potentially be used to select genetically balanced spermatozoa in male chromosomal rearrangement carriers, and along with the preimplantation genetic testing (PGT) improve the live birth rates for patients undergoing in vitro fertilization (IVF) treatment.

**Methods:** Even though HOST is an obsolete test to assess sperm vitality, according to recent studies, a specific HOST class, B+ type spermatozoa, showed a drastically lower proportion of chromosomally unbalanced cells compared to unselected spermatozoa. For each case here presented, we performed an IVF cycle with standard ovarian stimulation and oocyte retrieval. Semen samples of the translocation carrier patients were subjected to density gradient centrifugation followed by incubation in a hypo-osmotic solution. After HOST-morphology sperm sorting, intracytoplasmic sperm injection (ICSI) was performed. The details of each case are following presented.

Case 1: a 39-year-old man carrying a double translocation, both t(4;16)(q28;q21) reciprocal translocation and der(13;14)(q10;q10) Robertsonian translocation, and his partner, a 36-year-old woman. It was retrieved 19 cumulus-oocyte complexes, of which 14 metaphase II oocytes were fertilized by ICSI; embryo biopsy was performed on three blastocysts on day 5 and on two blastocysts on day 6; only one day 5 blastocyst (B5AA) was found to be euploid and chromosomally balanced according to PGT.

Case 2: a 37-year-old man carrying a reciprocal translocation t(11:18)(q21;q22) and his partner, a 32-year-old woman. In their case, 14 cumulus-oocyte complexes were retrieved, of which 9 metaphase II oocytes were fertilized by ICSI; five blastocysts were biopsied on day 5; according to PGT, only one blastocyst (B4AB) was found to be euploid and chromosomally balanced.

**Results:** In both cases, the euploid and chromosomally balanced blastocysts were transferred and resulted in successful pregnancies and births. The live births remain healthy and in normal development to date. Below are a few highlights of each case outcome.

Case 1: the only complication during pregnancy was subchorionic hematoma at 6 weeks, but with no high repercussions. In November 2022, a healthy female baby was delivered at the gestational age of 39 weeks.

Case 2: the repercussions during pregnancy were gestational diabetes and postpartum hemorrhage. In July 2022, a healthy male baby was delivered at the gestational age of 38 weeks.

**Conclusion:** This report demonstrates that HOST is a potential sperm selection method for patients with chromosomal rearrangements. Most remarkably, as far as we know, this is the first report of live births after using the combined technique of HOST-selection and PGT for chromosomal rearrangement carriers, especially in the rare case of the double chromosomal translocation carrier.

**P-161. Epidemiological analysis of the occurrence of spontaneous abortion in the five regions of Brazil between 2011 and 2021**

**Eduarda dos Santos Lima^1^, Carlos Mathias de Menezes Neto^1^, João Otávio Marques de Souza^1^, Maria Fernanda Santana Barroso^1^, Gabriel Alves de Souza Magalhães^1^, Estélio Henrique Martin Dantas^1^**

*^1^ Universidade Tiradentes - Aracaju - SE - Brasil.*

**Objective:** To comparatively analyze the occurrence of spontaneous abortion in the five regions of Brazil between 2011 and 2021.

**Methods:** This is a cross-sectional study, obtained through data collected from the records of the SUS Hospital Information System (SIH/SUS) and the Mortality Information System (SIM), in the Department of Informatics of the Unified Health System (DATASUS), from 2011 to 2021. Comparative data were analyzed in the five regions of the country in number of hospitalizations, ethnicity, age group and deaths to show the epidemiological profile of the occurrence of spontaneous abortion in Brazil between 2011 and 2021.

**Results:** In the space analyzed, 763,884 cases of hospitalizations due to spontaneous abortion were reported, according to SIH/SUS/DATASUS. The Southeast region had the highest prevalence with 279,106 (36.54%) of the cases, followed by the Northeast region with 273,395 (35.79%). Regarding ethnicity, brown predominated with 488,123 (63.62%), the majority in the Northeast region with 235,865 (48.32%), following a similar attitude in the mentioned data. With regard to age group, it was noted the prevalence of occurrence in the age group of 20 to 29 years, with 341,377 (44.49%) of the cases being registered, and these occurred, in short, in the Southeast and Northeast regions, with the same number of cases 122,448 (35.87%) each. Finally, of the registered cases, 154 (0.02%) died. Thus, such data corroborate the literature, which addresses that the most fertile decade for women is between 20 and 30 years.

**Conclusion:** It was observed that the Southeast region concentrates the highest occurrence of spontaneous abortion, however, when analyzing the ethnicity, the Northeast region stands out with the brown ethnicity, in relation to the age group, it behaves similar to the studies scientific studies on female fertility, as it showed that the occurrence of cases was accentuated in the 20 to 29 age group. Thus, the strengthening of preventive policies, based on the needs of each region, is crucial for the construction of public health policies, together with Primary Care Health teams, with the aim of reducing the risk factors that corroborate with the morbidity and mortality from spontaneous abortion.

**P-162. Note generated by the artificial intelligence program as a selection criterion for elective euploid embryo transfer**

**Andrea Mesquita Lima^1^, Sebastiao Evangelista Torquato^2^, Eduardo Gomes Sá^1^, Tulius Augustus Ferreira Freitas^1^, Gleicyane Sousa Santos Alam^1^, Ellayne Cavalcanti Queiroz^1^**

*^1^ BIOS - Fortaleza - CE - Brasil.*

*^2^ Sollirium - Fortaleza - CE - Brasil.*

**Objective:** The objective of this study was to evaluate whether there was a correlation between the score given by the embryoscope plus artificial intelligence program, Kidscore, and the pregnancy rate, when only euploid embryos were transferred, according to a genetic analysis report.

**Methods:** 133 cycles of elective transfers were analyzed, from February to November 2022, in which only embryos submitted to genetic analysis were transferred. The patients were divided into three groups according to the score that the embryo received from the embryoscope plus artificial intelligence software.

Group 1: Kidscore less than 5 (N = 30)

group 2: Kidscore between 5.1 and 7.0 (N = 40)

Group 3: Kidscore between 7.1 and 9.9 (N= 63)

**Results:** Patients in group 1, those with scores below 5, had pregnancy rates of 37%. Patients in group 2, those with scores between 5.1 and 7.0, had pregnancy rates of 45%. And the patients in group 3, those with the highest scores, had a pregnancy rate of 55%.

**Conclusion:** Among the cases analyzed, it was noticed that the score generated by the artificial intelligence program, together with the results of the genetic analysis, seem to be a good parameter to predict the pregnancy rate. An advantage of using Kidscore in these situations may be when there is more than 1 euploid embryo, and the score could be used as a tiebreaker for choosing which embryo to transfer.

**P-163. Functional ovarian reserve of women with Sickle Cell Disease**

**Caroline Santos Silva^1^, Nathália Muraiviechi Passos^2^, Raquel Rodrigues Mattos^3^, Evangelista Torquato^4^, Eduardo de Paula Mirada^3^, Jose Bessa Junior^1^**

*^1^ Universidade Estadual de Feira de Santana - Feira de Santana - BA - Brasil.*

*^2^ Instituto Gonçalo Moniz- FIOCRUZ - Feira de Santana - BA - Brasil.*

*^3^ FMUSP - Ribeirão Preto - SP - Brasil.*

*^4^ FMUSP - São Paulo - SP - Brasil.*

*^5^ Clinica Evangelista Torquato - Fortaleza - CE - Brasil.*

*^6^ Unichristus - Fortaleza - CE - Brasil.*

**Objective:** To evaluate the functional ovarian reserve in women with SCD using biochemical and ultrasound markers.

**Methods:** A cross-sectional study was carried out in the Municipal Care Program for People with Sickle Cell Disease. Fifty-nine participants who met the eligibility criteria were recruited for the study. Data collection was performed in three stages: clinical evaluation, laboratory evaluation, and ultrasound evaluation. Each participant's hemoglobin genotype was confirmed by hemoglobin electrophoresis. The collection of biological samples was carried out in an accredited laboratory accredited to conduct the study. The team that conducted the trials needed to be made aware of the characteristics of the participants and followed good laboratory and clinical practices. The biological material was discarded after analysis. The collection took place during the follicular phase (between the first and fourth day of the menstrual cycle) to determine the levels of LH, FSH, estradiol, sex hormone binding globulin (SHBG), anti-Mullerian hormone, ferritin, blood count, Complete and hemoglobin electrophoresis. Although AMH could have been performed on other days of the cycle, it is well established that FSH and estradiol for assessing ovarian reserve should be performed in the early follicular phase and CFA. For anti-Mullerian hormone analysis, serum and plasma samples were tested using the Access2 Immunoassay System test (Beckman Coulter), and the antral follicle count was performed using two-dimensional ultrasound.

**Results:** Fifty-nine women with a mean age of 34 ±10 years were evaluated. Most were diagnosed with the HbSS genotype and β-thalassemia (62.7%). Median AMH serum levels were 1.0 ng/mL (0.1 - 1.5 ng/mL). Lower levels of AMH were demonstrated among participants with HbSS hemoglobinopathy and β-thalassemia compared to participants with HbSC and HbCC hemoglobinopathy (0.5 ng/mL (0.1 - 2.2 ng/mL) vs 1.3 ng/mL). mL (0.0 - 3.2 ng/mL)). AMH values were lower in women who used oral contraceptives compared to those who did not use 0.58 ng/mL (0.14 - 2.37 ng/mL) x 1.08 ng/mL (0.28 - 2.57 ng/mL) (*p*=0.632), and in women who used Hydroxyurea, 0.45 ng/mL (0.01 - 1.9 ng/mL) x 2.47 (0.5 - 3.56 ng/ml), respectively. The median number of total antral follicles was 12 (7.5 - 15.3), and the median volume of ovaries was: direct ovary 7.95 cm^3^ (5.63 - 11.1cm^3^) and left ovary 6.1 cm^3^ (4.18 - 7.68 cm^3^). Only one participant had ultrasonographic parameters suggesting a meager functional ovarian reserve (total CFA = 3).

**Conclusion:** The study demonstrated decreased functional ovarian reserve in women with SCD; at least half of the women have AMH values lower than 1.0 ng/mL. These levels are lower than those observed in healthy women in the age group studied.

**P-164. Epidemiologic characteristics of patients undergoing oncofertility preservation at a leading public hospital**

**Ludmila Bercaire^1^, Bruna da Mata^1^, Carolina Santos1, Fernanda VillaLobos^1^, Valeria Santana^1^, Lais Yazbek^1^, Nilka Donadio^1^, Gilberto Freitas^1^, Artur Dzik^1^**

*^1^ Hospital Pérola Byington - São Paulo - SP - Brasil.*

**Objective:** Assess the number of women who underwent fertility preservation (FP) at the Pérola Byington Hospital (PBH), their ages and the underlying diseases in this group, in addition to the percentage of women who returned to the hospital for embryo transfer and the possible reasons of dropout.

**Methods:** Retrospective study based on review of medical records of patients treated at the PBH Assisted Reproduction Centre for FP prior to undergoing treatment for the underlying pathology from January 2010 to December 2018. The method of FP used was oocyte cryopreservation. Ovarian stimulation protocol was individualized according to age, ovarian reserve, comorbidities, and the menstrual cycle period at treatment beginning. Among the inclusion criteria were age ≤ 40 years, the presence of menarche, and absence of ovarian failure criteria. 228 patients underwent fertility preservation in this period, but 66 were excluded from the study due to lack of data in the medical records. In total, 162 patients were included in the study.

**Results:** Of the 162 patients evaluated, 4 underwent fertility preservation treatment in 2010, 6 in 2011, 9 in 2012, 21 in 2013, 15 in 2014, 26 in 2015, 28 in 2016, 30 in 2017 and 23 in 2018. Of this total, 21 returned to use gametes or frozen embryos. The indications for FP were: 124 patients with breast cancer, 10 with ovarian cancer, 14 with other cancers (colon, endometrium, lung, thyroid, lymphoma and sarcoma), 14 with other indications (endometriosis, ovarian mass under investigation, autoimmune diseases).

**Conclusion:** Discussion on FP is essential, especially in some groups of patients, such as oncology patients, who will undergo potentially gonadotoxic treatments or even the surgical removal of the gonads, but also in benign pathologies such as endometriosis, benign ovarian tumours, and autoimmune diseases, which treatments often lead to diminished ovarian function. In cancer patients, the start of stimulation can occur on any day of woman's menstrual cycle, allowing for FP without a delay in the initiation of cancer treatment.

A limitation in the study was the difficulty in obtaining the medical records of some patients due to the lack of data. Despite this, the results demonstrate an increase in demand for fertility preservation over the past decade and that 13% of patients who underwent FP returned for embryo transfer. Mean age of patients undergoing treatment was 31 years. Regarding FP indications, 76.6% were due to breast cancer, 6.2% ovarian cancer, 8.6% other cancers and 8,6% other causes. It is important to emphasize that the study was carried out in a leading hospital for gynecological oncology, thus, a large part of the study sample is composed by oncologic patients. Due to patient characteristics, possible reasons of dropout are oncological progression, long period of observation to rule out recurrence and other personal factors. Our results are encouraging and even higher than the reported in literature. According to Specchia et al. and Cobo et al., 4.5% and 7.4%. of FP returned to use their gametes, respectively. Porcu et al reported a patient return rate of 28%, so most women did not attempt pregnancy, and their oocytes are still crypreserved. Nevertheless, offering FP is no longer considered optional and must be included in every therapeutic program for women who receive an oncological or other diagnosis that can impair ovarian reserve in reproductive age.

**P-165. Miscarriage in assisted reproduction - Systematic review**

**Laís Viana Aragão Almeida^1^, Ana Flavia Faro Passsos^1^, Andrea Fortes Carvalho Barreto^1^, Nathalie da Cunha Caldas^1^, Flavia Gabriela Tojal Hora^1^**

*^1^ Universidade Tiradentes -UNIT - Aracaju - SE - Brasil.*

**Objective:** To systematically evaluate in the literature the incidence and causes of spontaneous abortion in assisted reproduction.

**Methods:** This is an integrative review of articles published in the PubMed, VHL and SciELO databases in the last ten years, using the following descriptors: "Miscarriage" and "Assisted Reproduction". The inclusion criteria were articles written in Portuguese and English, with full text availability in electronic support. Paid studies, carried out on animals, duplicates and those that are not related to the theme were excluded.

**Results:** Three review articles were selected for analysis, according to the inclusion and exclusion criteria. In the first study, 2709 cycles of IVF treatment were included, in which four hundred and eleven ended in early pregnancy loss, and 2,298 had live births. The rate of miscarriages was 14.1% and the influence factors were female age, female BMI, type of cycle, thickness of the endometrium and tubal factor. Female patients aged 40 years or older had increased chances of early pregnancy loss compared to those under 35. Female patients with a BMI of 25 or more were more likely to have miscarriages than those in the normal BMI range. The relationship between obesity and the increased rate of gestational loss after IVF was related to metabolomic, epigenetic or mitochondrial disorders of oocytes and embryos, or to the abnormal endocrine, metabolic and inflammatory uterine environment induced by obesity, related to the lower rates of embryo implantation and the increased risk of miscarriage observed after embryo transfers. The chances of pregnancy loss after the transfer of frozen embryos were higher than after the transfer of fresh embryos. A thin endometrium on the day of embryo transfer increased the chances of embryonic death. The transfer of more than two embryos had lower chances of miscarriage compared to the transfer of a single embryo. In the second article, the focus was on the association between a gradual increase in abortion at in vitro fertilization and female age, in addition to oocyte quality and gestational loss. Regarding age, rates increase by more than 45% among women over 45 years of age, when compared to those under 34 years of age. Regarding the decline in oocyte quality, which reflects a high rate of miscarriage, oocyte aneuploidy was correlated as the most frequent cause. In addition, the risk of miscarriage was higher in women undergoing IVF with a female cause for infertility compared to male factor or unexplained infertility, among which having a higher incidence of secondary infertility, as it is possible that women with secondary subfertility had a previous miscarriage. Then, in the third study, the relationship between a previous induced abortion, whether medicated or surgical, and the results of in vitro fertilization was evaluated. A total of 1,532 cycles of IVF treatment were included; 454 patients had a history of induced abortion and 1,078 did not. The rate of miscarriage was significantly higher (17.6%) vs (9.8%) and the endometrium was significantly thinner among patients with a history of induced abortion compared to those without.

**Conclusion:** Thus, it is understood that abortion rates in assisted reproduction are significant and recurrent in specific cases, which should have a more attentive and careful look so that the procedures have positive results.

**P-166. Influential factors in conception among patients with endometriosis undergoing Assisted Reproductive Techniques**

**Antônio Carvalho Azevedo^1^, Leonardo de Oliveira^1^, Ritta de Kássia Oliveira de Santana^1^, Hélison de Jesus Oliveira^1^, Danilo Santana Santos^1^, Aline de Jesus Lima^1^, Cley Gabriel Lima Carvalho Dantas^1^, Fabricia Correia de Azevedo1, Gabriel Silva Morato^1^, Hellen Néo da Rocha^1^, José Edeilson Dias Vitorino^1^, Yasmin Casado Fortunato^1^**

*^1^ Fundação Universidade Federal de Sergipe - São Cristóvão - SE - Brasil.*

**Objective:** This literature review aims to analyze the factors influencing conception success in patients with endometriosis undergoing Assisted Reproductive Techniques (ART).

**Methods:** The descriptors were determined using the following Boolean operators: "(Infertility) AND (Endometriosis) AND (Assisted Reproductive Technology)." The research was conducted on the databases U.S. National Library of Medicine (PUBMED), Virtual Health Library (BVS), and ScienceDirect. The primary search was conducted by applying filters for free articles and articles published between the years 2018 and 2023, resulting in a total of 335 articles. After excluding duplicates based on title and abstract, 68 articles remained. Following a comprehensive analysis of these studies, those with the least methodological limitations were selected, resulting in a total of 11 articles.

Results: The findings regarding the use of Ovarian Stimulation Protocols (OSP) in patients with endometriosis undergoing In Vitro Fertilization (IVF) / Intracytoplasmic Sperm Injection (ICSI) / Intrauterine Insemination (IUI) are favorable to the use of hormonal techniques. The main agents highlighted for this purpose are Gonadotropin-Releasing Hormone Agonists (GnRH-a) and Gonadotropin-Releasing Hormone Antagonists (GnRH-ant). Both have shown benefits when administered before IVF/ICSI/IUI procedures. In a case-control study, it was observed that patients treated with GnRH-a had higher Clinical Pregnancy Rate (CPR) and Live Birth Rate (LBR) compared to those without the use of GnRH-a. Concerning the use of GnRH-ant, a systematic review presented similar results regarding CPR. However, limitations were identified with the use of GnRH-ant in cases of advanced endometriosis and combined forms of endometriosis, as GnRH-a showed greater advantages, particularly when anti-Mullerian hormone levels ranged from 1.1 to 2.7 ng/ml and when fresh embryo transfer was performed, respectively. Analyzing the dosage of GnRH and its effects, it was noted that the values of GnRH-a used are higher than those used in GnRH-ant treatment, and in this context, the use of GnRH-a predicts a lower CPR. It is worth noting, though, that the majority of current studies indicate superiority with the use of GnRH-a, as there are more retrieved oocytes available for subsequent cryopreservation. Other drugs have also been employed to improve the outcomes of ART in patients with endometriosis, such as Atosiban, which showed a significant increase in pregnancy rates in these patients. The Ovarian Stimulation Protocol with Progestin (OSPP) was evaluated for its potential benefits when used prior to In Vitro Fertilization (IVF) for the suppression of endometriosis. A cohort study revealed that the OSPP yielded comparable results to GnRH-a and GnRH-ant protocols, with similar Implantation Rate, Clinical Pregnancy Rate (CPR), and Ongoing Pregnancy Rate as the ultra-long GnRH-a protocol (using 3.75 mg of triptorelin acetate) and the GnRH-ant protocol (0.25 mg/day). However, it is worth noting that the OSPP might be associated with inferior reproductive outcomes concerning biochemical pregnancy, clinical pregnancy, and Live Birth Rate (LBR) compared to the ultra-long GnRH-a protocol.

**Conclusion:** The use of GnRH-a, GnRH-ant, and PEOP has shown good results in patients with endometriosis undergoing Assisted Reproductive Technology (ART), as it promoted an increase in Total Gonadotropin Consumption (TGC) and Total Number of Viable embryos (TNV). Specifically, the analyzed literature suggests that GnRH-a exhibits superiority when compared to GnRH-ant, while PEOP has shown inferiority when compared to the other two hormonal techniques mentioned.

**P-167. Reproductive tourism: Brazil as a destination for this medical practice through a literature review**

**Alexandre Antônio de Lima Junior^1^, Fálba Bernadete Ramos dos Anjos^1^, Manuella Amlid Pimenta de Castro Cavalcanti Silva^1^, Adriana Fracasso^1^, Karollyne Skarlet Gomes da Silva^1^, Evandro Valentim da Silva^1^**

*^1^ Universidade Federal de Pernambuco - Recife - PE - Brasil.*

**Objective:** The objective of this work is to address reproductive tourism and Brazil as a destination for this medical practice through a literature review.

**Methods:** The research was conducted using the electronic databases PubMed and SciElo, focusing on publications between the years 2010 and 2022, as well as official resolutions from the Federal Council of Medicine and reports obtained from the Latin American Network of Assisted Reproduction. Articles, reports, and bulletins that covered aspects related to reproductive tourism were selected to gather data demonstrating that Brazil is one of the most sought-after destinations globally for this medical practice and its associated factors.

**Results:** Reproductive tourism, also known as "fertility tourism," has gained international prominence due to the increasing demand for this medical practice. Brazil is one of the most sought-after countries in Latin America, boasting over 180 clinics specializing in reproductive medicine (ANVISA, 2019). Reproductive tourism refers to patients seeking assisted reproductive treatments and procedures in other countries due to prohibitions and restrictions in their home countries but permitted elsewhere (Lauxen, 2013). Motivations for reproductive tourism vary, but the most significant ones are related to prevailing laws in their home countries or states, specialized clinics with advanced assisted reproductive techniques, and the cost involved in assisted reproduction processes. Patients seek countries where their currencies hold more value and the cost of living is lower. Assisted reproduction can cost almost double or triple in countries where these techniques are allowed (Paraskou, 2017; Almeida, 2015; Corrêa, 2015). Due to the low number of gamete donors and the prohibition of commercialization of gametes, both male and female, as well as compensated surrogacy, which are illegal in Brazil (CFM nº 2.294/2021), there is an exportation of patients to countries like Spain, Belgium, and the United States. In turn, Brazil receives patients seeking reproductive medicine due to lower costs compared to other countries or broader legislation, such as that of some African countries. Mozambique and Angola are among those countries seeking fertility treatment and access to assisted reproductive techniques in Brazil, as reported by G1 in 2012. The Latin American Network of Assisted Reproduction (REDLARA) showed that in 2019, Brazil ranked first in Latin America for the number of performed in vitro fertilization (IVF), artificial insemination (AI), and embryo transfers. Around 83,000 Brazilian babies were born through assisted reproduction treatments in 25 years (REDELARA, 2021). Reproductive tourism is, above all, a response to governments and institutions that, from a financial standpoint, do not provide accessibility to healthcare services or promote aspects of reproductive medicine. From a sociocultural perspective, it hinders and prohibits access or inclusion to assisted reproduction treatments and procedures for all. From a legal perspective, it forces its citizens to circumvent laws, access restrictions, or waiting lists in other countries or states to have the opportunity to start a family (Bergmann, 2011).

**Conclusion:** Based on the data obtained from the literature review, it is evident that Brazil is a favorable country for the practice of reproductive tourism. The legislation, costs, and sociocultural aspects of the country enable individuals and social groups to access assisted reproduction techniques and realize their dream of forming a family.

**P-168. Medical and psychosocial aspects related to in vitro fertilization in same-sex couples: A systematized review**

**Paloma Lisboa de Souza^1^, Laís Viana Aragão Almeida^1^, Ana Flavia Faro Passos^1^, Andréa Fortes Carvalho Barreto^1^, Nathalie da Cunha Caldas^1^, Flávia Gabriela Tojal Hora^1^**

*^1^ Universidade Tiradentes - Aracaju/SE - SE - Brasil.*

**Objective:** To understand the ethical norms for the use of assisted reproduction techniques for same-sex couples, and the psychosocial impact related to the difficulties encountered in relation to the treatment.

**Methods:** The articles were retrieved from the PUBMED and GOOGLE SCHOLAR database, conducted in July 2023, with the descriptors "Same-sex couples" and "IVF". In PUBMED, 26 results were found and in GOOGLE SCHOLAR 5740, with filters for articles published in the last ten years, emphasizing those that address the medical and psychosocial aspects of assisted reproduction in same-sex couples. With the reading of the titles, 10 studies were selected, and after analyzing them, there were a total of 7 articles

**Results:** Seeking to understand the access of homosexuals to assisted reproduction techniques, scientific studies are carried out, considering the difficulties faced in relation not only to treatment, but also to prejudice. In this context, compared to other countries, Brazil is not yet legislatively regulated, leading same-sex couples to resort to the judiciary so that they have the right of paternity admitted. Therefore, it is asked, has the country really come to accept that homosexual couples have children through assisted reproduction? Couples with formulation outside the conventional saying go through longer processes than couples within the standard established by society, going beyond legal difficulties, facing the prejudice rooted in society. As a result, recent studies have proven relationship satisfaction among gay couples after IVF and a more positive functioning of the relationship, with less stress and anxiety associated with parenting when compared to heterosexual parents, because the goal of assisted reproduction occurs with the same purpose, however, as the journey requires much more medical and psychological effort for same-sex couples, they become increasingly welcoming, privileging and always putting the health and comfort of their child first.

**Conclusion:** It is inferred the importance of ensuring a less arduous approach and process for same-sex couples, since they go through many difficulties in relation to medical and psychological treatment. Thus, today, fertility centers increasingly recognize the unique issues of same-sex couples, offering greater hospitality and medical and psychosocial treatment, seeking to help them in each adversity throughout the process.

**P-169. Medicine students' knowledge about late reproductive planning and its implications**

**Giovanna de Sá Veloso Oiticica Magalhães^1^, Ana Cláudia Moura Trigo^1^**

*^1^ Escola Bahiana de Medicina e Saúde Pública - Salvador - BA - Brasil.*

**Objective:** A medical career requires long preparation from young people, which often involves immersion in a preparatory course for college entrance exams, taking a long university medical college and a few years of specialization through medical residency. Therefore, the tendency to postpone family planning leads to the emergence of social dilemmas and challenges arising from the need to acquire financial and professional stability in the life that precedes pregnancy. The present investigation aimed to evaluate the knowledge of medical students in the metropolitan region of Salvador, regarding the possible challenges facing late reproductive planning.

**Methods:** cross-sectional and descriptive study, developed in accordance with the current regulations, submitted to the Research Ethics Committee and approved through the consolidated opinion number 5.238.222. The convenience sample composed of 216 medical students from the metropolitan region of Salvador aged between 18 and 40 years old was evaluated through the application of a questionnaire in Google Forms, containing 12 initial questions about the profile of the students, and 9 questions about the knowledge of the topic being discussed. Sociodemographic inquiries (marital status, age, age that intends to have children, the first or the next; and age that will graduate); regarding interest in future residencies/specializations and their approximate duration; about carrying out tests to investigate fertility and the use of contraceptive methods were also discussed. The participants received an awareness booklet on reproductive aspects, associated with advanced age and assisted reproduction to read, in order to generate increased knowledge and possible changes in behavior regarding the reproductive planning of the medicine school student in question.

**Results:** Most participants were single (95.83%), without children (98.61%), using contraceptive methods (72.69%) and intending to have children in the future (85.65%). Students of the 7th semester of the course (26%), with the objective of doing medical residency (95.4%) with a duration of 3 years (45.9%) were the most predominant; 98.6% of the participants never underwent tests to investigate their ovarian reserve. In the second stage of the questionnaire, the participants demonstrated, in general, expertise on the proposed topic; of the 9 questions asked, 8 were mostly answered correctly. Predominantly, the participants are aware of the negative influence of aging on female fertility; know that assisted reproduction procedures may not fully compensate for an age-dependent loss of fertility; understand that assisted reproduction procedures can be performed exceptionally in women over 50 years old; are aware that in women over 40 years of age, there is a reduction in implantation rates and an increase in spontaneous abortions, low response to ovarian stimulation and poor quality of the collected oocytes; they also understand the process of follicular atresia. In addition, the students showed that they understood the proportion, in numbers, of follicle loss with advancing age and expressed knowledge about the need, especially for women aged 35 and over, to pay attention to ovarian reserve tests and consider the preservation of fertility through egg freezing. However, the academics showed a lack of informational regarding the percentage drop, measured at 50%, in fertility in the age range between 20 and 30 years, when they erroneously stated that it was around 10%.

**Conclusion:** Despite confirming knowledge about the decrease in female fertility due to aging, the generation of medicine school students was unaware of the magnitude of this impact. This panorama may denote future challenges due to the age group that intends to start planning to become pregnant.

**P-170. Does Atosiban administration in embryo transfer context improve pregnancy rate?**

**Amanda Ferreira Vigo^1^, Michele Queiroz Balech^1^, Ludmila Bercaire^1^, Luiza Barbosa Brandao^1^, Nilka F Donadio^1^, Artur Dzik1, Gilberto Freitas^1^**

*^1^ Hospital da Mulher - Sao Paulo - SP - Brasil.*

**Objective:** The primary endpoint is to determine if the administration of oxytocin antagonist (Atosiban) before embryo transfer (ET) may improve reproductive outcomes, and who are the patients eligible.

**Methods:** Searched electronic databases Pubmed, LILACS, Cochrane using MeSH terms (Keywords): embryo transfer, Atosiban. A total number of 32 articles were found and 8 were included. All randomized controlled trials (RCTs) that evaluated use of Atosiban before ET in women undergoing in vitro fertilization (IVF) cycles.

Atosiban dose administered varied in studies, most of them applied an initial bolus of 6.75 mg, and the infusion was continued with 18 mg/h for 1 hour, but in some studies, it was maintained for the second hour at 6 mg/h until complete 37.5 mg.

**Results:** Atosiban is a uterine-especific oxitocine antagonist, used primarily for preterm labor. Some studies demonstrate that Atosiban may decrease uterine contractions and promote uterine receptivity. The main theory about improvement in pregnancy rate would be decrease in uterine hyperreactivity. Embryo implantation requires moderate uterine contractions and adequate endometrium blood supply. Excessive uterine contractions can reduce implantation rate in IVF cycles and even push the embryo to be expelled from uterine cavity. Ye He et al. observed higher uterine waves per minute in endometriotic patients. Clinical pregnancy rate per cycle and implantation rate per transfer were 58.3% and 41.0%, respectively, in the atosiban group, significantly higher than in control group (38.3% and 23.4%, respectively) (95% IC 1.08-4.68; P0.044). Yuan, C et al studied patients with previous difficult tranfer using Atosiban and only cryopreserved embryos. Clinical pregnancy rate per cycle and implantation rate per transfer in atosiban group (45.1% and 26.5%) were significantly higher than those of placebo group (15.6% and 9.7%, respectively; P < 0.05). A multicentric randomized double blind study with 800 subfertile women undergoing IVF treatment using a general population, didn’t found significant difference on live birth rate between atosiban and placebo groups (Ng, EH et al; Li et al). Tang CL et al compared atosiban in patients with recurrent implantation failure undergoing IVF treatment with fresh embryo and there wasn’t significant difference on live birth rate between atosiban and placebo groups (42.3% vs 35.1%, p=0.302, RR=1.206 (0.844-1.723). There weren’t significant differences between both groups in the positive pregnancy test, clinical pregnancy, ongoing pregnancy, miscarriage, multiple pregnancy, ectopic pregnancy, and implantation rates. Moraloglu, O et al on a randomized, placebo-controlled clinical study with 180 woman shown that clinical pregnancy rate (PR) per cycle and implantation rate per transfer were 46.7% and 20.4% in the atosiban-treated group, which were significantly higher than control group (28.9% and 12.6%, respectively, P = 0.01). Miscarriage rates of groups 1 and 2 were 16.7% and 24.4%, respectively (P = 0.01). Craciunas L et al compaired the use of others oxitocine antagonists as nolasiban (oral), barusiban (subcutaneous) and atosiban (intravenous): none of them could increase live birth rate or decrease miscarriage rates.

**Conclusion:** Good quality embryos and ideal intrauterine environment are basic determinants of IVF outcomes, but ET is the final and crucial physician-guided step. Use of Atosiban seems to increase pregnancy outcomes in assisted reproduction cycles in some specific patients: woman with endometriosis, previous difficult transfer (defined when intrauterine manipulation was required, transfer catheter was curved back, greater resistance was met, multiple attempts were necessary, the procedure was time consuming (>30 seconds), force was needed, or trauma occurred, even rarely, dilation was carried out) or in the context of frozen cycles. However, use of this strategy increases cycle value, length of hospital stay. Therefore, larger randomized studies are needed for better evaluation.

**P-171. Risks of congenital anomalies in Assisted Reproduction: A systematic review**

**Flávia Gabriela Tojal Hora^1^, Andréa Fortes Carvalho Barreto^1^, Nathalie da Cunha Caldas^1^, Paloma Lisboa de Souza^1^, Ana Flávia Faro Passos^1^, Laís Viana Aragão Almeida^1^**

*^1^ Universidade Tiradentes UNIT/SE - Aracaju - SE - Brasil.*

**Objective:** Evaluate the risks of congenital anomalies in assisted reproduction.

**Methods:** This is a systematic review using the PUBMED database, without restriction of dates and from the descriptor "malformation AND fertilization", in which 6,749 studies were obtained. Only articles in English were included, covering the theme of the risks of congenital anomalies in assisted reproduction through meta-analyses, which resulted in 69 studies. We excluded articles that did not use the methodology or used another language. Thus, after the selection, 12 articles relevant to the theme in question were included.

**Results:** Congenital anomalies are a group of structural or functional changes that occur in intrauterine life and can be detected before, during, or after birth. Malformations include structural, biochemical, chromosomal abnormalities, and genetic syndromes. Thus, it is important to evaluate the risks of these congenital malformations in the offspring of women who have undergone assisted reproduction techniques, in order to guide the next patients who will use these methods. Thus, from the analysis of the articles it was perceived a greater evidence of congenital malformations in artificial fertilization (IVF and ICSI) than in natural fertilization. However, when comparing the risks of malformations in babies after assisted reproduction, of any kind, there are still not enough studies to prove which has the greatest potential to develop malformations. However, even with the need for studies with better design to obtain more truthful results, it is clear that the most common malformations associated with assisted reproduction are urogenital, cardiovascular, ocular, auditory, cervical, musculoskeletal, respiratory, nervous, digestive and facial malformations, as well as cleft lip and palate.

**Conclusion:** Despite the need for more rigorous studies and more robust designs, the evidence points out that the risks of congenital malformations are higher in artificial fertilizations compared to natural methods, especially with regard to cardiovascular and genitourinary malformations, such as hypospadias and cryptorchidism.

**P-172. Time Between Vasectomy and Reversal: Does It Really Matter?**

**Filipe Tenorio Lira Neto^1^, Alexandre Barbosa Albuquerque^2^, Matheus Souza Nogueira^2^, Gabriel Cadide Melo^2^**

*^1^ Andros Recife - Recife - PE - Brasil.*

*^2^ IMIP - Recife - PE - Brasil.*

**Objective:** To evaluate the impact of the time since vasectomy in the outcomes of vasectomy reversal

**Methods:** Using a prospectively maintained database, we identified men who underwent microsurgical vasectomy reversal by a single male fertility specialist from June 2016 to May 2023. We included men who had at least one postoperative semen analysis. The participants were divided into 3 groups based on the time since vasectomy: Group 1 less than 5 years, Group 2 between 5 and 10 years, and Group 3 more than 10 years. Baseline demographic and clinical characteristics, type of procedure performed, patency rates, and total postoperative motile sperm count (TMSC) were the variables compared among the groups.

**Results:** One hundred forty-four participants were included in the study, 41 in group 1, 61 in group 2 and 42 in group 3. The mean age of participants was significantly different among the groups, increasing as the time since vasectomy increased. The mean time since vasectomy was 4, 8 and 14 years for groups 1, 2 and 3 respectively (*P*<0.01). The baseline estradiol levels were higher in group 1 when compared to the other two groups. There were no other statistically significant differences between the groups regarding baseline characteristics (Table 1). Vasoepididymostomy was required in 13% of the participants in group 1, in 31% of group 2, and in 50% of group 3 (*P*<0.01). The overall patency rate was 93%, 93% and 81% for groups 1, 2 and 3 respectively (*P*=0.07), while the patency rate of patients who had bilateral vasovasostomy was 97%, 100% and 94% for groups 1, 2 and 3 respectively (*P*=0.29). The patency rate of bilateral vasoepididymostomies was 50% in groups 1 and 2, and 70% in group 3 (*P*=0.50). The median TMSC count was 29 million for group 1, 17 million for group 2 and 13 million for group 3 (*P*=0.19). The were no differences among the groups regarding the median TMSC for vasovasostomies or vasoepididymostomies. In addition, the median TMSC was not different regardless of the type of procedure performed.

**Conclusion:** Vasectomy reversal is a procedure with a high patency rate, regardless of the time of obstruction, but the longer the time since vasectomy, the greater the probability of the need for a vasoepididymostomy. Surgeons who perform vasectomy reversal should be able to perform vasoepididymostomy to achieve high success rates. In addition, the obstruction interval does not impact on the TMSC after vasectomy reversal.

**Table 1:** 

Parameter	< 5 years	5 - 10 years	> 10 years	p value
Number, n	41	61	42	
Age, mean (SD) years	37 (5)	41 (7)	46 (7)	<0.01
Female age, mean (SD) years	30 (6)	31 (5)	32 (4)	0.27
Testosterone (ng/dL), mean (SD)	457 (152)	411 (148)	439 (148)	0.2
Estradiol (pg/mL), mean (SD)	38 (17)	29 (10)	27 (8)	<0.01
FSH (mUI/mL), median (IQR)	4.4 (3.4, 6.7)	4.1 (3.1, 5.6)	2.8 (3.0, 5.6)	0.68
LH (mUI/mL), median (IQR)	3.9 (2.6, 6.7)	3.7 (2.8, 5.0)	3.7 (2.7, 4.2)	0.26
Vasoepididymostomy (%)	13%	31%	50%	<0.01
Overall total progressive motile sperm count, median (IIQ) millions	29 (4, 63)	17 (8, 41)	13 (3, 35)	0.19
Bilateral vasovasostomy total progressive motile sperm count, median (IIQ) millions	30 (9, 59)	16 (6, 46)	13 (2, 54)	0.29
Bilateral vasoepididymostomy total progressive motile sperm count, median (IIQ) millions	300 (300, 300)	14 (8, 45)	10 (2, 27)	0.14

**P-173. Fertility preservation of oncological patients assisted in a public assisted reproduction center in Natal-RN, Brazil**

**Danielle Barbosa Morais^1^, Délis Oliveira Ferreira^1^, Gabriel Ribeiro Souza^1^, Francielton da Silva Lima^1^, Karina da Silva Maranhão^1^**

*^1^ Universidade Federal do Rio Grande do Norte - Natal - RN - Brasil.*

**Objective:** Cancer is one of the diseases that most lead to death in the world, and the severity of the disease can directly affect the reproductive capacity of affected individuals. Although the use of fertility preservation techniques is not new, it is a possibility that is still little known by a large part of the population. It is also noteworthy that in Brazil this is a possibility available even in the Unified Health System (SUS), through the associated Clinics and Assisted Reproduction Centers; as is the case of the Assisted Reproduction Center (CRA) of the Januário Cicco Maternity School (MEJC), located in the city of Natal-RN, Brazil, and linked to the Federal University of Rio Grande do Norte (UFRN), and to the Brazilian Hospital Services (EBSERH). In this sense, this study aimed to characterize the profile of cancer patients submitted to fertility preservation techniques at CRA/MEJC.

**Methods:** After approval by the UFRN Research Ethics Committee (CEP) and the National Research Ethics Committee (CONEP), a descriptive and retrospective observational study was carried out, based on secondary data from patients who met the eligibility criteria for treatment at CRA/MEJC, between 2013 and 2018. Information was then collected about the socioeconomic profile and clinical data of patients who consented to access data from their medical records.

**Results:** From a sample of 326 patients, 280 authorized the access of its medical records. Approximately 78% of these patients were from brown race, followed by 19% white and 0.4% black. As for the level of education, 14% and 50%, respectively, had completed primary or secondary education, 26% had completed higher education and 6% had incomplete higher education, when they started their treatment at CRA. These patients had an average age of 35.5 years at the time of their treatment, and about 63% had primary infertility, while 36% had secondary infertility. Among the medical records analyzed, 06 patients were submitted to fertility preservation procedures due to cancer, which represented less than 3% of the sample. All had primary infertility and ages between 23 and 37 years (mean of 27 years). Of these, 03 patients had Hodgin's Lymphoma, 01 had Non-Hodgkin's Lymphoma, 01 had Breast Cancer associated with Squamous Cell Carcinoma and 01 had Fibromyxoid Ingnal Sarcoma. One patient, carrier of Hodking's Lymphoma, also had associated severe male factor (Teratospermia) on its partner, and in this case embryonic cryopreservation was performed. The other patients underwent oocyte cryopreservation. Until the moment this study was carried out, no information was available about the return of these patients to continue their Assisted Human Reproduction (AHR) treatments after cancer treatment.

**Conclusion:** The low percentage of patients who resorted to fertility preservation may be associated with the general population's lack of knowledge about this possibility, where it is worth mentioning the fact that cancer patients have priority in the waiting list for treatment at the aforementioned CRA, given the severity of the disease and the high demand that this public center receives. Also noteworthy is the difficulty in obtaining information about the return of these patients to continue the treatment, which may be a direct reflection of the high mortality associated with cancer. This study demonstrates the great contribution of a public AHR center to allow cancer patients of any socioeconomic status to maintain the dream of obtaining a pregnancy even after cancer treatment. It also raises reflections on ethical issues related to the posthumous use of cryopreserved gametes, tissues or embryos.

**P-174. Evaluation of interferon beta and inflamossome AIM2 protein expression in deep endometriosis lesion**

**Vitória Souza Rocha^1^, Helena Malvezzi^1^, Sergio Podgaec^1^**

*^1^ Centro de Ensino e Pesquisa Albert Einstein - São Paulo - SP - Brasil.*

**Objective:** Endometriosis is described as an estrogen dependent and progesterone resistant inflammatory disease, categorized into four stages (minimal, mild, moderate and severe). Endometriosis is characterized by the presence of eutopic tissue outside the uterine cavity, in which, accompanied by a failure of the innate and adaptative immune system, acts on the release of pro-inflammatory cytokines, leading to endometrial cells to implant and proliferate in the peritoneal cavity and organs, resulting in endometriosis development and progression. Previous studies have already described an increase in the inflammatory response related to downregulation of some protein markers, such as interferon. Recently, it was found a link between the inflammasome complex gene expression and endometriosis. Therefore, the aim of this experimental project is to evaluate the protein expression of interferon beta type 1 (INF-1) and inflammasome absent in melanoma 2 (AIM2) in endometriotic lesions and compare it with eutopic endometrium and non-endometriosis endometrium. This study was approved by the project management for research - SGPP (3617-18), has a certificate of presentation of ethics assessment (CAE 05776919.3.0000.0071) and grants 2019/24351, São Paulo Research Foundation (FAPESP).

**Methods:** For the development of this project immunofluorescence reaction was used in samples of endometrial tissues from patients with and without endometriosis and in lesions of deep endometriosis. The patients were divided between endometriosis group (10 eutopic endometrium and 10 lesions) and control group (10 non-endometriosis endometrium). The selection criteria encompassed women between 18 and 50 years old that were submitted to laparoscopy surgery for endometriosis excision or pain evaluation and were excluded patients with autoimmune diseases, infections or neoplastic diseases. For the immunofluorescence reaction primary antibody used were anti-INF-1 1:150 (BS-0787R - Thermofisher, São Paulo, Brazil) 1 mg/ml 100uL, and the anti-AIM2 antibody 1:150 (MA5-38442 - Thermofisher, São Paulo, Brazil) 1mg/ml 100uL. The secondary antibody was 1:400 Alexa Fluor 488 (a11008 - Thermofisher, São Paulo, Brazil), and 1:400 Alexa Fluor 647 (ab150115 -Abcam, São Paulo, Brazil), for nucleus staining DAPI (D1306 - Thermofisher, São Paulo, Brazil) was used. Samples were immersed on ice during the surgery and freeze -80º Cwith the OCT tissue tek. Slices were cut with cryostat (Leica - RM2125 RTS, Buffalo Grove, IL United States of America) in a thickness of 5um. Immunofluorescesce reaction was analyzed by a Confocal microscopy (Zeiss AIM-SYSTEM 2501000723) and 9 images were taken per slide (Zen Black - Carl Zeiss 2.3 SP1 FP1 65 bit). To evaluate protein concentration, pixel concentration (ratio between INFB/DAPI and AIM2/DAPI) of each antibody was measured using Zen 2.5 blue edition (Carl Zeiss Microscopy GmbH, 2018).

**Results:** No difference between samples were observed for INF-B and AIM2 among groups (*p*>0.05).

**Conclusion:** Although IFNB1 mRNAwas described as elevated in endometriosis tissue and peritoneal fluid, its protein expression seems not to be altered in endometriotic lesions compared to eutopic and nonendometriosis endometrium. It’s important to state that INFB1 has a very short half-life detected inserum, which might be extrapolated to tissue, thus making harder to detect subtle differences between groups that however, would have great biological impact on cells behaver.

**P-175. Letrozole X Clomiphene for ovulation induction in women with Polycystic Ovary Syndrome: A systematized review**

**Laís Viana Aragão Almeida^1^, Ana Flavia Faro Passos^1^, Paloma Lisboa de Souza^1^, Andrea Fortes Carvalho Barreto^1^, Nathalie da Cunha Caldas^1^, Flavia Gabriela Tojal Hora^1^**

*^1^ Universidade Tiradentes- UNIT - Aracaju - SE - Brasil.*

**Objective:** To systematically evaluate in the literature the differences between letrozole and clomiphene for ovulation induction in women with Polycystic Ovary Syndrome (PCOS).

**Methods:** For the study, the VHL and PUBMED databases were consulted. In the selection of articles, the keywords “letrozole”, “clomiphene”, “ovulation” and “PCOS” were used, articulated with the Boolean operator “AND”. The filter “the last 10 years” was used. In VHL, 107 articles were found and in PUBMED, 107 were found. 5 articles from PUBMED and 3 from VHL were read according to the greatest alignment with the research proposal. The conduct used for data extraction was the analysis of the results of the searches carried out in the databases, this analysis was made from the reading of the articles.

**Results:** It was noticed that comparing the use of letrozole with clomiphene citrate in the treatment of infertile women with PCOS there is a higher probability of ovulation with letrozole. In studies to determine the efficacy and safety of letrozole compared to clomiphene citrate, pregnancy occurred in 29 of 50 patients in the letrozole group (58%) and in 24 of 51 patients in the clomiphene group (47%). Among women with PCOS in another data analysis, the probability of pregnancy was 43% for letrozole and 37% for clomiphene. Moreover, if we consider variables such as the probability of live birth, it is 32% for letrozole and 29% clomiphene, risk of multiple pregnancy was 19% letrozole for 9% clomiphene, risk of prematurity was 20% to 15%, the risk of small for gestational age 5% to 3%, the risk of hospitalization in the neonatal intensive care unit was 22% to 16% and the risk of congenital malformation from 8% to 2% among those with live birth. Therefore, when analyzing these data, it is notorious that the use of letrozole for women with PCOS, is more effective, it should be considered as a first-line pharmacological treatment for women with anovulation, because pregnancy rates are higher, the time of pregnancy and the chances of multiple pregnancy are lower. However, after analyzing the neonatal outcomes, the two treatments presented similar static data, requiring the individual analysis of each patient, since the hypothesis of higher risks in the use of clomiphene in relation to letrozole was not proven.

**Conclusion:** Studies have suggested a greater effectiveness of letrozole compared to clomiphene citrate, due to better ovulation induction in anovulatory women with PCOS, having increased pregnancy rates and a shorter time to achieve it.

**P-176. The influence of anabolic androgenic steroid abuse on male infertility**

**Cássia Fernanda dos Santos Rosa^1^, Luane Mascarenhas Magalhães^1^, Rebecca Schuster Dorea Leite^1^, Marianna Marques Rodrigues Dourado^1^, Lais Viana Aragão Almeida^1^, Matheus Porto Alves^1^, Gabriella Dória Monteiro Cardoso^1^**

*^1^ Tiradentes University - Aracaju - SE - Brasil.*

**Objective:** Analyze the correlation between anabolic androgenic steroid abuse to male infertility.

**Methods:** This is an integrative review of articles published in PubMed, Cochrane and SciELO databases in the last five years, using the following descriptors: "Infertility, Male", "testosterone congeners" and "Anabolic Androgenic Steroids". The inclusion criteria were articles written in Portuguese and English, with full text available electronically. Paid studies, performed on animals, duplicates and those that are not related to the theme were excluded.

**Results:** Two review articles were selected for analysis, according to the inclusion and exclusion criteria, and the first of them indicated that exogenous testosterone suppress the pituitary secretion of LH and FSH, which results in a decrease in testosterone concentrations within of the testis, promoting abnormal spermatogenesis. However, a pooled analysis of 30 studies revealed that the recovery of spermatogenesis after the cessation of exogenous testosterone use is uncertain. The probability of recovering a sperm density of at least 20 million/mL is 67% at 6 months after discontinuation, increasing to 90% at 12 months and reaching 100% at 24 months. Additionally, short-acting testosterone has been shown to have minimal impact on the pituitary axis and preserve spermatogenesis. Thus, testosterone gel is presented as a viable option to raise testosterone levels while maintaining fertility. A study observed that, in a group of 60 men who used 4.5% testosterone, administered at a dose of 125 µL, three times a day, through an intranasal pump, only 1 individual became azoospermic after 3 months of use, and three developed severe oligospermia (sperm density < 1 million/mL). In the second article, it was observed that users of Anabolic Androgenic Steroids (AAS) had a reduction in the baseline levels of LH and FSH, in addition to an increase in testosterone and estradiol, which led them to develop side effects such as acne, hair loss, gynecomastia, sexual dysfunction and reduced testicular volume. It was also observed, in users, the reduction of total sperm count and sperm morphology. The recovery of gonadotropin levels was seen from 13 to 24 weeks after AAS withdrawal, while endogenous testosterone serum levels are still reduced at 16 weeks. The time of exposure to AAS, as well as the dosages used and the type of preparations taken, can specifically affect the hypothalamic-pituitary-testicular axis recovery, and the substance may remain concentrated in the sperm for up to 24 months.

**Conclusion:** The abuse of these substances has a notorious negative influence on male fertility, mainly through the mechanism of testicular atrophy and azoospermia, which are not always reversible in the short term. For this reason, it is necessary to have a greater role in public health policies that reinforce, for the population, the harm caused by this indiscriminate use.

**P-177. Brazil in the world picture of maternal mortality and setback caused by the COVID-19 pandemic through a literature review**

**Alexandre Antônio de Lima Junior^1^, Fálba Bernadete Ramos dos Anjos^1^, Manuella Amlid Pimenta de Castro Cavalcanti Silva^1^, Adriana Fracasso^2^, Karollyne Skarlet Gomes da Silva^1^, Evandro Valentim da Silva^1^**

*^1^ Universidade Federal de Pernambuco - Recife - PE - Brasil.*

*^2^ Faculdade Integrada de Pernambuco - Recife - PE - Brasil.*

**Objective:** This work aims to conduct a literature review, focusing on Brazil in the global context of maternal mortality and the setback caused by the COVID-19 pandemic.

**Methods:** The research was carried out using the electronic databases PubMed, SciElo, and PAHO, with publications between the years 2017 and 2022, as well as bulletins and reports obtained from DATASUS, the Brazilian Obstetric Observatory, and the Pan American Health Organization. Articles, reports, and bulletins addressing aspects related to maternal mortality, its causes, and progress in Brazil were selected.

**Results:** The high rate of maternal mortality in the modern world is unacceptable, considering that most maternal deaths are preventable. The World Health Organization (WHO) and the United Nations (UN) have been working together with international agencies to establish ways to promote the reduction of causes and their impact on women's health, aiming at achieving the Sustainable Development Goals (SDGs) (Sociedade Beneficente Israelita Brasileira Albert Einstein, 2019). The main complications, accounting for almost 75% of all maternal deaths, include hypertension (pre-eclampsia and eclampsia), severe hemorrhages, and infections, particularly postpartum infections, as well as complications during childbirth and unsafe abortions (OPAS, 2020). Diseases like pre-eclampsia are among the most common pregnancy complications, and pregnant women with COVID-19 may have a higher likelihood of developing pre-eclampsia. This condition can lead to serious complications for both the mother and the fetus, such as renal and hepatic failure, premature placental abruption, and fetal death. Its incidence varies from 2% to 8% of pregnancies worldwide, responsible for about 14% of maternal deaths and 10% of fetal deaths (Stevens, 2018; Khalil, 2019; Di Mascio, 2020). During the COVID-19 pandemic, there was a 94% increase in maternal mortality in Brazil, pushing mortality rates back to levels seen two decades ago, as revealed by the United Nations Population Fund. Between 2000 and 2020, the global maternal mortality ratio decreased by 34.3% [WHO, UNICEF, UNFPA, 2021]. In Latin America, between 1990 and 2015, maternal mortality decreased by 16.4%, but from 2016 to 2020, there was a 15% increase. In 2022, the maternal mortality rate in Latin America and the Caribbean was 68 per 100,000 live births. Preliminary data from the Brazilian Obstetric Observatory indicate that Brazil had 1,252 maternal deaths in 2022, translating to a rate of 50.6 deaths per 100,000 births (WHO 2022).

The investigation of women of childbearing age (WCBA) deaths is one of the strategies used for maternal death surveillance, allowing the identification of unreported maternal deaths. Through this indicator, the total number of maternal deaths has been decreasing over the past decades, but starting in 2020, there has been an increase in the number of maternal deaths. It increased from 62,035 total maternal deaths in 2018, 62,683 in 2019, 71,879 in 2020, 94,826 in 2021, to 66,862 in 2022, as demonstrated by the Mortality Information System (SIM, 2023). Ministry of Health data and mappings showed that in Brazil, in 2016, the maternal mortality rate reached 64.4 per 100,000 live births. In 2017, it was 64.5; in 2018, it was 59.1; in 2019, the rate was 57.9; in 2020, it was 74.7, and in 2021, the rate was 117.4 per 100,000 live births. In 2021, during the pandemic, the mortality rate reached values similar to those seen in 1998 when the rate was 110.2 deaths (SIM, 2023).

**Conclusion:** Maternal mortality rate was on a declining trend due to various strategies aimed at improving women's health quality. Despite the COVID-19 pandemic, there are national and international perspectives for strengthening policies and actions to emphasize the importance of prenatal care in preventing pregnancy-related problems.

**P-178. Use of progesterone for recurrent abortion**

**Luane Mascarenhas Magalhães^1^, Cássia Fernanda dos Santos Rosa^1^, Marianna Rodrigues Marques Dourado^1^, Brenda Lima Meireles Martins^1^, Laís Viana Aragão Almeida^1^, Gabriella Dória Monteiro Cardoso^1^**

*^1^ Tiradentes University - Aracaju - SE - Brasil.*

**Objective:** Analyze the effectiveness of using progesterone in the treatment of recurrent abortion.

**Methods:** This is an integrative review of articles published in PubMed, Cochrane and SciELO databases in the last ten years, using the following descriptors: “Abortion, habitual”, “Progesterone” and “Recurrent abortion”. Paid studies, performed on animals, reviews and duplicates were excluded.

**Results:** Three studies filled out the inclusion criteria. The first one was multicentric, double-blind, placebo-controlled, randomized trial was conducted with 836 women divided into two groups, one designated to receive vaginal suppositories containing 400 mg of micronized progesterone and the other placebo. A live birth rate after 24 weeks of gestation of 65.8% x 63.3% was observed, with no significant increase in the pregnancy rate. The second randomized and comparative study evaluated the effectiveness of oral dydrogesterone 30 mg/day and vaginal progesterone 600 mg/day in 200 women. The successful continuation of a viable pregnancy at 24 weeks, as well as the term, was higher in patients receiving oral dydrogesterone compared to intravaginal progesterone (75 vs 70). However, despite the study demonstrating benefit in the use of progesterone, the difference between the two groups was not statistically significant (p=0.5267), not indicating a clear advantage of one treatment over the other. The third study, cohort, observational and prospective, was carried out with 116 women with a history of recurrent pregnancy loss, evaluating the use of vaginal micronized progesterone, prescribed at a dose of 100-200 mg every 12 hours, starting 3 days after the peak of LH (luteal onset). Therefore, it was observed that, in women with high expression of glandular epithelial nuclear cyclin E (nCyclinE) (> 20%) in the endometrial glands, the use of luteal-onset vaginal micronized progesterone was associated with a higher pregnancy success rate, with rates of 6% before its use and 69% in subsequent pregnancies.

**Conclusion:** There is still no consensus in the literature regarding the effectiveness of progesterone use among women with recurrent miscarriages. More comparative studies assessing dosage and route of administration are needed.

**P-179. The Fundamentals of postponing maternity and health education strategies for professionals in fertilization clinics**

**Laura Aires Cavalcante Leite^1^, Tairane Farias Lima^2^, Thaís Farias Lima^2^**

*^1^ Universidade Estadual da Paraíba - Campina Grande - PB - Brasil.*

*^2^ Unifacisa - Centro Universitário - Campina Grande - PB - Brasil.*

**Objective:** To analyze what are the factors that lead women of reproductive age to postpone motherhood and what strategies can be carried out by health professionals in Fertilization Clinics.

**Methods:** A search was carried out on a digital platform (Virtual Health Library - VHL), using Health Descriptors (DECS) entitled; Fertility Preservation; Birth rate; Fertility Clinics; Health education.

**Results:** The fertility rate, in the mid-2000s, placed Brazil below the reproduction level of its population. Among the factors that have contributed to the reduction in the fertility rate is the postponement of motherhood. Cunha, Rosa and Vasconcelos (2022) concluded in their research that women are postponing their first pregnancy and that the main reasons are women's aspirations in the labor market, a fact that, historically, is a struggle regarding wage inequalities compared to men. In addition, women who do not have offspring are more likely to survive, according to this study. Barcellos, Dantas and Féres-Carneiro (2022) also showed that conservatism, prejudices and machismo may have harmed the participants' post-separation period and possibly contributed to their resistance to establishing new relationships. It is, for the most part, in this context that patients arrive at the Fertilization Clinics. It is a fact that adherence to egg freezing treatment due to social factors is still low, inviting even more health professionals to pay attention to this public. The Ministry of Health designates as health education a systematic, continuous and permanent process that aims at training and developing the citizen's critical awareness, stimulating the search for collective solutions to the problems experienced and their "real participation" in the exercise of control Social. Individualized monitoring, including needs and anxieties, as well as qualified listening is one of the strategies that can be used. Knowledge breeds security. The explanation of the scientific evidence through the explanation of the physiological factors of ovarian aging, the natural pregnancy rates according to age and the greater chance of pregnancy with their own eggs in the future are dialogues that can be repeated as many times as the patient needs, in order to provide greater security. Projecting the future is also a barrier for many women. The fact that they do not currently have the desire to become pregnant makes the appreciation of fertility preservation even more distant for them. Dialogues that allow the patient this measurement certainly help in the process. Another point that may discourage patients from undertaking the treatment is the cost of the treatment, many view the treatment as something unattainable. The strategy of presenting available funding to patients allows for predictability in relation to their real possibilities.

**Conclusion:** The main barriers to postponing motherhood are female aspirations in the job market and difficulties in establishing new relationships. In view of this, strategies that bring a holistic view of this patient must be addressed by the Team. Some important points are: providing security regarding the treatment, passing on the availability of possibilities for carrying out the treatment and assistance in the future projection of the patient.

**P-180. Impacts of female age and life habits, associated with male factor, on the development of oocyte and embryos**

**Flávia Gabriela Tojal Hora^1^, Andréa Fortes Carvalho Barreto^1^, Nathalie da Cunha Caldas^1^, Paloma Lisboa de Souza^1^, Ana Flávia Faro Passos^1^, Laís Viana Aragão Almeida^1^**

*^1^ Federal University of Rio Grande do Norte - Natal - RN - Brasil.*

**Objective:** Several factors can alter the quantity and quality of oocytes and also of embryos produced in Assisted Human Reproduction (AHR) treatments, with emphasis on age and obesity in women, as well as the presence of infertility in men. In this sense, the objective of this study was to analyze the existing relationships between the oocyte and embryonic development of patients submitted to AHR treatment, with age and female life habits, and male factor infertility.

**Methods:** Data were collected from patients treated at the Assisted Reproduction Center of the Januário Cicco Maternity School (CRA/MEJC), Natal/RN, Brazil (Ethics committee of Federal University of Rio Grande do Norte, approval no 3.490.438), between 2013 and 2018, and who consented in the access of their medical records (n=280). The criteria for inclusion in this study were refined to participants with infertility due to anatomical factors, advanced age, obesity, and infertility of the couple due to male factors, reducing the final sample population of this study to 144 participants. To analyze the existence of a correlation between maternal age and BMI with embryonic development, cases in which there was a male factor as the etiology of infertility were excluded from the sample group, to reduce the influence of other factors on the quality of the embryo. Therefore, for these correlations, only the embryos of patients with infertility due to anatomical factors and no male factor were selected (n=94). Data analysis was performed using the SPSS software, and depending on the nature of the variables, simple linear regressions were performed with Spearman's coefficient, multiple linear regressions and means comparison test using the Mann-Whitney U test (p<0.05 significance), and descriptive statistical analysis to determine the socioeconomic profile of the sample group.

**Results:** Patients eligible for this study were 36 years old on average when they started their treatment, had completed high school (51%), were brown (74%) and overweight (54%). In 62% of cases, infertility was primary. A positive correlation was found between the patient's age and the number of oocytes collected, the number of grade 3 oocytes and the number of discarded oocytes. By correlating female age and embryonic development, a reduction in the symmetry of blastomeres according to embryonic development was observed in D2 and D3, in relation to age, as well as lower rates of cleavage and blastocysts. The increase in BMI in women was associated with a reduction in cleavage rates, with an increase in the amount of immature and discarded oocytes during treatment. By verifying whether the age and BMI of the patients, together, can predict the parameters of oocyte and embryonic quality, only the BMI proved to be statistically relevant. Among the male factors found in the partners of eligible patients for the present study, oligoasthenozoospermia stood out as an isolated factor, and varicocele associated with teratooligozoospermia as a combined factor. By comparing the parameters indicative of embryonic quality between the group with male factor infertility and the group without male factor, it was possible to verify that the group without male infertility had a greater number of embryos with symmetrical blastomeres and blastocysts. Regarding treatment success, it was found that there is an association between the presence of male factor and lower positive results for the betaHCG test.

**Conclusion:** The results of this study are in line with literature data that suggest that a woman's age and lifestyle can negatively affect female fertility, compromising oocyte and embryonic development. When male factor infertility is present, embryonic development is affected, reflecting a smaller number of embryos that reach the blastocyst stage and, consequently, lower levels of positive beta-HCG.

**P-181. Success and safety rates of techniques used in assisted human reproduction treatment**

**Layla Paiva Almeida^1^, Gabriel Ribeiro Souza^1^, Francielton Silva Lima^1^, Larissa Alves Honorato Ferreira^1^, Ruthnaldo Rodrigues Melo Lima^1^, Danielle Barbosa Morais^1^**

*^1^ Federal University of Rio Grande do Norte - Natal - RN - Brasil.*

**Objective:** The use of Assisted Human Reproduction (AHR) techniques is increasing, since in recent years there has been a popularization of family planning, greater knowledge about the possibilities of treating AHR, and an increase in research that has demonstrated the success of the procedures. Although considered safe, these techniques may present a percentage of failure resulting in miscarriages or congenital malformations, and these alterations constitute a vast field to be investigated. In this sense, the objective of this study was to evaluate the success and safety rates regarding the use of Assisted Human Reproduction techniques, based on the medical records of patients treated at the Assisted Reproduction Center (CRA) of the Januário Cicco Maternity School, Natal-RN, Brazil.

**Methods:** Upon approval by the UFRN Research Ethics Committee, the medical records of patients who consented to access data regarding the care they received at the CRA were analyzed. From an initial sample of 280 medical records, those whose patients dropped out or had not completed their treatment at the time of data collection, who had spontaneous pregnancies, who had a negative bHCG or who only performed fertility preservation were excluded; since the central objective of the study requires patients who, obligatorily, had positive bHCG from assisted reproduction treatment. Thus, the final sample number of this study was 89 patients. Biometric and clinical data were collected, such as body mass index (BMI), age, marital infertility factor present, AHR technique used, type of embryo transfer performed and characteristics of oocytes and embryos. A total of 13 variables were obtained, which were investigated for the existence or not of an association with the appearance of twin pregnancies, miscarriages, or congenital malformations, using the chi-square test or logistic regression.

**Results:** Among the analyzed variables, 12 showed no significant association with twin pregnancy or miscarriage, which were: the type of contraception used; the BMI; maternal age; as well as the type of pregnancy obtained (single or twin); the history of abortion; the number of oocytes and embryos obtained; the AHR technique used; the type of infertility (primary or secondary), the presence of female or male infertility, and the infertility factor (whether female or male). On the other hand, the type of embryo transfer performed showed a significant association with the appearance of abortion, in which the fresh transfer had a higher number of abortions as a result (X^2^(2)=9.723 and p=0.008). Of the medical records that made up the sample of this study, only one reported the occurrence of congenital malformation. In this case, the technique of in vitro fertilization (IVF) and fresh embryo transfer had been used. The patient was overweight, primary infertility, 35 years old, and had no history of previous miscarriages.

**Conclusion:** This study demonstrated the independence of the results of twin pregnancies and the number of abortions with the AHR procedures; and above all, the very low (or almost non-existent) relationship between these treatments and the occurrence of congenital malformations, reinforcing safety in the execution of assisted reproduction techniques for the health of the offspring. While most of the analyzed variables had no influence on the gestational complications reported in this study, the type of embryonic transfer performed was relevant in the abortion results, so that fresh transfers were associated with higher abortion rates. We also emphasize the need for further studies that investigate in depth the relationships between these variables, always focusing on the health of the offspring to be obtained, and based on the ethical principles that regulate RHA techniques and procedures.

